# Dose delivery uncertainties assessment in the field junction region of craniospinal irradiation with Volumetric Modulated Arc Therapy using a robustness index and experimental dose verification

**DOI:** 10.1371/journal.pone.0313260

**Published:** 2024-11-07

**Authors:** Vasiliki Peppa, Emmanouil Zoros, Antigoni Alexiou, George Pissakas, Pantelis Karaiskos

**Affiliations:** 1 Medical Physics Laboratory, Medical School, National and Kapodistrian University of Athens, Athens, Greece; 2 Department of Radiotherapy, Alexandra Hospital, Athens, Greece; Shiraz University of Medical Sciences, ISLAMIC REPUBLIC OF IRAN

## Abstract

Due to its inherent technical challenges, craniospinal irradiation (CSI) entails crucial considerations regarding plan complexity and robustness. The scope of this work was to establish and validate methods suitable for the evaluation of robustness, as well as for dose verification in CSI with VMAT. Five patients previously treated with CSI were retrospectively selected. For each patient, two technically different treatment plans were generated, based on the conventional (static overlap) and staggered (dynamic overlap) configuration. These techniques served as a benchmark to evaluate the potential of a metric proposed in this work, aimed at quantifying robustness, the Overlap Robustness Index (*ORI*). Furthermore, they were utilized to assess the suitability of two experimental methods relying on film dosimetry, as well as on Delta^4^ phantom for identifying sources of uncertainties in CSI applications. In accordance with the positional error simulation performed, the staggered approach yielded a statistically significant superior *ORI* value compared to the conventional one. Additionally, the strong correlation observed between the positional shift induced dose distribution changes and *ORI* results (Spearman’s r = -0.941, *p*-value < 0.001) demonstrated the sensitivity of *ORI* in detecting areas of steep dose gradients within the overlapping regions that could potentially compromise the quality of treatment. Concerning dose verification, analysis in terms of dose profiles revealed a superior dosimetric accuracy for the staggered technique relative to conventional for both film and Delta^4^ measurements. Film-based gamma index results showed that staggered technique outperformed the conventional for the majority of passing criteria considered, with differences in passing rates up to 8.1%. The two treatment techniques however, exhibited equivalent dose delivery accuracy for the clinically relevant passing criteria when Delta^4^ was employed, with passing rate differences less than 0.6%. Findings of this study revealed that *ORI* is suitable for quantifying robustness in CSI with VMAT, while radiochromic films appeared to be the best candidate for CSI dose verification in this work.

## Introduction

Craniospinal irradiation (CSI) constitutes a radiotherapy technique challenging in terms of planning and delivery due to the length of target volume, as well as the radiosensitivity of spinal cord. CSI performed with Volumetric Modulated Arc Therapy (VMAT) relies on arc sets of different isocenters that overlap in predefined regions, providing a homogenous dose distribution over the whole Planning Target Volume (PTV) length [1]. Dose homogeneity within the overlapping regions is achieved through the inverse treatment planning process by appropriately combining the penumbral regions of the adjacent arc sets. Robustness of treatment, however, could be compromised by the presence of steep dose gradients of the adjacent arc sets that optimizer may arrive, which would lead to overlapping areas susceptible to setup inaccuracies [2]. Moreover, the suspected beam model inaccuracy associated with the beam penumbrae [3], as well as the complexity of the arc sets matching within the overlapping regions [1] could affect the accuracy of dose delivery, rendering the need for augmented Quality Assurance (QA) procedures imperative.

A variety of CSI techniques aiming to reduce the dosimetric impact of positional errors in field junction areas have been proposed in the literature in an attempt to improve robustness of the treatment plans [1, 4–7]. In these studies, robustness was assessed posteriori based on a method proposed by Lomax [8]. In specific, different set-up error scenarios were simulated and the induced uncertainties in dose profiles of the adjacent arc sets, as well as in Dose Volume Histograms (DVHs) metrics of clinical interest were quantified. Although this method is well-established in CSI [1, 2, 4–7, 9], it requires an extensive implementation time that might poses challenges to its practical application in clinical settings. Nevertheless, metrics that enable an objective quantification of robustness in order to facilitate its effective evaluation and control during the treatment planning process are strongly encouraged in the literature [10, 11].

VMAT treatment plans in CSI may present an increased degree of modulation within the overlapping regions due to the potential high leaf sequence variability [12, 13] that the optimizer might end up for the Multi-Leaf Collimator (MLC) while matching the dose distributions of the adjacent arc sets, which could significantly affect the quality of treatment [10, 14]. Moreover, overlapping regions in VMAT irradiation techniques are created at large distances from the isocenters by combining the penumbrae of the adjacent arc sets, resulting in dose distributions that may suffer from dosimetric uncertainties associated with the beam model [15]. Despite this emerging knowledge, testing procedures appropriate to validate the dosimetric accuracy of Treatment Planning System (TPS) calculations within the challenging overlapping regions have been relatively understudied. Experimental works on dose verification in CSI are scarce [2, 5, 16, 17], intending mainly to analyze the treatment planning methodologies and their impact on robustness rather than investigating possible sources of uncertainties that could compromise the accuracy of delivery. Lee et al. [18] utilized various commonly available measurement arrays and ionization chamber measurements in order to assess their suitability for dose verification in CSI using a 3-isocenter IMRT technique. Monte Carlo simulation constitutes an independent tool for dose verification in VMAT techniques [19, 20] based on the patient CT data along with the MLC sequence files, however the effect of the mechanical limitations of the linac on the deliverability of the treatment plans, which play a crucial role in CSI due to the high degree of modulation within the overlapping regions, is not taken into consideration. On the other hand, an estimation of complexity within the overlapping regions of CSI through metrics suitable to assess the degree of dosimetric uncertainties associated with the linac mechanical parameters, although acknowledged [10, 11], has not been established in the literature. This could be attributed to the difficulty in the evaluation of relevant machine parameters [10] such as the aperture modulation [21], as well as the size and irregularity of beam apertures [22] within the specific region delimited by the overlap. Based on these assumptions, the experimental testing procedures can be considered the golden standard for dose verification within the overlapping regions of CSI with VMAT techniques that could also serve as a tool to manage complexity during the treatment planning process.

Two VMAT irradiation techniques, the conventional and staggered overlap technique [7], were employed in this study in order to assess robustness and verify the TPS dose within the overlapping regions of CSI. These techniques, which rely on a static and dynamic overlap configuration, respectively, were used as a benchmark to evaluate the potential of the robustness method developed in this work for identifying steep dose gradients that could lead to dose delivery accuracy sensitive to patient setup shifts. The conventional and staggered techniques were selected in this work since they have been evidenced to exhibit differences in robustness when subjected to junction errors [7]. Moreover, the distinct configuration of the adjacent arc sets between the two irradiation techniques within the overlapping regions is anticipated to exhibit differences in dose delivery accuracy, with the staggered approach potentially mitigating the dosimetric impact of uncertainties associated with the beam model at the penumbral regions [15]. To this end, a method was developed in order to define a metric suitable for the evaluation of robustness within the overlapping regions defined in CSI with VMAT. The sensitivity of the method was assessed through the comparison between the conventional and staggered overlap VMAT techniques in terms of the robustness metric determined, while it was validated by investigating the correlation between the robustness metric and corresponding results obtained from a positional error simulation analysis. In order to verify TPS dose within the overlapping regions, two experimental methods were developed based on film dosimetry, as well as on Delta^4^ phantom, an equipment commonly used in clinical settings. While it is acknowledged that film measurements and commercially available arrays have already been utilized in the literature for dose verification in CSI [2, 5, 16–18], the experimental measurements were included in this study in order to identify potential sources of uncertainties that might affect treatment plan complexity, towards developing a method for the comprehensive administration of robustness and complexity.

## Materials and methods

### Ethics

This retrospective study was approved by the Scientific Review Board of the General Hospital of Athens “Alexandra” (protocol code 756/19-10-2023 and date of approval: 24-10-2023). Informed consent for publication in written form was obtained from all patients involved in the study. Personally Identifiable Information (PII) was removed from Digital Imaging and Communications in Medicine (DICOM) files of each patient using Monaco® 6.1.2.0 (Elekta AB, Stockholm, Sweden) TPS. The authors had no access to information that could identify individual participants during or after data collection. The anonymized patient data were accessed by the authors once the study received approval from the Institutional Review Board on 24-10-2023.

### Evaluation of robustness

#### Patient selection and clinical treatment planning

**Fig 1:**
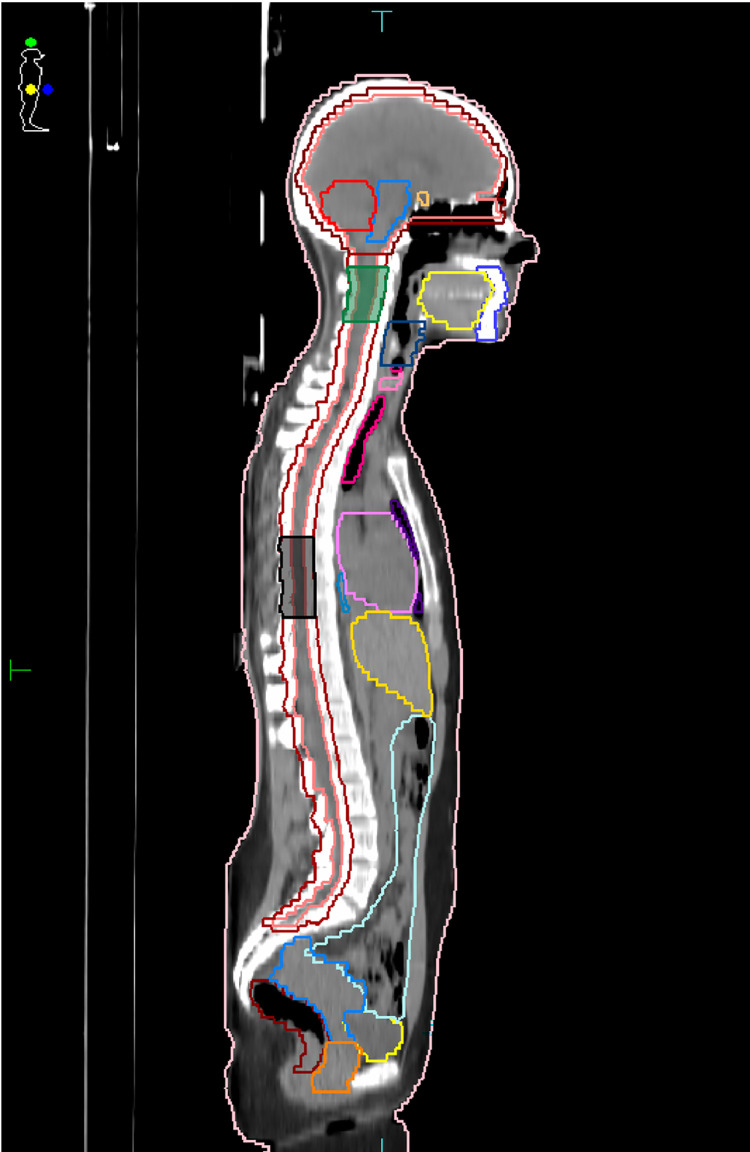
Print screen image from Monaco® TPS depicting the central sagittal slice of a representative patient along with the delineated structures including the PTV_upperOverlap_ (green) and PTV_lowerOverlap_ (black).

Five patients previously treated with CSI for medulloblastoma tumor in the Department of Radiotherapy of General Hospital of Athens “Alexandra” were retrospectively selected in this study. The patients were positioned supine with the head immobilized using a thermoplastic 5-point head, neck and shoulder mask along with a custom cushion. For each patient, the Computed Tomography (CT) scan was acquired with a 2.5 mm slice thickness and the CT images were imported to Monaco® 6.1.2.0 TPS to delineate the regions of interest. The Clinical Target Volume (CTV) included a brain and a spinal CTV, whereas the PTV was generated by applying margins of 5 and 7 mm to the brain and spinal CTV, respectively, resulting in PTV lengths in the superior-inferior direction ranging from 68.0 to 81.5 cm acrross all patients. In order to define the cranial-spinal and spinal-spinal overlapping regions, two special PTV structures ranging from 3 to 9 cm length, the PTV_upperOverlap_ and PTV_lowerOverlap_ (Fig 1), were also contoured [1, 2]. The Organs At Risk (OARs) included the lenses, eyes, optic nerves, parotids, oral cavity, larynx, esophagus, thyroid, lungs, heart, breasts, liver, spleen, kidneys, bladder, rectum, bowel bag and ovaries.

Two 6-MV VMAT plans, the static and dynamic, designed for the Infinity™ Linac (Elekta AB, Stockholm, Sweden) equipped with the Agility™ MLC (Elekta AB, Stockholm, Sweden) of 160 leaves (spatial resolution of 5 mm at isocenter) were retrospectively generated for each patient using the Monaco® 6.1.2.0 TPS. For both plans, three colinear isocenters were used, each accompanied by sets of four partial arcs, while a collimator angle of 0° was set to each arc (Table 1). The static and dynamic plan differed in the technique utilized to delimitate the overlapping regions. In specific, the adjacent arcs corresponding to a specific isocenter were set to overlap by a fixed length with each other in the static plan (conventional overlap technique), equal to the length of the considered overlap. For the dynamic plan (staggered overlap technique), the corresponding adjacent arcs were overlapped by staggering the field edges in a step size of 1/3, 1/4 or 1/6 of the overlapping length, with shorter steps utilized for longer overlaps [7]. For each patient, a dose of 36 Gy in 20 fractions was prescribed to the PTV. The planning objectives in the inverse treatment planning aimed a minimum dose of 95% of the prescribed dose to 98% of the PTV (D98% ≥ 95%) and a maximum dose of 105% of the prescribed dose to 2% of the PTV (D2% ≤ 105%). Special effort was made during the optimization process to achieve a homogenous dose within the overlapping regions, while reducing the dose to organs at risk to the minimum possible. Dose to medium in medium (D_m,m_) was calculated using the XVMC Monte Carlo dose engine [23, 24] with a statistical uncertainty lower than 1% within the PTV along with a dose grid resolution of 3 mm.

**Table 1:** Description of the arc arrangements (Upper Static (US), Upper Dynamic (UD), Mid Static (MS), Mid Dynamic (MD), Lower Static (LS), Lower Dynamic (LD)) involved in the delimitation of the PTV_upperOverlap_ and PTV_lowerlOverlap_ of the static and dynamic clinical treatment plans.

Number of isocenters	3
Number of overlaps	2
Number of arcs per isocenter	4
Start/Stop gantry angle per arc	Upper arcs (US/UD)
	(180–220°, 290–325°,
	35–70°, 140–180°)
	Mid arcs (MS/MD)
	(195–230°, 290–350°,
	35–70°, 140–170°)
	Lower arcs (LS/LD)
	(190–225°, 295–350°,
	30–65°, 140–170°)
Overlap length	3–9 cm

The clinical equivalence of the treatment plans was assessed in terms of DVH metrics relevant to PTV coverage and OARs sparing. Comparison between the irradiation techniques was also performed for the PTV_upperOverlap_ and the PTV_lowerOverlap_ using coverage metrics as well as the Heterogeneity Index (HI), expressed by the ratio of the minimum dose received by the hottest 5% of the tissue (D5%) to the minimum dose delivered to 95% of the tissue (D95%). The modulation degree, defined by Monaco TPS as the ratio of the total Monitor Units (MUs) to the mean segment MUs, as well as the total number of MUs were also recorded for each treatment plan.

#### Definition of the Overlap Robustness Index (ORI)

A method to assess the robustness within the overlapping regions in CSI with VMAT was developed in this work. This method is based on the evaluation of the dose gradient distribution along the longitudinal z-axis of the patient (dDdz) within the overlap that is associated with the adjacent arc sets. Taking into account that dose gradients of the adjacent arc sets are mainly generated by the optimization process along the z-axis of the patient, dDdz distribution could be assumed equivalent to dose gradient distribution within the overlap volume (dDdV). Based on this assumption, DVHs of the adjacent arc sets were utilized to determine robustness within the overlapping regions. In specific, dose was recalculated for the upper, mid and lower arc set of each clinical treatment plan and corresponding DVHs were exported from the TPS in text files (*.txt). The distribution of the DVH slope (dVdD)i along with its median value (dVdD)Median(i) was subsequently calculated for each arc set *i*. Ideally, DVHs of the arc sets would represent linear ramps to guarantee a smooth dose transition between isocenters, insensitive to possible misalignment errors [9]. To this end, a linear fit was applied to the DVH data of each arc set *i* using the equation:

%Volume(i)=(dVdD)Fit(i)×Dose(i)+b(i) (1)

The parameter (dVdD)Fit(i) obtained by this fit denoting the slope of the linear function, the coefficient of determination *R*_*i*_^2^, as well as the corresponding median DVH slope value (dVdD)Median(i) were utilized to define the linear coefficient $f_{{lin}_{i}}$, which describes the similarity of the DVH curve with a linear function as follows:

flini=Ri2×(dVdD)Median(i)(dVdD)Fit(i) (2)

The linear coefficient of the overlap is defined as the average *f*_*lin*_ over the two adjacent arc sets:

$$f_{lin}=\frac{1}{2}\sum_{i=1}^{2}f_{{lin}_{i}}$$ (3)

A value of 1.0 for the *f*_*lin*_ coefficient would indicate perfect linear ramp-like DVH curves for the adjacent arc sets.

To further consider that dose gradient distribution within the overlapping regions is associated with the overlap length, as well as with the total dose distributed within the overlap, the gradient coefficient, *f*_*grad*_, was also defined. The *f*_*grad*_ is the ratio of the average DVH slope over the two adjacent arc sets of the evaluated overlap of *h* cm length to the theoretical DVH slope of an arc set contributing to a 9 cm long overlap, that represents a typical extended overlap utilized in clinical settings [1, 7], considering perfect linear-ramp DVHs for each arc set. The gradient coefficient is calculated using the following formula:

$$f_{grad}=\frac{\frac{1}{2}\times \sum_{i=1}^{2}{DVH}_{slope}^{Overlap(h\;cm)}(i)}{{DVH}_{slope}^{{Overlap}_{theor}(9\;cm)}}$$ (4)

Considering a dose range from zero to the prescribed dose, *D*_*pr*,_ for the arc set forming the theoritical 9 cm overlap, and from D98%(i) to D2%(i) for each arc set *i* of the evaluated overlap, the equation above transforms into:

fgrad=12×∑i=12(Volume)Overlap(hcm)(D2%(i)−D98%(i))(Volume)Overlaptheor(9cm)Dpr (5)

Taking into account that each overlap could resemble a cylinder of radius *r* and length *h*, the equation above can be expressed as follows:

fgrad=12πr2h∑i=121(D2%(i)−D98%(i))πr29Dpr=h18×Dpr×∑i=121(D2%(i)−D98%(i)) (6)

The Overlap Robustness Index (*ORI*) is then defined as the product of the linear and gradient coefficients:

ORI=flin×fgrad=h×Dpr36×∑i=12Ri2×(dVdD)Median(i)(dVdD)Fit(i)×∑i=121(D2%(i)−D98%(i)) (7)

#### Evaluation of the ORI performance

The potential of the method developed in this work for the evaluation of robustness in overlapping regions of CSI with VMAT was assessed by comparing the two clinical treatment plans in terms of *ORI*. It should be noticed that *ORI* was calculated for each patient using a custom routine developed in MATLAB R2020b (The MathWorks Inc., Natick, MA). Comparisons were made for an indicative case, as well as for the patient cohort. The predicitve power of *ORI* on robustness was further assessed across all patients, by correlating the *ORI* results with corresponding data associated with different setup error scenarios. In specific, a simulation of ±3 mm shift was applied to the mid isocenter of each treatment plan along the cranial-caudal direction and changes in homogeneity within each overlap due to the convergence and divergence of the isocenters were evaluated by means of the ratio D2%D98% with and without the applied shift,(D2%/D98%)±3mm(D2%/D98%)0mm, hereinafter refered to as Inhomogeneity Ratio (*IR*_*(±3mm/0mm)*_). The ±3 mm shift was selected since it could mimic a realistic setup error associated with the treatment couch sag, calibration uncertainties, as well as the patient intrafraction motion. It should be noted that, in accordance with the literature [2, 4, 7], variations beyond rigid setup errors, including non-rigid deformations, anatomical changes, or intrafraction organ motion, were recognized as having a minor impact on robustness in the overlapping regions of CSI techniques, and thus they were not considered in this study. Differences in *ORI* and *IR*_*(±3mm/0mm)*_ results between the static and dynamic treatment plans across the patient cohort were evaluated for statistical significance using a paired sample Wilcoxon signed rank test with a significance criterion of *p* ≤ 0.05, while the *ORI* values were correlated with the *IR*_*(±3mm/0mm)*_ results by calculating the Spearman’s rank correlation coefficient along with the corresponding *p*-value. It should be noted that the specific statistical tests were selected since the data were not normally distributed, as determined by the Kolmogorov-Smirnov test. Statistical analysis was performed using MATLAB R2020b (The MathWorks Inc., Natick, MA).

#### Reccomendations on tolerance and action limits for ORI

Although the patient sample used in this study was too small to thoroughly evaluate tolerance and action limits for *ORI*, an effort was made to establish clinically relevant limits. Based on the (*D*2%/*D*98%)_0*mm*_ ratio, which is nominally equal to 105%/95%, as well as the accepted 5% dosimetric delivery accuracy in external beam radiotherapy [25], a ratio value of 110%/90% for the (*D*2%/*D*98%)_±3*mm*_ was considered within tolerance. This assumption corresponds to a tolerance limit of 1.11 for the *IR*_*(±3mm/0mm)*_. Taking into account that the D2% tolerance for the spinal cord equals 45Gy (125% relative isodose level in this study) [26], the action limit values of 125%/90% and 1.26 were assigned to the (*D*2%/*D*98%)_±3*mm*_ and *IR*_*(±3mm/0mm)*_, respectively. The corresponding tolerance and action limits for *ORI* were obtained by fitting a power function *a*×*x*^*b*^ to the *IR*_*(±3mm/0mm)*_ and *ORI* data.

### Experimental dose verification

#### Phantoms and dosimeters

**Fig 2:**
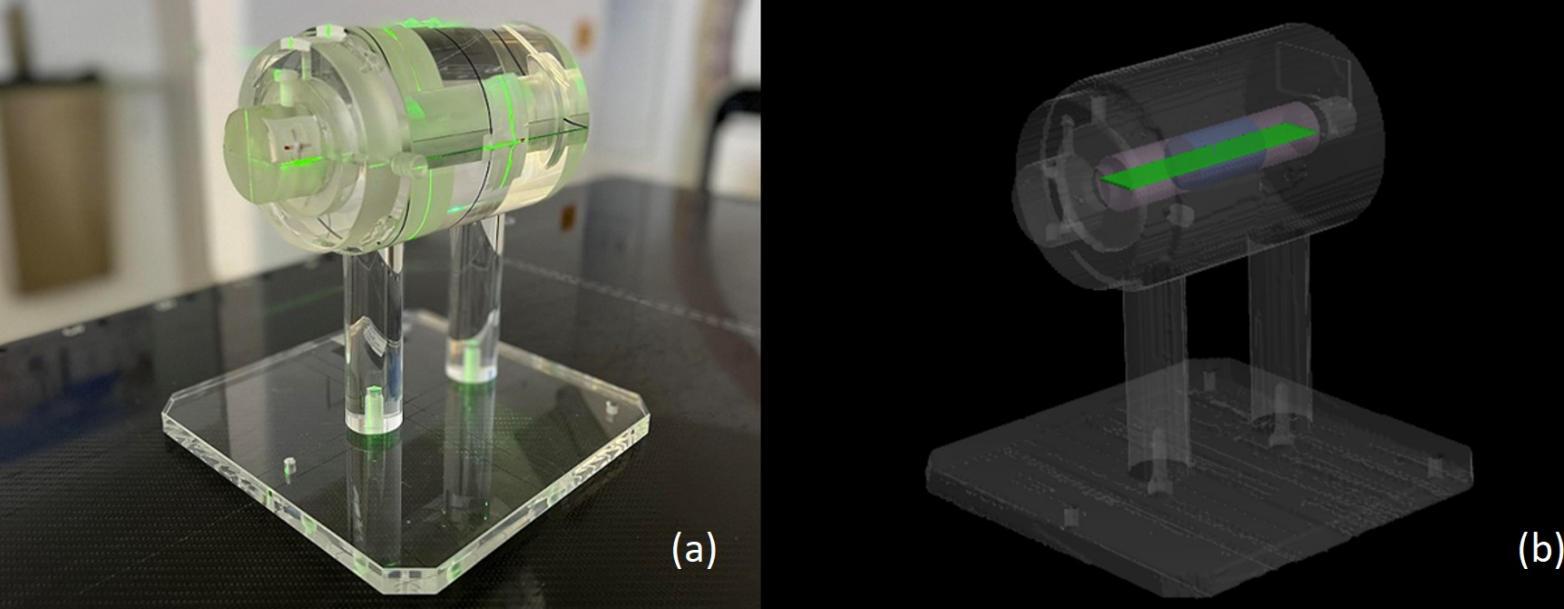
(a) Picture of the PMMA phantom incorporating the EBT3 radiochromic film. (b) Print screen image from Monaco TPS showing a 3D reconstruction of the PTV (pink), PTV_experimentalOverlap_ (blue) and film (green) contours created on the phantom.

In order to verify the delivered dose in the overlapping regions, the cylindrical polymethyl methacrylate (PMMA) physical phantom (IBA Dosimetry, Schwarzenbruck, Germany) of approximately 12.5 cm length and 9.7 cm diameter was used to replicate a patient geometry (Fig 2A). For this phantom, a custom cylindrical PMMA insert of 13 cm length and 2.1 cm diameter was constructed, which allows for placement of EBT3 films. Three fiducial markers defining the geometrical center of the phantom were placed at the surface of the phantom, while two fiducial markers were added to the surface of the cylindrical insert to facilitate the horizontal alignment of the film. Three metal pins were embedded to the cylindrical insert to ensure stability and reproducibility of the films during the irradiation, as well as for registration purposes. GAFchromicTM EBT3 films (Ashland Inc., Wayne, NJ) were employed in this work, which were cut in appropriate dimensions to fit the cylindrical insert. The film batch was calibrated at the Secondary Standard Dosimetry Laboratory of the Greek Atomic Energy Commission in a reference ^60^Co beam from a PICKER unit at doses in the range of 0.10–15 Gy. Given the negligible EBT3 relative energy dependence that is within 0.4% for the 6 MV irradiations relative to ^60^Co beam [27, 28], no energy dependence correction was applied in this work for the EBT3 films. Calibration film pieces of dimensions 3 × 3 cm^2^ were irradiated at a depth of 5 cm in an RW3 solid slab phantom. Experimental verification of the dose in the overlapping regions was also performed using the Delta^4^ phantom (ScanDidos, Sweden), which incorporates 1069 p-type silicon diodes built in two orthogonal planes with a spatial resolution of 0.5 cm at the central 6 × 6 cm^2^ area and 1 cm at the outer area.

#### Phantom treatment planning

The cylindrical phantom incorporating a dummy EBT3 film in the coronal plane was CT scanned using a reconstruction voxel of 0.98 × 0.98 × 1.25 mm^3^. Images were imported to Monaco® 6.1.2.0 TPS and a structure set mimicking a real CSI case was generated. This included a cylindrical PTV of 11.5 cm length and 2.5 cm diameter, as well as an overlapping region of 4 cm length and 2.5 cm diameter (PTV_experimentalOverlap_), which resembles a clinical overlap. It should be noted that the geometrical center of the PTV_experimentalOverlap_ coincided with the center of the phantom defined by the fiducial markers. The dummy EBT3 film as well as the cylindrical phantom excluding air cavities were also outlined. A 3D reconstruction of the CT images along with the structure set created can be seen in Fig 2B. A nominal relative electron density (RED) of 1.161 was assigned to the phantom to match the nominal PMMA mass density of 1.19 g/cc, according to the formulas used by Monaco® TPS to convert RED to mass density, whereas a RED value of 1 was assigned to the film structure.

Two treatment plans were performed for the PMMA phantom on the basis of the clinical static and dynamic treatment plans. In order to be able to perform TPS dose calculations on the PMMA phantom, the distance of the upper and mid isocenters used for the clinical plans was appropriately adjusted to fit the phantom. In specific, two isocenters located 8 cm apart were placed on the longitudinal axis of the PTV on either side of the PTV_experimentalOverlap_. The arc arrangements utilized for the upper and mid isocenter of the clinical cases were applied to the PTV_experimentalOverlap_ using the clinical conventional and staggered overlap techniques (Table 1). A dose of 36 Gy in 20 fractions was prescribed to the PTV with planning objectives in the inverse planning process similar to the ones used for the clinical cases, while pushing the optimizer to achieve a homogenous dose distribution within the PTV_experimentalOverlap_. Dose to medium in medium (D_m,m_) was calculated with the XVMC Monte Carlo code, using a dose grid resolution of 1 mm along with a statistical uncertainty within the PTV lower than 0.5%. For comparison purposes with the experimental results, dose was recalculated for each treatment plan separately for the upper and mid isocenter using the corresponding upper and mid arc sets (Upper Static (US), Upper Dynamic (UD), Mid Static (MS), Mid Dynamic (MD)), respectively.

Dose was recalculated for the US, MS, UD and MD arc sets on the Delta^4^ phantom using dose to water in medium (D_w,m_) reporting and the XVMC Monte Carlo code with a grid resolution of 1 mm and a statistical uncertainty of 0.5%. It should be noted that dose calculation relative to US/UD and MS/MD arc sets was performed by applying a shift of 4 cm to Delta^4^ phantom towards the caudal and cranial direction, respectively, so that the dose within the PTV_experimentalOverlap_ will be calculated on the central region of the two orthogonal detector planes where the spatial resolution of the silicon diodes is finer.

#### Irradiations

Four irradiations (US, MS, UD, MD) were performed in the Infinity™ Linac with the PMMA phantom incorporating the EBT3 films. Guided by the fiducial markers, the phantom was initially placed with its geometrical center coincide with the isocenter, whereas the film was horizontally aligned. A shift of 4 cm was applied to the treatment couch towards the caudal and cranial direction to perform the US/UD and MS/MD irradiations, respectively. The position of the PMMA phantom in each irradiation was verified by Cone Beam CT (CBCT) using the 3D X-Ray Volume Imaging (XVI) tool of the Infinity™ Linac. The placement of the EBT3 film within the phantom was optimized through co-registration of the CT image stack and CBCT using the three metal pins. The US, MS, UD and MD irradiations were also performed using the Delta^4^ phantom, which was appropriately displaced for each irradiation to match the corresponding TPS dose calculation.

#### Film handling, dosimetry and uncertainties

Each film piece in this study was treated following the guidelines outlined in the AAPM TG-235 report [29]. The dose read-out for both the calibration and experimental film pieces was preformed 24 hours after irradiation using the EPSON Perfection V850 Pro flatbed optical scanner in transmission mode. During the scanning process, all filters and image enhancement options were disabled, and the films were placed in landscape orientation with a 3 mm thick glass compression plate [30].

RGB positive images with a depth of 48 bits were acquired with a spatial resolution of 150 dpi, resulting in a pixel size of 0.169 mm. These images were saved in a tagged image file format (TIFF). To obtain dose values, the acquired pixel values of each film piece were converted using the calibration curve for the red color within the framework of the single-channel film dosimetry protocol [31].

For spatial registration, the dose map of each film was co-registered with the corresponding dose distributions calculated by the TPS. Metal pins of the insert and the corresponding holes in the film pieces were used as control points during the spatial registration process.

The uncertainties associated with the film measurements were estimated following the Guide to the Expression of Uncertainty in Measurement (GUM) [32], considering calibration, scanning, and spatial registration procedures as sources of uncertainty. A total uncertainty of 1.5% was calculated for doses ranging from 1 to 2 Gy, using a confidence level of 68% (k = 1) whereas for lower dose values it was on the order of 3%. Specifically, calibration data contributed to the type B uncertainty, including uncertainties from the calibration fit parameters and the delivered dose values during calibration. Type A uncertainties related to optical density measurements were assessed by performing consecutive scans to evaluate optical density reproducibility. The type B uncertainty associated with the optical scanner’s homogeneity was considered based on existing literature [33]. The uncertainty in spatial registration, arising from the co-registration of the film-measured dose distribution with the TPS-calculated dose distribution, was estimated to be 0.5 mm, corresponding to half the slice thickness of the phantom’s CT dataset.

#### Data analysis

The TPS calculated and corresponding EBT3 film and Delta^4^ measured dose distributions for each irradiation were compared within the PTV_experimentalOverlap_ using the global Gamma Index (GI) test [34] along with the clinically relevant passing criteria of 3%/2mm and 3%/3mm determined by the recommendations of TG-218 report [35]. Tighter criteria of 2%/2mm and 3%/1mm estimated from the uncertainty analysis were also applied, to detect subtle regional errors. It should be noted that TPS calculations always served as the reference dataset for the GI analysis conducted for EBT3 films for consistency purposes with the GI analysis implemented by the software of Delta^4^. Comparisons were also performed in terms of 1D dose profiles, as well as percentage dosimetric differences along the boundaries of the PTV_experimentalOverlap_ where the dose originated from the beam edges of the adjacent arc sets, associated with a degraded beam modelling accuracy [3], is superimposed.

## Results

### Evaluation of the treatment plans

The clinical criteria set during the inverse treatment planning were met in both clinical treatment plans, while the static and dynamic plans were found to be clinically equivalent in PTV coverage and OARs sparing. Quantitative evaluation of the clinical treatment plans by means of modulation degree, total treatment time and DVH metrics calculated for the PTV_upperOverlap_ and the PTV_lowerOverlap_ are summarized in Table 2. It can be seen that optimization process resulted in comparable treatment plans in terms of modulation degree, total MUs and homogeneity within the overlaps, with absolute differences in the median values of 0.2, 77, and 0.01, respectively. Regarding the coverage metrics calculated for the PTV_upperOverlap_ and the PTV_lowerOverlap_ of the clinical cases, the static and dynamic plan demonstrated nearly identical performance, with differences in the calculated median values up to 0.4%, lower than Monte Carlo Type A uncertainty. Similar to the clinical plans, the static and dynamic phantom treatment plans exhibited equivalence concerning modulation degree, total treatment time, homogeneity and coverage-based DVH indices within the PTV_experimentalOverlap_ with a maximum difference of 1% observed for the maximum dose.

**Table 2:** Plan evaluation parameters for the static and dynamic treatment plans calculated for the clinical cases and the PMMA phantom.

	Clinical treatment plans		QA treatment plans	
	Static	Dynamic	Static	Dynamic
	Median [min,max]	Median [min,max]		
Modulation degree	2.9 [2.3,3.0]	2.7 [2.2,2.8]	1.6	1.6
MUs	1422 [1352,1492]	1499 [1446,1517]	549	546
**PTV_upper/experimentalOverlap_**				
**Coverage**				
D98% (%)	96.5 [95.4,98.7]	96.6 [96.2,98.1]	98.5	98.9
D2% (%)	104.7 [102.8,105.0]	104.3 [103.9,104.9]	102.8	102.5
D_max_ (%)	109.0 [105.1,109.5]	108.6 [108.4,108.7]	105.1	104.1
**Homogeneity**				
HI	1.07 [1.05,1.09]	1.06 [1.05,1.08]	1.03	1.03
**PTV_lowerOverlap_**				
**Coverage**				
D98% (%)	96.6 [95.9,97.3]	96.8 [96.6,97.6]		
D2% (%)	103.9 [102.1,104.7]	104.2 [103.9,104.8]		
D_max_ (%)	108.6 [105.0,109.1]	108.9 [108.4,109.3]		
**Homogeneity**				
HI	1.06 [1.05,1.07]	1.05 [1.04,1.07]		

### Evaluation of the ORI performance

#### Indicative clinical case

**Fig 3:**
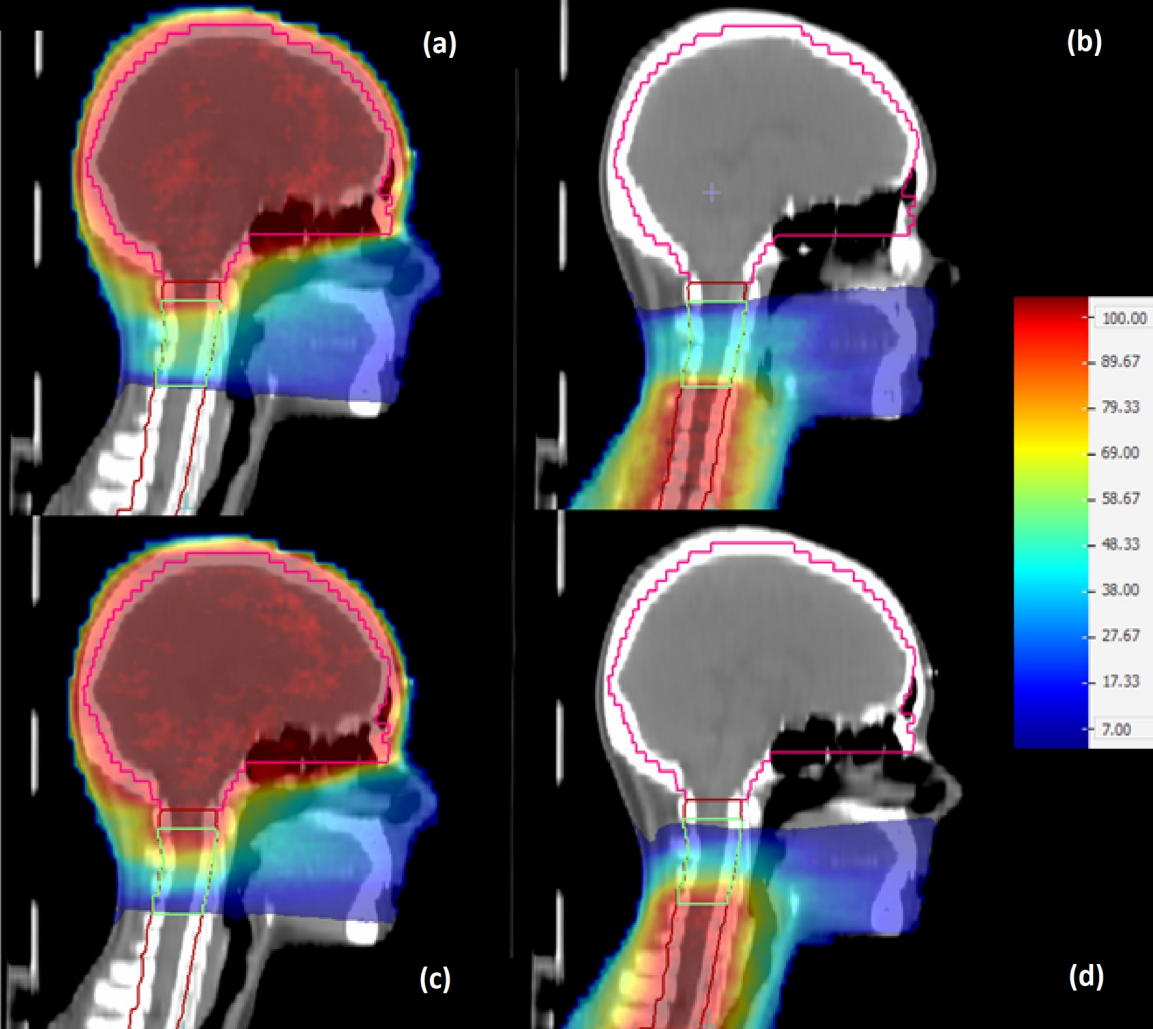
Print screen image from Monaco TPS depicting the central sagittal slice of the clinical case depicted in Fig 1 along with a colormap representation of the % dose distribution relative to the prescribed dose within the PTV_upperOverlap_ (green) associated with the (a) US, (b) MS, (c) UD and (d) MD arc sets.

**Fig 4:**
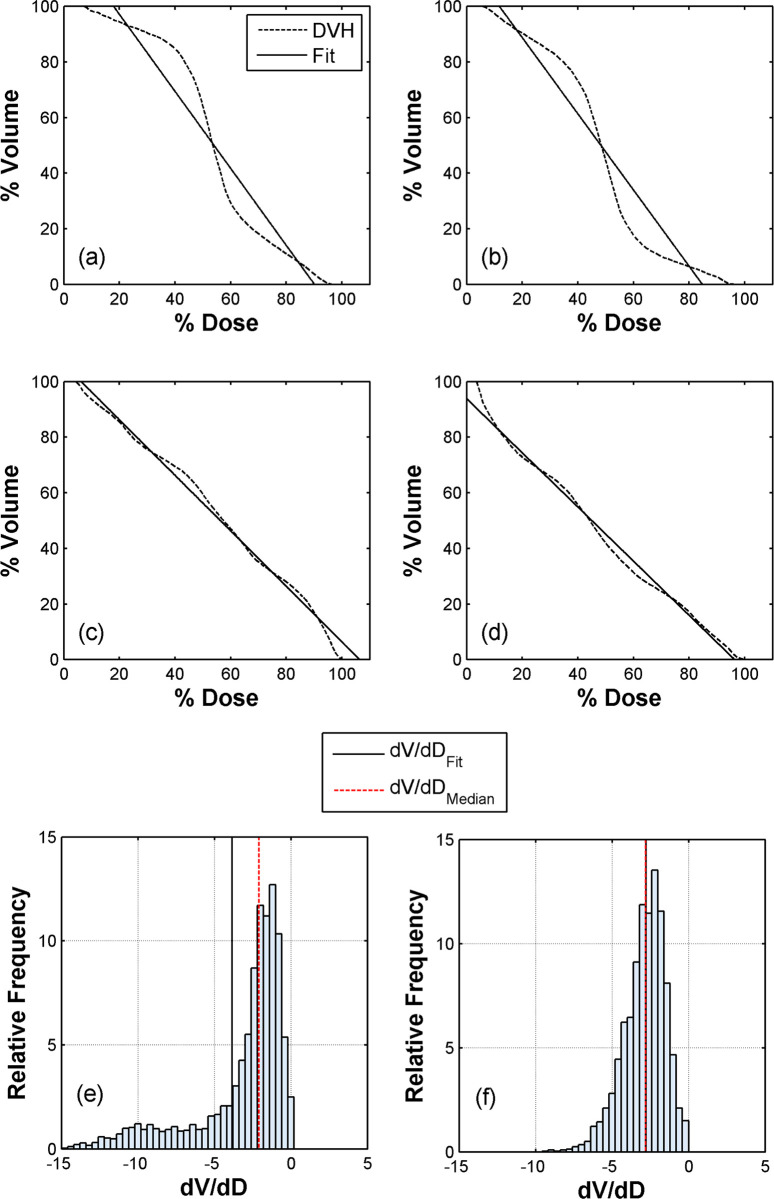
DVHs obtained from the clinical case for the (a) US, (b) MS, (c) UD and (d) MD arc sets within the PTV_upperOverlap_ (dashed lines) and the corresponding linear fits (solid lines). Indicative calculated histograms of the DVH slope distributions are also presented for the (e) US and (f) UD arc sets. The black solid and dashed red lines correspond to the (dVdD)Fit and (dVdD)Median values, respectively.

Fig 3 presents a colormap representation of the spatial dose distribution on the central sagittal slice of the clinical case shown in Fig 1, calculated within PTV_upperOverlap_ using the US, MS, UD and MD arc sets. A generally smoother dose transition was observed within the PTV_upperOverlap_ for the dynamic plan compared to static plan, where steep dose gradients can be seen close to the boundaries of the overlap. These observations agree with the findings in Fig 4A–4D, where the DVHs along with the corresponding linear fit functions are presented for the US, MS, UD and MD arc sets. Shallow and steep-sloped DVH regions corresponding to steep and low dose gradients within the PTV_upperOverlap_, respectively, were observed for the static plan, whereas an almost constant slope can be seen for the DVHs of the dynamic plan. The linear fit demonstrated an excellence performance for the dynamic plan with *R*-squared values of 0.99 for both UD and MD arc sets, whereas a poorer performance was observed for the static plan with resultant *R*-squared values for the US and MS arc sets of 0.94.

Indicative DVH slope distributions calculated for the US and UD arc sets are shown in Fig 4E and 4F. The static plan resulted in a significantly broader distribution compared to dynamic plan, with corresponding Root Mean Square (RMS) values of 4.42, 4.34, 3.18 and 3.27 for the US, MS, UD and MD arc sets, respectively. Table 3 presents the (dVdD)Fit values obtained by the fits along with the corresponding (dVdD)Median, *f_lin_, f_grad_* and *ORI* results. The static plan-based (dVdD)Fit results presented a shift towards higher values relative to the corresponding (dVdD)Median results, with a (dVdD)Median(dVdD)Fit ratio of 0.55 for both arc sets. For the dynamic plan, the (dVdD)Fit values were found in excellent agreement with the corresponding (dVdD)Median results, with (dVdD)Median(dVdD)Fit ratios of 1.01 and 0.96 for the UD and ΜD arc sets, respectively. The resultant *f*_*lin*_ value related to the static plan was equal to 0.51, demonstrating a major deviation from the linear model, whereas an almost ideal performance was seen for the dynamic plan, with a respective value of 0.98. The static and dynamic irradiation techniques yielded comparable gradient coefficient values on the order of 0.5, nevertheless the linear coefficient appeared to dominate the resultant *ORI* values, with the dynamic plan exhibiting a higher *ORI* value by approximately 0.2 compared to static plan.

**Table 3:** Calculated coefficients with the 95% confidence intervals (in the parentheses) of the linear fit applied to the DVH of each arc set within the PTV_upperOverlap_ of the clinical case depicted in Fig 1 along with the corresponding *R*-squared values. Median values of the DVH slope obtained for each arc set along with the corresponding *f*_*lin*,_ *f*_*grad*_ and *ORI* results are also presented.

		Linear fit results			(dVdD)Median	*f*_*lin*_	*f*_*grad*_	*ORI*
Arc Set	(dVdD)Fit		*b*	*R* ^*2*^				
US	-3.85 (-3.88, -3.81)		124.70 (124.00, 125.50)	0.94	-2.10	0.51	0.55	0.28
MS	-3.81 (-3.84, -3.77)		116.1 0 (115.30, 116.80)	0.94	-2.10			
UD	-2.78 (-2.78, -2.77)		106.30 (106.10, 106.40)	0.99	-2.80	0.98	0.48	0.47
MD	-2.71 (-2.71, -2.70)		93.85 (93.67, 94.03)	0.99	-2.60			

#### Patient cohort

**Fig 5:**
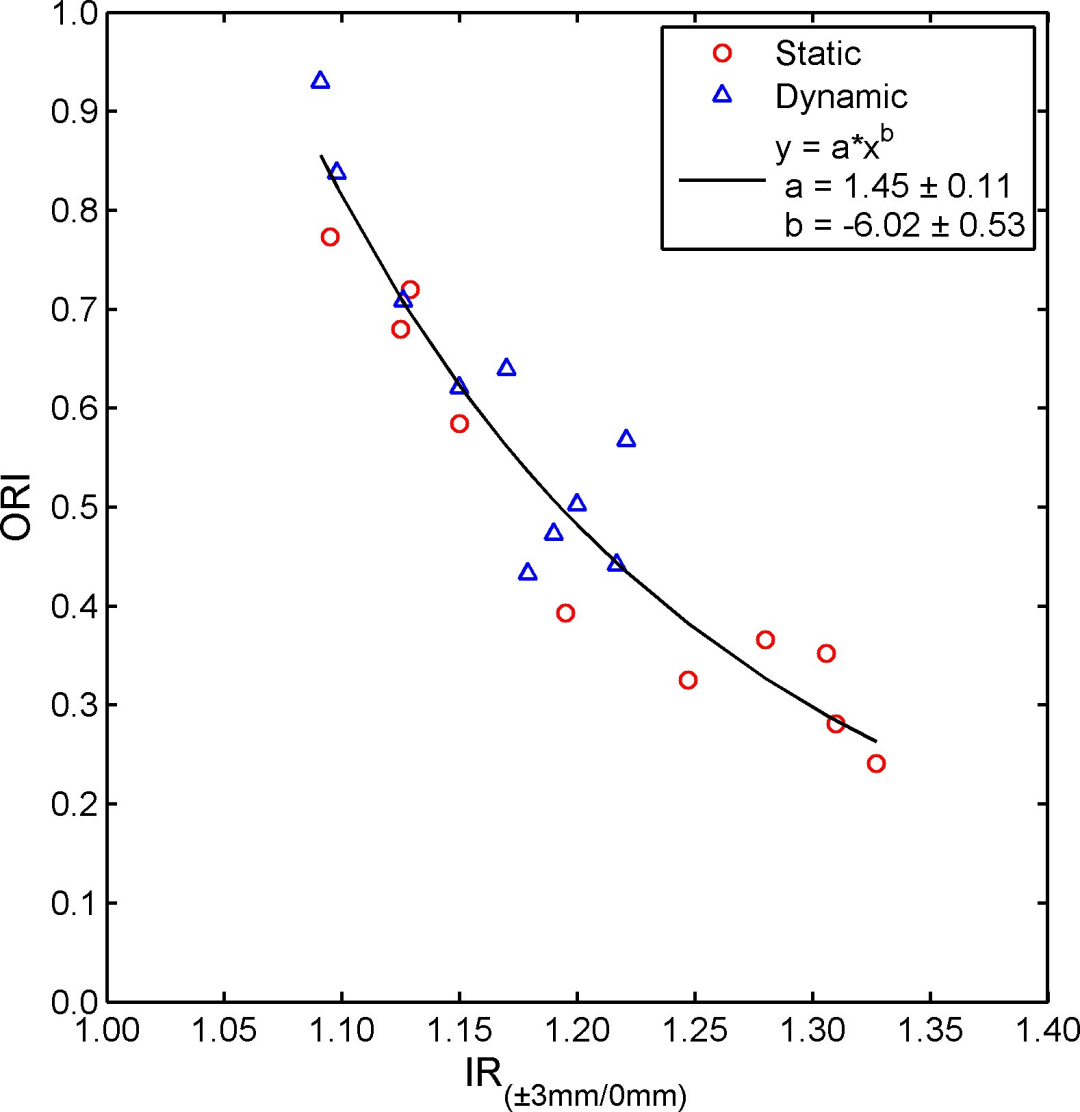
Scatter plot illustrating the correlation between the *IR*_*(±3mm/0mm)*_ and *ORI* results calculated withn the PTV_upperOverlap_ and PTV_lowerOverlap_ across all patients using the static and dynamic irradiation techniques (Spearman’s r = -0.941, *p*-value < 0.001). Results of the power fit performed on the dataset are also presented.

Table 4 summarizes the comparison of the *IR*_*(±3mm/0mm)*_ and *ORI* results between the static and dynamic treatment plans that were calculated within the considered overlaps. The two irradiation techniques resulted in statistically significant differences for both *IR*_*(±3mm/0mm)*_ and *ORI* results, with the dynamic plan yielding higher *ORI* and lower *IR*_*(±3mm/0mm)*_ values compared to static plan. The corresponding absolute differences in the calculated median values between the two irradiation techniques were equal to 0.22 and -0.05, respectively. These findings align with the Spearman correlation analysis performed, indicating a very strong inverse correlation (*r* < - 0.9, *p* < 0.001) between the calculated *ORI* and *IR*_*(±3mm/0mm)*_ values. This trend can be observed in Fig 5, where it is evident that a 5% increase in the *IR*_*(±3mm/0mm)*_ corresponds to a decrease in *ORI* of approximately 0.1. The results of fitting equation *a*×*x*^*b*^ to the *IR*_*(±3mm/0mm)*_ and *ORI* data in Fig 5 demonstrated an excellent performance with an *R*-squared value of 0.91. The fitting parameters were *a* = 1.45±0.11 and *b* = −6.02±0.53. Based on these fitting results, a tolerance limit of 0.8 and an action limit of 0.4 could be recommended for the *ORI*.

**Table 4:** A comparison of the *IR*_*(±3mm/0mm)*_ and *ORI* results between the static and dynamic irradiation techniques calculated within the PTV_upperOverlap_ and PTV_lowerOverlap_ across all patients. Statistical significance (*p* ≤ 0.05) was tested using a paired sample Wilcoxon signed rank test.

	*IR*_*(±3mm/0mm)*_		*p-*value		*ORI*		*p-*value
	Static	Dynamic		Static		Dynamic	
Median	1.221	1.174	0.031	0.379		0.594	0.004
Minimum	1.095	1.091		0.241		0.433	
Maximum	1.327	1.221		0.773		0.930	

### Experimental dose verification

**Fig 6:**
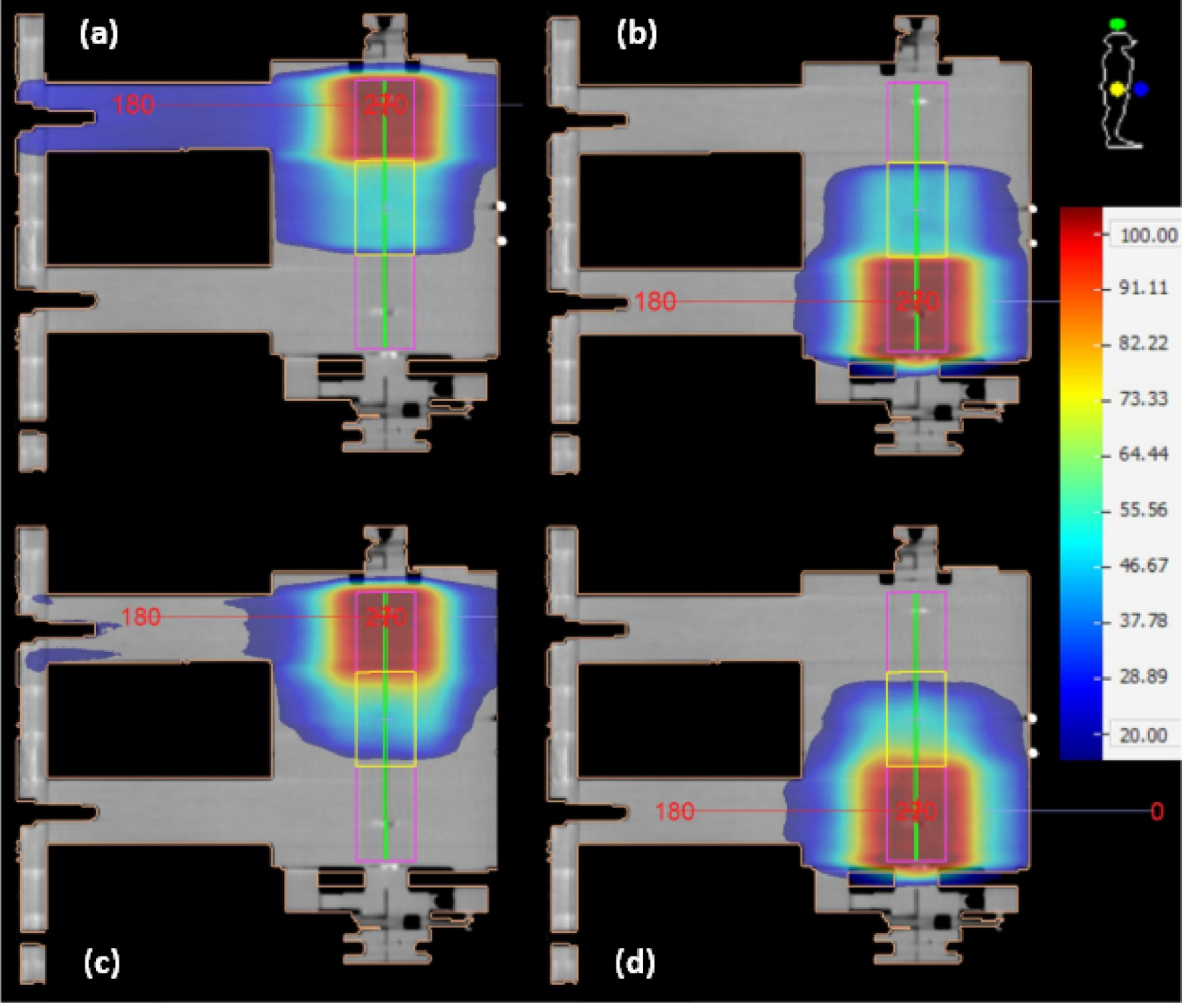
Print screen image from Monaco TPS depicting the central sagittal slice of the PMMA phantom along with a colormap representation of the % dose distribution relative to the prescribed dose within the PTV_experimentalOverlap_ (yellow) associated with the (a) US, (b) MS, (c) UD and (d) MD arc sets.

The phantom-based static and dynamic plans presented equivalent dose gradient distributions and *OR*I values with the corresponding clinical plans within the PTV_experimentalOverlap_, with the dynamic plan demonstrating a significantly smoother dose transition by the adjacent arc sets (Fig 6) compared to static plan, resulting in an *ORI* value of 0.51 over 0.26. Although the phantom treatment plans were generated for a geometry that is much simpler than a real clinical case, their equivalence with the corresponding clinical plans in terms of coverage, homogeneity and *ORI* results rendered them appropriate alternatives that could be used for the experimental verification of the conventional and staggered irradiation techniques.

**Fig 7:**
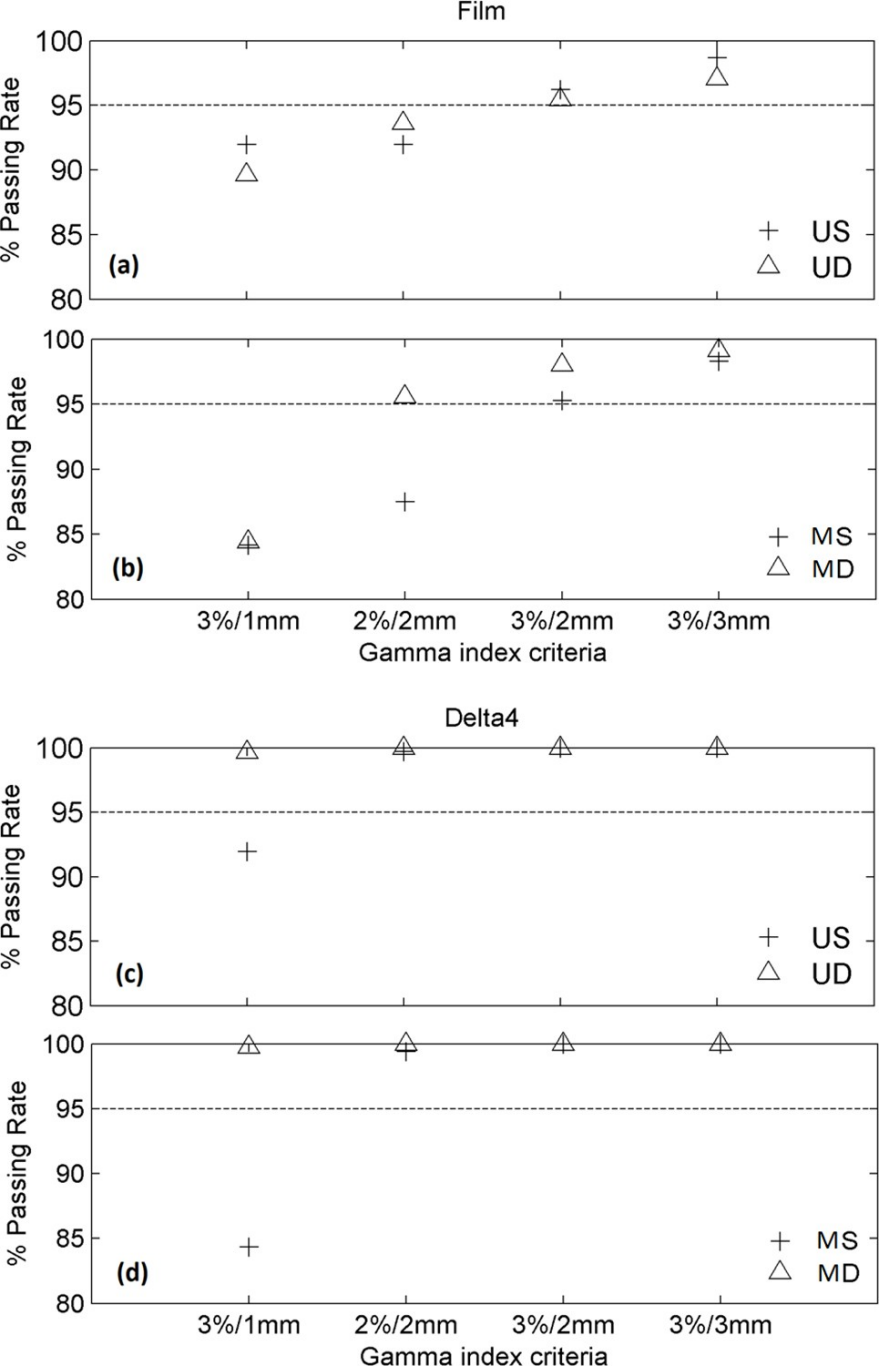
Gamma values recorded in the comparison of TPS calculations with EBT3 films (up) and Delta^4^ phantom (down) for the (a,c) US/UD and (b,d) MS/MD arc sets within the PTV_experimentalOverlap_.

Fig 7A and 7B present the comparison of TPS calculations against EBT3 film measurements within the PTV_experimentalOverlap_ in terms of the GI test. Results in Fig 7A demonstrate that US and UD arc sets were comparable for all the considered gamma criteria, while an excellent agreement was observed between the TPS and experimental results for the clinical criteria 3%/2mm and 3%/3mm (>95% of points met the passing criteria for both irradiation techniques). The corresponding GI comparison for the MS and MD arc sets in Fig 7B showed that although both irradiation techniques resulted in an excellent agreement between the calculations and measurements for the clinical criteria 3%/2mm and 3%/3mm with a passing rate >95%, the agreement associated with the MD arc set was better than that achieved by the MS for all gamma criteria applied. The maximum difference between the two irradiation techniques was observed for the 2%/2mm criterion, where the static plan yielded 8% more GI failing points compared to dynamic plan. The corresponding GI comparison between TPS calculations and Delta^4^ measurements presented in Fig 7C and 7D resulted in an excellent agreement for each arc set and for all the considered gamma criteria with GI passing rates higher than 99%, apart from the passing criterion 3%/1mm. For the latter, the static plan demonstrated a worse degree of agreement by 7.7 and 15.4% for the upper and mid arc sets, respectively, compared to dynamic plan.

**Fig 8:**
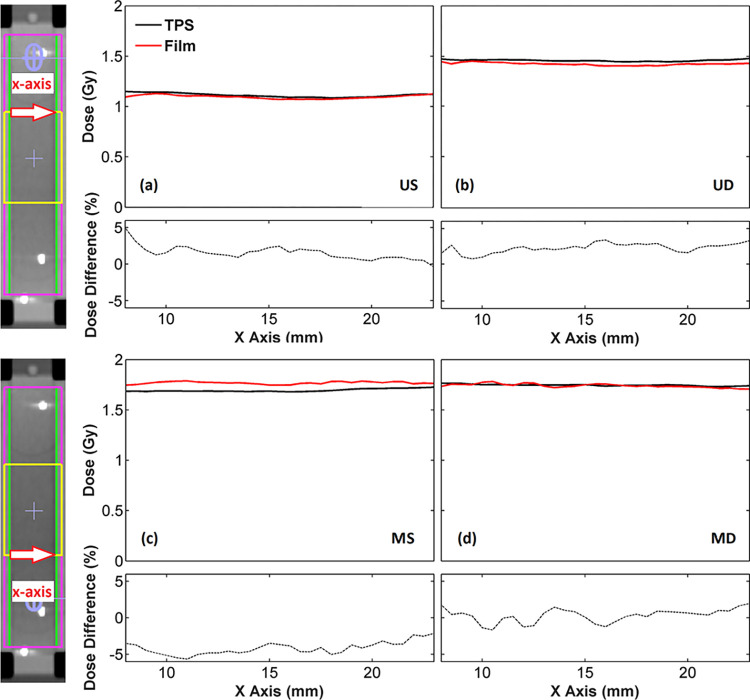
TPS and EBT3 films dose data along the x-axis corresponding to the upper (up) and lower (down) boundaries of the PTV_experimentalOverlap_ (yellow) obtained for the (a) US, (b) UD, (c) MS and (d) MD arc sets. The lines along which dose profile data were retrieved are depicted at the coronal CT slices of PMMA phantom incorporating the film. Corresponding % relative local dose differences, (%TPSexperiment−1), are also presented beneath each profile plot.

Comparisons of the TPS calculations against EBT3 film and Delta^4^ measurements in terms of 1D dose profiles, as well as percentage dosimetric differences (%TPSexperiment−1) along the boundaries of the PTV_experimentalOverlap_ were in accordance with the findings of GI test. In Fig 8, EBT3 measured and corresponding calculated TPS dose profiles along the superior and inferior boundary of the PTV_experimentalOverlap_ are plotted together with the corresponding dosimetric differences. A good agreement can be seen in Fig 8A and 8B for the dose profile data of the US and UD arc sets along the superior boundary of the PTV_experimentalOverlap_, with median values of the percentage differences equal to 1.34 and 2.56%, respectively. Corresponding comparisons between TPS and film results in Fig 8C and 8D along the inferior boundary of the PTV_experimentalOverlap_ relative to the MS and MD arc sets, respectively, showed an excellent agreement for the dose profiles of the dynamic plan with a median value of the percentage differences equal to 0.65%. The static plan, however, demonstrated an increased TPS dose underestimation at all profile points, yielding a median value of the percentage differences equal to -4.05%.

**Fig 9:**
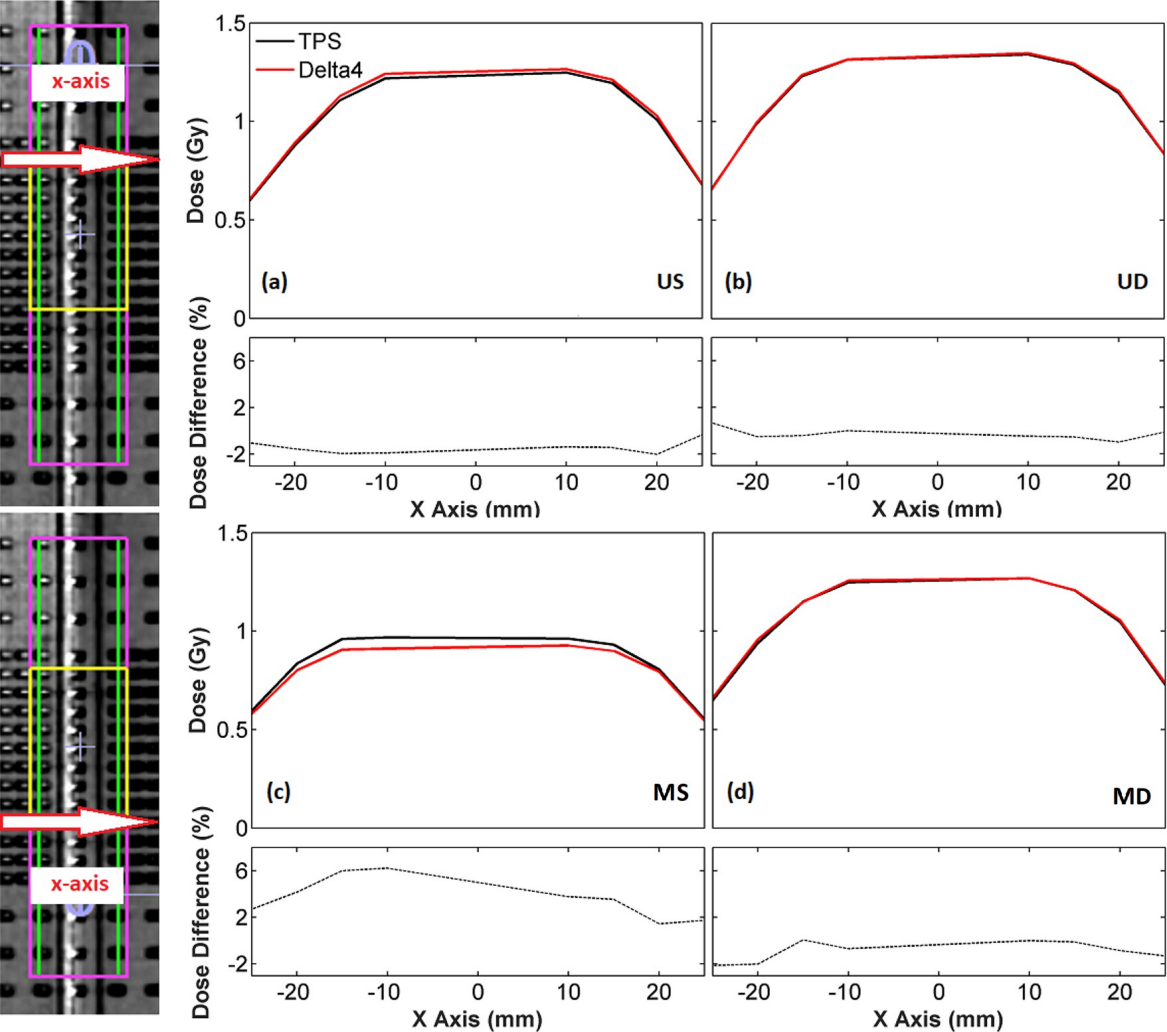
TPS and Delta^4^ dose data along the x-axis corresponding to the upper (up) and lower (down) boundaries of the PTV_experimentalOverlap_ (yellow) ±1.25 cm on either side of the boundaries for the (a) US, (b) UD, (c) MS and (d) MD arc sets. The lines along which dose profile data were retrieved are depicted at the coronal CT slices of Delta^4^ phantom incorporating the coronal detector plane. Corresponding % relative local dose differences, (%TPSexperiment−1), are also presented beneath each profile plot.

Fig 9 presents the same data as Fig 8 for the comparison between TPS and Delta^4^ results, which were plotted along the boundaries of the PTV_experimentalOverlap_ ±1.25 cm on either side of each boundary along x-axis. A good agreement can be seen in Fig 9A between the TPS and Delta^4^ dose profile data associated with the US arc set along the superior boundary of the PTV_experimentalOverlap_, which resulted in a median value of the percentage differences equal to -1.48%. Corresponding results for the UD arc set in Fig 9B demonstrated an excellent agreement between the TPS and Delta^4^ results with a median value of the percentage differences equal to -0.45%. TPS and Delta^4^ dose profiles along the inferior boundary of the PTV_experimentalOverlap_ associated with the dynamic plan in Fig 9D were found in an excellent agreement, with a median value of the percentage differences equal to -0.77%, yet a significant dose overestimation by the TPS was observed for the static plan in Fig 9C, resulting in a median value of the percentage differences of 3.66%.

## Discussion

A method was developed in this work to assess robustness within the challenging overlapping areas created in VMAT craniospinal axis irradiation techniques [2, 4, 5, 7, 9, 36], where the dose of the adjacent arc sets with different isocenters is superimposed. This method is based upon the DVHs of the adjacent arc sets, which were utilized to define the Overlap Robustness Index, a metric describing the dose gradient distribution within the overlapping regions through two coefficients, the linear and the gradient. The linear coefficient quantifies the dose gradient linearity along the overlap length that was generated by the optimizer for the adjacent arc sets, serving to identify potential steep dose gradient regions that would compromise the accuracy of dose delivery. The gradient coefficient estimates the average dose gradient within the overlap that is associated with the total dose distributed, as well as the length of the overlap. The method was applied to two VMAT techniques generated for five patients treated with CSI. The conventional overlap technique, where the fixed adjacent field setting applied does not guarantee that the optimization process will end up with a solution robust to mechanical and set-up inaccuracies [4], as well as the staggered overlap technique, a more sophisticated treatment strategy with staggering adjacent field setting that guides optimizer to provide an improved robustness against junction errors [7].

Results of this work relative to robustness showed that although the two treatment techniques were found equivalent in terms of coverage and dose homogeneity within the overlapping regions considered, the resultant *ORI* values presented significant discrepancies between the two irradiation techniques. For the individual case, the staggered overlap technique presented a 0.2 increase in the calculated *ORI* value compared to the conventional approach. This difference is associated with the notable difference of 0.4 observed in the linear coefficient between the two irradiation techniques, while the gradient coefficient remained comparable for both treatment plans. Specifically, the linear fit was deemed suitable for the staggered technique which presented a ramp-like DVH for each adjacent arc set, yet it produced erroneous results for the corresponding sigmoid-like DVHs of the conventional technique where low and high dose gradients were observed. These findings suggest that DVH linearity of the adjacent arc sets may be the most dominant parameter affecting robustness when shorter overlaps, such as the ones used in the cranial-spinal region, are involved. The analysis conducted for the patient cohort with the simulation of the ±3 mm shift error showed that the introduced shift yielded significant dose inhomogeneity differences between the two techniques within the overlapping regions, as expected [7]. The statistical analysis performed for the *ORI* results aligned with these findings, demonstrating significant discrepancies between the two treatment plans. The capability of *ORI* in detecting dosimetric uncertainties pertain to setup errors within the overlapping regions that could significantly affect quality of treatment [2, 4, 5, 7, 9] was further evaluated through the correlation between the shift induced inhomogeneity changes and *ORI* results. The strong correlation observed validated the sensitivity of *ORI*, while results of this analysis indicated that an increase in dose inhomogeneity within the overlap by 5% would translate to a decrease in *ORI* by approximately 0.1. Based on the findings of this study, tolerance and action limits of 0.8 and 0.4, respectively, could be suggested for the *ORI*. However, due to the small patient sample size used in this work, these values are acknowledged as indicative and cannot currently serve as consensus recommendations.

Wang et al. [7] evaluated robustness in conventional overlap and staggered overlap VMAT techniques by investigating the effect of positional error simulation on homogeneity-based metrics within the PTV. Results of this work for the ±3 mm shift applied between isocenters towards the cranial-caudal direction agree with the corresponding findings of Wang et al., indicating the staggered overlap technique more robust compared to the conventional one. Furthermore, they stated that longer overlaps are advantageous in decreasing dose gradients, a concept mathematically represented in this study through the incorporation of gradient coefficient in the definition of *ORI*. Seppälä et al. [2] investigated the effect of setup inaccuracies on dose distributions of 3D-CRT and dynamic split field IMRT (sfIMRT) technique, where the fields are set to overlap each other at least by 4 cm, by applying a longitudinal ±3 mm shift between the isocenters. In accordance with *ORI* and positional error simulation results of this work, they concluded that the shallower dose gradients provided by the sfIMRT technique compared to 3D-CRT yielded considerably slighter shift induced dose inhomogeneities with resultant D2%/D98% ratios of 1.25 and 2.17, respectively. Myers et al. [4] evaluated robustness for the conventional overlap and gradient-optimization VMAT techniques through the dosimetric differences between the 1D profiles of the shifted and non-shifted plans associated with the adjacent arc sets through the junction area. Although the gradient-optimization technique presented staircase instead of linear ramp-like dose profiles with increasing length of the junction area, they stated similar to the *ORI* results of this work, that shallow-sloped profiles would lead to more robust treatment delivery. Strojnik et al. [9] used the 1D profiles of the adjacent arc sets to define the idealized field junction claiming that the presence of linear ramp-like dose profiles along the transitional regions would minimize dose inhomogeneity when setup errors occur, in accordance with *ORI*-related findings of this study. It should be noticed that, in the aforementioned studies [4, 9] dose profiles of the adjacent arc sets were utilized for the evaluation of robustness, whilst the method developed in this work was based on the DVHs of the adjacent arc sets. DVHs offer the advantage of representing the 3D dose distribution within the volume of the overlap, in contrast to the 1D dose profiles taken along the PTV through the junction area, which may not be representative of the 3D dose data.

To the best of our knowledge, *ORI* poses the first single dedicated parameter to quantify robustness within the overlapping regions defined in VMAT irradiation of craniospinal axis, which could be also utilized for other treatment sites irradiated with VMAT techniques that involve overlapping areas. The substantial discrepancies observed in *ORI* values when comparing irradiation techniques of varying robustness, which were further reinforced by the strong correlation between the *ORI* and corresponding simulated positional error analysis results render *ORI* a metric appropriate for the evaluation of robustness. The main advantage of the method proposed in this work is that DVHs used for the calculation of *ORI* can be readily exported from a TPS and handled by the end-users. Since commercial TPSs do not currently include tools for calculating indices such as *ORI*, an in-house routine was developed in this work for its calculation using MATLAB. Given the simple formalism of *ORI*, as well as the ease with which DVHs can be exported from a TPS as text files, *ORI* could be also calculated by developing simple programs in widely accessible third party software packages. Developing such a program by a non-expert user is anticipated to take less than one hour, while the calculation of *ORI* thereafter would take no longer than five minutes. In our assessmnet, the incorporation of tools in TPSs for integrating *ORI* into every day clinical routine would be feasible, in order to facilitate the handling of plan robustness during the treatment planning process of CSI, as well as the decision-making process when plan quality is evaluated [10]. A limitation of *ORI* is that it forms an index not normalized to 1, as the gradient coefficient *f*_*grad*_ can exceed 1 for overlap lengths greated than 9 cm. While this could lead to misinterpretation of the *ORI* results, it was observed in this study that longer overlaps are associated with lower linear coefficient (*f*_*lin*_) values, making *ORI* values above 1 scarce. In such extreme cases, however, the optimization processs would likely have resulted in an ideal solution in terms of robustness, with *f*_*lin*_ values approaching 1, and thus *ORI* values beyond 1 can be considered to be within a safe range. An additional limitation of this study with regards to robustness is that a limited number of patients was used for the analysis of the results due to the scarcity of CSI cases in clinical practice. It should be noticed however, that small sample sizes ranging from 1 to 6 patients are common in studies related to CSI [2, 4, 5, 7, 9, 18, 36]. Additional research with a large cohort of patients is necessary to comprehensively classify *ORI* in order to establish clinically acceptable thresholds for the robust analysis, as well action limits when treatment plans tend to become vulnerable to setup errors.

EBT3 radiochromic films, as well as Delta^4^ phantom were utilized in this work in order to evaluate their applicability for dose verification within the challenging overlapping regions defined in VMAT techniques of CSI, where complexity is likely to be high. Dose was verified separately for each of the adjacent arc sets contributing to the definition of the overlapping region. The validity of the experimental methods was confirmed by conducting a comparison between the conventional and staggered overlap irradiation techniques, which were found to demonstrate considerable differences in dose transition through the overlapping regions by the adjacent arc sets. Results of this work in terms of gamma index analysis performed between the TPS calculations and corresponding EBT3 film and Delta^4^ measurements revealed that both irradiation techniques can be considered clinically acceptable, yielding gamma index passing rates higher than 95% for the clinical criteria 3%/2mm and 3%/3mm [35] for each of the adjacent arc sets. GI results of the comparisons between the TPS calculations and film measurements indicated that the staggered overlap technique exhibited higher dosimetric accuracy compared to conventional technique for the majority of passing criteria considered. Corresponding comparisons between the TPS and Delta^4^ results demonstrated that the two irradiation techniques were equivalent for the passing criteria 2%/2mm, 3%/2mm and 3%/3mm, resulting in passing rates higher than 99%. The staggered overlap technique, however, significantly outperformed the conventional technique when the stringent criterion 3%/1mm was considered. Percentage dose differences between the TPS calculations and film measurements along the boundaries of the overlapping regions resulted in median values up to 2.6% and -4.1% for the staggered and conventional overlap technique, respectively. Considering that the uncertainty ascribed to film results was lower than 1.5% for dose values higher than 1 Gy, the staggered overlap technique was found to provide a significantly improved dosimetric accuracy along the boundaries of the overlap compared to the conventional technique. These results are in accordance with the corresponding percentage dosimetric differences observed between the TPS and Delta^4^ results, which demonstrated median values up to 0.8% and 3.7% for the staggered and conventional overlap technique, respectively.

In a manner akin to robustness, results of this study indicated that the staggered overlap technique surpassed the conventional approach in terms of dose delivery accuracy, although the two treatment plans demonstrated equivalent dose distributions in terms of coverage and homogeneity within the overlapping region. This could be attributed to the fact that, unlike the conventional approach, the overlapping of the jaws in the staggered overlap technique is intentionally performed in sections, serving to compensate the undesired superposition of the dose emerging by the beam edges that is associated with increased uncertainties of the beam model [3]. Moreover, a visual inspection of the VMAT segments created within the overlapping region for the adjacent arc sets by the inverse treatment planning process showed that the staggered overlap technique may exhibit a lower leaf sequence variability compared to conventional technique, which could be related to a lower degree of complexity [12, 13] that would provide an increased treatment delivery accuracy [10]. Currently, plan complexity in clinical practice is mainly described by the total number of MUs and modulation degree [11]. These metrics, however, appeared unsuitable for predicting complexity within the overlapping region of CSI, since the two treatment techniques yielded almost identical MUs and modulation degrees. Tools appropriate for evaluating the complexity within the challenging overlapping regions of CSI would be highly desirable [10] in order to address the complexity during the treatment planning process towards improving the overall quality of treatment.

Findings of this work imply that the experimental method developed for dose verification in CSI with VMAT techniques using EBT3 films demonstrated higher sensitivity in detecting systematic dosimetric uncertainties compared to Delta^4^ phantom, probably due to the finer spatial resolution of EBT3 films (1×1 mm^2^) with respect to Delta^4^ (5 mm at the central 6 × 6 cm^2^ area). The PMMA phantom in conjunction with the EBT3 films was found suitable to benchmark TPS calculations within the overlapping regions of CSI with VMAT, since it was shown capable of detecting differences between the two treatment techniques when performing GI analysis, as well as comparisons of dose profiles along the boundaries of the overlap, as expected. These results agree with the findings presented by Seppälä et al. [2], who demonstrated that the TPS calculated and film dosimetry results recorded the largest differences in the overlapping area of the phantom where the sharpest dose gradients by the adjacent arc sets were observed. The laborious implementation of EBT3 film dosimetry, however, may render the proposed film-based experimental procedure inappropriate for patient-specific QA. On the other hand, it could serve as a tool to establish uniform commissioning procedures for TPS dose calculations within the overlapping regions of CSI with VMAT, as well as to effectively manage complexity of the relevant treatment plans. Delta^4^ was evidenced suitable for identifying dosimetric uncertainties along the boundaries of the overlapping regions, yet it exhibited limited sensitivity in GI analysis when clinically relevant passing criteria were applied within the overlapping region, indicating that potential differences of clinical interest between the treatment plans and deliveries might not be detected. The performance of Delta^4^ demonstrated high sensitivity only when the stringent criterion of 3%/1mm was applied, yet the clinical interpretation of such results still relies on intuitive judgment [35]. These outcomes differ from findings of Lee et al. [18], who showed that MapCHECK and ArcCHECK diode arrays (Sun Nuclear Corp., Melbourne, FL) demonstrate sensitivity in GI analysis within the overlapping regions of CSI when clinically relevant passing criteria are used. It should be noticed however, that the proposed experimental methods were validated utilizing a different IMRT technique that involved overlapping regions of 13–15 cm. Studenski et al. [16] stated similar to this work, that the relatively coarse resolution of 7 mm associated with the MapCHECK diodes may raise concerns regarding its capability to detect dosimetric uncertainties within the overlapping regions where high dose gradients are present.

## Conclusions

A method was developed in this study in order to define a metric suitable for the objective quantification of robustness within the overlapping regions outlined in CSI with VMAT techniques, which have been found to exhibit steep dose gradients. The implementation of this method offered a better understanding of the treatment plan characteristics associated with robustness, revealing that linear-ramp like DVHs for the adjacent arc sets involved in the definition of the overlapping regions combined with low dose gradients, would yield treatment deliveries less sensitive to setup errors. The proposed metric was validated through the comparison between the staggered and conventional overlap techniques that demonstrate differences in robustness, as well as via a correlation analysis against clinically relevant simulated positional errors, and was shown capable of detecting dosimetric uncertainties associated with geometric accuracy. This tool can be readily adopted in clinical settings to facilitate the management of robustness in CSI during the treatment planning process, still further work is required in order to enable scoring and reporting of the robustness metric presented, ensuring uniformity of practice across various institutions. The aforementioned irradiation techniques also served as a benchmark to assess the potential of two experimental methods developed in this work based on EBT3 film dosimetry, as well as on Delta^4^ phantom that is commonly used in clinical practice, for TPS dose verification within the overlapping regions of CSI with VMAT. Findings of this study suggest that film dosimetry presented an increased sensitivity in identifying underlying uncertainties pertain to beam model and treatment plan complexity within the overlapping regions that affect treatment delivery accuracy, whereas Delta^4^ phantom demonstrated a limited sensitivity when assessing the clinically relevant results of this work. The methods proposed in this work for the evaluation of robustness, as well as for dose verification hold promising potential for the administration of robustness and complexity during the treatment planning process towards enhancing the overall quality of treatment in CSI applications.

## Supporting information

S1 Data (DOCX)
