# Supplementary material for: Dose delivery uncertainties assessment in the field junction region of craniospinal irradiation with Volumetric Modulated Arc Therapy using a robustness index and experimental dose verification
Source: PLoS One. 2024 Nov 7;19(11):e0313260. doi: 10.1371/journal.pone.0313260 (PMC11542795; doi:10.1371/journal.pone.0313260)
Supplement: S1 Data — (DOCX) [file pone.0313260.s001.docx]

**S1 Table**

|  | US | | | |  | MS | | | |
| --- | --- | --- | --- | --- | --- | --- | --- | --- | --- |
|  | Percentage Volume (%) | | Histogram Data | |  | Percentage Volume (%) | | Histogram Data | |
| Dose (Gy) | DVH data | Linear Fit Data | dV/dD | Relative Frequency | Dose (Gy) | DVH data | Linear Fit Data | dV/dD | Relative Frequency |
| 0.00 | 100.00 | 124.74 | -17.20 | 0.00 | 0.00 | 100.00 | 116.09 | -14.20 | 0.00 |
| 2.41 | 100.00 | 115.49 | -16.80 | 0.00 | 1.89 | 100.00 | 108.91 | -13.80 | 0.07 |
| 2.42 | 100.00 | 115.45 | -16.40 | 0.03 | 1.90 | 100.00 | 108.88 | -13.40 | 0.14 |
| 2.49 | 100.00 | 115.18 | -16.00 | 0.00 | 1.98 | 100.00 | 108.57 | -13.00 | 0.07 |
| 2.51 | 99.99 | 115.10 | -15.60 | 0.00 | 1.99 | 99.99 | 108.53 | -12.60 | 0.20 |
| 2.53 | 99.99 | 115.03 | -15.20 | 0.07 | 2.00 | 99.99 | 108.50 | -12.20 | 0.38 |
| 2.54 | 99.99 | 114.99 | -14.80 | 0.03 | 2.04 | 99.98 | 108.34 | -11.80 | 0.51 |
| 2.56 | 99.99 | 114.91 | -14.40 | 0.10 | 2.05 | 99.98 | 108.31 | -11.40 | 0.68 |
| 2.58 | 99.99 | 114.83 | -14.00 | 0.21 | 2.06 | 99.98 | 108.27 | -11.00 | 1.02 |
| 2.60 | 99.98 | 114.76 | -13.60 | 0.27 | 2.07 | 99.98 | 108.23 | -10.60 | 0.99 |
| 2.64 | 99.97 | 114.60 | -13.20 | 0.17 | 2.08 | 99.97 | 108.19 | -10.20 | 0.82 |
| 2.65 | 99.96 | 114.56 | -12.80 | 0.31 | 2.10 | 99.97 | 108.12 | -9.80 | 1.40 |
| 2.66 | 99.96 | 114.53 | -12.40 | 0.58 | 2.11 | 99.96 | 108.08 | -9.40 | 1.19 |
| 2.68 | 99.95 | 114.45 | -12.00 | 0.51 | 2.12 | 99.96 | 108.04 | -9.00 | 1.71 |
| 2.69 | 99.94 | 114.41 | -11.60 | 0.48 | 2.16 | 99.94 | 107.89 | -8.60 | 1.30 |
| 2.70 | 99.94 | 114.37 | -11.20 | 0.72 | 2.17 | 99.93 | 107.85 | -8.20 | 1.33 |
| 2.71 | 99.94 | 114.33 | -10.80 | 0.99 | 2.18 | 99.92 | 107.81 | -7.80 | 1.13 |
| 2.72 | 99.92 | 114.29 | -10.40 | 1.03 | 2.20 | 99.90 | 107.73 | -7.40 | 1.06 |
| 2.73 | 99.91 | 114.26 | -10.00 | 1.20 | 2.22 | 99.87 | 107.66 | -7.00 | 0.85 |
| 2.75 | 99.89 | 114.18 | -9.60 | 0.92 | 2.23 | 99.86 | 107.62 | -6.60 | 0.89 |
| 2.76 | 99.88 | 114.14 | -9.20 | 1.16 | 2.25 | 99.85 | 107.54 | -6.20 | 0.79 |
| 2.77 | 99.86 | 114.10 | -8.80 | 0.96 | 2.28 | 99.81 | 107.43 | -5.80 | 0.85 |
| 2.78 | 99.85 | 114.06 | -8.40 | 0.82 | 2.29 | 99.79 | 107.39 | -5.40 | 1.50 |
| 2.80 | 99.81 | 113.99 | -8.00 | 0.92 | 2.30 | 99.77 | 107.35 | -5.00 | 1.50 |
| 2.81 | 99.80 | 113.95 | -7.60 | 1.06 | 2.31 | 99.76 | 107.32 | -4.60 | 1.43 |
| 2.82 | 99.79 | 113.91 | -7.20 | 0.86 | 2.32 | 99.75 | 107.28 | -4.20 | 2.56 |
| 2.83 | 99.78 | 113.87 | -6.80 | 0.89 | 2.34 | 99.72 | 107.20 | -3.80 | 2.08 |
| 2.84 | 99.75 | 113.83 | -6.40 | 1.16 | 2.36 | 99.71 | 107.13 | -3.40 | 3.58 |
| 2.85 | 99.74 | 113.79 | -6.00 | 0.92 | 2.37 | 99.69 | 107.09 | -3.00 | 4.40 |
| 2.86 | 99.72 | 113.76 | -5.60 | 0.96 | 2.38 | 99.69 | 107.05 | -2.60 | 6.38 |
| 2.87 | 99.70 | 113.72 | -5.20 | 1.58 | 2.39 | 99.69 | 107.01 | -2.20 | 9.49 |
| 2.88 | 99.68 | 113.68 | -4.80 | 1.64 | 2.40 | 99.67 | 106.97 | -1.80 | 11.81 |
| 2.89 | 99.66 | 113.64 | -4.40 | 2.05 | 2.41 | 99.66 | 106.94 | -1.40 | 13.08 |
| 2.90 | 99.65 | 113.60 | -4.00 | 2.05 | 2.42 | 99.65 | 106.90 | -1.00 | 11.20 |
| 2.91 | 99.64 | 113.56 | -3.60 | 3.01 | 2.43 | 99.63 | 106.86 | -0.60 | 9.32 |
| 2.92 | 99.63 | 113.53 | -3.20 | 4.25 | 2.44 | 99.62 | 106.82 | -0.20 | 4.27 |
| 2.93 | 99.61 | 113.49 | -2.80 | 5.51 | 2.45 | 99.61 | 106.78 | 0.20 | 0.00 |
| 2.94 | 99.58 | 113.45 | -2.40 | 8.70 | 2.46 | 99.60 | 106.74 |  |  |
| 2.95 | 99.56 | 113.41 | -2.00 | 11.71 | 2.48 | 99.58 | 106.67 |  |  |
| 2.96 | 99.55 | 113.37 | -1.60 | 11.20 | 2.49 | 99.57 | 106.63 |  |  |
| 2.97 | 99.53 | 113.33 | -1.20 | 12.71 | 2.52 | 99.52 | 106.52 |  |  |
| 2.98 | 99.51 | 113.29 | -0.80 | 10.34 | 2.53 | 99.51 | 106.48 |  |  |
| 2.99 | 99.48 | 113.26 | -0.40 | 5.38 | 2.54 | 99.50 | 106.44 |  |  |
| 3.00 | 99.46 | 113.22 | 0.00 | 2.50 | 2.55 | 99.47 | 106.40 |  |  |
| 3.01 | 99.44 | 113.18 |  |  | 2.56 | 99.45 | 106.36 |  |  |
| 3.02 | 99.42 | 113.14 |  |  | 2.57 | 99.43 | 106.33 |  |  |
| 3.03 | 99.40 | 113.10 |  |  | 2.59 | 99.40 | 106.25 |  |  |
| 3.04 | 99.38 | 113.06 |  |  | 2.60 | 99.36 | 106.21 |  |  |
| 3.05 | 99.34 | 113.03 |  |  | 2.61 | 99.34 | 106.17 |  |  |
| 3.06 | 99.33 | 112.99 |  |  | 2.62 | 99.33 | 106.14 |  |  |
| 3.07 | 99.30 | 112.95 |  |  | 2.63 | 99.31 | 106.10 |  |  |
| 3.08 | 99.27 | 112.91 |  |  | 2.64 | 99.30 | 106.06 |  |  |
| 3.09 | 99.25 | 112.87 |  |  | 2.65 | 99.29 | 106.02 |  |  |
| 3.10 | 99.22 | 112.83 |  |  | 2.66 | 99.26 | 105.98 |  |  |
| 3.12 | 99.17 | 112.76 |  |  | 2.67 | 99.23 | 105.95 |  |  |
| 3.13 | 99.15 | 112.72 |  |  | 2.68 | 99.20 | 105.91 |  |  |
| 3.15 | 99.10 | 112.64 |  |  | 2.69 | 99.18 | 105.87 |  |  |
| 3.16 | 99.05 | 112.60 |  |  | 2.70 | 99.17 | 105.83 |  |  |
| 3.17 | 99.03 | 112.56 |  |  | 2.71 | 99.15 | 105.79 |  |  |
| 3.18 | 99.01 | 112.52 |  |  | 2.72 | 99.13 | 105.76 |  |  |
| 3.19 | 98.99 | 112.49 |  |  | 2.73 | 99.11 | 105.72 |  |  |
| 3.20 | 98.97 | 112.45 |  |  | 2.74 | 99.09 | 105.68 |  |  |
| 3.21 | 98.95 | 112.41 |  |  | 2.75 | 99.09 | 105.64 |  |  |
| 3.22 | 98.93 | 112.37 |  |  | 2.76 | 99.06 | 105.60 |  |  |
| 3.23 | 98.90 | 112.33 |  |  | 2.77 | 99.05 | 105.56 |  |  |
| 3.24 | 98.88 | 112.29 |  |  | 2.78 | 99.03 | 105.53 |  |  |
| 3.25 | 98.87 | 112.26 |  |  | 2.79 | 99.02 | 105.49 |  |  |
| 3.27 | 98.83 | 112.18 |  |  | 2.80 | 98.99 | 105.45 |  |  |
| 3.28 | 98.82 | 112.14 |  |  | 2.81 | 98.98 | 105.41 |  |  |
| 3.29 | 98.80 | 112.10 |  |  | 2.82 | 98.97 | 105.37 |  |  |
| 3.30 | 98.79 | 112.06 |  |  | 2.83 | 98.95 | 105.34 |  |  |
| 3.31 | 98.78 | 112.02 |  |  | 2.85 | 98.94 | 105.26 |  |  |
| 3.32 | 98.76 | 111.99 |  |  | 2.86 | 98.93 | 105.22 |  |  |
| 3.34 | 98.73 | 111.91 |  |  | 2.87 | 98.91 | 105.18 |  |  |
| 3.35 | 98.72 | 111.87 |  |  | 2.88 | 98.90 | 105.15 |  |  |
| 3.36 | 98.70 | 111.83 |  |  | 2.89 | 98.88 | 105.11 |  |  |
| 3.37 | 98.68 | 111.79 |  |  | 2.90 | 98.85 | 105.07 |  |  |
| 3.38 | 98.66 | 111.76 |  |  | 2.91 | 98.83 | 105.03 |  |  |
| 3.39 | 98.63 | 111.72 |  |  | 2.92 | 98.82 | 104.99 |  |  |
| 3.40 | 98.62 | 111.68 |  |  | 2.93 | 98.80 | 104.96 |  |  |
| 3.41 | 98.60 | 111.64 |  |  | 2.94 | 98.79 | 104.92 |  |  |
| 3.42 | 98.58 | 111.60 |  |  | 2.95 | 98.77 | 104.88 |  |  |
| 3.43 | 98.56 | 111.56 |  |  | 2.97 | 98.73 | 104.80 |  |  |
| 3.44 | 98.54 | 111.52 |  |  | 2.98 | 98.72 | 104.77 |  |  |
| 3.45 | 98.53 | 111.49 |  |  | 2.99 | 98.70 | 104.73 |  |  |
| 3.46 | 98.51 | 111.45 |  |  | 3.00 | 98.69 | 104.69 |  |  |
| 3.47 | 98.48 | 111.41 |  |  | 3.01 | 98.68 | 104.65 |  |  |
| 3.48 | 98.47 | 111.37 |  |  | 3.02 | 98.66 | 104.61 |  |  |
| 3.49 | 98.47 | 111.33 |  |  | 3.03 | 98.64 | 104.58 |  |  |
| 3.50 | 98.44 | 111.29 |  |  | 3.04 | 98.62 | 104.54 |  |  |
| 3.51 | 98.43 | 111.26 |  |  | 3.05 | 98.59 | 104.50 |  |  |
| 3.52 | 98.42 | 111.22 |  |  | 3.06 | 98.57 | 104.46 |  |  |
| 3.53 | 98.41 | 111.18 |  |  | 3.07 | 98.54 | 104.42 |  |  |
| 3.54 | 98.40 | 111.14 |  |  | 3.08 | 98.51 | 104.39 |  |  |
| 3.56 | 98.38 | 111.06 |  |  | 3.09 | 98.48 | 104.35 |  |  |
| 3.57 | 98.37 | 111.02 |  |  | 3.10 | 98.45 | 104.31 |  |  |
| 3.58 | 98.34 | 110.99 |  |  | 3.11 | 98.44 | 104.27 |  |  |
| 3.59 | 98.33 | 110.95 |  |  | 3.12 | 98.42 | 104.23 |  |  |
| 3.60 | 98.32 | 110.91 |  |  | 3.14 | 98.38 | 104.16 |  |  |
| 3.61 | 98.31 | 110.87 |  |  | 3.15 | 98.37 | 104.12 |  |  |
| 3.62 | 98.30 | 110.83 |  |  | 3.17 | 98.32 | 104.04 |  |  |
| 3.63 | 98.29 | 110.79 |  |  | 3.18 | 98.31 | 104.00 |  |  |
| 3.64 | 98.27 | 110.76 |  |  | 3.20 | 98.28 | 103.93 |  |  |
| 3.65 | 98.27 | 110.72 |  |  | 3.21 | 98.26 | 103.89 |  |  |
| 3.66 | 98.26 | 110.68 |  |  | 3.22 | 98.23 | 103.85 |  |  |
| 3.68 | 98.24 | 110.60 |  |  | 3.23 | 98.22 | 103.81 |  |  |
| 3.70 | 98.22 | 110.52 |  |  | 3.24 | 98.20 | 103.78 |  |  |
| 3.71 | 98.21 | 110.49 |  |  | 3.25 | 98.18 | 103.74 |  |  |
| 3.72 | 98.21 | 110.45 |  |  | 3.26 | 98.16 | 103.70 |  |  |
| 3.74 | 98.19 | 110.37 |  |  | 3.27 | 98.15 | 103.66 |  |  |
| 3.78 | 98.16 | 110.22 |  |  | 3.28 | 98.13 | 103.62 |  |  |
| 3.79 | 98.15 | 110.18 |  |  | 3.29 | 98.12 | 103.59 |  |  |
| 3.80 | 98.15 | 110.14 |  |  | 3.32 | 98.06 | 103.47 |  |  |
| 3.81 | 98.14 | 110.10 |  |  | 3.34 | 98.03 | 103.40 |  |  |
| 3.82 | 98.14 | 110.06 |  |  | 3.35 | 98.01 | 103.36 |  |  |
| 3.83 | 98.13 | 110.02 |  |  | 3.36 | 97.99 | 103.32 |  |  |
| 3.84 | 98.12 | 109.99 |  |  | 3.37 | 97.99 | 103.28 |  |  |
| 3.85 | 98.11 | 109.95 |  |  | 3.38 | 97.97 | 103.24 |  |  |
| 3.86 | 98.11 | 109.91 |  |  | 3.39 | 97.94 | 103.21 |  |  |
| 3.87 | 98.10 | 109.87 |  |  | 3.40 | 97.93 | 103.17 |  |  |
| 3.88 | 98.10 | 109.83 |  |  | 3.41 | 97.90 | 103.13 |  |  |
| 3.89 | 98.09 | 109.79 |  |  | 3.42 | 97.89 | 103.09 |  |  |
| 3.90 | 98.09 | 109.76 |  |  | 3.43 | 97.87 | 103.05 |  |  |
| 3.91 | 98.08 | 109.72 |  |  | 3.44 | 97.85 | 103.01 |  |  |
| 3.92 | 98.08 | 109.68 |  |  | 3.45 | 97.83 | 102.98 |  |  |
| 3.94 | 98.07 | 109.60 |  |  | 3.46 | 97.81 | 102.94 |  |  |
| 3.95 | 98.06 | 109.56 |  |  | 3.48 | 97.78 | 102.86 |  |  |
| 3.96 | 98.06 | 109.52 |  |  | 3.49 | 97.76 | 102.82 |  |  |
| 3.98 | 98.06 | 109.45 |  |  | 3.50 | 97.73 | 102.79 |  |  |
| 3.99 | 98.06 | 109.41 |  |  | 3.51 | 97.71 | 102.75 |  |  |
| 4.00 | 98.05 | 109.37 |  |  | 3.52 | 97.69 | 102.71 |  |  |
| 4.01 | 98.04 | 109.33 |  |  | 3.53 | 97.66 | 102.67 |  |  |
| 4.02 | 98.04 | 109.29 |  |  | 3.54 | 97.63 | 102.63 |  |  |
| 4.03 | 98.04 | 109.25 |  |  | 3.56 | 97.57 | 102.56 |  |  |
| 4.04 | 98.03 | 109.22 |  |  | 3.57 | 97.54 | 102.52 |  |  |
| 4.05 | 98.03 | 109.18 |  |  | 3.60 | 97.47 | 102.41 |  |  |
| 4.06 | 98.02 | 109.14 |  |  | 3.61 | 97.44 | 102.37 |  |  |
| 4.08 | 98.02 | 109.06 |  |  | 3.62 | 97.41 | 102.33 |  |  |
| 4.09 | 98.02 | 109.02 |  |  | 3.63 | 97.38 | 102.29 |  |  |
| 4.10 | 98.02 | 108.99 |  |  | 3.64 | 97.36 | 102.25 |  |  |
| 4.11 | 98.01 | 108.95 |  |  | 3.65 | 97.34 | 102.22 |  |  |
| 4.12 | 98.01 | 108.91 |  |  | 3.66 | 97.32 | 102.18 |  |  |
| 4.13 | 98.01 | 108.87 |  |  | 3.67 | 97.30 | 102.14 |  |  |
| 4.14 | 98.00 | 108.83 |  |  | 3.68 | 97.28 | 102.10 |  |  |
| 4.15 | 98.00 | 108.79 |  |  | 3.70 | 97.24 | 102.03 |  |  |
| 4.16 | 97.99 | 108.75 |  |  | 3.71 | 97.20 | 101.99 |  |  |
| 4.17 | 97.99 | 108.72 |  |  | 3.72 | 97.19 | 101.95 |  |  |
| 4.18 | 97.98 | 108.68 |  |  | 3.73 | 97.14 | 101.91 |  |  |
| 4.19 | 97.98 | 108.64 |  |  | 3.74 | 97.10 | 101.87 |  |  |
| 4.20 | 97.98 | 108.60 |  |  | 3.75 | 97.08 | 101.83 |  |  |
| 4.22 | 97.97 | 108.52 |  |  | 3.76 | 97.06 | 101.80 |  |  |
| 4.23 | 97.95 | 108.49 |  |  | 3.79 | 96.97 | 101.68 |  |  |
| 4.24 | 97.95 | 108.45 |  |  | 3.80 | 96.95 | 101.64 |  |  |
| 4.25 | 97.94 | 108.41 |  |  | 3.81 | 96.91 | 101.61 |  |  |
| 4.26 | 97.93 | 108.37 |  |  | 3.83 | 96.86 | 101.53 |  |  |
| 4.27 | 97.93 | 108.33 |  |  | 3.84 | 96.85 | 101.49 |  |  |
| 4.28 | 97.92 | 108.29 |  |  | 3.85 | 96.82 | 101.45 |  |  |
| 4.31 | 97.91 | 108.18 |  |  | 3.86 | 96.78 | 101.42 |  |  |
| 4.32 | 97.90 | 108.14 |  |  | 3.87 | 96.75 | 101.38 |  |  |
| 4.33 | 97.90 | 108.10 |  |  | 3.88 | 96.73 | 101.34 |  |  |
| 4.34 | 97.88 | 108.06 |  |  | 3.89 | 96.71 | 101.30 |  |  |
| 4.35 | 97.88 | 108.02 |  |  | 3.90 | 96.69 | 101.26 |  |  |
| 4.36 | 97.87 | 107.99 |  |  | 3.91 | 96.67 | 101.23 |  |  |
| 4.37 | 97.87 | 107.95 |  |  | 3.92 | 96.66 | 101.19 |  |  |
| 4.38 | 97.86 | 107.91 |  |  | 3.93 | 96.63 | 101.15 |  |  |
| 4.39 | 97.84 | 107.87 |  |  | 3.94 | 96.61 | 101.11 |  |  |
| 4.40 | 97.83 | 107.83 |  |  | 3.96 | 96.57 | 101.04 |  |  |
| 4.41 | 97.82 | 107.79 |  |  | 3.97 | 96.56 | 101.00 |  |  |
| 4.42 | 97.80 | 107.75 |  |  | 3.98 | 96.53 | 100.96 |  |  |
| 4.43 | 97.80 | 107.72 |  |  | 3.99 | 96.51 | 100.92 |  |  |
| 4.44 | 97.78 | 107.68 |  |  | 4.00 | 96.49 | 100.88 |  |  |
| 4.45 | 97.76 | 107.64 |  |  | 4.01 | 96.48 | 100.85 |  |  |
| 4.46 | 97.75 | 107.60 |  |  | 4.02 | 96.44 | 100.81 |  |  |
| 4.47 | 97.73 | 107.56 |  |  | 4.03 | 96.43 | 100.77 |  |  |
| 4.48 | 97.72 | 107.52 |  |  | 4.04 | 96.40 | 100.73 |  |  |
| 4.49 | 97.71 | 107.49 |  |  | 4.06 | 96.36 | 100.66 |  |  |
| 4.51 | 97.68 | 107.41 |  |  | 4.07 | 96.34 | 100.62 |  |  |
| 4.52 | 97.67 | 107.37 |  |  | 4.08 | 96.32 | 100.58 |  |  |
| 4.53 | 97.65 | 107.33 |  |  | 4.09 | 96.30 | 100.54 |  |  |
| 4.54 | 97.64 | 107.29 |  |  | 4.10 | 96.28 | 100.50 |  |  |
| 4.55 | 97.62 | 107.25 |  |  | 4.11 | 96.26 | 100.46 |  |  |
| 4.56 | 97.60 | 107.22 |  |  | 4.12 | 96.24 | 100.43 |  |  |
| 4.57 | 97.58 | 107.18 |  |  | 4.13 | 96.22 | 100.39 |  |  |
| 4.58 | 97.57 | 107.14 |  |  | 4.14 | 96.20 | 100.35 |  |  |
| 4.59 | 97.55 | 107.10 |  |  | 4.16 | 96.16 | 100.27 |  |  |
| 4.60 | 97.52 | 107.06 |  |  | 4.17 | 96.14 | 100.24 |  |  |
| 4.61 | 97.50 | 107.02 |  |  | 4.18 | 96.11 | 100.20 |  |  |
| 4.63 | 97.46 | 106.95 |  |  | 4.19 | 96.08 | 100.16 |  |  |
| 4.64 | 97.43 | 106.91 |  |  | 4.20 | 96.07 | 100.12 |  |  |
| 4.65 | 97.40 | 106.87 |  |  | 4.21 | 96.04 | 100.08 |  |  |
| 4.66 | 97.39 | 106.83 |  |  | 4.22 | 96.02 | 100.05 |  |  |
| 4.68 | 97.35 | 106.75 |  |  | 4.23 | 96.01 | 100.01 |  |  |
| 4.69 | 97.34 | 106.72 |  |  | 4.24 | 95.99 | 99.97 |  |  |
| 4.70 | 97.31 | 106.68 |  |  | 4.25 | 95.97 | 99.93 |  |  |
| 4.71 | 97.27 | 106.64 |  |  | 4.26 | 95.94 | 99.89 |  |  |
| 4.72 | 97.24 | 106.60 |  |  | 4.27 | 95.93 | 99.86 |  |  |
| 4.73 | 97.21 | 106.56 |  |  | 4.28 | 95.91 | 99.82 |  |  |
| 4.74 | 97.18 | 106.52 |  |  | 4.29 | 95.88 | 99.78 |  |  |
| 4.76 | 97.12 | 106.45 |  |  | 4.30 | 95.85 | 99.74 |  |  |
| 4.77 | 97.09 | 106.41 |  |  | 4.31 | 95.84 | 99.70 |  |  |
| 4.78 | 97.08 | 106.37 |  |  | 4.32 | 95.82 | 99.67 |  |  |
| 4.79 | 97.06 | 106.33 |  |  | 4.33 | 95.81 | 99.63 |  |  |
| 4.80 | 97.04 | 106.29 |  |  | 4.34 | 95.78 | 99.59 |  |  |
| 4.81 | 97.02 | 106.25 |  |  | 4.36 | 95.75 | 99.51 |  |  |
| 4.82 | 97.00 | 106.22 |  |  | 4.37 | 95.72 | 99.48 |  |  |
| 4.83 | 97.00 | 106.18 |  |  | 4.38 | 95.71 | 99.44 |  |  |
| 4.84 | 96.99 | 106.14 |  |  | 4.39 | 95.69 | 99.40 |  |  |
| 4.86 | 96.97 | 106.06 |  |  | 4.40 | 95.66 | 99.36 |  |  |
| 4.87 | 96.95 | 106.02 |  |  | 4.41 | 95.65 | 99.32 |  |  |
| 4.88 | 96.93 | 105.98 |  |  | 4.42 | 95.64 | 99.28 |  |  |
| 4.89 | 96.92 | 105.95 |  |  | 4.43 | 95.61 | 99.25 |  |  |
| 4.90 | 96.92 | 105.91 |  |  | 4.44 | 95.60 | 99.21 |  |  |
| 4.92 | 96.90 | 105.83 |  |  | 4.46 | 95.55 | 99.13 |  |  |
| 4.93 | 96.88 | 105.79 |  |  | 4.47 | 95.54 | 99.09 |  |  |
| 4.95 | 96.87 | 105.72 |  |  | 4.48 | 95.52 | 99.06 |  |  |
| 4.99 | 96.83 | 105.56 |  |  | 4.49 | 95.50 | 99.02 |  |  |
| 5.00 | 96.82 | 105.52 |  |  | 4.50 | 95.49 | 98.98 |  |  |
| 5.01 | 96.81 | 105.48 |  |  | 4.51 | 95.47 | 98.94 |  |  |
| 5.02 | 96.80 | 105.45 |  |  | 4.53 | 95.42 | 98.87 |  |  |
| 5.03 | 96.79 | 105.41 |  |  | 4.54 | 95.40 | 98.83 |  |  |
| 5.05 | 96.78 | 105.33 |  |  | 4.55 | 95.38 | 98.79 |  |  |
| 5.06 | 96.77 | 105.29 |  |  | 4.56 | 95.36 | 98.75 |  |  |
| 5.07 | 96.76 | 105.25 |  |  | 4.57 | 95.33 | 98.71 |  |  |
| 5.08 | 96.75 | 105.22 |  |  | 4.58 | 95.31 | 98.68 |  |  |
| 5.09 | 96.74 | 105.18 |  |  | 4.60 | 95.28 | 98.60 |  |  |
| 5.10 | 96.73 | 105.14 |  |  | 4.62 | 95.22 | 98.52 |  |  |
| 5.12 | 96.71 | 105.06 |  |  | 4.63 | 95.21 | 98.49 |  |  |
| 5.13 | 96.70 | 105.02 |  |  | 4.64 | 95.18 | 98.45 |  |  |
| 5.14 | 96.69 | 104.98 |  |  | 4.65 | 95.15 | 98.41 |  |  |
| 5.15 | 96.69 | 104.95 |  |  | 4.66 | 95.11 | 98.37 |  |  |
| 5.16 | 96.68 | 104.91 |  |  | 4.67 | 95.07 | 98.33 |  |  |
| 5.17 | 96.67 | 104.87 |  |  | 4.68 | 95.04 | 98.30 |  |  |
| 5.18 | 96.66 | 104.83 |  |  | 4.69 | 95.01 | 98.26 |  |  |
| 5.20 | 96.65 | 104.75 |  |  | 4.70 | 94.98 | 98.22 |  |  |
| 5.21 | 96.64 | 104.72 |  |  | 4.71 | 94.96 | 98.18 |  |  |
| 5.22 | 96.63 | 104.68 |  |  | 4.72 | 94.94 | 98.14 |  |  |
| 5.23 | 96.63 | 104.64 |  |  | 4.73 | 94.92 | 98.10 |  |  |
| 5.24 | 96.62 | 104.60 |  |  | 4.74 | 94.89 | 98.07 |  |  |
| 5.25 | 96.61 | 104.56 |  |  | 4.75 | 94.85 | 98.03 |  |  |
| 5.26 | 96.60 | 104.52 |  |  | 4.77 | 94.81 | 97.95 |  |  |
| 5.28 | 96.57 | 104.45 |  |  | 4.78 | 94.77 | 97.91 |  |  |
| 5.29 | 96.56 | 104.41 |  |  | 4.79 | 94.75 | 97.88 |  |  |
| 5.30 | 96.56 | 104.37 |  |  | 4.80 | 94.74 | 97.84 |  |  |
| 5.31 | 96.54 | 104.33 |  |  | 4.81 | 94.70 | 97.80 |  |  |
| 5.32 | 96.54 | 104.29 |  |  | 4.82 | 94.68 | 97.76 |  |  |
| 5.34 | 96.52 | 104.22 |  |  | 4.83 | 94.65 | 97.72 |  |  |
| 5.35 | 96.52 | 104.18 |  |  | 4.84 | 94.63 | 97.69 |  |  |
| 5.36 | 96.50 | 104.14 |  |  | 4.85 | 94.62 | 97.65 |  |  |
| 5.37 | 96.48 | 104.10 |  |  | 4.86 | 94.58 | 97.61 |  |  |
| 5.38 | 96.47 | 104.06 |  |  | 4.88 | 94.53 | 97.53 |  |  |
| 5.39 | 96.47 | 104.02 |  |  | 4.89 | 94.51 | 97.50 |  |  |
| 5.40 | 96.46 | 103.98 |  |  | 4.90 | 94.50 | 97.46 |  |  |
| 5.41 | 96.46 | 103.95 |  |  | 4.91 | 94.48 | 97.42 |  |  |
| 5.42 | 96.45 | 103.91 |  |  | 4.92 | 94.47 | 97.38 |  |  |
| 5.43 | 96.44 | 103.87 |  |  | 4.93 | 94.44 | 97.34 |  |  |
| 5.44 | 96.43 | 103.83 |  |  | 4.94 | 94.43 | 97.31 |  |  |
| 5.46 | 96.42 | 103.75 |  |  | 4.95 | 94.40 | 97.27 |  |  |
| 5.47 | 96.42 | 103.72 |  |  | 4.96 | 94.38 | 97.23 |  |  |
| 5.48 | 96.40 | 103.68 |  |  | 4.97 | 94.37 | 97.19 |  |  |
| 5.49 | 96.39 | 103.64 |  |  | 4.98 | 94.36 | 97.15 |  |  |
| 5.50 | 96.38 | 103.60 |  |  | 4.99 | 94.33 | 97.12 |  |  |
| 5.51 | 96.38 | 103.56 |  |  | 5.00 | 94.30 | 97.08 |  |  |
| 5.52 | 96.37 | 103.52 |  |  | 5.01 | 94.29 | 97.04 |  |  |
| 5.53 | 96.37 | 103.48 |  |  | 5.02 | 94.27 | 97.00 |  |  |
| 5.55 | 96.34 | 103.41 |  |  | 5.03 | 94.25 | 96.96 |  |  |
| 5.57 | 96.32 | 103.33 |  |  | 5.04 | 94.24 | 96.93 |  |  |
| 5.58 | 96.31 | 103.29 |  |  | 5.05 | 94.22 | 96.89 |  |  |
| 5.59 | 96.30 | 103.25 |  |  | 5.06 | 94.20 | 96.85 |  |  |
| 5.60 | 96.28 | 103.22 |  |  | 5.07 | 94.19 | 96.81 |  |  |
| 5.61 | 96.27 | 103.18 |  |  | 5.08 | 94.17 | 96.77 |  |  |
| 5.62 | 96.27 | 103.14 |  |  | 5.10 | 94.13 | 96.70 |  |  |
| 5.63 | 96.26 | 103.10 |  |  | 5.11 | 94.11 | 96.66 |  |  |
| 5.64 | 96.25 | 103.06 |  |  | 5.12 | 94.09 | 96.62 |  |  |
| 5.65 | 96.24 | 103.02 |  |  | 5.13 | 94.07 | 96.58 |  |  |
| 5.66 | 96.23 | 102.98 |  |  | 5.14 | 94.06 | 96.54 |  |  |
| 5.67 | 96.23 | 102.95 |  |  | 5.15 | 94.02 | 96.51 |  |  |
| 5.68 | 96.20 | 102.91 |  |  | 5.16 | 94.01 | 96.47 |  |  |
| 5.69 | 96.19 | 102.87 |  |  | 5.17 | 93.99 | 96.43 |  |  |
| 5.70 | 96.18 | 102.83 |  |  | 5.18 | 93.98 | 96.39 |  |  |
| 5.71 | 96.16 | 102.79 |  |  | 5.20 | 93.95 | 96.32 |  |  |
| 5.72 | 96.14 | 102.75 |  |  | 5.23 | 93.90 | 96.20 |  |  |
| 5.73 | 96.12 | 102.71 |  |  | 5.25 | 93.87 | 96.13 |  |  |
| 5.75 | 96.10 | 102.64 |  |  | 5.26 | 93.85 | 96.09 |  |  |
| 5.76 | 96.09 | 102.60 |  |  | 5.27 | 93.84 | 96.05 |  |  |
| 5.77 | 96.08 | 102.56 |  |  | 5.28 | 93.83 | 96.01 |  |  |
| 5.78 | 96.06 | 102.52 |  |  | 5.30 | 93.78 | 95.94 |  |  |
| 5.79 | 96.05 | 102.48 |  |  | 5.31 | 93.77 | 95.90 |  |  |
| 5.80 | 96.03 | 102.45 |  |  | 5.32 | 93.75 | 95.86 |  |  |
| 5.81 | 96.02 | 102.41 |  |  | 5.33 | 93.73 | 95.82 |  |  |
| 5.82 | 96.01 | 102.37 |  |  | 5.34 | 93.70 | 95.78 |  |  |
| 5.83 | 96.01 | 102.33 |  |  | 5.36 | 93.68 | 95.71 |  |  |
| 5.84 | 95.98 | 102.29 |  |  | 5.37 | 93.66 | 95.67 |  |  |
| 5.85 | 95.97 | 102.25 |  |  | 5.38 | 93.65 | 95.63 |  |  |
| 5.86 | 95.96 | 102.21 |  |  | 5.39 | 93.64 | 95.59 |  |  |
| 5.87 | 95.94 | 102.18 |  |  | 5.40 | 93.62 | 95.55 |  |  |
| 5.88 | 95.93 | 102.14 |  |  | 5.41 | 93.61 | 95.52 |  |  |
| 5.89 | 95.92 | 102.10 |  |  | 5.43 | 93.57 | 95.44 |  |  |
| 5.90 | 95.92 | 102.06 |  |  | 5.44 | 93.55 | 95.40 |  |  |
| 5.91 | 95.90 | 102.02 |  |  | 5.46 | 93.52 | 95.33 |  |  |
| 5.92 | 95.89 | 101.98 |  |  | 5.47 | 93.52 | 95.29 |  |  |
| 5.93 | 95.87 | 101.95 |  |  | 5.48 | 93.50 | 95.25 |  |  |
| 5.94 | 95.86 | 101.91 |  |  | 5.49 | 93.49 | 95.21 |  |  |
| 5.95 | 95.86 | 101.87 |  |  | 5.50 | 93.47 | 95.17 |  |  |
| 5.96 | 95.83 | 101.83 |  |  | 5.52 | 93.44 | 95.10 |  |  |
| 5.97 | 95.83 | 101.79 |  |  | 5.54 | 93.38 | 95.02 |  |  |
| 5.98 | 95.81 | 101.75 |  |  | 5.55 | 93.38 | 94.98 |  |  |
| 5.99 | 95.80 | 101.71 |  |  | 5.56 | 93.36 | 94.95 |  |  |
| 6.00 | 95.79 | 101.68 |  |  | 5.57 | 93.34 | 94.91 |  |  |
| 6.01 | 95.78 | 101.64 |  |  | 5.58 | 93.33 | 94.87 |  |  |
| 6.03 | 95.76 | 101.56 |  |  | 5.59 | 93.30 | 94.83 |  |  |
| 6.04 | 95.75 | 101.52 |  |  | 5.60 | 93.27 | 94.79 |  |  |
| 6.05 | 95.73 | 101.48 |  |  | 5.61 | 93.27 | 94.76 |  |  |
| 6.06 | 95.72 | 101.45 |  |  | 5.62 | 93.25 | 94.72 |  |  |
| 6.07 | 95.71 | 101.41 |  |  | 5.63 | 93.24 | 94.68 |  |  |
| 6.08 | 95.70 | 101.37 |  |  | 5.64 | 93.21 | 94.64 |  |  |
| 6.10 | 95.67 | 101.29 |  |  | 5.65 | 93.20 | 94.60 |  |  |
| 6.11 | 95.66 | 101.25 |  |  | 5.66 | 93.17 | 94.57 |  |  |
| 6.12 | 95.65 | 101.21 |  |  | 5.67 | 93.16 | 94.53 |  |  |
| 6.13 | 95.64 | 101.18 |  |  | 5.68 | 93.14 | 94.49 |  |  |
| 6.14 | 95.62 | 101.14 |  |  | 5.69 | 93.12 | 94.45 |  |  |
| 6.15 | 95.61 | 101.10 |  |  | 5.71 | 93.07 | 94.37 |  |  |
| 6.16 | 95.60 | 101.06 |  |  | 5.73 | 93.04 | 94.30 |  |  |
| 6.17 | 95.59 | 101.02 |  |  | 5.74 | 93.02 | 94.26 |  |  |
| 6.18 | 95.58 | 100.98 |  |  | 5.75 | 93.00 | 94.22 |  |  |
| 6.19 | 95.57 | 100.95 |  |  | 5.76 | 92.98 | 94.18 |  |  |
| 6.20 | 95.55 | 100.91 |  |  | 5.77 | 92.95 | 94.15 |  |  |
| 6.21 | 95.54 | 100.87 |  |  | 5.80 | 92.89 | 94.03 |  |  |
| 6.22 | 95.53 | 100.83 |  |  | 5.81 | 92.87 | 93.99 |  |  |
| 6.23 | 95.51 | 100.79 |  |  | 5.82 | 92.85 | 93.96 |  |  |
| 6.24 | 95.50 | 100.75 |  |  | 5.83 | 92.83 | 93.92 |  |  |
| 6.25 | 95.48 | 100.71 |  |  | 5.84 | 92.80 | 93.88 |  |  |
| 6.27 | 95.42 | 100.64 |  |  | 5.85 | 92.77 | 93.84 |  |  |
| 6.28 | 95.40 | 100.60 |  |  | 5.87 | 92.73 | 93.77 |  |  |
| 6.29 | 95.38 | 100.56 |  |  | 5.88 | 92.69 | 93.73 |  |  |
| 6.30 | 95.36 | 100.52 |  |  | 5.89 | 92.66 | 93.69 |  |  |
| 6.31 | 95.34 | 100.48 |  |  | 5.90 | 92.63 | 93.65 |  |  |
| 6.32 | 95.33 | 100.45 |  |  | 5.92 | 92.57 | 93.58 |  |  |
| 6.33 | 95.31 | 100.41 |  |  | 5.93 | 92.56 | 93.54 |  |  |
| 6.34 | 95.28 | 100.37 |  |  | 5.94 | 92.53 | 93.50 |  |  |
| 6.35 | 95.26 | 100.33 |  |  | 5.95 | 92.52 | 93.46 |  |  |
| 6.36 | 95.25 | 100.29 |  |  | 5.96 | 92.50 | 93.42 |  |  |
| 6.40 | 95.20 | 100.14 |  |  | 5.97 | 92.49 | 93.39 |  |  |
| 6.41 | 95.19 | 100.10 |  |  | 5.98 | 92.47 | 93.35 |  |  |
| 6.42 | 95.18 | 100.06 |  |  | 5.99 | 92.45 | 93.31 |  |  |
| 6.43 | 95.15 | 100.02 |  |  | 6.00 | 92.44 | 93.27 |  |  |
| 6.44 | 95.12 | 99.98 |  |  | 6.01 | 92.41 | 93.23 |  |  |
| 6.45 | 95.11 | 99.95 |  |  | 6.02 | 92.39 | 93.19 |  |  |
| 6.46 | 95.09 | 99.91 |  |  | 6.03 | 92.37 | 93.16 |  |  |
| 6.47 | 95.07 | 99.87 |  |  | 6.04 | 92.35 | 93.12 |  |  |
| 6.48 | 95.05 | 99.83 |  |  | 6.05 | 92.31 | 93.08 |  |  |
| 6.49 | 95.04 | 99.79 |  |  | 6.06 | 92.31 | 93.04 |  |  |
| 6.50 | 95.01 | 99.75 |  |  | 6.07 | 92.28 | 93.00 |  |  |
| 6.52 | 95.00 | 99.68 |  |  | 6.08 | 92.26 | 92.97 |  |  |
| 6.54 | 94.96 | 99.60 |  |  | 6.09 | 92.25 | 92.93 |  |  |
| 6.55 | 94.96 | 99.56 |  |  | 6.10 | 92.23 | 92.89 |  |  |
| 6.56 | 94.94 | 99.52 |  |  | 6.11 | 92.21 | 92.85 |  |  |
| 6.59 | 94.93 | 99.41 |  |  | 6.13 | 92.16 | 92.78 |  |  |
| 6.60 | 94.92 | 99.37 |  |  | 6.15 | 92.14 | 92.70 |  |  |
| 6.61 | 94.92 | 99.33 |  |  | 6.16 | 92.10 | 92.66 |  |  |
| 6.62 | 94.91 | 99.29 |  |  | 6.17 | 92.09 | 92.62 |  |  |
| 6.63 | 94.90 | 99.25 |  |  | 6.19 | 92.05 | 92.55 |  |  |
| 6.64 | 94.89 | 99.21 |  |  | 6.20 | 92.03 | 92.51 |  |  |
| 6.65 | 94.89 | 99.18 |  |  | 6.21 | 92.01 | 92.47 |  |  |
| 6.66 | 94.88 | 99.14 |  |  | 6.22 | 92.00 | 92.43 |  |  |
| 6.67 | 94.87 | 99.10 |  |  | 6.23 | 91.99 | 92.40 |  |  |
| 6.68 | 94.87 | 99.06 |  |  | 6.24 | 91.96 | 92.36 |  |  |
| 6.69 | 94.87 | 99.02 |  |  | 6.25 | 91.94 | 92.32 |  |  |
| 6.70 | 94.86 | 98.98 |  |  | 6.26 | 91.92 | 92.28 |  |  |
| 6.71 | 94.85 | 98.94 |  |  | 6.27 | 91.89 | 92.24 |  |  |
| 6.72 | 94.85 | 98.91 |  |  | 6.28 | 91.87 | 92.21 |  |  |
| 6.73 | 94.84 | 98.87 |  |  | 6.29 | 91.86 | 92.17 |  |  |
| 6.74 | 94.83 | 98.83 |  |  | 6.30 | 91.84 | 92.13 |  |  |
| 6.75 | 94.83 | 98.79 |  |  | 6.31 | 91.82 | 92.09 |  |  |
| 6.77 | 94.81 | 98.71 |  |  | 6.32 | 91.80 | 92.05 |  |  |
| 6.79 | 94.81 | 98.64 |  |  | 6.33 | 91.79 | 92.02 |  |  |
| 6.80 | 94.80 | 98.60 |  |  | 6.34 | 91.77 | 91.98 |  |  |
| 6.81 | 94.79 | 98.56 |  |  | 6.35 | 91.75 | 91.94 |  |  |
| 6.82 | 94.78 | 98.52 |  |  | 6.36 | 91.73 | 91.90 |  |  |
| 6.83 | 94.77 | 98.48 |  |  | 6.37 | 91.70 | 91.86 |  |  |
| 6.84 | 94.76 | 98.44 |  |  | 6.38 | 91.68 | 91.82 |  |  |
| 6.85 | 94.75 | 98.41 |  |  | 6.39 | 91.67 | 91.79 |  |  |
| 6.86 | 94.75 | 98.37 |  |  | 6.40 | 91.65 | 91.75 |  |  |
| 6.87 | 94.74 | 98.33 |  |  | 6.41 | 91.63 | 91.71 |  |  |
| 6.88 | 94.73 | 98.29 |  |  | 6.43 | 91.60 | 91.63 |  |  |
| 6.89 | 94.73 | 98.25 |  |  | 6.44 | 91.57 | 91.60 |  |  |
| 6.90 | 94.73 | 98.21 |  |  | 6.45 | 91.56 | 91.56 |  |  |
| 6.91 | 94.73 | 98.18 |  |  | 6.46 | 91.54 | 91.52 |  |  |
| 6.92 | 94.73 | 98.14 |  |  | 6.47 | 91.52 | 91.48 |  |  |
| 6.94 | 94.70 | 98.06 |  |  | 6.48 | 91.49 | 91.44 |  |  |
| 6.95 | 94.69 | 98.02 |  |  | 6.49 | 91.49 | 91.41 |  |  |
| 6.96 | 94.69 | 97.98 |  |  | 6.50 | 91.47 | 91.37 |  |  |
| 6.97 | 94.68 | 97.94 |  |  | 6.51 | 91.44 | 91.33 |  |  |
| 6.98 | 94.67 | 97.91 |  |  | 6.52 | 91.44 | 91.29 |  |  |
| 6.99 | 94.65 | 97.87 |  |  | 6.53 | 91.42 | 91.25 |  |  |
| 7.00 | 94.65 | 97.83 |  |  | 6.54 | 91.40 | 91.22 |  |  |
| 7.01 | 94.65 | 97.79 |  |  | 6.55 | 91.38 | 91.18 |  |  |
| 7.02 | 94.64 | 97.75 |  |  | 6.56 | 91.36 | 91.14 |  |  |
| 7.03 | 94.64 | 97.71 |  |  | 6.57 | 91.34 | 91.10 |  |  |
| 7.04 | 94.63 | 97.68 |  |  | 6.58 | 91.32 | 91.06 |  |  |
| 7.05 | 94.62 | 97.64 |  |  | 6.60 | 91.29 | 90.99 |  |  |
| 7.06 | 94.62 | 97.60 |  |  | 6.61 | 91.27 | 90.95 |  |  |
| 7.07 | 94.60 | 97.56 |  |  | 6.62 | 91.26 | 90.91 |  |  |
| 7.08 | 94.59 | 97.52 |  |  | 6.63 | 91.25 | 90.87 |  |  |
| 7.09 | 94.59 | 97.48 |  |  | 6.64 | 91.23 | 90.84 |  |  |
| 7.10 | 94.57 | 97.44 |  |  | 6.65 | 91.22 | 90.80 |  |  |
| 7.11 | 94.56 | 97.41 |  |  | 6.67 | 91.19 | 90.72 |  |  |
| 7.12 | 94.55 | 97.37 |  |  | 6.68 | 91.18 | 90.68 |  |  |
| 7.13 | 94.53 | 97.33 |  |  | 6.69 | 91.16 | 90.64 |  |  |
| 7.14 | 94.51 | 97.29 |  |  | 6.70 | 91.15 | 90.61 |  |  |
| 7.15 | 94.50 | 97.25 |  |  | 6.71 | 91.13 | 90.57 |  |  |
| 7.16 | 94.48 | 97.21 |  |  | 6.73 | 91.11 | 90.49 |  |  |
| 7.17 | 94.47 | 97.18 |  |  | 6.75 | 91.08 | 90.42 |  |  |
| 7.18 | 94.45 | 97.14 |  |  | 6.76 | 91.06 | 90.38 |  |  |
| 7.19 | 94.43 | 97.10 |  |  | 6.77 | 91.05 | 90.34 |  |  |
| 7.21 | 94.40 | 97.02 |  |  | 6.78 | 91.04 | 90.30 |  |  |
| 7.22 | 94.40 | 96.98 |  |  | 6.79 | 91.03 | 90.26 |  |  |
| 7.23 | 94.38 | 96.94 |  |  | 6.80 | 91.02 | 90.23 |  |  |
| 7.24 | 94.37 | 96.91 |  |  | 6.81 | 91.01 | 90.19 |  |  |
| 7.25 | 94.35 | 96.87 |  |  | 6.82 | 91.00 | 90.15 |  |  |
| 7.28 | 94.32 | 96.75 |  |  | 6.83 | 90.97 | 90.11 |  |  |
| 7.29 | 94.30 | 96.71 |  |  | 6.84 | 90.96 | 90.07 |  |  |
| 7.30 | 94.29 | 96.68 |  |  | 6.85 | 90.95 | 90.04 |  |  |
| 7.33 | 94.26 | 96.56 |  |  | 6.86 | 90.94 | 90.00 |  |  |
| 7.34 | 94.25 | 96.52 |  |  | 6.87 | 90.91 | 89.96 |  |  |
| 7.35 | 94.23 | 96.48 |  |  | 6.88 | 90.90 | 89.92 |  |  |
| 7.36 | 94.23 | 96.44 |  |  | 6.89 | 90.88 | 89.88 |  |  |
| 7.37 | 94.22 | 96.41 |  |  | 6.90 | 90.87 | 89.85 |  |  |
| 7.38 | 94.20 | 96.37 |  |  | 6.91 | 90.86 | 89.81 |  |  |
| 7.39 | 94.19 | 96.33 |  |  | 6.92 | 90.85 | 89.77 |  |  |
| 7.40 | 94.18 | 96.29 |  |  | 6.93 | 90.83 | 89.73 |  |  |
| 7.41 | 94.17 | 96.25 |  |  | 6.94 | 90.82 | 89.69 |  |  |
| 7.42 | 94.16 | 96.21 |  |  | 6.95 | 90.79 | 89.66 |  |  |
| 7.43 | 94.15 | 96.18 |  |  | 6.96 | 90.77 | 89.62 |  |  |
| 7.44 | 94.14 | 96.14 |  |  | 6.97 | 90.77 | 89.58 |  |  |
| 7.45 | 94.13 | 96.10 |  |  | 6.99 | 90.74 | 89.50 |  |  |
| 7.46 | 94.12 | 96.06 |  |  | 7.00 | 90.73 | 89.46 |  |  |
| 7.47 | 94.11 | 96.02 |  |  | 7.01 | 90.72 | 89.43 |  |  |
| 7.48 | 94.09 | 95.98 |  |  | 7.02 | 90.70 | 89.39 |  |  |
| 7.49 | 94.09 | 95.94 |  |  | 7.03 | 90.67 | 89.35 |  |  |
| 7.50 | 94.07 | 95.91 |  |  | 7.04 | 90.65 | 89.31 |  |  |
| 7.51 | 94.06 | 95.87 |  |  | 7.05 | 90.64 | 89.27 |  |  |
| 7.52 | 94.06 | 95.83 |  |  | 7.06 | 90.62 | 89.24 |  |  |
| 7.54 | 94.03 | 95.75 |  |  | 7.07 | 90.61 | 89.20 |  |  |
| 7.55 | 94.02 | 95.71 |  |  | 7.08 | 90.60 | 89.16 |  |  |
| 7.56 | 94.01 | 95.67 |  |  | 7.09 | 90.57 | 89.12 |  |  |
| 7.57 | 94.00 | 95.64 |  |  | 7.10 | 90.56 | 89.08 |  |  |
| 7.58 | 93.98 | 95.60 |  |  | 7.11 | 90.54 | 89.05 |  |  |
| 7.59 | 93.98 | 95.56 |  |  | 7.12 | 90.52 | 89.01 |  |  |
| 7.60 | 93.97 | 95.52 |  |  | 7.13 | 90.51 | 88.97 |  |  |
| 7.61 | 93.96 | 95.48 |  |  | 7.14 | 90.48 | 88.93 |  |  |
| 7.62 | 93.94 | 95.44 |  |  | 7.15 | 90.48 | 88.89 |  |  |
| 7.63 | 93.93 | 95.41 |  |  | 7.16 | 90.46 | 88.86 |  |  |
| 7.64 | 93.91 | 95.37 |  |  | 7.18 | 90.44 | 88.78 |  |  |
| 7.65 | 93.89 | 95.33 |  |  | 7.19 | 90.41 | 88.74 |  |  |
| 7.66 | 93.88 | 95.29 |  |  | 7.20 | 90.40 | 88.70 |  |  |
| 7.67 | 93.87 | 95.25 |  |  | 7.21 | 90.38 | 88.67 |  |  |
| 7.68 | 93.84 | 95.21 |  |  | 7.22 | 90.36 | 88.63 |  |  |
| 7.69 | 93.84 | 95.17 |  |  | 7.23 | 90.36 | 88.59 |  |  |
| 7.70 | 93.83 | 95.14 |  |  | 7.24 | 90.34 | 88.55 |  |  |
| 7.71 | 93.83 | 95.10 |  |  | 7.25 | 90.33 | 88.51 |  |  |
| 7.72 | 93.82 | 95.06 |  |  | 7.26 | 90.31 | 88.48 |  |  |
| 7.73 | 93.81 | 95.02 |  |  | 7.27 | 90.29 | 88.44 |  |  |
| 7.74 | 93.80 | 94.98 |  |  | 7.28 | 90.27 | 88.40 |  |  |
| 7.75 | 93.78 | 94.94 |  |  | 7.29 | 90.25 | 88.36 |  |  |
| 7.76 | 93.76 | 94.91 |  |  | 7.30 | 90.23 | 88.32 |  |  |
| 7.77 | 93.75 | 94.87 |  |  | 7.31 | 90.21 | 88.29 |  |  |
| 7.78 | 93.73 | 94.83 |  |  | 7.32 | 90.19 | 88.25 |  |  |
| 7.79 | 93.71 | 94.79 |  |  | 7.33 | 90.18 | 88.21 |  |  |
| 7.81 | 93.69 | 94.71 |  |  | 7.34 | 90.17 | 88.17 |  |  |
| 7.82 | 93.66 | 94.67 |  |  | 7.35 | 90.14 | 88.13 |  |  |
| 7.83 | 93.64 | 94.64 |  |  | 7.36 | 90.11 | 88.09 |  |  |
| 7.84 | 93.62 | 94.60 |  |  | 7.37 | 90.10 | 88.06 |  |  |
| 7.85 | 93.62 | 94.56 |  |  | 7.38 | 90.08 | 88.02 |  |  |
| 7.86 | 93.60 | 94.52 |  |  | 7.39 | 90.07 | 87.98 |  |  |
| 7.87 | 93.58 | 94.48 |  |  | 7.40 | 90.04 | 87.94 |  |  |
| 7.88 | 93.56 | 94.44 |  |  | 7.41 | 90.03 | 87.90 |  |  |
| 7.90 | 93.52 | 94.37 |  |  | 7.42 | 90.01 | 87.87 |  |  |
| 7.91 | 93.51 | 94.33 |  |  | 7.43 | 90.00 | 87.83 |  |  |
| 7.92 | 93.50 | 94.29 |  |  | 7.44 | 89.98 | 87.79 |  |  |
| 7.94 | 93.49 | 94.21 |  |  | 7.45 | 89.97 | 87.75 |  |  |
| 7.96 | 93.45 | 94.14 |  |  | 7.46 | 89.96 | 87.71 |  |  |
| 7.97 | 93.43 | 94.10 |  |  | 7.47 | 89.93 | 87.68 |  |  |
| 7.98 | 93.42 | 94.06 |  |  | 7.48 | 89.91 | 87.64 |  |  |
| 7.99 | 93.40 | 94.02 |  |  | 7.49 | 89.90 | 87.60 |  |  |
| 8.00 | 93.38 | 93.98 |  |  | 7.50 | 89.87 | 87.56 |  |  |
| 8.01 | 93.36 | 93.94 |  |  | 7.51 | 89.85 | 87.52 |  |  |
| 8.02 | 93.34 | 93.91 |  |  | 7.52 | 89.83 | 87.49 |  |  |
| 8.04 | 93.32 | 93.83 |  |  | 7.53 | 89.81 | 87.45 |  |  |
| 8.05 | 93.31 | 93.79 |  |  | 7.54 | 89.79 | 87.41 |  |  |
| 8.06 | 93.30 | 93.75 |  |  | 7.55 | 89.78 | 87.37 |  |  |
| 8.08 | 93.28 | 93.67 |  |  | 7.56 | 89.76 | 87.33 |  |  |
| 8.09 | 93.26 | 93.64 |  |  | 7.58 | 89.72 | 87.26 |  |  |
| 8.10 | 93.25 | 93.60 |  |  | 7.59 | 89.69 | 87.22 |  |  |
| 8.12 | 93.21 | 93.52 |  |  | 7.60 | 89.68 | 87.18 |  |  |
| 8.13 | 93.20 | 93.48 |  |  | 7.61 | 89.67 | 87.14 |  |  |
| 8.14 | 93.18 | 93.44 |  |  | 7.62 | 89.64 | 87.11 |  |  |
| 8.15 | 93.16 | 93.41 |  |  | 7.63 | 89.62 | 87.07 |  |  |
| 8.16 | 93.14 | 93.37 |  |  | 7.64 | 89.60 | 87.03 |  |  |
| 8.17 | 93.13 | 93.33 |  |  | 7.65 | 89.59 | 86.99 |  |  |
| 8.18 | 93.12 | 93.29 |  |  | 7.66 | 89.56 | 86.95 |  |  |
| 8.19 | 93.12 | 93.25 |  |  | 7.67 | 89.55 | 86.91 |  |  |
| 8.20 | 93.10 | 93.21 |  |  | 7.69 | 89.51 | 86.84 |  |  |
| 8.21 | 93.09 | 93.17 |  |  | 7.70 | 89.48 | 86.80 |  |  |
| 8.22 | 93.08 | 93.14 |  |  | 7.71 | 89.45 | 86.76 |  |  |
| 8.23 | 93.06 | 93.10 |  |  | 7.73 | 89.42 | 86.69 |  |  |
| 8.24 | 93.05 | 93.06 |  |  | 7.74 | 89.40 | 86.65 |  |  |
| 8.25 | 93.04 | 93.02 |  |  | 7.75 | 89.38 | 86.61 |  |  |
| 8.26 | 93.03 | 92.98 |  |  | 7.76 | 89.37 | 86.57 |  |  |
| 8.27 | 93.02 | 92.94 |  |  | 7.77 | 89.33 | 86.53 |  |  |
| 8.28 | 93.01 | 92.91 |  |  | 7.78 | 89.31 | 86.50 |  |  |
| 8.29 | 93.00 | 92.87 |  |  | 7.79 | 89.30 | 86.46 |  |  |
| 8.30 | 92.99 | 92.83 |  |  | 7.80 | 89.28 | 86.42 |  |  |
| 8.32 | 92.96 | 92.75 |  |  | 7.81 | 89.27 | 86.38 |  |  |
| 8.33 | 92.95 | 92.71 |  |  | 7.83 | 89.25 | 86.31 |  |  |
| 8.34 | 92.94 | 92.67 |  |  | 7.84 | 89.22 | 86.27 |  |  |
| 8.35 | 92.93 | 92.64 |  |  | 7.85 | 89.20 | 86.23 |  |  |
| 8.36 | 92.93 | 92.60 |  |  | 7.86 | 89.18 | 86.19 |  |  |
| 8.37 | 92.92 | 92.56 |  |  | 7.87 | 89.17 | 86.15 |  |  |
| 8.38 | 92.91 | 92.52 |  |  | 7.88 | 89.14 | 86.12 |  |  |
| 8.39 | 92.91 | 92.48 |  |  | 7.89 | 89.11 | 86.08 |  |  |
| 8.40 | 92.90 | 92.44 |  |  | 7.90 | 89.09 | 86.04 |  |  |
| 8.42 | 92.89 | 92.37 |  |  | 7.91 | 89.06 | 86.00 |  |  |
| 8.43 | 92.88 | 92.33 |  |  | 7.92 | 89.05 | 85.96 |  |  |
| 8.45 | 92.86 | 92.25 |  |  | 7.93 | 89.04 | 85.93 |  |  |
| 8.46 | 92.85 | 92.21 |  |  | 7.94 | 89.02 | 85.89 |  |  |
| 8.47 | 92.85 | 92.17 |  |  | 7.95 | 89.01 | 85.85 |  |  |
| 8.48 | 92.84 | 92.14 |  |  | 7.96 | 89.00 | 85.81 |  |  |
| 8.49 | 92.84 | 92.10 |  |  | 7.97 | 88.98 | 85.77 |  |  |
| 8.51 | 92.82 | 92.02 |  |  | 7.98 | 88.95 | 85.73 |  |  |
| 8.53 | 92.81 | 91.94 |  |  | 7.99 | 88.95 | 85.70 |  |  |
| 8.54 | 92.81 | 91.90 |  |  | 8.01 | 88.90 | 85.62 |  |  |
| 8.55 | 92.79 | 91.87 |  |  | 8.02 | 88.89 | 85.58 |  |  |
| 8.56 | 92.79 | 91.83 |  |  | 8.03 | 88.87 | 85.54 |  |  |
| 8.57 | 92.78 | 91.79 |  |  | 8.04 | 88.86 | 85.51 |  |  |
| 8.58 | 92.78 | 91.75 |  |  | 8.05 | 88.84 | 85.47 |  |  |
| 8.59 | 92.77 | 91.71 |  |  | 8.06 | 88.82 | 85.43 |  |  |
| 8.60 | 92.76 | 91.67 |  |  | 8.07 | 88.80 | 85.39 |  |  |
| 8.62 | 92.75 | 91.60 |  |  | 8.08 | 88.79 | 85.35 |  |  |
| 8.63 | 92.74 | 91.56 |  |  | 8.09 | 88.77 | 85.32 |  |  |
| 8.64 | 92.73 | 91.52 |  |  | 8.10 | 88.75 | 85.28 |  |  |
| 8.65 | 92.72 | 91.48 |  |  | 8.11 | 88.73 | 85.24 |  |  |
| 8.68 | 92.71 | 91.37 |  |  | 8.12 | 88.71 | 85.20 |  |  |
| 8.69 | 92.70 | 91.33 |  |  | 8.13 | 88.69 | 85.16 |  |  |
| 8.70 | 92.70 | 91.29 |  |  | 8.14 | 88.67 | 85.13 |  |  |
| 8.71 | 92.69 | 91.25 |  |  | 8.15 | 88.65 | 85.09 |  |  |
| 8.73 | 92.66 | 91.17 |  |  | 8.16 | 88.63 | 85.05 |  |  |
| 8.74 | 92.65 | 91.14 |  |  | 8.17 | 88.61 | 85.01 |  |  |
| 8.75 | 92.65 | 91.10 |  |  | 8.19 | 88.59 | 84.94 |  |  |
| 8.76 | 92.64 | 91.06 |  |  | 8.20 | 88.58 | 84.90 |  |  |
| 8.77 | 92.63 | 91.02 |  |  | 8.21 | 88.55 | 84.86 |  |  |
| 8.78 | 92.62 | 90.98 |  |  | 8.23 | 88.53 | 84.78 |  |  |
| 8.79 | 92.61 | 90.94 |  |  | 8.24 | 88.52 | 84.75 |  |  |
| 8.80 | 92.60 | 90.90 |  |  | 8.27 | 88.46 | 84.63 |  |  |
| 8.81 | 92.58 | 90.87 |  |  | 8.28 | 88.45 | 84.59 |  |  |
| 8.82 | 92.58 | 90.83 |  |  | 8.29 | 88.43 | 84.56 |  |  |
| 8.83 | 92.57 | 90.79 |  |  | 8.30 | 88.43 | 84.52 |  |  |
| 8.84 | 92.56 | 90.75 |  |  | 8.31 | 88.41 | 84.48 |  |  |
| 8.85 | 92.56 | 90.71 |  |  | 8.32 | 88.39 | 84.44 |  |  |
| 8.86 | 92.55 | 90.67 |  |  | 8.33 | 88.38 | 84.40 |  |  |
| 8.87 | 92.54 | 90.64 |  |  | 8.34 | 88.38 | 84.36 |  |  |
| 8.88 | 92.54 | 90.60 |  |  | 8.35 | 88.35 | 84.33 |  |  |
| 8.89 | 92.53 | 90.56 |  |  | 8.36 | 88.33 | 84.29 |  |  |
| 8.90 | 92.52 | 90.52 |  |  | 8.37 | 88.31 | 84.25 |  |  |
| 8.91 | 92.51 | 90.48 |  |  | 8.38 | 88.29 | 84.21 |  |  |
| 8.92 | 92.51 | 90.44 |  |  | 8.39 | 88.27 | 84.17 |  |  |
| 8.93 | 92.49 | 90.40 |  |  | 8.40 | 88.26 | 84.14 |  |  |
| 8.94 | 92.49 | 90.37 |  |  | 8.41 | 88.24 | 84.10 |  |  |
| 8.95 | 92.48 | 90.33 |  |  | 8.42 | 88.23 | 84.06 |  |  |
| 8.96 | 92.48 | 90.29 |  |  | 8.45 | 88.17 | 83.95 |  |  |
| 8.97 | 92.47 | 90.25 |  |  | 8.46 | 88.16 | 83.91 |  |  |
| 8.98 | 92.46 | 90.21 |  |  | 8.47 | 88.13 | 83.87 |  |  |
| 9.00 | 92.44 | 90.14 |  |  | 8.48 | 88.11 | 83.83 |  |  |
| 9.01 | 92.44 | 90.10 |  |  | 8.49 | 88.09 | 83.79 |  |  |
| 9.02 | 92.42 | 90.06 |  |  | 8.50 | 88.08 | 83.76 |  |  |
| 9.03 | 92.42 | 90.02 |  |  | 8.51 | 88.05 | 83.72 |  |  |
| 9.04 | 92.41 | 89.98 |  |  | 8.52 | 88.05 | 83.68 |  |  |
| 9.06 | 92.40 | 89.90 |  |  | 8.53 | 88.03 | 83.64 |  |  |
| 9.07 | 92.39 | 89.87 |  |  | 8.54 | 88.01 | 83.60 |  |  |
| 9.08 | 92.38 | 89.83 |  |  | 8.55 | 87.99 | 83.57 |  |  |
| 9.09 | 92.38 | 89.79 |  |  | 8.56 | 87.98 | 83.53 |  |  |
| 9.10 | 92.37 | 89.75 |  |  | 8.57 | 87.97 | 83.49 |  |  |
| 9.11 | 92.36 | 89.71 |  |  | 8.58 | 87.95 | 83.45 |  |  |
| 9.12 | 92.33 | 89.67 |  |  | 8.59 | 87.93 | 83.41 |  |  |
| 9.13 | 92.32 | 89.64 |  |  | 8.60 | 87.91 | 83.38 |  |  |
| 9.14 | 92.32 | 89.60 |  |  | 8.61 | 87.89 | 83.34 |  |  |
| 9.16 | 92.31 | 89.52 |  |  | 8.62 | 87.88 | 83.30 |  |  |
| 9.17 | 92.30 | 89.48 |  |  | 8.63 | 87.86 | 83.26 |  |  |
| 9.18 | 92.30 | 89.44 |  |  | 8.64 | 87.85 | 83.22 |  |  |
| 9.19 | 92.28 | 89.40 |  |  | 8.65 | 87.83 | 83.18 |  |  |
| 9.20 | 92.28 | 89.37 |  |  | 8.66 | 87.81 | 83.15 |  |  |
| 9.21 | 92.27 | 89.33 |  |  | 8.67 | 87.79 | 83.11 |  |  |
| 9.22 | 92.25 | 89.29 |  |  | 8.68 | 87.76 | 83.07 |  |  |
| 9.23 | 92.23 | 89.25 |  |  | 8.69 | 87.74 | 83.03 |  |  |
| 9.24 | 92.21 | 89.21 |  |  | 8.70 | 87.74 | 82.99 |  |  |
| 9.25 | 92.21 | 89.17 |  |  | 8.71 | 87.71 | 82.96 |  |  |
| 9.26 | 92.20 | 89.14 |  |  | 8.72 | 87.69 | 82.92 |  |  |
| 9.27 | 92.19 | 89.10 |  |  | 8.73 | 87.67 | 82.88 |  |  |
| 9.28 | 92.18 | 89.06 |  |  | 8.74 | 87.66 | 82.84 |  |  |
| 9.29 | 92.17 | 89.02 |  |  | 8.75 | 87.63 | 82.80 |  |  |
| 9.30 | 92.17 | 88.98 |  |  | 8.77 | 87.60 | 82.73 |  |  |
| 9.31 | 92.15 | 88.94 |  |  | 8.78 | 87.57 | 82.69 |  |  |
| 9.32 | 92.15 | 88.90 |  |  | 8.79 | 87.55 | 82.65 |  |  |
| 9.33 | 92.15 | 88.87 |  |  | 8.80 | 87.54 | 82.61 |  |  |
| 9.34 | 92.13 | 88.83 |  |  | 8.81 | 87.51 | 82.58 |  |  |
| 9.35 | 92.12 | 88.79 |  |  | 8.82 | 87.49 | 82.54 |  |  |
| 9.36 | 92.11 | 88.75 |  |  | 8.83 | 87.46 | 82.50 |  |  |
| 9.38 | 92.11 | 88.67 |  |  | 8.85 | 87.41 | 82.42 |  |  |
| 9.39 | 92.09 | 88.63 |  |  | 8.86 | 87.39 | 82.39 |  |  |
| 9.40 | 92.09 | 88.60 |  |  | 8.87 | 87.37 | 82.35 |  |  |
| 9.42 | 92.06 | 88.52 |  |  | 8.88 | 87.36 | 82.31 |  |  |
| 9.44 | 92.05 | 88.44 |  |  | 8.89 | 87.34 | 82.27 |  |  |
| 9.45 | 92.04 | 88.40 |  |  | 8.90 | 87.33 | 82.23 |  |  |
| 9.48 | 91.99 | 88.29 |  |  | 8.91 | 87.31 | 82.20 |  |  |
| 9.49 | 91.98 | 88.25 |  |  | 8.92 | 87.31 | 82.16 |  |  |
| 9.50 | 91.97 | 88.21 |  |  | 8.93 | 87.30 | 82.12 |  |  |
| 9.51 | 91.97 | 88.17 |  |  | 8.94 | 87.29 | 82.08 |  |  |
| 9.52 | 91.95 | 88.13 |  |  | 8.95 | 87.28 | 82.04 |  |  |
| 9.53 | 91.93 | 88.10 |  |  | 8.97 | 87.24 | 81.97 |  |  |
| 9.54 | 91.92 | 88.06 |  |  | 8.98 | 87.23 | 81.93 |  |  |
| 9.55 | 91.90 | 88.02 |  |  | 8.99 | 87.20 | 81.89 |  |  |
| 9.56 | 91.89 | 87.98 |  |  | 9.00 | 87.19 | 81.85 |  |  |
| 9.57 | 91.87 | 87.94 |  |  | 9.01 | 87.18 | 81.81 |  |  |
| 9.59 | 91.84 | 87.87 |  |  | 9.02 | 87.17 | 81.78 |  |  |
| 9.60 | 91.83 | 87.83 |  |  | 9.03 | 87.16 | 81.74 |  |  |
| 9.61 | 91.82 | 87.79 |  |  | 9.04 | 87.14 | 81.70 |  |  |
| 9.62 | 91.81 | 87.75 |  |  | 9.05 | 87.13 | 81.66 |  |  |
| 9.63 | 91.78 | 87.71 |  |  | 9.06 | 87.11 | 81.62 |  |  |
| 9.64 | 91.78 | 87.67 |  |  | 9.08 | 87.06 | 81.55 |  |  |
| 9.65 | 91.77 | 87.63 |  |  | 9.09 | 87.03 | 81.51 |  |  |
| 9.66 | 91.76 | 87.60 |  |  | 9.10 | 87.01 | 81.47 |  |  |
| 9.67 | 91.75 | 87.56 |  |  | 9.11 | 86.99 | 81.43 |  |  |
| 9.68 | 91.74 | 87.52 |  |  | 9.12 | 86.98 | 81.40 |  |  |
| 9.69 | 91.71 | 87.48 |  |  | 9.13 | 86.96 | 81.36 |  |  |
| 9.70 | 91.70 | 87.44 |  |  | 9.14 | 86.95 | 81.32 |  |  |
| 9.71 | 91.69 | 87.40 |  |  | 9.15 | 86.93 | 81.28 |  |  |
| 9.72 | 91.67 | 87.37 |  |  | 9.16 | 86.92 | 81.24 |  |  |
| 9.73 | 91.67 | 87.33 |  |  | 9.18 | 86.90 | 81.17 |  |  |
| 9.74 | 91.66 | 87.29 |  |  | 9.19 | 86.89 | 81.13 |  |  |
| 9.75 | 91.65 | 87.25 |  |  | 9.20 | 86.87 | 81.09 |  |  |
| 9.76 | 91.63 | 87.21 |  |  | 9.21 | 86.86 | 81.05 |  |  |
| 9.77 | 91.62 | 87.17 |  |  | 9.22 | 86.85 | 81.02 |  |  |
| 9.78 | 91.61 | 87.13 |  |  | 9.23 | 86.83 | 80.98 |  |  |
| 9.79 | 91.60 | 87.10 |  |  | 9.24 | 86.82 | 80.94 |  |  |
| 9.80 | 91.58 | 87.06 |  |  | 9.25 | 86.81 | 80.90 |  |  |
| 9.81 | 91.56 | 87.02 |  |  | 9.26 | 86.80 | 80.86 |  |  |
| 9.82 | 91.55 | 86.98 |  |  | 9.27 | 86.78 | 80.83 |  |  |
| 9.83 | 91.54 | 86.94 |  |  | 9.28 | 86.76 | 80.79 |  |  |
| 9.85 | 91.53 | 86.87 |  |  | 9.29 | 86.73 | 80.75 |  |  |
| 9.86 | 91.51 | 86.83 |  |  | 9.30 | 86.71 | 80.71 |  |  |
| 9.87 | 91.50 | 86.79 |  |  | 9.31 | 86.70 | 80.67 |  |  |
| 9.88 | 91.49 | 86.75 |  |  | 9.32 | 86.68 | 80.63 |  |  |
| 9.89 | 91.47 | 86.71 |  |  | 9.33 | 86.65 | 80.60 |  |  |
| 9.90 | 91.46 | 86.67 |  |  | 9.34 | 86.64 | 80.56 |  |  |
| 9.94 | 91.41 | 86.52 |  |  | 9.35 | 86.61 | 80.52 |  |  |
| 9.95 | 91.40 | 86.48 |  |  | 9.36 | 86.60 | 80.48 |  |  |
| 9.96 | 91.39 | 86.44 |  |  | 9.37 | 86.59 | 80.44 |  |  |
| 9.97 | 91.37 | 86.40 |  |  | 9.38 | 86.56 | 80.41 |  |  |
| 9.98 | 91.37 | 86.37 |  |  | 9.39 | 86.55 | 80.37 |  |  |
| 9.99 | 91.37 | 86.33 |  |  | 9.41 | 86.52 | 80.29 |  |  |
| 10.00 | 91.35 | 86.29 |  |  | 9.42 | 86.51 | 80.25 |  |  |
| 10.01 | 91.34 | 86.25 |  |  | 9.43 | 86.50 | 80.22 |  |  |
| 10.02 | 91.33 | 86.21 |  |  | 9.45 | 86.47 | 80.14 |  |  |
| 10.04 | 91.31 | 86.13 |  |  | 9.46 | 86.45 | 80.10 |  |  |
| 10.07 | 91.29 | 86.02 |  |  | 9.47 | 86.44 | 80.06 |  |  |
| 10.08 | 91.26 | 85.98 |  |  | 9.48 | 86.42 | 80.03 |  |  |
| 10.09 | 91.25 | 85.94 |  |  | 9.49 | 86.40 | 79.99 |  |  |
| 10.10 | 91.24 | 85.90 |  |  | 9.52 | 86.38 | 79.87 |  |  |
| 10.11 | 91.23 | 85.87 |  |  | 9.53 | 86.36 | 79.84 |  |  |
| 10.12 | 91.22 | 85.83 |  |  | 9.54 | 86.35 | 79.80 |  |  |
| 10.13 | 91.21 | 85.79 |  |  | 9.55 | 86.32 | 79.76 |  |  |
| 10.14 | 91.20 | 85.75 |  |  | 9.56 | 86.32 | 79.72 |  |  |
| 10.15 | 91.19 | 85.71 |  |  | 9.57 | 86.30 | 79.68 |  |  |
| 10.16 | 91.19 | 85.67 |  |  | 9.58 | 86.29 | 79.65 |  |  |
| 10.17 | 91.18 | 85.63 |  |  | 9.59 | 86.26 | 79.61 |  |  |
| 10.18 | 91.17 | 85.60 |  |  | 9.60 | 86.25 | 79.57 |  |  |
| 10.19 | 91.17 | 85.56 |  |  | 9.61 | 86.23 | 79.53 |  |  |
| 10.20 | 91.16 | 85.52 |  |  | 9.62 | 86.22 | 79.49 |  |  |
| 10.21 | 91.15 | 85.48 |  |  | 9.63 | 86.20 | 79.45 |  |  |
| 10.22 | 91.15 | 85.44 |  |  | 9.64 | 86.18 | 79.42 |  |  |
| 10.23 | 91.14 | 85.40 |  |  | 9.65 | 86.15 | 79.38 |  |  |
| 10.24 | 91.13 | 85.36 |  |  | 9.66 | 86.13 | 79.34 |  |  |
| 10.25 | 91.12 | 85.33 |  |  | 9.67 | 86.12 | 79.30 |  |  |
| 10.27 | 91.12 | 85.25 |  |  | 9.68 | 86.10 | 79.26 |  |  |
| 10.28 | 91.11 | 85.21 |  |  | 9.69 | 86.09 | 79.23 |  |  |
| 10.29 | 91.09 | 85.17 |  |  | 9.70 | 86.07 | 79.19 |  |  |
| 10.30 | 91.08 | 85.13 |  |  | 9.71 | 86.06 | 79.15 |  |  |
| 10.31 | 91.07 | 85.10 |  |  | 9.72 | 86.02 | 79.11 |  |  |
| 10.32 | 91.06 | 85.06 |  |  | 9.73 | 86.01 | 79.07 |  |  |
| 10.33 | 91.06 | 85.02 |  |  | 9.74 | 85.99 | 79.04 |  |  |
| 10.35 | 91.05 | 84.94 |  |  | 9.75 | 85.97 | 79.00 |  |  |
| 10.37 | 91.03 | 84.86 |  |  | 9.76 | 85.95 | 78.96 |  |  |
| 10.38 | 91.01 | 84.83 |  |  | 9.77 | 85.92 | 78.92 |  |  |
| 10.39 | 91.01 | 84.79 |  |  | 9.78 | 85.88 | 78.88 |  |  |
| 10.40 | 91.01 | 84.75 |  |  | 9.79 | 85.86 | 78.85 |  |  |
| 10.41 | 91.00 | 84.71 |  |  | 9.80 | 85.84 | 78.81 |  |  |
| 10.42 | 90.98 | 84.67 |  |  | 9.81 | 85.82 | 78.77 |  |  |
| 10.43 | 90.97 | 84.63 |  |  | 9.82 | 85.80 | 78.73 |  |  |
| 10.44 | 90.97 | 84.60 |  |  | 9.83 | 85.79 | 78.69 |  |  |
| 10.45 | 90.97 | 84.56 |  |  | 9.84 | 85.75 | 78.66 |  |  |
| 10.46 | 90.95 | 84.52 |  |  | 9.85 | 85.73 | 78.62 |  |  |
| 10.47 | 90.95 | 84.48 |  |  | 9.86 | 85.70 | 78.58 |  |  |
| 10.48 | 90.93 | 84.44 |  |  | 9.87 | 85.69 | 78.54 |  |  |
| 10.49 | 90.91 | 84.40 |  |  | 9.88 | 85.66 | 78.50 |  |  |
| 10.50 | 90.90 | 84.36 |  |  | 9.91 | 85.60 | 78.39 |  |  |
| 10.52 | 90.88 | 84.29 |  |  | 9.92 | 85.59 | 78.35 |  |  |
| 10.53 | 90.86 | 84.25 |  |  | 9.94 | 85.53 | 78.27 |  |  |
| 10.54 | 90.85 | 84.21 |  |  | 9.95 | 85.52 | 78.24 |  |  |
| 10.55 | 90.83 | 84.17 |  |  | 9.96 | 85.50 | 78.20 |  |  |
| 10.57 | 90.79 | 84.10 |  |  | 9.97 | 85.48 | 78.16 |  |  |
| 10.58 | 90.78 | 84.06 |  |  | 9.98 | 85.46 | 78.12 |  |  |
| 10.59 | 90.76 | 84.02 |  |  | 9.99 | 85.45 | 78.08 |  |  |
| 10.60 | 90.75 | 83.98 |  |  | 10.00 | 85.41 | 78.05 |  |  |
| 10.61 | 90.73 | 83.94 |  |  | 10.01 | 85.38 | 78.01 |  |  |
| 10.62 | 90.72 | 83.90 |  |  | 10.02 | 85.37 | 77.97 |  |  |
| 10.63 | 90.70 | 83.86 |  |  | 10.03 | 85.35 | 77.93 |  |  |
| 10.64 | 90.70 | 83.83 |  |  | 10.05 | 85.31 | 77.86 |  |  |
| 10.65 | 90.69 | 83.79 |  |  | 10.07 | 85.28 | 77.78 |  |  |
| 10.66 | 90.68 | 83.75 |  |  | 10.08 | 85.25 | 77.74 |  |  |
| 10.67 | 90.67 | 83.71 |  |  | 10.09 | 85.23 | 77.70 |  |  |
| 10.68 | 90.66 | 83.67 |  |  | 10.10 | 85.21 | 77.67 |  |  |
| 10.69 | 90.64 | 83.63 |  |  | 10.11 | 85.18 | 77.63 |  |  |
| 10.70 | 90.61 | 83.60 |  |  | 10.12 | 85.17 | 77.59 |  |  |
| 10.71 | 90.60 | 83.56 |  |  | 10.13 | 85.15 | 77.55 |  |  |
| 10.72 | 90.59 | 83.52 |  |  | 10.14 | 85.13 | 77.51 |  |  |
| 10.75 | 90.54 | 83.40 |  |  | 10.15 | 85.10 | 77.48 |  |  |
| 10.76 | 90.52 | 83.36 |  |  | 10.16 | 85.08 | 77.44 |  |  |
| 10.77 | 90.50 | 83.33 |  |  | 10.17 | 85.06 | 77.40 |  |  |
| 10.78 | 90.49 | 83.29 |  |  | 10.18 | 85.04 | 77.36 |  |  |
| 10.79 | 90.47 | 83.25 |  |  | 10.19 | 85.02 | 77.32 |  |  |
| 10.80 | 90.45 | 83.21 |  |  | 10.20 | 85.01 | 77.29 |  |  |
| 10.81 | 90.43 | 83.17 |  |  | 10.21 | 84.98 | 77.25 |  |  |
| 10.82 | 90.40 | 83.13 |  |  | 10.23 | 84.95 | 77.17 |  |  |
| 10.83 | 90.39 | 83.10 |  |  | 10.24 | 84.94 | 77.13 |  |  |
| 10.85 | 90.35 | 83.02 |  |  | 10.25 | 84.93 | 77.09 |  |  |
| 10.89 | 90.28 | 82.86 |  |  | 10.26 | 84.91 | 77.06 |  |  |
| 10.90 | 90.27 | 82.83 |  |  | 10.27 | 84.89 | 77.02 |  |  |
| 10.91 | 90.25 | 82.79 |  |  | 10.28 | 84.86 | 76.98 |  |  |
| 10.92 | 90.24 | 82.75 |  |  | 10.29 | 84.84 | 76.94 |  |  |
| 10.93 | 90.22 | 82.71 |  |  | 10.30 | 84.82 | 76.90 |  |  |
| 10.94 | 90.21 | 82.67 |  |  | 10.31 | 84.80 | 76.87 |  |  |
| 10.95 | 90.20 | 82.63 |  |  | 10.33 | 84.77 | 76.79 |  |  |
| 10.96 | 90.19 | 82.60 |  |  | 10.34 | 84.75 | 76.75 |  |  |
| 10.97 | 90.18 | 82.56 |  |  | 10.36 | 84.72 | 76.68 |  |  |
| 10.99 | 90.16 | 82.48 |  |  | 10.37 | 84.70 | 76.64 |  |  |
| 11.00 | 90.14 | 82.44 |  |  | 10.38 | 84.68 | 76.60 |  |  |
| 11.02 | 90.12 | 82.36 |  |  | 10.39 | 84.67 | 76.56 |  |  |
| 11.03 | 90.11 | 82.33 |  |  | 10.40 | 84.64 | 76.52 |  |  |
| 11.04 | 90.10 | 82.29 |  |  | 10.41 | 84.62 | 76.49 |  |  |
| 11.05 | 90.09 | 82.25 |  |  | 10.42 | 84.59 | 76.45 |  |  |
| 11.06 | 90.08 | 82.21 |  |  | 10.43 | 84.58 | 76.41 |  |  |
| 11.08 | 90.06 | 82.13 |  |  | 10.44 | 84.57 | 76.37 |  |  |
| 11.09 | 90.06 | 82.09 |  |  | 10.45 | 84.55 | 76.33 |  |  |
| 11.10 | 90.05 | 82.06 |  |  | 10.46 | 84.53 | 76.30 |  |  |
| 11.11 | 90.04 | 82.02 |  |  | 10.48 | 84.49 | 76.22 |  |  |
| 11.12 | 90.03 | 81.98 |  |  | 10.49 | 84.48 | 76.18 |  |  |
| 11.15 | 89.99 | 81.86 |  |  | 10.50 | 84.47 | 76.14 |  |  |
| 11.16 | 89.99 | 81.83 |  |  | 10.51 | 84.45 | 76.11 |  |  |
| 11.19 | 89.96 | 81.71 |  |  | 10.53 | 84.42 | 76.03 |  |  |
| 11.20 | 89.96 | 81.67 |  |  | 10.54 | 84.41 | 75.99 |  |  |
| 11.22 | 89.93 | 81.59 |  |  | 10.55 | 84.40 | 75.95 |  |  |
| 11.24 | 89.92 | 81.52 |  |  | 10.56 | 84.39 | 75.92 |  |  |
| 11.25 | 89.91 | 81.48 |  |  | 10.57 | 84.37 | 75.88 |  |  |
| 11.26 | 89.90 | 81.44 |  |  | 10.58 | 84.35 | 75.84 |  |  |
| 11.27 | 89.90 | 81.40 |  |  | 10.59 | 84.33 | 75.80 |  |  |
| 11.28 | 89.89 | 81.36 |  |  | 10.60 | 84.32 | 75.76 |  |  |
| 11.29 | 89.88 | 81.33 |  |  | 10.62 | 84.28 | 75.69 |  |  |
| 11.30 | 89.87 | 81.29 |  |  | 10.63 | 84.26 | 75.65 |  |  |
| 11.31 | 89.86 | 81.25 |  |  | 10.64 | 84.23 | 75.61 |  |  |
| 11.32 | 89.85 | 81.21 |  |  | 10.65 | 84.21 | 75.57 |  |  |
| 11.33 | 89.84 | 81.17 |  |  | 10.66 | 84.19 | 75.53 |  |  |
| 11.36 | 89.81 | 81.06 |  |  | 10.67 | 84.18 | 75.50 |  |  |
| 11.37 | 89.81 | 81.02 |  |  | 10.69 | 84.16 | 75.42 |  |  |
| 11.38 | 89.81 | 80.98 |  |  | 10.70 | 84.14 | 75.38 |  |  |
| 11.39 | 89.80 | 80.94 |  |  | 10.71 | 84.12 | 75.34 |  |  |
| 11.40 | 89.78 | 80.90 |  |  | 10.73 | 84.08 | 75.27 |  |  |
| 11.41 | 89.78 | 80.86 |  |  | 10.74 | 84.07 | 75.23 |  |  |
| 11.42 | 89.76 | 80.83 |  |  | 10.75 | 84.05 | 75.19 |  |  |
| 11.43 | 89.75 | 80.79 |  |  | 10.76 | 84.03 | 75.15 |  |  |
| 11.45 | 89.74 | 80.71 |  |  | 10.77 | 84.01 | 75.12 |  |  |
| 11.46 | 89.73 | 80.67 |  |  | 10.78 | 83.99 | 75.08 |  |  |
| 11.47 | 89.73 | 80.63 |  |  | 10.79 | 83.97 | 75.04 |  |  |
| 11.50 | 89.70 | 80.52 |  |  | 10.80 | 83.96 | 75.00 |  |  |
| 11.52 | 89.69 | 80.44 |  |  | 10.81 | 83.94 | 74.96 |  |  |
| 11.53 | 89.68 | 80.40 |  |  | 10.82 | 83.93 | 74.93 |  |  |
| 11.54 | 89.66 | 80.36 |  |  | 10.83 | 83.91 | 74.89 |  |  |
| 11.55 | 89.66 | 80.33 |  |  | 10.84 | 83.89 | 74.85 |  |  |
| 11.56 | 89.64 | 80.29 |  |  | 10.85 | 83.88 | 74.81 |  |  |
| 11.57 | 89.62 | 80.25 |  |  | 10.86 | 83.86 | 74.77 |  |  |
| 11.58 | 89.61 | 80.21 |  |  | 10.87 | 83.84 | 74.74 |  |  |
| 11.59 | 89.60 | 80.17 |  |  | 10.88 | 83.82 | 74.70 |  |  |
| 11.61 | 89.58 | 80.09 |  |  | 10.89 | 83.81 | 74.66 |  |  |
| 11.62 | 89.57 | 80.06 |  |  | 10.90 | 83.79 | 74.62 |  |  |
| 11.63 | 89.57 | 80.02 |  |  | 10.91 | 83.77 | 74.58 |  |  |
| 11.64 | 89.56 | 79.98 |  |  | 10.92 | 83.76 | 74.54 |  |  |
| 11.65 | 89.55 | 79.94 |  |  | 10.93 | 83.73 | 74.51 |  |  |
| 11.66 | 89.54 | 79.90 |  |  | 10.94 | 83.71 | 74.47 |  |  |
| 11.67 | 89.54 | 79.86 |  |  | 10.96 | 83.68 | 74.39 |  |  |
| 11.68 | 89.52 | 79.83 |  |  | 10.97 | 83.67 | 74.35 |  |  |
| 11.69 | 89.51 | 79.79 |  |  | 10.98 | 83.65 | 74.32 |  |  |
| 11.70 | 89.50 | 79.75 |  |  | 10.99 | 83.64 | 74.28 |  |  |
| 11.71 | 89.49 | 79.71 |  |  | 11.00 | 83.62 | 74.24 |  |  |
| 11.72 | 89.48 | 79.67 |  |  | 11.02 | 83.60 | 74.16 |  |  |
| 11.73 | 89.48 | 79.63 |  |  | 11.03 | 83.57 | 74.13 |  |  |
| 11.74 | 89.46 | 79.59 |  |  | 11.04 | 83.55 | 74.09 |  |  |
| 11.75 | 89.44 | 79.56 |  |  | 11.05 | 83.53 | 74.05 |  |  |
| 11.76 | 89.43 | 79.52 |  |  | 11.06 | 83.52 | 74.01 |  |  |
| 11.77 | 89.42 | 79.48 |  |  | 11.07 | 83.50 | 73.97 |  |  |
| 11.78 | 89.40 | 79.44 |  |  | 11.08 | 83.49 | 73.94 |  |  |
| 11.79 | 89.38 | 79.40 |  |  | 11.09 | 83.46 | 73.90 |  |  |
| 11.80 | 89.37 | 79.36 |  |  | 11.10 | 83.45 | 73.86 |  |  |
| 11.81 | 89.36 | 79.33 |  |  | 11.11 | 83.44 | 73.82 |  |  |
| 11.82 | 89.36 | 79.29 |  |  | 11.12 | 83.42 | 73.78 |  |  |
| 11.83 | 89.34 | 79.25 |  |  | 11.13 | 83.40 | 73.75 |  |  |
| 11.84 | 89.33 | 79.21 |  |  | 11.14 | 83.38 | 73.71 |  |  |
| 11.85 | 89.32 | 79.17 |  |  | 11.15 | 83.36 | 73.67 |  |  |
| 11.86 | 89.31 | 79.13 |  |  | 11.16 | 83.35 | 73.63 |  |  |
| 11.87 | 89.30 | 79.09 |  |  | 11.17 | 83.33 | 73.59 |  |  |
| 11.89 | 89.26 | 79.02 |  |  | 11.18 | 83.32 | 73.56 |  |  |
| 11.90 | 89.23 | 78.98 |  |  | 11.19 | 83.30 | 73.52 |  |  |
| 11.91 | 89.22 | 78.94 |  |  | 11.21 | 83.24 | 73.44 |  |  |
| 11.92 | 89.21 | 78.90 |  |  | 11.23 | 83.20 | 73.36 |  |  |
| 11.93 | 89.20 | 78.86 |  |  | 11.24 | 83.17 | 73.33 |  |  |
| 11.94 | 89.19 | 78.82 |  |  | 11.26 | 83.12 | 73.25 |  |  |
| 11.95 | 89.18 | 78.79 |  |  | 11.27 | 83.08 | 73.21 |  |  |
| 11.96 | 89.17 | 78.75 |  |  | 11.28 | 83.06 | 73.17 |  |  |
| 11.97 | 89.16 | 78.71 |  |  | 11.29 | 83.04 | 73.14 |  |  |
| 11.98 | 89.14 | 78.67 |  |  | 11.30 | 83.02 | 73.10 |  |  |
| 11.99 | 89.14 | 78.63 |  |  | 11.31 | 83.00 | 73.06 |  |  |
| 12.00 | 89.12 | 78.59 |  |  | 11.32 | 82.97 | 73.02 |  |  |
| 12.01 | 89.11 | 78.56 |  |  | 11.33 | 82.95 | 72.98 |  |  |
| 12.02 | 89.10 | 78.52 |  |  | 11.34 | 82.93 | 72.95 |  |  |
| 12.03 | 89.09 | 78.48 |  |  | 11.35 | 82.91 | 72.91 |  |  |
| 12.04 | 89.08 | 78.44 |  |  | 11.36 | 82.88 | 72.87 |  |  |
| 12.05 | 89.07 | 78.40 |  |  | 11.37 | 82.86 | 72.83 |  |  |
| 12.06 | 89.07 | 78.36 |  |  | 11.38 | 82.84 | 72.79 |  |  |
| 12.07 | 89.05 | 78.32 |  |  | 11.39 | 82.81 | 72.76 |  |  |
| 12.08 | 89.04 | 78.29 |  |  | 11.41 | 82.75 | 72.68 |  |  |
| 12.09 | 89.03 | 78.25 |  |  | 11.42 | 82.73 | 72.64 |  |  |
| 12.10 | 89.02 | 78.21 |  |  | 11.43 | 82.70 | 72.60 |  |  |
| 12.11 | 89.01 | 78.17 |  |  | 11.45 | 82.66 | 72.53 |  |  |
| 12.12 | 89.01 | 78.13 |  |  | 11.46 | 82.64 | 72.49 |  |  |
| 12.13 | 88.99 | 78.09 |  |  | 11.47 | 82.61 | 72.45 |  |  |
| 12.14 | 88.99 | 78.06 |  |  | 11.48 | 82.60 | 72.41 |  |  |
| 12.15 | 88.97 | 78.02 |  |  | 11.49 | 82.57 | 72.38 |  |  |
| 12.16 | 88.97 | 77.98 |  |  | 11.50 | 82.55 | 72.34 |  |  |
| 12.17 | 88.96 | 77.94 |  |  | 11.51 | 82.52 | 72.30 |  |  |
| 12.18 | 88.95 | 77.90 |  |  | 11.52 | 82.49 | 72.26 |  |  |
| 12.19 | 88.94 | 77.86 |  |  | 11.53 | 82.47 | 72.22 |  |  |
| 12.20 | 88.94 | 77.82 |  |  | 11.54 | 82.45 | 72.19 |  |  |
| 12.21 | 88.92 | 77.79 |  |  | 11.55 | 82.44 | 72.15 |  |  |
| 12.22 | 88.91 | 77.75 |  |  | 11.56 | 82.41 | 72.11 |  |  |
| 12.23 | 88.90 | 77.71 |  |  | 11.57 | 82.39 | 72.07 |  |  |
| 12.24 | 88.89 | 77.67 |  |  | 11.58 | 82.37 | 72.03 |  |  |
| 12.25 | 88.87 | 77.63 |  |  | 11.60 | 82.34 | 71.96 |  |  |
| 12.26 | 88.87 | 77.59 |  |  | 11.62 | 82.29 | 71.88 |  |  |
| 12.27 | 88.86 | 77.56 |  |  | 11.65 | 82.20 | 71.77 |  |  |
| 12.28 | 88.84 | 77.52 |  |  | 11.66 | 82.19 | 71.73 |  |  |
| 12.29 | 88.84 | 77.48 |  |  | 11.67 | 82.17 | 71.69 |  |  |
| 12.32 | 88.80 | 77.36 |  |  | 11.68 | 82.12 | 71.65 |  |  |
| 12.33 | 88.80 | 77.32 |  |  | 11.69 | 82.09 | 71.61 |  |  |
| 12.34 | 88.79 | 77.29 |  |  | 11.70 | 82.08 | 71.58 |  |  |
| 12.35 | 88.77 | 77.25 |  |  | 11.72 | 82.04 | 71.50 |  |  |
| 12.36 | 88.76 | 77.21 |  |  | 11.73 | 82.02 | 71.46 |  |  |
| 12.37 | 88.75 | 77.17 |  |  | 11.74 | 82.00 | 71.42 |  |  |
| 12.38 | 88.73 | 77.13 |  |  | 11.75 | 81.98 | 71.39 |  |  |
| 12.39 | 88.72 | 77.09 |  |  | 11.76 | 81.95 | 71.35 |  |  |
| 12.40 | 88.71 | 77.06 |  |  | 11.77 | 81.92 | 71.31 |  |  |
| 12.42 | 88.67 | 76.98 |  |  | 11.78 | 81.89 | 71.27 |  |  |
| 12.44 | 88.65 | 76.90 |  |  | 11.81 | 81.80 | 71.16 |  |  |
| 12.46 | 88.62 | 76.82 |  |  | 11.82 | 81.78 | 71.12 |  |  |
| 12.48 | 88.58 | 76.75 |  |  | 11.83 | 81.73 | 71.08 |  |  |
| 12.49 | 88.56 | 76.71 |  |  | 11.84 | 81.71 | 71.04 |  |  |
| 12.50 | 88.55 | 76.67 |  |  | 11.85 | 81.68 | 71.01 |  |  |
| 12.51 | 88.53 | 76.63 |  |  | 11.86 | 81.65 | 70.97 |  |  |
| 12.52 | 88.51 | 76.59 |  |  | 11.87 | 81.62 | 70.93 |  |  |
| 12.53 | 88.50 | 76.56 |  |  | 11.88 | 81.59 | 70.89 |  |  |
| 12.54 | 88.50 | 76.52 |  |  | 11.89 | 81.57 | 70.85 |  |  |
| 12.55 | 88.49 | 76.48 |  |  | 11.90 | 81.54 | 70.81 |  |  |
| 12.56 | 88.47 | 76.44 |  |  | 11.91 | 81.51 | 70.78 |  |  |
| 12.57 | 88.46 | 76.40 |  |  | 11.92 | 81.50 | 70.74 |  |  |
| 12.58 | 88.44 | 76.36 |  |  | 11.93 | 81.47 | 70.70 |  |  |
| 12.59 | 88.42 | 76.32 |  |  | 11.94 | 81.45 | 70.66 |  |  |
| 12.61 | 88.40 | 76.25 |  |  | 11.96 | 81.38 | 70.59 |  |  |
| 12.62 | 88.39 | 76.21 |  |  | 11.97 | 81.35 | 70.55 |  |  |
| 12.63 | 88.38 | 76.17 |  |  | 11.98 | 81.32 | 70.51 |  |  |
| 12.64 | 88.37 | 76.13 |  |  | 12.00 | 81.25 | 70.43 |  |  |
| 12.65 | 88.35 | 76.09 |  |  | 12.02 | 81.19 | 70.36 |  |  |
| 12.66 | 88.34 | 76.06 |  |  | 12.04 | 81.15 | 70.28 |  |  |
| 12.67 | 88.32 | 76.02 |  |  | 12.05 | 81.11 | 70.24 |  |  |
| 12.68 | 88.30 | 75.98 |  |  | 12.06 | 81.10 | 70.21 |  |  |
| 12.69 | 88.30 | 75.94 |  |  | 12.07 | 81.08 | 70.17 |  |  |
| 12.71 | 88.28 | 75.86 |  |  | 12.08 | 81.05 | 70.13 |  |  |
| 12.72 | 88.26 | 75.82 |  |  | 12.09 | 81.01 | 70.09 |  |  |
| 12.73 | 88.23 | 75.79 |  |  | 12.10 | 80.98 | 70.05 |  |  |
| 12.74 | 88.22 | 75.75 |  |  | 12.11 | 80.95 | 70.02 |  |  |
| 12.75 | 88.20 | 75.71 |  |  | 12.12 | 80.93 | 69.98 |  |  |
| 12.76 | 88.19 | 75.67 |  |  | 12.14 | 80.89 | 69.90 |  |  |
| 12.77 | 88.17 | 75.63 |  |  | 12.15 | 80.86 | 69.86 |  |  |
| 12.78 | 88.15 | 75.59 |  |  | 12.16 | 80.83 | 69.83 |  |  |
| 12.79 | 88.13 | 75.56 |  |  | 12.17 | 80.81 | 69.79 |  |  |
| 12.80 | 88.11 | 75.52 |  |  | 12.18 | 80.78 | 69.75 |  |  |
| 12.81 | 88.09 | 75.48 |  |  | 12.19 | 80.76 | 69.71 |  |  |
| 12.82 | 88.07 | 75.44 |  |  | 12.20 | 80.73 | 69.67 |  |  |
| 12.83 | 88.04 | 75.40 |  |  | 12.21 | 80.72 | 69.63 |  |  |
| 12.84 | 88.01 | 75.36 |  |  | 12.22 | 80.69 | 69.60 |  |  |
| 12.85 | 88.00 | 75.32 |  |  | 12.23 | 80.68 | 69.56 |  |  |
| 12.87 | 87.98 | 75.25 |  |  | 12.24 | 80.65 | 69.52 |  |  |
| 12.88 | 87.96 | 75.21 |  |  | 12.25 | 80.64 | 69.48 |  |  |
| 12.89 | 87.93 | 75.17 |  |  | 12.26 | 80.61 | 69.44 |  |  |
| 12.90 | 87.92 | 75.13 |  |  | 12.27 | 80.60 | 69.41 |  |  |
| 12.91 | 87.88 | 75.09 |  |  | 12.28 | 80.58 | 69.37 |  |  |
| 12.92 | 87.86 | 75.05 |  |  | 12.29 | 80.56 | 69.33 |  |  |
| 12.93 | 87.83 | 75.02 |  |  | 12.30 | 80.53 | 69.29 |  |  |
| 12.94 | 87.82 | 74.98 |  |  | 12.31 | 80.52 | 69.25 |  |  |
| 12.96 | 87.78 | 74.90 |  |  | 12.32 | 80.50 | 69.22 |  |  |
| 12.97 | 87.76 | 74.86 |  |  | 12.33 | 80.48 | 69.18 |  |  |
| 12.98 | 87.74 | 74.82 |  |  | 12.34 | 80.46 | 69.14 |  |  |
| 12.99 | 87.72 | 74.79 |  |  | 12.35 | 80.44 | 69.10 |  |  |
| 13.00 | 87.71 | 74.75 |  |  | 12.36 | 80.41 | 69.06 |  |  |
| 13.01 | 87.69 | 74.71 |  |  | 12.37 | 80.38 | 69.03 |  |  |
| 13.02 | 87.67 | 74.67 |  |  | 12.39 | 80.33 | 68.95 |  |  |
| 13.04 | 87.63 | 74.59 |  |  | 12.40 | 80.31 | 68.91 |  |  |
| 13.05 | 87.63 | 74.55 |  |  | 12.41 | 80.29 | 68.87 |  |  |
| 13.06 | 87.61 | 74.52 |  |  | 12.42 | 80.26 | 68.84 |  |  |
| 13.07 | 87.59 | 74.48 |  |  | 12.43 | 80.25 | 68.80 |  |  |
| 13.08 | 87.58 | 74.44 |  |  | 12.44 | 80.22 | 68.76 |  |  |
| 13.09 | 87.57 | 74.40 |  |  | 12.45 | 80.21 | 68.72 |  |  |
| 13.11 | 87.53 | 74.32 |  |  | 12.46 | 80.18 | 68.68 |  |  |
| 13.13 | 87.50 | 74.25 |  |  | 12.47 | 80.15 | 68.65 |  |  |
| 13.14 | 87.46 | 74.21 |  |  | 12.48 | 80.12 | 68.61 |  |  |
| 13.15 | 87.44 | 74.17 |  |  | 12.49 | 80.10 | 68.57 |  |  |
| 13.16 | 87.41 | 74.13 |  |  | 12.50 | 80.08 | 68.53 |  |  |
| 13.17 | 87.39 | 74.09 |  |  | 12.51 | 80.06 | 68.49 |  |  |
| 13.18 | 87.38 | 74.05 |  |  | 12.52 | 80.04 | 68.46 |  |  |
| 13.19 | 87.34 | 74.02 |  |  | 12.54 | 80.00 | 68.38 |  |  |
| 13.20 | 87.31 | 73.98 |  |  | 12.55 | 79.97 | 68.34 |  |  |
| 13.21 | 87.29 | 73.94 |  |  | 12.56 | 79.94 | 68.30 |  |  |
| 13.22 | 87.27 | 73.90 |  |  | 12.57 | 79.90 | 68.26 |  |  |
| 13.24 | 87.22 | 73.82 |  |  | 12.58 | 79.87 | 68.23 |  |  |
| 13.26 | 87.20 | 73.75 |  |  | 12.59 | 79.83 | 68.19 |  |  |
| 13.27 | 87.18 | 73.71 |  |  | 12.60 | 79.80 | 68.15 |  |  |
| 13.28 | 87.17 | 73.67 |  |  | 12.61 | 79.77 | 68.11 |  |  |
| 13.30 | 87.14 | 73.59 |  |  | 12.62 | 79.72 | 68.07 |  |  |
| 13.31 | 87.11 | 73.55 |  |  | 12.63 | 79.69 | 68.04 |  |  |
| 13.32 | 87.10 | 73.52 |  |  | 12.65 | 79.63 | 67.96 |  |  |
| 13.33 | 87.08 | 73.48 |  |  | 12.66 | 79.61 | 67.92 |  |  |
| 13.34 | 87.07 | 73.44 |  |  | 12.67 | 79.58 | 67.88 |  |  |
| 13.35 | 87.05 | 73.40 |  |  | 12.68 | 79.55 | 67.85 |  |  |
| 13.36 | 87.03 | 73.36 |  |  | 12.69 | 79.50 | 67.81 |  |  |
| 13.37 | 87.00 | 73.32 |  |  | 12.70 | 79.46 | 67.77 |  |  |
| 13.38 | 86.99 | 73.29 |  |  | 12.72 | 79.40 | 67.69 |  |  |
| 13.39 | 86.97 | 73.25 |  |  | 12.73 | 79.37 | 67.66 |  |  |
| 13.42 | 86.90 | 73.13 |  |  | 12.74 | 79.34 | 67.62 |  |  |
| 13.43 | 86.89 | 73.09 |  |  | 12.75 | 79.32 | 67.58 |  |  |
| 13.45 | 86.86 | 73.02 |  |  | 12.76 | 79.27 | 67.54 |  |  |
| 13.46 | 86.85 | 72.98 |  |  | 12.78 | 79.21 | 67.47 |  |  |
| 13.47 | 86.84 | 72.94 |  |  | 12.79 | 79.18 | 67.43 |  |  |
| 13.48 | 86.82 | 72.90 |  |  | 12.80 | 79.15 | 67.39 |  |  |
| 13.49 | 86.81 | 72.86 |  |  | 12.82 | 79.11 | 67.31 |  |  |
| 13.50 | 86.78 | 72.82 |  |  | 12.84 | 79.04 | 67.24 |  |  |
| 13.51 | 86.76 | 72.79 |  |  | 12.85 | 79.02 | 67.20 |  |  |
| 13.52 | 86.75 | 72.75 |  |  | 12.87 | 78.94 | 67.12 |  |  |
| 13.53 | 86.73 | 72.71 |  |  | 12.88 | 78.91 | 67.08 |  |  |
| 13.54 | 86.71 | 72.67 |  |  | 12.89 | 78.88 | 67.05 |  |  |
| 13.55 | 86.69 | 72.63 |  |  | 12.90 | 78.85 | 67.01 |  |  |
| 13.56 | 86.67 | 72.59 |  |  | 12.91 | 78.81 | 66.97 |  |  |
| 13.57 | 86.64 | 72.55 |  |  | 12.93 | 78.77 | 66.89 |  |  |
| 13.58 | 86.63 | 72.52 |  |  | 12.94 | 78.74 | 66.86 |  |  |
| 13.59 | 86.62 | 72.48 |  |  | 12.95 | 78.70 | 66.82 |  |  |
| 13.60 | 86.60 | 72.44 |  |  | 12.96 | 78.66 | 66.78 |  |  |
| 13.61 | 86.58 | 72.40 |  |  | 12.97 | 78.61 | 66.74 |  |  |
| 13.62 | 86.54 | 72.36 |  |  | 12.98 | 78.60 | 66.70 |  |  |
| 13.63 | 86.51 | 72.32 |  |  | 12.99 | 78.57 | 66.67 |  |  |
| 13.64 | 86.48 | 72.29 |  |  | 13.00 | 78.55 | 66.63 |  |  |
| 13.65 | 86.46 | 72.25 |  |  | 13.01 | 78.52 | 66.59 |  |  |
| 13.66 | 86.44 | 72.21 |  |  | 13.02 | 78.49 | 66.55 |  |  |
| 13.68 | 86.40 | 72.13 |  |  | 13.03 | 78.47 | 66.51 |  |  |
| 13.69 | 86.38 | 72.09 |  |  | 13.04 | 78.44 | 66.48 |  |  |
| 13.70 | 86.36 | 72.05 |  |  | 13.05 | 78.38 | 66.44 |  |  |
| 13.71 | 86.33 | 72.02 |  |  | 13.06 | 78.35 | 66.40 |  |  |
| 13.73 | 86.28 | 71.94 |  |  | 13.07 | 78.30 | 66.36 |  |  |
| 13.75 | 86.25 | 71.86 |  |  | 13.08 | 78.26 | 66.32 |  |  |
| 13.76 | 86.23 | 71.82 |  |  | 13.09 | 78.23 | 66.29 |  |  |
| 13.77 | 86.20 | 71.78 |  |  | 13.10 | 78.20 | 66.25 |  |  |
| 13.78 | 86.18 | 71.75 |  |  | 13.11 | 78.19 | 66.21 |  |  |
| 13.79 | 86.16 | 71.71 |  |  | 13.12 | 78.15 | 66.17 |  |  |
| 13.80 | 86.15 | 71.67 |  |  | 13.13 | 78.13 | 66.13 |  |  |
| 13.81 | 86.13 | 71.63 |  |  | 13.14 | 78.09 | 66.10 |  |  |
| 13.82 | 86.12 | 71.59 |  |  | 13.15 | 78.07 | 66.06 |  |  |
| 13.83 | 86.10 | 71.55 |  |  | 13.16 | 78.05 | 66.02 |  |  |
| 13.84 | 86.07 | 71.52 |  |  | 13.17 | 78.02 | 65.98 |  |  |
| 13.85 | 86.06 | 71.48 |  |  | 13.18 | 77.99 | 65.94 |  |  |
| 13.86 | 86.04 | 71.44 |  |  | 13.19 | 77.95 | 65.90 |  |  |
| 13.87 | 86.02 | 71.40 |  |  | 13.22 | 77.87 | 65.79 |  |  |
| 13.88 | 85.99 | 71.36 |  |  | 13.23 | 77.84 | 65.75 |  |  |
| 13.89 | 85.97 | 71.32 |  |  | 13.24 | 77.81 | 65.71 |  |  |
| 13.90 | 85.95 | 71.28 |  |  | 13.25 | 77.77 | 65.68 |  |  |
| 13.91 | 85.93 | 71.25 |  |  | 13.26 | 77.74 | 65.64 |  |  |
| 13.92 | 85.90 | 71.21 |  |  | 13.27 | 77.71 | 65.60 |  |  |
| 13.93 | 85.88 | 71.17 |  |  | 13.28 | 77.69 | 65.56 |  |  |
| 13.94 | 85.85 | 71.13 |  |  | 13.29 | 77.66 | 65.52 |  |  |
| 13.95 | 85.83 | 71.09 |  |  | 13.30 | 77.63 | 65.49 |  |  |
| 13.96 | 85.81 | 71.05 |  |  | 13.31 | 77.62 | 65.45 |  |  |
| 13.97 | 85.80 | 71.02 |  |  | 13.32 | 77.59 | 65.41 |  |  |
| 13.98 | 85.78 | 70.98 |  |  | 13.33 | 77.55 | 65.37 |  |  |
| 13.99 | 85.75 | 70.94 |  |  | 13.34 | 77.53 | 65.33 |  |  |
| 14.00 | 85.72 | 70.90 |  |  | 13.35 | 77.51 | 65.30 |  |  |
| 14.01 | 85.71 | 70.86 |  |  | 13.36 | 77.48 | 65.26 |  |  |
| 14.02 | 85.68 | 70.82 |  |  | 13.37 | 77.45 | 65.22 |  |  |
| 14.03 | 85.67 | 70.78 |  |  | 13.38 | 77.41 | 65.18 |  |  |
| 14.04 | 85.66 | 70.75 |  |  | 13.39 | 77.39 | 65.14 |  |  |
| 14.05 | 85.62 | 70.71 |  |  | 13.41 | 77.33 | 65.07 |  |  |
| 14.06 | 85.59 | 70.67 |  |  | 13.43 | 77.25 | 64.99 |  |  |
| 14.07 | 85.57 | 70.63 |  |  | 13.44 | 77.21 | 64.95 |  |  |
| 14.10 | 85.51 | 70.52 |  |  | 13.45 | 77.17 | 64.92 |  |  |
| 14.11 | 85.48 | 70.48 |  |  | 13.46 | 77.14 | 64.88 |  |  |
| 14.12 | 85.45 | 70.44 |  |  | 13.47 | 77.12 | 64.84 |  |  |
| 14.13 | 85.43 | 70.40 |  |  | 13.48 | 77.08 | 64.80 |  |  |
| 14.14 | 85.42 | 70.36 |  |  | 13.49 | 77.05 | 64.76 |  |  |
| 14.17 | 85.35 | 70.25 |  |  | 13.50 | 77.00 | 64.72 |  |  |
| 14.18 | 85.32 | 70.21 |  |  | 13.51 | 76.96 | 64.69 |  |  |
| 14.20 | 85.28 | 70.13 |  |  | 13.52 | 76.90 | 64.65 |  |  |
| 14.21 | 85.24 | 70.09 |  |  | 13.53 | 76.87 | 64.61 |  |  |
| 14.22 | 85.22 | 70.05 |  |  | 13.54 | 76.83 | 64.57 |  |  |
| 14.23 | 85.19 | 70.02 |  |  | 13.55 | 76.79 | 64.53 |  |  |
| 14.24 | 85.15 | 69.98 |  |  | 13.56 | 76.73 | 64.50 |  |  |
| 14.25 | 85.13 | 69.94 |  |  | 13.57 | 76.70 | 64.46 |  |  |
| 14.26 | 85.09 | 69.90 |  |  | 13.58 | 76.67 | 64.42 |  |  |
| 14.27 | 85.07 | 69.86 |  |  | 13.59 | 76.64 | 64.38 |  |  |
| 14.28 | 85.06 | 69.82 |  |  | 13.60 | 76.59 | 64.34 |  |  |
| 14.29 | 85.06 | 69.78 |  |  | 13.61 | 76.55 | 64.31 |  |  |
| 14.30 | 85.04 | 69.75 |  |  | 13.62 | 76.53 | 64.27 |  |  |
| 14.32 | 84.99 | 69.67 |  |  | 13.63 | 76.49 | 64.23 |  |  |
| 14.33 | 84.95 | 69.63 |  |  | 13.64 | 76.44 | 64.19 |  |  |
| 14.34 | 84.93 | 69.59 |  |  | 13.65 | 76.40 | 64.15 |  |  |
| 14.35 | 84.91 | 69.55 |  |  | 13.66 | 76.35 | 64.12 |  |  |
| 14.36 | 84.89 | 69.52 |  |  | 13.67 | 76.32 | 64.08 |  |  |
| 14.37 | 84.86 | 69.48 |  |  | 13.68 | 76.28 | 64.04 |  |  |
| 14.38 | 84.84 | 69.44 |  |  | 13.69 | 76.24 | 64.00 |  |  |
| 14.39 | 84.81 | 69.40 |  |  | 13.70 | 76.19 | 63.96 |  |  |
| 14.40 | 84.78 | 69.36 |  |  | 13.71 | 76.15 | 63.93 |  |  |
| 14.41 | 84.76 | 69.32 |  |  | 13.72 | 76.12 | 63.89 |  |  |
| 14.43 | 84.71 | 69.25 |  |  | 13.73 | 76.08 | 63.85 |  |  |
| 14.44 | 84.69 | 69.21 |  |  | 13.74 | 76.02 | 63.81 |  |  |
| 14.45 | 84.67 | 69.17 |  |  | 13.75 | 75.96 | 63.77 |  |  |
| 14.46 | 84.64 | 69.13 |  |  | 13.76 | 75.92 | 63.74 |  |  |
| 14.47 | 84.60 | 69.09 |  |  | 13.77 | 75.88 | 63.70 |  |  |
| 14.49 | 84.55 | 69.02 |  |  | 13.78 | 75.85 | 63.66 |  |  |
| 14.50 | 84.52 | 68.98 |  |  | 13.80 | 75.76 | 63.58 |  |  |
| 14.53 | 84.44 | 68.86 |  |  | 13.81 | 75.72 | 63.55 |  |  |
| 14.54 | 84.40 | 68.82 |  |  | 13.82 | 75.68 | 63.51 |  |  |
| 14.55 | 84.38 | 68.78 |  |  | 13.83 | 75.63 | 63.47 |  |  |
| 14.56 | 84.34 | 68.75 |  |  | 13.84 | 75.60 | 63.43 |  |  |
| 14.57 | 84.32 | 68.71 |  |  | 13.85 | 75.57 | 63.39 |  |  |
| 14.58 | 84.29 | 68.67 |  |  | 13.86 | 75.54 | 63.35 |  |  |
| 14.60 | 84.23 | 68.59 |  |  | 13.87 | 75.48 | 63.32 |  |  |
| 14.61 | 84.19 | 68.55 |  |  | 13.88 | 75.45 | 63.28 |  |  |
| 14.62 | 84.17 | 68.51 |  |  | 13.89 | 75.41 | 63.24 |  |  |
| 14.63 | 84.14 | 68.48 |  |  | 13.90 | 75.36 | 63.20 |  |  |
| 14.64 | 84.11 | 68.44 |  |  | 13.91 | 75.32 | 63.16 |  |  |
| 14.65 | 84.07 | 68.40 |  |  | 13.92 | 75.28 | 63.13 |  |  |
| 14.67 | 84.02 | 68.32 |  |  | 13.93 | 75.23 | 63.09 |  |  |
| 14.68 | 83.99 | 68.28 |  |  | 13.94 | 75.20 | 63.05 |  |  |
| 14.69 | 83.95 | 68.25 |  |  | 13.95 | 75.15 | 63.01 |  |  |
| 14.70 | 83.93 | 68.21 |  |  | 13.96 | 75.11 | 62.97 |  |  |
| 14.71 | 83.88 | 68.17 |  |  | 13.97 | 75.06 | 62.94 |  |  |
| 14.72 | 83.84 | 68.13 |  |  | 13.98 | 75.03 | 62.90 |  |  |
| 14.73 | 83.81 | 68.09 |  |  | 13.99 | 74.99 | 62.86 |  |  |
| 14.74 | 83.76 | 68.05 |  |  | 14.00 | 74.94 | 62.82 |  |  |
| 14.75 | 83.72 | 68.01 |  |  | 14.01 | 74.89 | 62.78 |  |  |
| 14.76 | 83.69 | 67.98 |  |  | 14.02 | 74.84 | 62.75 |  |  |
| 14.77 | 83.66 | 67.94 |  |  | 14.03 | 74.79 | 62.71 |  |  |
| 14.78 | 83.62 | 67.90 |  |  | 14.05 | 74.71 | 62.63 |  |  |
| 14.79 | 83.60 | 67.86 |  |  | 14.06 | 74.67 | 62.59 |  |  |
| 14.80 | 83.57 | 67.82 |  |  | 14.07 | 74.63 | 62.56 |  |  |
| 14.82 | 83.52 | 67.75 |  |  | 14.08 | 74.58 | 62.52 |  |  |
| 14.83 | 83.47 | 67.71 |  |  | 14.09 | 74.53 | 62.48 |  |  |
| 14.84 | 83.42 | 67.67 |  |  | 14.10 | 74.46 | 62.44 |  |  |
| 14.86 | 83.36 | 67.59 |  |  | 14.11 | 74.42 | 62.40 |  |  |
| 14.87 | 83.33 | 67.55 |  |  | 14.12 | 74.35 | 62.37 |  |  |
| 14.88 | 83.29 | 67.51 |  |  | 14.13 | 74.30 | 62.33 |  |  |
| 14.89 | 83.26 | 67.48 |  |  | 14.14 | 74.27 | 62.29 |  |  |
| 14.90 | 83.20 | 67.44 |  |  | 14.16 | 74.21 | 62.21 |  |  |
| 14.91 | 83.15 | 67.40 |  |  | 14.17 | 74.17 | 62.17 |  |  |
| 14.92 | 83.12 | 67.36 |  |  | 14.18 | 74.13 | 62.14 |  |  |
| 14.93 | 83.08 | 67.32 |  |  | 14.19 | 74.07 | 62.10 |  |  |
| 14.94 | 83.04 | 67.28 |  |  | 14.20 | 74.02 | 62.06 |  |  |
| 14.95 | 82.99 | 67.25 |  |  | 14.21 | 73.95 | 62.02 |  |  |
| 14.96 | 82.93 | 67.21 |  |  | 14.22 | 73.93 | 61.98 |  |  |
| 14.97 | 82.91 | 67.17 |  |  | 14.23 | 73.91 | 61.95 |  |  |
| 14.98 | 82.89 | 67.13 |  |  | 14.24 | 73.87 | 61.91 |  |  |
| 14.99 | 82.84 | 67.09 |  |  | 14.25 | 73.85 | 61.87 |  |  |
| 15.00 | 82.81 | 67.05 |  |  | 14.26 | 73.79 | 61.83 |  |  |
| 15.01 | 82.78 | 67.01 |  |  | 14.27 | 73.73 | 61.79 |  |  |
| 15.02 | 82.76 | 66.98 |  |  | 14.28 | 73.69 | 61.76 |  |  |
| 15.03 | 82.72 | 66.94 |  |  | 14.29 | 73.66 | 61.72 |  |  |
| 15.04 | 82.68 | 66.90 |  |  | 14.30 | 73.62 | 61.68 |  |  |
| 15.05 | 82.64 | 66.86 |  |  | 14.31 | 73.58 | 61.64 |  |  |
| 15.06 | 82.61 | 66.82 |  |  | 14.32 | 73.52 | 61.60 |  |  |
| 15.07 | 82.57 | 66.78 |  |  | 14.33 | 73.49 | 61.57 |  |  |
| 15.08 | 82.55 | 66.75 |  |  | 14.34 | 73.45 | 61.53 |  |  |
| 15.09 | 82.51 | 66.71 |  |  | 14.35 | 73.40 | 61.49 |  |  |
| 15.10 | 82.47 | 66.67 |  |  | 14.36 | 73.36 | 61.45 |  |  |
| 15.11 | 82.44 | 66.63 |  |  | 14.37 | 73.30 | 61.41 |  |  |
| 15.12 | 82.40 | 66.59 |  |  | 14.38 | 73.26 | 61.38 |  |  |
| 15.13 | 82.38 | 66.55 |  |  | 14.39 | 73.22 | 61.34 |  |  |
| 15.14 | 82.34 | 66.51 |  |  | 14.40 | 73.17 | 61.30 |  |  |
| 15.15 | 82.29 | 66.48 |  |  | 14.42 | 73.07 | 61.22 |  |  |
| 15.16 | 82.26 | 66.44 |  |  | 14.43 | 73.02 | 61.19 |  |  |
| 15.17 | 82.20 | 66.40 |  |  | 14.44 | 72.96 | 61.15 |  |  |
| 15.18 | 82.16 | 66.36 |  |  | 14.45 | 72.91 | 61.11 |  |  |
| 15.19 | 82.11 | 66.32 |  |  | 14.46 | 72.85 | 61.07 |  |  |
| 15.20 | 82.05 | 66.28 |  |  | 14.47 | 72.80 | 61.03 |  |  |
| 15.21 | 82.03 | 66.25 |  |  | 14.48 | 72.76 | 60.99 |  |  |
| 15.22 | 81.98 | 66.21 |  |  | 14.49 | 72.73 | 60.96 |  |  |
| 15.23 | 81.92 | 66.17 |  |  | 14.50 | 72.68 | 60.92 |  |  |
| 15.24 | 81.88 | 66.13 |  |  | 14.51 | 72.64 | 60.88 |  |  |
| 15.25 | 81.83 | 66.09 |  |  | 14.52 | 72.61 | 60.84 |  |  |
| 15.26 | 81.78 | 66.05 |  |  | 14.53 | 72.57 | 60.80 |  |  |
| 15.27 | 81.74 | 66.01 |  |  | 14.55 | 72.46 | 60.73 |  |  |
| 15.28 | 81.68 | 65.98 |  |  | 14.56 | 72.42 | 60.69 |  |  |
| 15.29 | 81.64 | 65.94 |  |  | 14.57 | 72.39 | 60.65 |  |  |
| 15.30 | 81.60 | 65.90 |  |  | 14.58 | 72.34 | 60.61 |  |  |
| 15.31 | 81.55 | 65.86 |  |  | 14.60 | 72.25 | 60.54 |  |  |
| 15.32 | 81.51 | 65.82 |  |  | 14.62 | 72.18 | 60.46 |  |  |
| 15.33 | 81.46 | 65.78 |  |  | 14.63 | 72.14 | 60.42 |  |  |
| 15.34 | 81.45 | 65.75 |  |  | 14.64 | 72.09 | 60.39 |  |  |
| 15.35 | 81.41 | 65.71 |  |  | 14.65 | 72.06 | 60.35 |  |  |
| 15.36 | 81.36 | 65.67 |  |  | 14.66 | 72.01 | 60.31 |  |  |
| 15.37 | 81.33 | 65.63 |  |  | 14.67 | 71.96 | 60.27 |  |  |
| 15.38 | 81.28 | 65.59 |  |  | 14.68 | 71.92 | 60.23 |  |  |
| 15.39 | 81.22 | 65.55 |  |  | 14.69 | 71.87 | 60.20 |  |  |
| 15.40 | 81.18 | 65.51 |  |  | 14.71 | 71.79 | 60.12 |  |  |
| 15.41 | 81.15 | 65.48 |  |  | 14.72 | 71.75 | 60.08 |  |  |
| 15.42 | 81.11 | 65.44 |  |  | 14.73 | 71.71 | 60.04 |  |  |
| 15.44 | 81.00 | 65.36 |  |  | 14.74 | 71.63 | 60.01 |  |  |
| 15.45 | 80.96 | 65.32 |  |  | 14.75 | 71.60 | 59.97 |  |  |
| 15.46 | 80.91 | 65.28 |  |  | 14.76 | 71.54 | 59.93 |  |  |
| 15.47 | 80.86 | 65.24 |  |  | 14.77 | 71.50 | 59.89 |  |  |
| 15.48 | 80.82 | 65.21 |  |  | 14.78 | 71.45 | 59.85 |  |  |
| 15.49 | 80.78 | 65.17 |  |  | 14.79 | 71.41 | 59.82 |  |  |
| 15.50 | 80.72 | 65.13 |  |  | 14.80 | 71.36 | 59.78 |  |  |
| 15.51 | 80.66 | 65.09 |  |  | 14.81 | 71.31 | 59.74 |  |  |
| 15.52 | 80.63 | 65.05 |  |  | 14.82 | 71.28 | 59.70 |  |  |
| 15.54 | 80.52 | 64.98 |  |  | 14.83 | 71.22 | 59.66 |  |  |
| 15.55 | 80.47 | 64.94 |  |  | 14.84 | 71.17 | 59.62 |  |  |
| 15.56 | 80.43 | 64.90 |  |  | 14.85 | 71.12 | 59.59 |  |  |
| 15.57 | 80.38 | 64.86 |  |  | 14.87 | 71.02 | 59.51 |  |  |
| 15.58 | 80.34 | 64.82 |  |  | 14.88 | 70.98 | 59.47 |  |  |
| 15.59 | 80.30 | 64.78 |  |  | 14.89 | 70.91 | 59.43 |  |  |
| 15.60 | 80.24 | 64.74 |  |  | 14.90 | 70.85 | 59.40 |  |  |
| 15.61 | 80.17 | 64.71 |  |  | 14.91 | 70.79 | 59.36 |  |  |
| 15.62 | 80.13 | 64.67 |  |  | 14.92 | 70.74 | 59.32 |  |  |
| 15.63 | 80.08 | 64.63 |  |  | 14.93 | 70.70 | 59.28 |  |  |
| 15.64 | 80.04 | 64.59 |  |  | 14.94 | 70.64 | 59.24 |  |  |
| 15.65 | 80.01 | 64.55 |  |  | 14.95 | 70.58 | 59.21 |  |  |
| 15.66 | 79.95 | 64.51 |  |  | 14.96 | 70.54 | 59.17 |  |  |
| 15.67 | 79.88 | 64.48 |  |  | 14.98 | 70.42 | 59.09 |  |  |
| 15.68 | 79.83 | 64.44 |  |  | 14.99 | 70.37 | 59.05 |  |  |
| 15.70 | 79.76 | 64.36 |  |  | 15.00 | 70.33 | 59.02 |  |  |
| 15.71 | 79.71 | 64.32 |  |  | 15.01 | 70.27 | 58.98 |  |  |
| 15.72 | 79.66 | 64.28 |  |  | 15.02 | 70.21 | 58.94 |  |  |
| 15.73 | 79.59 | 64.24 |  |  | 15.04 | 70.11 | 58.86 |  |  |
| 15.74 | 79.53 | 64.21 |  |  | 15.05 | 70.06 | 58.83 |  |  |
| 15.75 | 79.47 | 64.17 |  |  | 15.06 | 70.03 | 58.79 |  |  |
| 15.76 | 79.43 | 64.13 |  |  | 15.07 | 69.96 | 58.75 |  |  |
| 15.77 | 79.37 | 64.09 |  |  | 15.08 | 69.90 | 58.71 |  |  |
| 15.78 | 79.32 | 64.05 |  |  | 15.09 | 69.86 | 58.67 |  |  |
| 15.79 | 79.28 | 64.01 |  |  | 15.10 | 69.81 | 58.64 |  |  |
| 15.80 | 79.25 | 63.98 |  |  | 15.11 | 69.74 | 58.60 |  |  |
| 15.81 | 79.20 | 63.94 |  |  | 15.12 | 69.69 | 58.56 |  |  |
| 15.82 | 79.16 | 63.90 |  |  | 15.13 | 69.65 | 58.52 |  |  |
| 15.83 | 79.12 | 63.86 |  |  | 15.14 | 69.60 | 58.48 |  |  |
| 15.84 | 79.07 | 63.82 |  |  | 15.15 | 69.56 | 58.44 |  |  |
| 15.85 | 79.02 | 63.78 |  |  | 15.16 | 69.51 | 58.41 |  |  |
| 15.86 | 78.98 | 63.74 |  |  | 15.17 | 69.44 | 58.37 |  |  |
| 15.87 | 78.95 | 63.71 |  |  | 15.19 | 69.34 | 58.29 |  |  |
| 15.88 | 78.89 | 63.67 |  |  | 15.20 | 69.30 | 58.25 |  |  |
| 15.90 | 78.80 | 63.59 |  |  | 15.22 | 69.17 | 58.18 |  |  |
| 15.91 | 78.74 | 63.55 |  |  | 15.23 | 69.15 | 58.14 |  |  |
| 15.92 | 78.69 | 63.51 |  |  | 15.25 | 69.04 | 58.06 |  |  |
| 15.93 | 78.66 | 63.48 |  |  | 15.26 | 68.99 | 58.03 |  |  |
| 15.94 | 78.61 | 63.44 |  |  | 15.27 | 68.92 | 57.99 |  |  |
| 15.95 | 78.56 | 63.40 |  |  | 15.28 | 68.88 | 57.95 |  |  |
| 15.96 | 78.52 | 63.36 |  |  | 15.29 | 68.79 | 57.91 |  |  |
| 15.97 | 78.48 | 63.32 |  |  | 15.30 | 68.76 | 57.87 |  |  |
| 15.98 | 78.43 | 63.28 |  |  | 15.31 | 68.69 | 57.84 |  |  |
| 15.99 | 78.38 | 63.24 |  |  | 15.32 | 68.64 | 57.80 |  |  |
| 16.00 | 78.32 | 63.21 |  |  | 15.33 | 68.58 | 57.76 |  |  |
| 16.01 | 78.28 | 63.17 |  |  | 15.34 | 68.51 | 57.72 |  |  |
| 16.02 | 78.23 | 63.13 |  |  | 15.35 | 68.47 | 57.68 |  |  |
| 16.03 | 78.17 | 63.09 |  |  | 15.36 | 68.38 | 57.65 |  |  |
| 16.04 | 78.12 | 63.05 |  |  | 15.37 | 68.31 | 57.61 |  |  |
| 16.05 | 78.07 | 63.01 |  |  | 15.38 | 68.24 | 57.57 |  |  |
| 16.06 | 78.02 | 62.98 |  |  | 15.39 | 68.17 | 57.53 |  |  |
| 16.07 | 77.97 | 62.94 |  |  | 15.40 | 68.09 | 57.49 |  |  |
| 16.08 | 77.91 | 62.90 |  |  | 15.41 | 68.02 | 57.46 |  |  |
| 16.09 | 77.86 | 62.86 |  |  | 15.42 | 67.92 | 57.42 |  |  |
| 16.10 | 77.81 | 62.82 |  |  | 15.45 | 67.69 | 57.30 |  |  |
| 16.11 | 77.74 | 62.78 |  |  | 15.46 | 67.61 | 57.26 |  |  |
| 16.12 | 77.70 | 62.74 |  |  | 15.47 | 67.55 | 57.23 |  |  |
| 16.13 | 77.67 | 62.71 |  |  | 15.48 | 67.49 | 57.19 |  |  |
| 16.14 | 77.61 | 62.67 |  |  | 15.49 | 67.39 | 57.15 |  |  |
| 16.16 | 77.52 | 62.59 |  |  | 15.50 | 67.33 | 57.11 |  |  |
| 16.17 | 77.47 | 62.55 |  |  | 15.51 | 67.24 | 57.07 |  |  |
| 16.18 | 77.42 | 62.51 |  |  | 15.52 | 67.16 | 57.04 |  |  |
| 16.19 | 77.36 | 62.48 |  |  | 15.53 | 67.10 | 57.00 |  |  |
| 16.20 | 77.32 | 62.44 |  |  | 15.54 | 67.04 | 56.96 |  |  |
| 16.21 | 77.27 | 62.40 |  |  | 15.55 | 66.96 | 56.92 |  |  |
| 16.22 | 77.20 | 62.36 |  |  | 15.56 | 66.89 | 56.88 |  |  |
| 16.23 | 77.13 | 62.32 |  |  | 15.57 | 66.79 | 56.85 |  |  |
| 16.24 | 77.09 | 62.28 |  |  | 15.58 | 66.73 | 56.81 |  |  |
| 16.25 | 77.05 | 62.24 |  |  | 15.59 | 66.67 | 56.77 |  |  |
| 16.26 | 77.00 | 62.21 |  |  | 15.60 | 66.58 | 56.73 |  |  |
| 16.27 | 76.94 | 62.17 |  |  | 15.61 | 66.51 | 56.69 |  |  |
| 16.28 | 76.89 | 62.13 |  |  | 15.62 | 66.44 | 56.66 |  |  |
| 16.29 | 76.83 | 62.09 |  |  | 15.63 | 66.36 | 56.62 |  |  |
| 16.30 | 76.78 | 62.05 |  |  | 15.64 | 66.26 | 56.58 |  |  |
| 16.31 | 76.74 | 62.01 |  |  | 15.65 | 66.20 | 56.54 |  |  |
| 16.32 | 76.71 | 61.98 |  |  | 15.66 | 66.11 | 56.50 |  |  |
| 16.33 | 76.65 | 61.94 |  |  | 15.67 | 66.04 | 56.47 |  |  |
| 16.34 | 76.60 | 61.90 |  |  | 15.68 | 65.96 | 56.43 |  |  |
| 16.35 | 76.56 | 61.86 |  |  | 15.69 | 65.88 | 56.39 |  |  |
| 16.36 | 76.52 | 61.82 |  |  | 15.70 | 65.80 | 56.35 |  |  |
| 16.37 | 76.45 | 61.78 |  |  | 15.71 | 65.74 | 56.31 |  |  |
| 16.38 | 76.40 | 61.74 |  |  | 15.72 | 65.66 | 56.28 |  |  |
| 16.39 | 76.36 | 61.71 |  |  | 15.73 | 65.57 | 56.24 |  |  |
| 16.40 | 76.31 | 61.67 |  |  | 15.74 | 65.50 | 56.20 |  |  |
| 16.41 | 76.26 | 61.63 |  |  | 15.75 | 65.41 | 56.16 |  |  |
| 16.42 | 76.22 | 61.59 |  |  | 15.76 | 65.31 | 56.12 |  |  |
| 16.43 | 76.16 | 61.55 |  |  | 15.77 | 65.26 | 56.09 |  |  |
| 16.44 | 76.11 | 61.51 |  |  | 15.78 | 65.17 | 56.05 |  |  |
| 16.45 | 76.05 | 61.47 |  |  | 15.79 | 65.09 | 56.01 |  |  |
| 16.46 | 76.00 | 61.44 |  |  | 15.80 | 65.00 | 55.97 |  |  |
| 16.47 | 75.95 | 61.40 |  |  | 15.81 | 64.93 | 55.93 |  |  |
| 16.48 | 75.89 | 61.36 |  |  | 15.82 | 64.84 | 55.89 |  |  |
| 16.49 | 75.82 | 61.32 |  |  | 15.83 | 64.75 | 55.86 |  |  |
| 16.50 | 75.77 | 61.28 |  |  | 15.84 | 64.66 | 55.82 |  |  |
| 16.51 | 75.74 | 61.24 |  |  | 15.85 | 64.58 | 55.78 |  |  |
| 16.52 | 75.70 | 61.21 |  |  | 15.86 | 64.50 | 55.74 |  |  |
| 16.53 | 75.65 | 61.17 |  |  | 15.87 | 64.42 | 55.70 |  |  |
| 16.54 | 75.59 | 61.13 |  |  | 15.88 | 64.32 | 55.67 |  |  |
| 16.55 | 75.55 | 61.09 |  |  | 15.89 | 64.21 | 55.63 |  |  |
| 16.56 | 75.51 | 61.05 |  |  | 15.90 | 64.13 | 55.59 |  |  |
| 16.57 | 75.45 | 61.01 |  |  | 15.91 | 64.04 | 55.55 |  |  |
| 16.58 | 75.38 | 60.97 |  |  | 15.92 | 63.96 | 55.51 |  |  |
| 16.59 | 75.35 | 60.94 |  |  | 15.93 | 63.90 | 55.48 |  |  |
| 16.60 | 75.31 | 60.90 |  |  | 15.94 | 63.77 | 55.44 |  |  |
| 16.61 | 75.27 | 60.86 |  |  | 15.95 | 63.69 | 55.40 |  |  |
| 16.62 | 75.22 | 60.82 |  |  | 15.96 | 63.59 | 55.36 |  |  |
| 16.63 | 75.17 | 60.78 |  |  | 15.97 | 63.50 | 55.32 |  |  |
| 16.64 | 75.12 | 60.74 |  |  | 15.98 | 63.39 | 55.29 |  |  |
| 16.65 | 75.06 | 60.71 |  |  | 15.99 | 63.30 | 55.25 |  |  |
| 16.66 | 75.01 | 60.67 |  |  | 16.00 | 63.22 | 55.21 |  |  |
| 16.67 | 74.94 | 60.63 |  |  | 16.01 | 63.12 | 55.17 |  |  |
| 16.68 | 74.87 | 60.59 |  |  | 16.02 | 63.03 | 55.13 |  |  |
| 16.69 | 74.83 | 60.55 |  |  | 16.03 | 62.92 | 55.10 |  |  |
| 16.70 | 74.76 | 60.51 |  |  | 16.04 | 62.84 | 55.06 |  |  |
| 16.72 | 74.62 | 60.44 |  |  | 16.05 | 62.74 | 55.02 |  |  |
| 16.73 | 74.54 | 60.40 |  |  | 16.06 | 62.65 | 54.98 |  |  |
| 16.74 | 74.48 | 60.36 |  |  | 16.07 | 62.57 | 54.94 |  |  |
| 16.75 | 74.41 | 60.32 |  |  | 16.08 | 62.47 | 54.91 |  |  |
| 16.76 | 74.34 | 60.28 |  |  | 16.09 | 62.36 | 54.87 |  |  |
| 16.77 | 74.26 | 60.24 |  |  | 16.11 | 62.17 | 54.79 |  |  |
| 16.78 | 74.20 | 60.21 |  |  | 16.12 | 62.05 | 54.75 |  |  |
| 16.79 | 74.13 | 60.17 |  |  | 16.13 | 61.98 | 54.71 |  |  |
| 16.80 | 74.08 | 60.13 |  |  | 16.14 | 61.89 | 54.68 |  |  |
| 16.81 | 74.01 | 60.09 |  |  | 16.15 | 61.80 | 54.64 |  |  |
| 16.82 | 73.95 | 60.05 |  |  | 16.16 | 61.73 | 54.60 |  |  |
| 16.83 | 73.88 | 60.01 |  |  | 16.17 | 61.65 | 54.56 |  |  |
| 16.84 | 73.82 | 59.97 |  |  | 16.18 | 61.53 | 54.52 |  |  |
| 16.85 | 73.77 | 59.94 |  |  | 16.19 | 61.44 | 54.49 |  |  |
| 16.86 | 73.70 | 59.90 |  |  | 16.20 | 61.37 | 54.45 |  |  |
| 16.87 | 73.62 | 59.86 |  |  | 16.21 | 61.29 | 54.41 |  |  |
| 16.88 | 73.55 | 59.82 |  |  | 16.22 | 61.19 | 54.37 |  |  |
| 16.89 | 73.47 | 59.78 |  |  | 16.23 | 61.11 | 54.33 |  |  |
| 16.90 | 73.40 | 59.74 |  |  | 16.24 | 61.01 | 54.30 |  |  |
| 16.91 | 73.34 | 59.71 |  |  | 16.25 | 60.93 | 54.26 |  |  |
| 16.92 | 73.25 | 59.67 |  |  | 16.26 | 60.83 | 54.22 |  |  |
| 16.93 | 73.17 | 59.63 |  |  | 16.27 | 60.74 | 54.18 |  |  |
| 16.94 | 73.10 | 59.59 |  |  | 16.28 | 60.66 | 54.14 |  |  |
| 16.95 | 73.05 | 59.55 |  |  | 16.29 | 60.56 | 54.11 |  |  |
| 16.96 | 72.99 | 59.51 |  |  | 16.30 | 60.48 | 54.07 |  |  |
| 16.97 | 72.92 | 59.47 |  |  | 16.31 | 60.38 | 54.03 |  |  |
| 16.98 | 72.85 | 59.44 |  |  | 16.32 | 60.31 | 53.99 |  |  |
| 16.99 | 72.77 | 59.40 |  |  | 16.33 | 60.23 | 53.95 |  |  |
| 17.00 | 72.71 | 59.36 |  |  | 16.34 | 60.15 | 53.92 |  |  |
| 17.01 | 72.65 | 59.32 |  |  | 16.35 | 60.04 | 53.88 |  |  |
| 17.02 | 72.57 | 59.28 |  |  | 16.36 | 59.97 | 53.84 |  |  |
| 17.03 | 72.50 | 59.24 |  |  | 16.37 | 59.88 | 53.80 |  |  |
| 17.04 | 72.45 | 59.21 |  |  | 16.38 | 59.80 | 53.76 |  |  |
| 17.05 | 72.38 | 59.17 |  |  | 16.39 | 59.72 | 53.73 |  |  |
| 17.07 | 72.21 | 59.09 |  |  | 16.40 | 59.67 | 53.69 |  |  |
| 17.08 | 72.16 | 59.05 |  |  | 16.41 | 59.62 | 53.65 |  |  |
| 17.09 | 72.08 | 59.01 |  |  | 16.42 | 59.53 | 53.61 |  |  |
| 17.11 | 71.92 | 58.94 |  |  | 16.43 | 59.40 | 53.57 |  |  |
| 17.12 | 71.82 | 58.90 |  |  | 16.44 | 59.28 | 53.53 |  |  |
| 17.13 | 71.72 | 58.86 |  |  | 16.45 | 59.21 | 53.50 |  |  |
| 17.14 | 71.65 | 58.82 |  |  | 16.46 | 59.12 | 53.46 |  |  |
| 17.15 | 71.57 | 58.78 |  |  | 16.47 | 59.01 | 53.42 |  |  |
| 17.16 | 71.52 | 58.74 |  |  | 16.48 | 58.94 | 53.38 |  |  |
| 17.17 | 71.45 | 58.71 |  |  | 16.49 | 58.83 | 53.34 |  |  |
| 17.18 | 71.38 | 58.67 |  |  | 16.50 | 58.75 | 53.31 |  |  |
| 17.19 | 71.32 | 58.63 |  |  | 16.51 | 58.66 | 53.27 |  |  |
| 17.20 | 71.23 | 58.59 |  |  | 16.52 | 58.56 | 53.23 |  |  |
| 17.21 | 71.16 | 58.55 |  |  | 16.53 | 58.48 | 53.19 |  |  |
| 17.22 | 71.10 | 58.51 |  |  | 16.54 | 58.38 | 53.15 |  |  |
| 17.23 | 71.01 | 58.47 |  |  | 16.55 | 58.29 | 53.12 |  |  |
| 17.24 | 70.96 | 58.44 |  |  | 16.56 | 58.22 | 53.08 |  |  |
| 17.25 | 70.87 | 58.40 |  |  | 16.57 | 58.12 | 53.04 |  |  |
| 17.26 | 70.81 | 58.36 |  |  | 16.58 | 58.02 | 53.00 |  |  |
| 17.27 | 70.72 | 58.32 |  |  | 16.59 | 57.92 | 52.96 |  |  |
| 17.28 | 70.64 | 58.28 |  |  | 16.60 | 57.83 | 52.93 |  |  |
| 17.29 | 70.55 | 58.24 |  |  | 16.61 | 57.75 | 52.89 |  |  |
| 17.30 | 70.46 | 58.20 |  |  | 16.62 | 57.64 | 52.85 |  |  |
| 17.31 | 70.39 | 58.17 |  |  | 16.63 | 57.53 | 52.81 |  |  |
| 17.32 | 70.29 | 58.13 |  |  | 16.64 | 57.41 | 52.77 |  |  |
| 17.33 | 70.20 | 58.09 |  |  | 16.65 | 57.32 | 52.74 |  |  |
| 17.34 | 70.14 | 58.05 |  |  | 16.67 | 57.15 | 52.66 |  |  |
| 17.35 | 70.05 | 58.01 |  |  | 16.68 | 57.06 | 52.62 |  |  |
| 17.36 | 69.97 | 57.97 |  |  | 16.69 | 56.96 | 52.58 |  |  |
| 17.37 | 69.88 | 57.94 |  |  | 16.70 | 56.87 | 52.55 |  |  |
| 17.38 | 69.82 | 57.90 |  |  | 16.73 | 56.54 | 52.43 |  |  |
| 17.39 | 69.73 | 57.86 |  |  | 16.74 | 56.44 | 52.39 |  |  |
| 17.40 | 69.66 | 57.82 |  |  | 16.75 | 56.34 | 52.36 |  |  |
| 17.41 | 69.60 | 57.78 |  |  | 16.76 | 56.26 | 52.32 |  |  |
| 17.42 | 69.53 | 57.74 |  |  | 16.77 | 56.18 | 52.28 |  |  |
| 17.43 | 69.45 | 57.70 |  |  | 16.78 | 56.07 | 52.24 |  |  |
| 17.44 | 69.38 | 57.67 |  |  | 16.79 | 55.99 | 52.20 |  |  |
| 17.45 | 69.31 | 57.63 |  |  | 16.80 | 55.90 | 52.16 |  |  |
| 17.46 | 69.23 | 57.59 |  |  | 16.81 | 55.78 | 52.13 |  |  |
| 17.47 | 69.12 | 57.55 |  |  | 16.82 | 55.71 | 52.09 |  |  |
| 17.48 | 69.02 | 57.51 |  |  | 16.83 | 55.58 | 52.05 |  |  |
| 17.49 | 68.94 | 57.47 |  |  | 16.84 | 55.49 | 52.01 |  |  |
| 17.50 | 68.87 | 57.44 |  |  | 16.85 | 55.42 | 51.97 |  |  |
| 17.51 | 68.78 | 57.40 |  |  | 16.86 | 55.30 | 51.94 |  |  |
| 17.52 | 68.67 | 57.36 |  |  | 16.87 | 55.22 | 51.90 |  |  |
| 17.53 | 68.57 | 57.32 |  |  | 16.88 | 55.10 | 51.86 |  |  |
| 17.54 | 68.48 | 57.28 |  |  | 16.89 | 55.03 | 51.82 |  |  |
| 17.55 | 68.40 | 57.24 |  |  | 16.90 | 54.94 | 51.78 |  |  |
| 17.56 | 68.32 | 57.20 |  |  | 16.91 | 54.85 | 51.75 |  |  |
| 17.57 | 68.23 | 57.17 |  |  | 16.92 | 54.77 | 51.71 |  |  |
| 17.58 | 68.16 | 57.13 |  |  | 16.93 | 54.69 | 51.67 |  |  |
| 17.59 | 68.06 | 57.09 |  |  | 16.94 | 54.56 | 51.63 |  |  |
| 17.60 | 67.98 | 57.05 |  |  | 16.95 | 54.45 | 51.59 |  |  |
| 17.61 | 67.89 | 57.01 |  |  | 16.96 | 54.35 | 51.56 |  |  |
| 17.62 | 67.77 | 56.97 |  |  | 16.97 | 54.24 | 51.52 |  |  |
| 17.63 | 67.65 | 56.94 |  |  | 16.98 | 54.17 | 51.48 |  |  |
| 17.64 | 67.53 | 56.90 |  |  | 16.99 | 54.09 | 51.44 |  |  |
| 17.65 | 67.45 | 56.86 |  |  | 17.00 | 53.99 | 51.40 |  |  |
| 17.66 | 67.35 | 56.82 |  |  | 17.01 | 53.88 | 51.37 |  |  |
| 17.67 | 67.23 | 56.78 |  |  | 17.02 | 53.78 | 51.33 |  |  |
| 17.68 | 67.14 | 56.74 |  |  | 17.03 | 53.68 | 51.29 |  |  |
| 17.69 | 67.04 | 56.70 |  |  | 17.04 | 53.57 | 51.25 |  |  |
| 17.70 | 66.98 | 56.67 |  |  | 17.05 | 53.49 | 51.21 |  |  |
| 17.71 | 66.87 | 56.63 |  |  | 17.06 | 53.40 | 51.18 |  |  |
| 17.73 | 66.69 | 56.55 |  |  | 17.07 | 53.29 | 51.14 |  |  |
| 17.74 | 66.57 | 56.51 |  |  | 17.08 | 53.17 | 51.10 |  |  |
| 17.75 | 66.47 | 56.47 |  |  | 17.09 | 53.05 | 51.06 |  |  |
| 17.76 | 66.38 | 56.44 |  |  | 17.10 | 52.96 | 51.02 |  |  |
| 17.77 | 66.26 | 56.40 |  |  | 17.11 | 52.86 | 50.98 |  |  |
| 17.78 | 66.18 | 56.36 |  |  | 17.12 | 52.75 | 50.95 |  |  |
| 17.79 | 66.08 | 56.32 |  |  | 17.13 | 52.63 | 50.91 |  |  |
| 17.80 | 65.96 | 56.28 |  |  | 17.14 | 52.55 | 50.87 |  |  |
| 17.81 | 65.86 | 56.24 |  |  | 17.15 | 52.46 | 50.83 |  |  |
| 17.82 | 65.76 | 56.20 |  |  | 17.16 | 52.35 | 50.79 |  |  |
| 17.83 | 65.66 | 56.17 |  |  | 17.17 | 52.23 | 50.76 |  |  |
| 17.84 | 65.55 | 56.13 |  |  | 17.18 | 52.12 | 50.72 |  |  |
| 17.86 | 65.31 | 56.05 |  |  | 17.19 | 52.03 | 50.68 |  |  |
| 17.87 | 65.20 | 56.01 |  |  | 17.20 | 51.91 | 50.64 |  |  |
| 17.88 | 65.10 | 55.97 |  |  | 17.21 | 51.81 | 50.60 |  |  |
| 17.89 | 64.97 | 55.94 |  |  | 17.22 | 51.71 | 50.57 |  |  |
| 17.90 | 64.87 | 55.90 |  |  | 17.23 | 51.59 | 50.53 |  |  |
| 17.91 | 64.79 | 55.86 |  |  | 17.24 | 51.50 | 50.49 |  |  |
| 17.92 | 64.68 | 55.82 |  |  | 17.26 | 51.30 | 50.41 |  |  |
| 17.93 | 64.59 | 55.78 |  |  | 17.27 | 51.21 | 50.38 |  |  |
| 17.94 | 64.48 | 55.74 |  |  | 17.28 | 51.12 | 50.34 |  |  |
| 17.95 | 64.36 | 55.70 |  |  | 17.29 | 51.03 | 50.30 |  |  |
| 17.96 | 64.26 | 55.67 |  |  | 17.30 | 50.94 | 50.26 |  |  |
| 17.97 | 64.14 | 55.63 |  |  | 17.31 | 50.87 | 50.22 |  |  |
| 17.98 | 64.02 | 55.59 |  |  | 17.32 | 50.75 | 50.19 |  |  |
| 17.99 | 63.92 | 55.55 |  |  | 17.33 | 50.67 | 50.15 |  |  |
| 18.00 | 63.81 | 55.51 |  |  | 17.34 | 50.58 | 50.11 |  |  |
| 18.01 | 63.74 | 55.47 |  |  | 17.35 | 50.49 | 50.07 |  |  |
| 18.02 | 63.65 | 55.44 |  |  | 17.36 | 50.39 | 50.03 |  |  |
| 18.03 | 63.55 | 55.40 |  |  | 17.37 | 50.29 | 50.00 |  |  |
| 18.04 | 63.44 | 55.36 |  |  | 17.38 | 50.19 | 49.96 |  |  |
| 18.05 | 63.35 | 55.32 |  |  | 17.39 | 50.11 | 49.92 |  |  |
| 18.06 | 63.25 | 55.28 |  |  | 17.40 | 49.99 | 49.88 |  |  |
| 18.07 | 63.15 | 55.24 |  |  | 17.41 | 49.90 | 49.84 |  |  |
| 18.08 | 63.05 | 55.20 |  |  | 17.42 | 49.79 | 49.80 |  |  |
| 18.09 | 62.95 | 55.17 |  |  | 17.43 | 49.71 | 49.77 |  |  |
| 18.10 | 62.82 | 55.13 |  |  | 17.44 | 49.61 | 49.73 |  |  |
| 18.11 | 62.73 | 55.09 |  |  | 17.45 | 49.53 | 49.69 |  |  |
| 18.12 | 62.63 | 55.05 |  |  | 17.46 | 49.44 | 49.65 |  |  |
| 18.13 | 62.56 | 55.01 |  |  | 17.47 | 49.36 | 49.61 |  |  |
| 18.14 | 62.46 | 54.97 |  |  | 17.48 | 49.28 | 49.58 |  |  |
| 18.15 | 62.35 | 54.93 |  |  | 17.49 | 49.20 | 49.54 |  |  |
| 18.16 | 62.27 | 54.90 |  |  | 17.50 | 49.12 | 49.50 |  |  |
| 18.17 | 62.18 | 54.86 |  |  | 17.51 | 49.02 | 49.46 |  |  |
| 18.18 | 62.08 | 54.82 |  |  | 17.52 | 48.93 | 49.42 |  |  |
| 18.19 | 61.95 | 54.78 |  |  | 17.53 | 48.84 | 49.39 |  |  |
| 18.20 | 61.84 | 54.74 |  |  | 17.54 | 48.74 | 49.35 |  |  |
| 18.21 | 61.76 | 54.70 |  |  | 17.55 | 48.65 | 49.31 |  |  |
| 18.22 | 61.67 | 54.67 |  |  | 17.56 | 48.53 | 49.27 |  |  |
| 18.23 | 61.55 | 54.63 |  |  | 17.57 | 48.45 | 49.23 |  |  |
| 18.24 | 61.43 | 54.59 |  |  | 17.58 | 48.38 | 49.20 |  |  |
| 18.25 | 61.32 | 54.55 |  |  | 17.59 | 48.28 | 49.16 |  |  |
| 18.26 | 61.24 | 54.51 |  |  | 17.60 | 48.19 | 49.12 |  |  |
| 18.27 | 61.15 | 54.47 |  |  | 17.61 | 48.11 | 49.08 |  |  |
| 18.28 | 61.04 | 54.43 |  |  | 17.62 | 48.02 | 49.04 |  |  |
| 18.29 | 60.96 | 54.40 |  |  | 17.63 | 47.91 | 49.01 |  |  |
| 18.30 | 60.87 | 54.36 |  |  | 17.64 | 47.80 | 48.97 |  |  |
| 18.31 | 60.77 | 54.32 |  |  | 17.65 | 47.72 | 48.93 |  |  |
| 18.32 | 60.70 | 54.28 |  |  | 17.66 | 47.62 | 48.89 |  |  |
| 18.33 | 60.62 | 54.24 |  |  | 17.68 | 47.45 | 48.82 |  |  |
| 18.34 | 60.52 | 54.20 |  |  | 17.69 | 47.35 | 48.78 |  |  |
| 18.35 | 60.45 | 54.17 |  |  | 17.70 | 47.25 | 48.74 |  |  |
| 18.36 | 60.32 | 54.13 |  |  | 17.71 | 47.15 | 48.70 |  |  |
| 18.37 | 60.20 | 54.09 |  |  | 17.72 | 47.05 | 48.66 |  |  |
| 18.39 | 60.00 | 54.01 |  |  | 17.73 | 46.96 | 48.62 |  |  |
| 18.40 | 59.89 | 53.97 |  |  | 17.74 | 46.87 | 48.59 |  |  |
| 18.41 | 59.79 | 53.93 |  |  | 17.75 | 46.76 | 48.55 |  |  |
| 18.42 | 59.70 | 53.90 |  |  | 17.76 | 46.68 | 48.51 |  |  |
| 18.44 | 59.48 | 53.82 |  |  | 17.77 | 46.60 | 48.47 |  |  |
| 18.45 | 59.37 | 53.78 |  |  | 17.78 | 46.52 | 48.43 |  |  |
| 18.46 | 59.28 | 53.74 |  |  | 17.79 | 46.42 | 48.40 |  |  |
| 18.47 | 59.14 | 53.70 |  |  | 17.80 | 46.32 | 48.36 |  |  |
| 18.48 | 59.02 | 53.67 |  |  | 17.81 | 46.21 | 48.32 |  |  |
| 18.49 | 58.93 | 53.63 |  |  | 17.82 | 46.11 | 48.28 |  |  |
| 18.50 | 58.82 | 53.59 |  |  | 17.83 | 46.02 | 48.24 |  |  |
| 18.51 | 58.69 | 53.55 |  |  | 17.84 | 45.92 | 48.21 |  |  |
| 18.52 | 58.61 | 53.51 |  |  | 17.85 | 45.82 | 48.17 |  |  |
| 18.53 | 58.48 | 53.47 |  |  | 17.86 | 45.74 | 48.13 |  |  |
| 18.54 | 58.38 | 53.43 |  |  | 17.87 | 45.63 | 48.09 |  |  |
| 18.55 | 58.26 | 53.40 |  |  | 17.88 | 45.55 | 48.05 |  |  |
| 18.56 | 58.12 | 53.36 |  |  | 17.89 | 45.45 | 48.02 |  |  |
| 18.57 | 58.02 | 53.32 |  |  | 17.90 | 45.36 | 47.98 |  |  |
| 18.58 | 57.91 | 53.28 |  |  | 17.91 | 45.25 | 47.94 |  |  |
| 18.59 | 57.81 | 53.24 |  |  | 17.92 | 45.14 | 47.90 |  |  |
| 18.60 | 57.70 | 53.20 |  |  | 17.93 | 45.04 | 47.86 |  |  |
| 18.61 | 57.60 | 53.17 |  |  | 17.94 | 44.95 | 47.83 |  |  |
| 18.62 | 57.47 | 53.13 |  |  | 17.95 | 44.87 | 47.79 |  |  |
| 18.63 | 57.36 | 53.09 |  |  | 17.96 | 44.78 | 47.75 |  |  |
| 18.64 | 57.27 | 53.05 |  |  | 17.97 | 44.67 | 47.71 |  |  |
| 18.66 | 57.04 | 52.97 |  |  | 17.98 | 44.57 | 47.67 |  |  |
| 18.67 | 56.94 | 52.93 |  |  | 17.99 | 44.44 | 47.64 |  |  |
| 18.68 | 56.83 | 52.90 |  |  | 18.00 | 44.35 | 47.60 |  |  |
| 18.69 | 56.74 | 52.86 |  |  | 18.01 | 44.23 | 47.56 |  |  |
| 18.70 | 56.61 | 52.82 |  |  | 18.02 | 44.12 | 47.52 |  |  |
| 18.71 | 56.49 | 52.78 |  |  | 18.03 | 44.02 | 47.48 |  |  |
| 18.72 | 56.37 | 52.74 |  |  | 18.04 | 43.93 | 47.45 |  |  |
| 18.73 | 56.25 | 52.70 |  |  | 18.05 | 43.81 | 47.41 |  |  |
| 18.74 | 56.14 | 52.67 |  |  | 18.06 | 43.73 | 47.37 |  |  |
| 18.75 | 56.00 | 52.63 |  |  | 18.07 | 43.60 | 47.33 |  |  |
| 18.76 | 55.88 | 52.59 |  |  | 18.08 | 43.49 | 47.29 |  |  |
| 18.77 | 55.75 | 52.55 |  |  | 18.09 | 43.40 | 47.25 |  |  |
| 18.78 | 55.67 | 52.51 |  |  | 18.10 | 43.30 | 47.22 |  |  |
| 18.79 | 55.59 | 52.47 |  |  | 18.11 | 43.20 | 47.18 |  |  |
| 18.80 | 55.48 | 52.43 |  |  | 18.12 | 43.11 | 47.14 |  |  |
| 18.81 | 55.36 | 52.40 |  |  | 18.13 | 43.00 | 47.10 |  |  |
| 18.82 | 55.22 | 52.36 |  |  | 18.14 | 42.93 | 47.06 |  |  |
| 18.83 | 55.08 | 52.32 |  |  | 18.15 | 42.84 | 47.03 |  |  |
| 18.84 | 54.96 | 52.28 |  |  | 18.16 | 42.75 | 46.99 |  |  |
| 18.85 | 54.86 | 52.24 |  |  | 18.17 | 42.65 | 46.95 |  |  |
| 18.86 | 54.73 | 52.20 |  |  | 18.18 | 42.56 | 46.91 |  |  |
| 18.87 | 54.63 | 52.17 |  |  | 18.19 | 42.47 | 46.87 |  |  |
| 18.88 | 54.48 | 52.13 |  |  | 18.20 | 42.39 | 46.84 |  |  |
| 18.89 | 54.37 | 52.09 |  |  | 18.21 | 42.28 | 46.80 |  |  |
| 18.90 | 54.27 | 52.05 |  |  | 18.22 | 42.15 | 46.76 |  |  |
| 18.91 | 54.15 | 52.01 |  |  | 18.23 | 42.09 | 46.72 |  |  |
| 18.92 | 54.02 | 51.97 |  |  | 18.24 | 41.97 | 46.68 |  |  |
| 18.93 | 53.92 | 51.93 |  |  | 18.25 | 41.85 | 46.65 |  |  |
| 18.94 | 53.80 | 51.90 |  |  | 18.26 | 41.75 | 46.61 |  |  |
| 18.95 | 53.66 | 51.86 |  |  | 18.27 | 41.64 | 46.57 |  |  |
| 18.96 | 53.56 | 51.82 |  |  | 18.28 | 41.50 | 46.53 |  |  |
| 18.97 | 53.45 | 51.78 |  |  | 18.29 | 41.38 | 46.49 |  |  |
| 18.98 | 53.29 | 51.74 |  |  | 18.30 | 41.27 | 46.46 |  |  |
| 18.99 | 53.15 | 51.70 |  |  | 18.31 | 41.15 | 46.42 |  |  |
| 19.00 | 53.03 | 51.66 |  |  | 18.32 | 41.04 | 46.38 |  |  |
| 19.01 | 52.92 | 51.63 |  |  | 18.33 | 40.96 | 46.34 |  |  |
| 19.02 | 52.80 | 51.59 |  |  | 18.34 | 40.87 | 46.30 |  |  |
| 19.03 | 52.69 | 51.55 |  |  | 18.35 | 40.79 | 46.27 |  |  |
| 19.04 | 52.56 | 51.51 |  |  | 18.36 | 40.68 | 46.23 |  |  |
| 19.05 | 52.45 | 51.47 |  |  | 18.37 | 40.58 | 46.19 |  |  |
| 19.07 | 52.18 | 51.40 |  |  | 18.38 | 40.49 | 46.15 |  |  |
| 19.08 | 52.06 | 51.36 |  |  | 18.39 | 40.37 | 46.11 |  |  |
| 19.09 | 51.96 | 51.32 |  |  | 18.40 | 40.26 | 46.07 |  |  |
| 19.11 | 51.71 | 51.24 |  |  | 18.41 | 40.16 | 46.04 |  |  |
| 19.12 | 51.58 | 51.20 |  |  | 18.42 | 40.05 | 46.00 |  |  |
| 19.13 | 51.44 | 51.16 |  |  | 18.43 | 39.94 | 45.96 |  |  |
| 19.14 | 51.33 | 51.13 |  |  | 18.44 | 39.83 | 45.92 |  |  |
| 19.15 | 51.18 | 51.09 |  |  | 18.45 | 39.72 | 45.88 |  |  |
| 19.16 | 51.06 | 51.05 |  |  | 18.46 | 39.60 | 45.85 |  |  |
| 19.17 | 50.98 | 51.01 |  |  | 18.47 | 39.48 | 45.81 |  |  |
| 19.18 | 50.88 | 50.97 |  |  | 18.48 | 39.40 | 45.77 |  |  |
| 19.19 | 50.80 | 50.93 |  |  | 18.49 | 39.31 | 45.73 |  |  |
| 19.20 | 50.69 | 50.90 |  |  | 18.50 | 39.22 | 45.69 |  |  |
| 19.21 | 50.58 | 50.86 |  |  | 18.51 | 39.11 | 45.66 |  |  |
| 19.22 | 50.49 | 50.82 |  |  | 18.52 | 39.01 | 45.62 |  |  |
| 19.23 | 50.38 | 50.78 |  |  | 18.53 | 38.92 | 45.58 |  |  |
| 19.24 | 50.28 | 50.74 |  |  | 18.54 | 38.81 | 45.54 |  |  |
| 19.25 | 50.17 | 50.70 |  |  | 18.55 | 38.69 | 45.50 |  |  |
| 19.26 | 50.07 | 50.66 |  |  | 18.56 | 38.61 | 45.47 |  |  |
| 19.27 | 49.94 | 50.63 |  |  | 18.57 | 38.54 | 45.43 |  |  |
| 19.28 | 49.84 | 50.59 |  |  | 18.58 | 38.43 | 45.39 |  |  |
| 19.29 | 49.74 | 50.55 |  |  | 18.59 | 38.35 | 45.35 |  |  |
| 19.30 | 49.65 | 50.51 |  |  | 18.60 | 38.26 | 45.31 |  |  |
| 19.31 | 49.54 | 50.47 |  |  | 18.61 | 38.17 | 45.28 |  |  |
| 19.32 | 49.43 | 50.43 |  |  | 18.62 | 38.05 | 45.24 |  |  |
| 19.33 | 49.35 | 50.40 |  |  | 18.63 | 37.96 | 45.20 |  |  |
| 19.34 | 49.22 | 50.36 |  |  | 18.64 | 37.88 | 45.16 |  |  |
| 19.35 | 49.16 | 50.32 |  |  | 18.65 | 37.79 | 45.12 |  |  |
| 19.36 | 49.02 | 50.28 |  |  | 18.67 | 37.62 | 45.05 |  |  |
| 19.37 | 48.92 | 50.24 |  |  | 18.68 | 37.52 | 45.01 |  |  |
| 19.38 | 48.82 | 50.20 |  |  | 18.69 | 37.45 | 44.97 |  |  |
| 19.39 | 48.73 | 50.16 |  |  | 18.70 | 37.34 | 44.93 |  |  |
| 19.40 | 48.66 | 50.13 |  |  | 18.72 | 37.13 | 44.86 |  |  |
| 19.41 | 48.56 | 50.09 |  |  | 18.74 | 36.91 | 44.78 |  |  |
| 19.42 | 48.42 | 50.05 |  |  | 18.75 | 36.80 | 44.74 |  |  |
| 19.43 | 48.33 | 50.01 |  |  | 18.76 | 36.67 | 44.70 |  |  |
| 19.44 | 48.24 | 49.97 |  |  | 18.77 | 36.58 | 44.67 |  |  |
| 19.45 | 48.14 | 49.93 |  |  | 18.78 | 36.49 | 44.63 |  |  |
| 19.46 | 48.05 | 49.90 |  |  | 18.79 | 36.38 | 44.59 |  |  |
| 19.48 | 47.85 | 49.82 |  |  | 18.80 | 36.28 | 44.55 |  |  |
| 19.49 | 47.74 | 49.78 |  |  | 18.81 | 36.17 | 44.51 |  |  |
| 19.50 | 47.65 | 49.74 |  |  | 18.82 | 36.09 | 44.48 |  |  |
| 19.51 | 47.56 | 49.70 |  |  | 18.83 | 35.98 | 44.44 |  |  |
| 19.52 | 47.47 | 49.66 |  |  | 18.84 | 35.86 | 44.40 |  |  |
| 19.53 | 47.41 | 49.63 |  |  | 18.85 | 35.72 | 44.36 |  |  |
| 19.54 | 47.31 | 49.59 |  |  | 18.86 | 35.61 | 44.32 |  |  |
| 19.55 | 47.18 | 49.55 |  |  | 18.87 | 35.54 | 44.29 |  |  |
| 19.56 | 47.08 | 49.51 |  |  | 18.88 | 35.44 | 44.25 |  |  |
| 19.57 | 46.97 | 49.47 |  |  | 18.89 | 35.36 | 44.21 |  |  |
| 19.58 | 46.89 | 49.43 |  |  | 18.90 | 35.26 | 44.17 |  |  |
| 19.59 | 46.78 | 49.40 |  |  | 18.91 | 35.13 | 44.13 |  |  |
| 19.60 | 46.67 | 49.36 |  |  | 18.92 | 35.05 | 44.10 |  |  |
| 19.61 | 46.54 | 49.32 |  |  | 18.93 | 34.96 | 44.06 |  |  |
| 19.62 | 46.44 | 49.28 |  |  | 18.94 | 34.86 | 44.02 |  |  |
| 19.63 | 46.33 | 49.24 |  |  | 18.95 | 34.76 | 43.98 |  |  |
| 19.65 | 46.10 | 49.16 |  |  | 18.96 | 34.66 | 43.94 |  |  |
| 19.66 | 46.01 | 49.13 |  |  | 18.97 | 34.56 | 43.91 |  |  |
| 19.67 | 45.92 | 49.09 |  |  | 18.98 | 34.44 | 43.87 |  |  |
| 19.68 | 45.81 | 49.05 |  |  | 18.99 | 34.35 | 43.83 |  |  |
| 19.69 | 45.72 | 49.01 |  |  | 19.00 | 34.24 | 43.79 |  |  |
| 19.70 | 45.61 | 48.97 |  |  | 19.01 | 34.17 | 43.75 |  |  |
| 19.71 | 45.53 | 48.93 |  |  | 19.02 | 34.07 | 43.72 |  |  |
| 19.73 | 45.39 | 48.86 |  |  | 19.03 | 33.96 | 43.68 |  |  |
| 19.74 | 45.29 | 48.82 |  |  | 19.04 | 33.85 | 43.64 |  |  |
| 19.75 | 45.19 | 48.78 |  |  | 19.05 | 33.75 | 43.60 |  |  |
| 19.76 | 45.08 | 48.74 |  |  | 19.07 | 33.56 | 43.52 |  |  |
| 19.77 | 45.00 | 48.70 |  |  | 19.08 | 33.48 | 43.49 |  |  |
| 19.78 | 44.87 | 48.66 |  |  | 19.09 | 33.37 | 43.45 |  |  |
| 19.79 | 44.80 | 48.63 |  |  | 19.10 | 33.26 | 43.41 |  |  |
| 19.80 | 44.70 | 48.59 |  |  | 19.11 | 33.16 | 43.37 |  |  |
| 19.81 | 44.60 | 48.55 |  |  | 19.12 | 33.07 | 43.33 |  |  |
| 19.82 | 44.50 | 48.51 |  |  | 19.13 | 32.98 | 43.30 |  |  |
| 19.83 | 44.41 | 48.47 |  |  | 19.14 | 32.88 | 43.26 |  |  |
| 19.84 | 44.32 | 48.43 |  |  | 19.16 | 32.69 | 43.18 |  |  |
| 19.85 | 44.24 | 48.40 |  |  | 19.17 | 32.60 | 43.14 |  |  |
| 19.86 | 44.15 | 48.36 |  |  | 19.18 | 32.52 | 43.11 |  |  |
| 19.87 | 44.04 | 48.32 |  |  | 19.19 | 32.41 | 43.07 |  |  |
| 19.88 | 43.94 | 48.28 |  |  | 19.20 | 32.31 | 43.03 |  |  |
| 19.89 | 43.85 | 48.24 |  |  | 19.22 | 32.09 | 42.95 |  |  |
| 19.91 | 43.66 | 48.16 |  |  | 19.23 | 31.98 | 42.92 |  |  |
| 19.92 | 43.57 | 48.13 |  |  | 19.24 | 31.90 | 42.88 |  |  |
| 19.93 | 43.47 | 48.09 |  |  | 19.25 | 31.79 | 42.84 |  |  |
| 19.94 | 43.39 | 48.05 |  |  | 19.26 | 31.68 | 42.80 |  |  |
| 19.96 | 43.19 | 47.97 |  |  | 19.27 | 31.60 | 42.76 |  |  |
| 19.97 | 43.10 | 47.93 |  |  | 19.28 | 31.50 | 42.73 |  |  |
| 19.98 | 43.02 | 47.89 |  |  | 19.29 | 31.39 | 42.69 |  |  |
| 19.99 | 42.90 | 47.86 |  |  | 19.30 | 31.32 | 42.65 |  |  |
| 20.00 | 42.80 | 47.82 |  |  | 19.31 | 31.22 | 42.61 |  |  |
| 20.01 | 42.70 | 47.78 |  |  | 19.32 | 31.12 | 42.57 |  |  |
| 20.02 | 42.63 | 47.74 |  |  | 19.33 | 31.02 | 42.54 |  |  |
| 20.03 | 42.53 | 47.70 |  |  | 19.34 | 30.92 | 42.50 |  |  |
| 20.04 | 42.44 | 47.66 |  |  | 19.35 | 30.80 | 42.46 |  |  |
| 20.05 | 42.35 | 47.63 |  |  | 19.36 | 30.68 | 42.42 |  |  |
| 20.06 | 42.27 | 47.59 |  |  | 19.37 | 30.59 | 42.38 |  |  |
| 20.07 | 42.14 | 47.55 |  |  | 19.38 | 30.51 | 42.34 |  |  |
| 20.08 | 42.02 | 47.51 |  |  | 19.39 | 30.42 | 42.31 |  |  |
| 20.09 | 41.91 | 47.47 |  |  | 19.40 | 30.32 | 42.27 |  |  |
| 20.10 | 41.82 | 47.43 |  |  | 19.41 | 30.23 | 42.23 |  |  |
| 20.11 | 41.73 | 47.39 |  |  | 19.42 | 30.16 | 42.19 |  |  |
| 20.12 | 41.65 | 47.36 |  |  | 19.43 | 30.04 | 42.15 |  |  |
| 20.13 | 41.56 | 47.32 |  |  | 19.44 | 29.97 | 42.12 |  |  |
| 20.14 | 41.47 | 47.28 |  |  | 19.45 | 29.88 | 42.08 |  |  |
| 20.15 | 41.40 | 47.24 |  |  | 19.46 | 29.79 | 42.04 |  |  |
| 20.16 | 41.30 | 47.20 |  |  | 19.47 | 29.68 | 42.00 |  |  |
| 20.17 | 41.20 | 47.16 |  |  | 19.48 | 29.59 | 41.96 |  |  |
| 20.18 | 41.11 | 47.13 |  |  | 19.49 | 29.51 | 41.93 |  |  |
| 20.19 | 41.00 | 47.09 |  |  | 19.50 | 29.43 | 41.89 |  |  |
| 20.20 | 40.90 | 47.05 |  |  | 19.51 | 29.35 | 41.85 |  |  |
| 20.21 | 40.79 | 47.01 |  |  | 19.52 | 29.25 | 41.81 |  |  |
| 20.22 | 40.71 | 46.97 |  |  | 19.53 | 29.16 | 41.77 |  |  |
| 20.23 | 40.63 | 46.93 |  |  | 19.54 | 29.05 | 41.74 |  |  |
| 20.24 | 40.50 | 46.89 |  |  | 19.55 | 28.92 | 41.70 |  |  |
| 20.25 | 40.42 | 46.86 |  |  | 19.56 | 28.84 | 41.66 |  |  |
| 20.26 | 40.31 | 46.82 |  |  | 19.57 | 28.74 | 41.62 |  |  |
| 20.27 | 40.21 | 46.78 |  |  | 19.58 | 28.66 | 41.58 |  |  |
| 20.28 | 40.15 | 46.74 |  |  | 19.59 | 28.58 | 41.55 |  |  |
| 20.29 | 40.04 | 46.70 |  |  | 19.60 | 28.50 | 41.51 |  |  |
| 20.30 | 39.95 | 46.66 |  |  | 19.61 | 28.39 | 41.47 |  |  |
| 20.31 | 39.86 | 46.63 |  |  | 19.62 | 28.31 | 41.43 |  |  |
| 20.32 | 39.78 | 46.59 |  |  | 19.63 | 28.20 | 41.39 |  |  |
| 20.33 | 39.69 | 46.55 |  |  | 19.64 | 28.09 | 41.36 |  |  |
| 20.34 | 39.57 | 46.51 |  |  | 19.65 | 28.00 | 41.32 |  |  |
| 20.35 | 39.48 | 46.47 |  |  | 19.66 | 27.92 | 41.28 |  |  |
| 20.36 | 39.37 | 46.43 |  |  | 19.67 | 27.81 | 41.24 |  |  |
| 20.37 | 39.30 | 46.39 |  |  | 19.68 | 27.69 | 41.20 |  |  |
| 20.38 | 39.21 | 46.36 |  |  | 19.70 | 27.56 | 41.13 |  |  |
| 20.39 | 39.10 | 46.32 |  |  | 19.71 | 27.48 | 41.09 |  |  |
| 20.40 | 38.95 | 46.28 |  |  | 19.72 | 27.41 | 41.05 |  |  |
| 20.41 | 38.85 | 46.24 |  |  | 19.73 | 27.34 | 41.01 |  |  |
| 20.42 | 38.73 | 46.20 |  |  | 19.74 | 27.24 | 40.97 |  |  |
| 20.43 | 38.63 | 46.16 |  |  | 19.75 | 27.17 | 40.94 |  |  |
| 20.44 | 38.50 | 46.13 |  |  | 19.76 | 27.10 | 40.90 |  |  |
| 20.45 | 38.36 | 46.09 |  |  | 19.77 | 27.03 | 40.86 |  |  |
| 20.46 | 38.22 | 46.05 |  |  | 19.78 | 26.96 | 40.82 |  |  |
| 20.47 | 38.10 | 46.01 |  |  | 19.79 | 26.89 | 40.78 |  |  |
| 20.48 | 38.00 | 45.97 |  |  | 19.80 | 26.81 | 40.75 |  |  |
| 20.49 | 37.90 | 45.93 |  |  | 19.81 | 26.74 | 40.71 |  |  |
| 20.50 | 37.78 | 45.89 |  |  | 19.82 | 26.67 | 40.67 |  |  |
| 20.51 | 37.69 | 45.86 |  |  | 19.83 | 26.60 | 40.63 |  |  |
| 20.52 | 37.56 | 45.82 |  |  | 19.84 | 26.52 | 40.59 |  |  |
| 20.53 | 37.41 | 45.78 |  |  | 19.85 | 26.46 | 40.56 |  |  |
| 20.54 | 37.32 | 45.74 |  |  | 19.86 | 26.39 | 40.52 |  |  |
| 20.55 | 37.20 | 45.70 |  |  | 19.87 | 26.31 | 40.48 |  |  |
| 20.56 | 37.12 | 45.66 |  |  | 19.88 | 26.25 | 40.44 |  |  |
| 20.57 | 37.00 | 45.63 |  |  | 19.89 | 26.15 | 40.40 |  |  |
| 20.58 | 36.91 | 45.59 |  |  | 19.90 | 26.08 | 40.37 |  |  |
| 20.59 | 36.77 | 45.55 |  |  | 19.91 | 26.00 | 40.33 |  |  |
| 20.60 | 36.67 | 45.51 |  |  | 19.92 | 25.91 | 40.29 |  |  |
| 20.61 | 36.56 | 45.47 |  |  | 19.93 | 25.84 | 40.25 |  |  |
| 20.62 | 36.46 | 45.43 |  |  | 19.94 | 25.77 | 40.21 |  |  |
| 20.63 | 36.36 | 45.39 |  |  | 19.95 | 25.72 | 40.18 |  |  |
| 20.64 | 36.25 | 45.36 |  |  | 19.96 | 25.66 | 40.14 |  |  |
| 20.65 | 36.17 | 45.32 |  |  | 19.97 | 25.57 | 40.10 |  |  |
| 20.66 | 36.07 | 45.28 |  |  | 19.98 | 25.50 | 40.06 |  |  |
| 20.67 | 35.98 | 45.24 |  |  | 19.99 | 25.43 | 40.02 |  |  |
| 20.68 | 35.90 | 45.20 |  |  | 20.00 | 25.37 | 39.99 |  |  |
| 20.69 | 35.81 | 45.16 |  |  | 20.01 | 25.29 | 39.95 |  |  |
| 20.70 | 35.73 | 45.13 |  |  | 20.02 | 25.22 | 39.91 |  |  |
| 20.72 | 35.55 | 45.05 |  |  | 20.03 | 25.16 | 39.87 |  |  |
| 20.73 | 35.45 | 45.01 |  |  | 20.04 | 25.11 | 39.83 |  |  |
| 20.74 | 35.35 | 44.97 |  |  | 20.05 | 25.05 | 39.79 |  |  |
| 20.75 | 35.26 | 44.93 |  |  | 20.06 | 24.98 | 39.76 |  |  |
| 20.76 | 35.16 | 44.89 |  |  | 20.07 | 24.94 | 39.72 |  |  |
| 20.77 | 35.05 | 44.86 |  |  | 20.08 | 24.88 | 39.68 |  |  |
| 20.78 | 34.95 | 44.82 |  |  | 20.09 | 24.82 | 39.64 |  |  |
| 20.79 | 34.86 | 44.78 |  |  | 20.11 | 24.71 | 39.57 |  |  |
| 20.80 | 34.79 | 44.74 |  |  | 20.12 | 24.65 | 39.53 |  |  |
| 20.81 | 34.68 | 44.70 |  |  | 20.14 | 24.54 | 39.45 |  |  |
| 20.82 | 34.60 | 44.66 |  |  | 20.16 | 24.41 | 39.38 |  |  |
| 20.83 | 34.50 | 44.62 |  |  | 20.17 | 24.36 | 39.34 |  |  |
| 20.84 | 34.41 | 44.59 |  |  | 20.18 | 24.30 | 39.30 |  |  |
| 20.85 | 34.33 | 44.55 |  |  | 20.19 | 24.23 | 39.26 |  |  |
| 20.86 | 34.26 | 44.51 |  |  | 20.20 | 24.16 | 39.22 |  |  |
| 20.87 | 34.18 | 44.47 |  |  | 20.21 | 24.11 | 39.19 |  |  |
| 20.88 | 34.08 | 44.43 |  |  | 20.23 | 24.00 | 39.11 |  |  |
| 20.89 | 33.98 | 44.39 |  |  | 20.24 | 23.94 | 39.07 |  |  |
| 20.90 | 33.90 | 44.36 |  |  | 20.25 | 23.88 | 39.03 |  |  |
| 20.91 | 33.81 | 44.32 |  |  | 20.26 | 23.84 | 39.00 |  |  |
| 20.92 | 33.73 | 44.28 |  |  | 20.27 | 23.77 | 38.96 |  |  |
| 20.93 | 33.67 | 44.24 |  |  | 20.28 | 23.72 | 38.92 |  |  |
| 20.94 | 33.59 | 44.20 |  |  | 20.29 | 23.66 | 38.88 |  |  |
| 20.95 | 33.54 | 44.16 |  |  | 20.30 | 23.61 | 38.84 |  |  |
| 20.97 | 33.39 | 44.09 |  |  | 20.31 | 23.57 | 38.81 |  |  |
| 20.98 | 33.32 | 44.05 |  |  | 20.32 | 23.52 | 38.77 |  |  |
| 20.99 | 33.24 | 44.01 |  |  | 20.33 | 23.47 | 38.73 |  |  |
| 21.00 | 33.16 | 43.97 |  |  | 20.34 | 23.39 | 38.69 |  |  |
| 21.01 | 33.09 | 43.93 |  |  | 20.36 | 23.31 | 38.61 |  |  |
| 21.03 | 32.95 | 43.86 |  |  | 20.37 | 23.23 | 38.58 |  |  |
| 21.04 | 32.86 | 43.82 |  |  | 20.38 | 23.18 | 38.54 |  |  |
| 21.05 | 32.81 | 43.78 |  |  | 20.39 | 23.13 | 38.50 |  |  |
| 21.06 | 32.72 | 43.74 |  |  | 20.40 | 23.09 | 38.46 |  |  |
| 21.07 | 32.64 | 43.70 |  |  | 20.41 | 23.03 | 38.42 |  |  |
| 21.08 | 32.58 | 43.66 |  |  | 20.43 | 22.93 | 38.35 |  |  |
| 21.09 | 32.52 | 43.62 |  |  | 20.44 | 22.87 | 38.31 |  |  |
| 21.10 | 32.45 | 43.59 |  |  | 20.45 | 22.83 | 38.27 |  |  |
| 21.11 | 32.39 | 43.55 |  |  | 20.46 | 22.77 | 38.23 |  |  |
| 21.12 | 32.33 | 43.51 |  |  | 20.47 | 22.72 | 38.20 |  |  |
| 21.13 | 32.27 | 43.47 |  |  | 20.48 | 22.65 | 38.16 |  |  |
| 21.14 | 32.21 | 43.43 |  |  | 20.49 | 22.59 | 38.12 |  |  |
| 21.15 | 32.13 | 43.39 |  |  | 20.50 | 22.53 | 38.08 |  |  |
| 21.16 | 32.06 | 43.36 |  |  | 20.51 | 22.49 | 38.04 |  |  |
| 21.17 | 31.99 | 43.32 |  |  | 20.52 | 22.45 | 38.01 |  |  |
| 21.18 | 31.92 | 43.28 |  |  | 20.53 | 22.40 | 37.97 |  |  |
| 21.19 | 31.85 | 43.24 |  |  | 20.54 | 22.35 | 37.93 |  |  |
| 21.20 | 31.78 | 43.20 |  |  | 20.55 | 22.30 | 37.89 |  |  |
| 21.21 | 31.71 | 43.16 |  |  | 20.56 | 22.25 | 37.85 |  |  |
| 21.22 | 31.66 | 43.12 |  |  | 20.57 | 22.20 | 37.82 |  |  |
| 21.23 | 31.58 | 43.09 |  |  | 20.58 | 22.17 | 37.78 |  |  |
| 21.24 | 31.53 | 43.05 |  |  | 20.59 | 22.13 | 37.74 |  |  |
| 21.25 | 31.47 | 43.01 |  |  | 20.60 | 22.09 | 37.70 |  |  |
| 21.26 | 31.41 | 42.97 |  |  | 20.61 | 22.03 | 37.66 |  |  |
| 21.27 | 31.36 | 42.93 |  |  | 20.62 | 21.98 | 37.63 |  |  |
| 21.28 | 31.31 | 42.89 |  |  | 20.63 | 21.94 | 37.59 |  |  |
| 21.29 | 31.25 | 42.86 |  |  | 20.64 | 21.88 | 37.55 |  |  |
| 21.30 | 31.17 | 42.82 |  |  | 20.65 | 21.84 | 37.51 |  |  |
| 21.31 | 31.11 | 42.78 |  |  | 20.66 | 21.78 | 37.47 |  |  |
| 21.32 | 31.07 | 42.74 |  |  | 20.67 | 21.75 | 37.43 |  |  |
| 21.33 | 31.00 | 42.70 |  |  | 20.69 | 21.65 | 37.36 |  |  |
| 21.34 | 30.95 | 42.66 |  |  | 20.70 | 21.62 | 37.32 |  |  |
| 21.35 | 30.88 | 42.62 |  |  | 20.71 | 21.58 | 37.28 |  |  |
| 21.36 | 30.80 | 42.59 |  |  | 20.72 | 21.51 | 37.24 |  |  |
| 21.37 | 30.75 | 42.55 |  |  | 20.73 | 21.45 | 37.21 |  |  |
| 21.38 | 30.68 | 42.51 |  |  | 20.74 | 21.41 | 37.17 |  |  |
| 21.39 | 30.63 | 42.47 |  |  | 20.75 | 21.36 | 37.13 |  |  |
| 21.40 | 30.55 | 42.43 |  |  | 20.76 | 21.32 | 37.09 |  |  |
| 21.41 | 30.49 | 42.39 |  |  | 20.77 | 21.26 | 37.05 |  |  |
| 21.42 | 30.43 | 42.36 |  |  | 20.78 | 21.22 | 37.02 |  |  |
| 21.43 | 30.36 | 42.32 |  |  | 20.79 | 21.19 | 36.98 |  |  |
| 21.44 | 30.30 | 42.28 |  |  | 20.80 | 21.15 | 36.94 |  |  |
| 21.45 | 30.22 | 42.24 |  |  | 20.81 | 21.10 | 36.90 |  |  |
| 21.46 | 30.16 | 42.20 |  |  | 20.83 | 21.01 | 36.83 |  |  |
| 21.47 | 30.10 | 42.16 |  |  | 20.84 | 20.97 | 36.79 |  |  |
| 21.48 | 30.03 | 42.12 |  |  | 20.85 | 20.92 | 36.75 |  |  |
| 21.49 | 29.97 | 42.09 |  |  | 20.86 | 20.87 | 36.71 |  |  |
| 21.50 | 29.91 | 42.05 |  |  | 20.87 | 20.85 | 36.67 |  |  |
| 21.51 | 29.87 | 42.01 |  |  | 20.88 | 20.79 | 36.64 |  |  |
| 21.52 | 29.82 | 41.97 |  |  | 20.89 | 20.75 | 36.60 |  |  |
| 21.53 | 29.75 | 41.93 |  |  | 20.90 | 20.72 | 36.56 |  |  |
| 21.54 | 29.70 | 41.89 |  |  | 20.91 | 20.66 | 36.52 |  |  |
| 21.55 | 29.63 | 41.86 |  |  | 20.92 | 20.62 | 36.48 |  |  |
| 21.56 | 29.58 | 41.82 |  |  | 20.93 | 20.59 | 36.45 |  |  |
| 21.57 | 29.50 | 41.78 |  |  | 20.94 | 20.54 | 36.41 |  |  |
| 21.58 | 29.44 | 41.74 |  |  | 20.95 | 20.50 | 36.37 |  |  |
| 21.59 | 29.38 | 41.70 |  |  | 20.96 | 20.45 | 36.33 |  |  |
| 21.60 | 29.33 | 41.66 |  |  | 20.97 | 20.42 | 36.29 |  |  |
| 21.61 | 29.28 | 41.62 |  |  | 20.98 | 20.37 | 36.26 |  |  |
| 21.62 | 29.21 | 41.59 |  |  | 20.99 | 20.34 | 36.22 |  |  |
| 21.64 | 29.12 | 41.51 |  |  | 21.00 | 20.31 | 36.18 |  |  |
| 21.65 | 29.08 | 41.47 |  |  | 21.01 | 20.26 | 36.14 |  |  |
| 21.66 | 29.03 | 41.43 |  |  | 21.02 | 20.23 | 36.10 |  |  |
| 21.67 | 28.99 | 41.39 |  |  | 21.03 | 20.18 | 36.06 |  |  |
| 21.68 | 28.93 | 41.35 |  |  | 21.04 | 20.12 | 36.03 |  |  |
| 21.69 | 28.88 | 41.32 |  |  | 21.05 | 20.09 | 35.99 |  |  |
| 21.70 | 28.82 | 41.28 |  |  | 21.06 | 20.04 | 35.95 |  |  |
| 21.71 | 28.78 | 41.24 |  |  | 21.08 | 19.98 | 35.87 |  |  |
| 21.72 | 28.74 | 41.20 |  |  | 21.09 | 19.94 | 35.84 |  |  |
| 21.73 | 28.69 | 41.16 |  |  | 21.10 | 19.89 | 35.80 |  |  |
| 21.74 | 28.62 | 41.12 |  |  | 21.12 | 19.81 | 35.72 |  |  |
| 21.75 | 28.57 | 41.09 |  |  | 21.13 | 19.77 | 35.68 |  |  |
| 21.76 | 28.52 | 41.05 |  |  | 21.14 | 19.72 | 35.65 |  |  |
| 21.77 | 28.47 | 41.01 |  |  | 21.15 | 19.68 | 35.61 |  |  |
| 21.78 | 28.43 | 40.97 |  |  | 21.16 | 19.63 | 35.57 |  |  |
| 21.79 | 28.38 | 40.93 |  |  | 21.17 | 19.58 | 35.53 |  |  |
| 21.81 | 28.30 | 40.85 |  |  | 21.18 | 19.55 | 35.49 |  |  |
| 21.82 | 28.25 | 40.82 |  |  | 21.19 | 19.50 | 35.46 |  |  |
| 21.83 | 28.20 | 40.78 |  |  | 21.20 | 19.47 | 35.42 |  |  |
| 21.84 | 28.14 | 40.74 |  |  | 21.21 | 19.42 | 35.38 |  |  |
| 21.85 | 28.11 | 40.70 |  |  | 21.22 | 19.39 | 35.34 |  |  |
| 21.87 | 28.01 | 40.62 |  |  | 21.23 | 19.34 | 35.30 |  |  |
| 21.88 | 27.97 | 40.59 |  |  | 21.24 | 19.31 | 35.27 |  |  |
| 21.89 | 27.92 | 40.55 |  |  | 21.25 | 19.26 | 35.23 |  |  |
| 21.90 | 27.88 | 40.51 |  |  | 21.26 | 19.23 | 35.19 |  |  |
| 21.91 | 27.81 | 40.47 |  |  | 21.27 | 19.16 | 35.15 |  |  |
| 21.92 | 27.77 | 40.43 |  |  | 21.28 | 19.10 | 35.11 |  |  |
| 21.93 | 27.73 | 40.39 |  |  | 21.29 | 19.06 | 35.08 |  |  |
| 21.94 | 27.67 | 40.35 |  |  | 21.30 | 19.03 | 35.04 |  |  |
| 21.95 | 27.63 | 40.32 |  |  | 21.31 | 18.99 | 35.00 |  |  |
| 21.96 | 27.60 | 40.28 |  |  | 21.32 | 18.94 | 34.96 |  |  |
| 21.97 | 27.56 | 40.24 |  |  | 21.33 | 18.90 | 34.92 |  |  |
| 21.98 | 27.52 | 40.20 |  |  | 21.34 | 18.85 | 34.88 |  |  |
| 21.99 | 27.45 | 40.16 |  |  | 21.35 | 18.81 | 34.85 |  |  |
| 22.00 | 27.41 | 40.12 |  |  | 21.36 | 18.77 | 34.81 |  |  |
| 22.01 | 27.36 | 40.09 |  |  | 21.38 | 18.66 | 34.73 |  |  |
| 22.03 | 27.28 | 40.01 |  |  | 21.39 | 18.62 | 34.69 |  |  |
| 22.04 | 27.24 | 39.97 |  |  | 21.40 | 18.55 | 34.66 |  |  |
| 22.06 | 27.15 | 39.89 |  |  | 21.41 | 18.50 | 34.62 |  |  |
| 22.08 | 27.07 | 39.82 |  |  | 21.42 | 18.45 | 34.58 |  |  |
| 22.09 | 27.03 | 39.78 |  |  | 21.43 | 18.39 | 34.54 |  |  |
| 22.10 | 26.99 | 39.74 |  |  | 21.44 | 18.33 | 34.50 |  |  |
| 22.11 | 26.95 | 39.70 |  |  | 21.45 | 18.28 | 34.47 |  |  |
| 22.12 | 26.93 | 39.66 |  |  | 21.46 | 18.23 | 34.43 |  |  |
| 22.13 | 26.89 | 39.62 |  |  | 21.49 | 18.11 | 34.31 |  |  |
| 22.14 | 26.85 | 39.59 |  |  | 21.50 | 18.08 | 34.28 |  |  |
| 22.15 | 26.82 | 39.55 |  |  | 21.51 | 18.04 | 34.24 |  |  |
| 22.16 | 26.79 | 39.51 |  |  | 21.52 | 18.00 | 34.20 |  |  |
| 22.17 | 26.76 | 39.47 |  |  | 21.53 | 17.97 | 34.16 |  |  |
| 22.18 | 26.72 | 39.43 |  |  | 21.54 | 17.92 | 34.12 |  |  |
| 22.19 | 26.69 | 39.39 |  |  | 21.55 | 17.89 | 34.09 |  |  |
| 22.20 | 26.65 | 39.35 |  |  | 21.56 | 17.85 | 34.05 |  |  |
| 22.21 | 26.60 | 39.32 |  |  | 21.57 | 17.79 | 34.01 |  |  |
| 22.22 | 26.57 | 39.28 |  |  | 21.58 | 17.74 | 33.97 |  |  |
| 22.23 | 26.54 | 39.24 |  |  | 21.59 | 17.71 | 33.93 |  |  |
| 22.24 | 26.49 | 39.20 |  |  | 21.60 | 17.67 | 33.90 |  |  |
| 22.25 | 26.43 | 39.16 |  |  | 21.61 | 17.63 | 33.86 |  |  |
| 22.26 | 26.40 | 39.12 |  |  | 21.62 | 17.58 | 33.82 |  |  |
| 22.27 | 26.37 | 39.09 |  |  | 21.63 | 17.55 | 33.78 |  |  |
| 22.28 | 26.34 | 39.05 |  |  | 21.64 | 17.51 | 33.74 |  |  |
| 22.29 | 26.30 | 39.01 |  |  | 21.65 | 17.48 | 33.70 |  |  |
| 22.30 | 26.27 | 38.97 |  |  | 21.66 | 17.45 | 33.67 |  |  |
| 22.31 | 26.23 | 38.93 |  |  | 21.67 | 17.40 | 33.63 |  |  |
| 22.32 | 26.20 | 38.89 |  |  | 21.68 | 17.36 | 33.59 |  |  |
| 22.33 | 26.16 | 38.85 |  |  | 21.69 | 17.33 | 33.55 |  |  |
| 22.34 | 26.12 | 38.82 |  |  | 21.70 | 17.30 | 33.51 |  |  |
| 22.35 | 26.08 | 38.78 |  |  | 21.71 | 17.26 | 33.48 |  |  |
| 22.36 | 26.04 | 38.74 |  |  | 21.72 | 17.23 | 33.44 |  |  |
| 22.37 | 26.02 | 38.70 |  |  | 21.74 | 17.17 | 33.36 |  |  |
| 22.38 | 25.98 | 38.66 |  |  | 21.75 | 17.14 | 33.32 |  |  |
| 22.39 | 25.94 | 38.62 |  |  | 21.76 | 17.11 | 33.29 |  |  |
| 22.40 | 25.92 | 38.59 |  |  | 21.77 | 17.08 | 33.25 |  |  |
| 22.41 | 25.88 | 38.55 |  |  | 21.78 | 17.05 | 33.21 |  |  |
| 22.42 | 25.84 | 38.51 |  |  | 21.79 | 17.02 | 33.17 |  |  |
| 22.43 | 25.79 | 38.47 |  |  | 21.80 | 16.98 | 33.13 |  |  |
| 22.44 | 25.75 | 38.43 |  |  | 21.81 | 16.93 | 33.10 |  |  |
| 22.45 | 25.73 | 38.39 |  |  | 21.82 | 16.90 | 33.06 |  |  |
| 22.46 | 25.69 | 38.35 |  |  | 21.83 | 16.86 | 33.02 |  |  |
| 22.48 | 25.63 | 38.28 |  |  | 21.84 | 16.82 | 32.98 |  |  |
| 22.49 | 25.61 | 38.24 |  |  | 21.85 | 16.80 | 32.94 |  |  |
| 22.50 | 25.57 | 38.20 |  |  | 21.86 | 16.77 | 32.91 |  |  |
| 22.51 | 25.52 | 38.16 |  |  | 21.87 | 16.73 | 32.87 |  |  |
| 22.52 | 25.49 | 38.12 |  |  | 21.88 | 16.69 | 32.83 |  |  |
| 22.53 | 25.45 | 38.09 |  |  | 21.89 | 16.65 | 32.79 |  |  |
| 22.54 | 25.40 | 38.05 |  |  | 21.90 | 16.61 | 32.75 |  |  |
| 22.55 | 25.38 | 38.01 |  |  | 21.91 | 16.57 | 32.72 |  |  |
| 22.56 | 25.33 | 37.97 |  |  | 21.92 | 16.53 | 32.68 |  |  |
| 22.57 | 25.30 | 37.93 |  |  | 21.93 | 16.50 | 32.64 |  |  |
| 22.58 | 25.26 | 37.89 |  |  | 21.94 | 16.48 | 32.60 |  |  |
| 22.59 | 25.23 | 37.85 |  |  | 21.95 | 16.45 | 32.56 |  |  |
| 22.60 | 25.20 | 37.82 |  |  | 21.96 | 16.43 | 32.52 |  |  |
| 22.61 | 25.16 | 37.78 |  |  | 21.97 | 16.40 | 32.49 |  |  |
| 22.62 | 25.12 | 37.74 |  |  | 21.98 | 16.38 | 32.45 |  |  |
| 22.63 | 25.07 | 37.70 |  |  | 21.99 | 16.36 | 32.41 |  |  |
| 22.64 | 25.02 | 37.66 |  |  | 22.00 | 16.32 | 32.37 |  |  |
| 22.65 | 24.96 | 37.62 |  |  | 22.01 | 16.29 | 32.33 |  |  |
| 22.67 | 24.88 | 37.55 |  |  | 22.02 | 16.26 | 32.30 |  |  |
| 22.69 | 24.79 | 37.47 |  |  | 22.03 | 16.24 | 32.26 |  |  |
| 22.70 | 24.74 | 37.43 |  |  | 22.04 | 16.22 | 32.22 |  |  |
| 22.71 | 24.70 | 37.39 |  |  | 22.05 | 16.18 | 32.18 |  |  |
| 22.72 | 24.65 | 37.35 |  |  | 22.06 | 16.15 | 32.14 |  |  |
| 22.73 | 24.63 | 37.32 |  |  | 22.07 | 16.13 | 32.11 |  |  |
| 22.74 | 24.59 | 37.28 |  |  | 22.08 | 16.11 | 32.07 |  |  |
| 22.75 | 24.54 | 37.24 |  |  | 22.09 | 16.09 | 32.03 |  |  |
| 22.76 | 24.51 | 37.20 |  |  | 22.10 | 16.07 | 31.99 |  |  |
| 22.77 | 24.47 | 37.16 |  |  | 22.11 | 16.04 | 31.95 |  |  |
| 22.78 | 24.43 | 37.12 |  |  | 22.12 | 16.02 | 31.92 |  |  |
| 22.79 | 24.39 | 37.08 |  |  | 22.14 | 15.95 | 31.84 |  |  |
| 22.80 | 24.36 | 37.05 |  |  | 22.15 | 15.93 | 31.80 |  |  |
| 22.81 | 24.33 | 37.01 |  |  | 22.16 | 15.91 | 31.76 |  |  |
| 22.83 | 24.26 | 36.93 |  |  | 22.17 | 15.88 | 31.73 |  |  |
| 22.84 | 24.21 | 36.89 |  |  | 22.18 | 15.84 | 31.69 |  |  |
| 22.85 | 24.18 | 36.85 |  |  | 22.21 | 15.74 | 31.57 |  |  |
| 22.86 | 24.14 | 36.82 |  |  | 22.22 | 15.71 | 31.54 |  |  |
| 22.87 | 24.11 | 36.78 |  |  | 22.23 | 15.68 | 31.50 |  |  |
| 22.88 | 24.07 | 36.74 |  |  | 22.24 | 15.64 | 31.46 |  |  |
| 22.89 | 24.03 | 36.70 |  |  | 22.25 | 15.61 | 31.42 |  |  |
| 22.90 | 24.00 | 36.66 |  |  | 22.26 | 15.57 | 31.38 |  |  |
| 22.91 | 23.97 | 36.62 |  |  | 22.27 | 15.55 | 31.35 |  |  |
| 22.92 | 23.93 | 36.58 |  |  | 22.28 | 15.53 | 31.31 |  |  |
| 22.93 | 23.90 | 36.55 |  |  | 22.29 | 15.50 | 31.27 |  |  |
| 22.94 | 23.87 | 36.51 |  |  | 22.30 | 15.46 | 31.23 |  |  |
| 22.95 | 23.84 | 36.47 |  |  | 22.31 | 15.43 | 31.19 |  |  |
| 22.96 | 23.80 | 36.43 |  |  | 22.32 | 15.41 | 31.15 |  |  |
| 22.97 | 23.75 | 36.39 |  |  | 22.33 | 15.39 | 31.12 |  |  |
| 22.98 | 23.74 | 36.35 |  |  | 22.34 | 15.35 | 31.08 |  |  |
| 22.99 | 23.70 | 36.32 |  |  | 22.35 | 15.31 | 31.04 |  |  |
| 23.00 | 23.67 | 36.28 |  |  | 22.36 | 15.28 | 31.00 |  |  |
| 23.02 | 23.62 | 36.20 |  |  | 22.37 | 15.25 | 30.96 |  |  |
| 23.03 | 23.58 | 36.16 |  |  | 22.38 | 15.24 | 30.93 |  |  |
| 23.04 | 23.56 | 36.12 |  |  | 22.39 | 15.22 | 30.89 |  |  |
| 23.05 | 23.54 | 36.08 |  |  | 22.40 | 15.20 | 30.85 |  |  |
| 23.06 | 23.51 | 36.05 |  |  | 22.41 | 15.18 | 30.81 |  |  |
| 23.07 | 23.49 | 36.01 |  |  | 22.42 | 15.18 | 30.77 |  |  |
| 23.09 | 23.42 | 35.93 |  |  | 22.43 | 15.14 | 30.74 |  |  |
| 23.11 | 23.37 | 35.85 |  |  | 22.44 | 15.11 | 30.70 |  |  |
| 23.12 | 23.35 | 35.82 |  |  | 22.45 | 15.09 | 30.66 |  |  |
| 23.13 | 23.32 | 35.78 |  |  | 22.46 | 15.07 | 30.62 |  |  |
| 23.14 | 23.31 | 35.74 |  |  | 22.49 | 14.99 | 30.51 |  |  |
| 23.15 | 23.27 | 35.70 |  |  | 22.50 | 14.97 | 30.47 |  |  |
| 23.16 | 23.26 | 35.66 |  |  | 22.51 | 14.94 | 30.43 |  |  |
| 23.17 | 23.23 | 35.62 |  |  | 22.52 | 14.89 | 30.39 |  |  |
| 23.19 | 23.18 | 35.55 |  |  | 22.54 | 14.83 | 30.32 |  |  |
| 23.20 | 23.16 | 35.51 |  |  | 22.55 | 14.80 | 30.28 |  |  |
| 23.21 | 23.13 | 35.47 |  |  | 22.56 | 14.77 | 30.24 |  |  |
| 23.22 | 23.12 | 35.43 |  |  | 22.57 | 14.74 | 30.20 |  |  |
| 23.24 | 23.08 | 35.35 |  |  | 22.58 | 14.71 | 30.17 |  |  |
| 23.25 | 23.05 | 35.32 |  |  | 22.59 | 14.70 | 30.13 |  |  |
| 23.26 | 23.02 | 35.28 |  |  | 22.60 | 14.65 | 30.09 |  |  |
| 23.27 | 23.01 | 35.24 |  |  | 22.61 | 14.63 | 30.05 |  |  |
| 23.28 | 22.98 | 35.20 |  |  | 22.62 | 14.60 | 30.01 |  |  |
| 23.29 | 22.94 | 35.16 |  |  | 22.63 | 14.58 | 29.97 |  |  |
| 23.30 | 22.92 | 35.12 |  |  | 22.66 | 14.47 | 29.86 |  |  |
| 23.31 | 22.89 | 35.08 |  |  | 22.67 | 14.44 | 29.82 |  |  |
| 23.32 | 22.84 | 35.05 |  |  | 22.68 | 14.41 | 29.78 |  |  |
| 23.33 | 22.80 | 35.01 |  |  | 22.69 | 14.38 | 29.75 |  |  |
| 23.34 | 22.77 | 34.97 |  |  | 22.70 | 14.36 | 29.71 |  |  |
| 23.35 | 22.76 | 34.93 |  |  | 22.71 | 14.34 | 29.67 |  |  |
| 23.36 | 22.74 | 34.89 |  |  | 22.72 | 14.32 | 29.63 |  |  |
| 23.37 | 22.71 | 34.85 |  |  | 22.73 | 14.29 | 29.59 |  |  |
| 23.38 | 22.70 | 34.82 |  |  | 22.74 | 14.27 | 29.56 |  |  |
| 23.39 | 22.67 | 34.78 |  |  | 22.75 | 14.25 | 29.52 |  |  |
| 23.40 | 22.64 | 34.74 |  |  | 22.76 | 14.21 | 29.48 |  |  |
| 23.41 | 22.62 | 34.70 |  |  | 22.77 | 14.17 | 29.44 |  |  |
| 23.42 | 22.60 | 34.66 |  |  | 22.78 | 14.15 | 29.40 |  |  |
| 23.43 | 22.58 | 34.62 |  |  | 22.79 | 14.14 | 29.37 |  |  |
| 23.44 | 22.55 | 34.58 |  |  | 22.80 | 14.13 | 29.33 |  |  |
| 23.45 | 22.51 | 34.55 |  |  | 22.81 | 14.11 | 29.29 |  |  |
| 23.46 | 22.47 | 34.51 |  |  | 22.82 | 14.09 | 29.25 |  |  |
| 23.47 | 22.45 | 34.47 |  |  | 22.83 | 14.06 | 29.21 |  |  |
| 23.48 | 22.42 | 34.43 |  |  | 22.84 | 14.03 | 29.18 |  |  |
| 23.49 | 22.37 | 34.39 |  |  | 22.85 | 14.00 | 29.14 |  |  |
| 23.50 | 22.32 | 34.35 |  |  | 22.86 | 13.97 | 29.10 |  |  |
| 23.52 | 22.28 | 34.28 |  |  | 22.87 | 13.94 | 29.06 |  |  |
| 23.53 | 22.26 | 34.24 |  |  | 22.88 | 13.91 | 29.02 |  |  |
| 23.54 | 22.23 | 34.20 |  |  | 22.89 | 13.88 | 28.99 |  |  |
| 23.55 | 22.20 | 34.16 |  |  | 22.90 | 13.87 | 28.95 |  |  |
| 23.56 | 22.17 | 34.12 |  |  | 22.91 | 13.84 | 28.91 |  |  |
| 23.57 | 22.15 | 34.08 |  |  | 22.92 | 13.82 | 28.87 |  |  |
| 23.58 | 22.12 | 34.05 |  |  | 22.93 | 13.80 | 28.83 |  |  |
| 23.59 | 22.09 | 34.01 |  |  | 22.94 | 13.78 | 28.79 |  |  |
| 23.60 | 22.07 | 33.97 |  |  | 22.95 | 13.75 | 28.76 |  |  |
| 23.61 | 22.03 | 33.93 |  |  | 22.96 | 13.73 | 28.72 |  |  |
| 23.62 | 22.00 | 33.89 |  |  | 23.00 | 13.64 | 28.57 |  |  |
| 23.63 | 21.96 | 33.85 |  |  | 23.01 | 13.61 | 28.53 |  |  |
| 23.64 | 21.94 | 33.81 |  |  | 23.02 | 13.58 | 28.49 |  |  |
| 23.65 | 21.91 | 33.78 |  |  | 23.04 | 13.54 | 28.41 |  |  |
| 23.66 | 21.88 | 33.74 |  |  | 23.05 | 13.52 | 28.38 |  |  |
| 23.67 | 21.85 | 33.70 |  |  | 23.06 | 13.52 | 28.34 |  |  |
| 23.68 | 21.82 | 33.66 |  |  | 23.07 | 13.50 | 28.30 |  |  |
| 23.69 | 21.78 | 33.62 |  |  | 23.08 | 13.47 | 28.26 |  |  |
| 23.70 | 21.76 | 33.58 |  |  | 23.09 | 13.45 | 28.22 |  |  |
| 23.71 | 21.74 | 33.55 |  |  | 23.10 | 13.43 | 28.19 |  |  |
| 23.72 | 21.71 | 33.51 |  |  | 23.11 | 13.40 | 28.15 |  |  |
| 23.73 | 21.69 | 33.47 |  |  | 23.12 | 13.39 | 28.11 |  |  |
| 23.74 | 21.66 | 33.43 |  |  | 23.13 | 13.36 | 28.07 |  |  |
| 23.76 | 21.59 | 33.35 |  |  | 23.14 | 13.33 | 28.03 |  |  |
| 23.77 | 21.58 | 33.31 |  |  | 23.15 | 13.31 | 28.00 |  |  |
| 23.78 | 21.56 | 33.28 |  |  | 23.16 | 13.28 | 27.96 |  |  |
| 23.79 | 21.53 | 33.24 |  |  | 23.17 | 13.26 | 27.92 |  |  |
| 23.80 | 21.50 | 33.20 |  |  | 23.18 | 13.23 | 27.88 |  |  |
| 23.81 | 21.48 | 33.16 |  |  | 23.19 | 13.21 | 27.84 |  |  |
| 23.82 | 21.45 | 33.12 |  |  | 23.20 | 13.17 | 27.81 |  |  |
| 23.83 | 21.42 | 33.08 |  |  | 23.21 | 13.14 | 27.77 |  |  |
| 23.84 | 21.39 | 33.05 |  |  | 23.22 | 13.11 | 27.73 |  |  |
| 23.85 | 21.36 | 33.01 |  |  | 23.23 | 13.08 | 27.69 |  |  |
| 23.86 | 21.35 | 32.97 |  |  | 23.24 | 13.07 | 27.65 |  |  |
| 23.87 | 21.31 | 32.93 |  |  | 23.25 | 13.06 | 27.62 |  |  |
| 23.88 | 21.29 | 32.89 |  |  | 23.27 | 13.03 | 27.54 |  |  |
| 23.89 | 21.28 | 32.85 |  |  | 23.28 | 13.02 | 27.50 |  |  |
| 23.90 | 21.25 | 32.81 |  |  | 23.30 | 12.99 | 27.42 |  |  |
| 23.91 | 21.24 | 32.78 |  |  | 23.31 | 12.97 | 27.39 |  |  |
| 23.92 | 21.21 | 32.74 |  |  | 23.33 | 12.95 | 27.31 |  |  |
| 23.93 | 21.18 | 32.70 |  |  | 23.34 | 12.93 | 27.27 |  |  |
| 23.95 | 21.13 | 32.62 |  |  | 23.35 | 12.92 | 27.23 |  |  |
| 23.96 | 21.11 | 32.58 |  |  | 23.36 | 12.90 | 27.20 |  |  |
| 23.97 | 21.09 | 32.55 |  |  | 23.37 | 12.89 | 27.16 |  |  |
| 23.98 | 21.08 | 32.51 |  |  | 23.38 | 12.87 | 27.12 |  |  |
| 23.99 | 21.06 | 32.47 |  |  | 23.39 | 12.85 | 27.08 |  |  |
| 24.01 | 21.00 | 32.39 |  |  | 23.40 | 12.84 | 27.04 |  |  |
| 24.02 | 20.97 | 32.35 |  |  | 23.41 | 12.82 | 27.01 |  |  |
| 24.04 | 20.95 | 32.28 |  |  | 23.42 | 12.81 | 26.97 |  |  |
| 24.05 | 20.90 | 32.24 |  |  | 23.43 | 12.80 | 26.93 |  |  |
| 24.06 | 20.86 | 32.20 |  |  | 23.44 | 12.79 | 26.89 |  |  |
| 24.07 | 20.83 | 32.16 |  |  | 23.46 | 12.76 | 26.82 |  |  |
| 24.08 | 20.80 | 32.12 |  |  | 23.47 | 12.73 | 26.78 |  |  |
| 24.09 | 20.79 | 32.08 |  |  | 23.48 | 12.72 | 26.74 |  |  |
| 24.10 | 20.76 | 32.05 |  |  | 23.49 | 12.71 | 26.70 |  |  |
| 24.11 | 20.73 | 32.01 |  |  | 23.50 | 12.71 | 26.66 |  |  |
| 24.13 | 20.69 | 31.93 |  |  | 23.51 | 12.69 | 26.63 |  |  |
| 24.14 | 20.67 | 31.89 |  |  | 23.52 | 12.67 | 26.59 |  |  |
| 24.15 | 20.64 | 31.85 |  |  | 23.53 | 12.64 | 26.55 |  |  |
| 24.16 | 20.63 | 31.81 |  |  | 23.55 | 12.61 | 26.47 |  |  |
| 24.18 | 20.56 | 31.74 |  |  | 23.56 | 12.59 | 26.44 |  |  |
| 24.19 | 20.53 | 31.70 |  |  | 23.57 | 12.58 | 26.40 |  |  |
| 24.20 | 20.50 | 31.66 |  |  | 23.58 | 12.56 | 26.36 |  |  |
| 24.21 | 20.48 | 31.62 |  |  | 23.59 | 12.53 | 26.32 |  |  |
| 24.22 | 20.44 | 31.58 |  |  | 23.60 | 12.52 | 26.28 |  |  |
| 24.23 | 20.43 | 31.55 |  |  | 23.61 | 12.49 | 26.24 |  |  |
| 24.24 | 20.40 | 31.51 |  |  | 23.62 | 12.47 | 26.21 |  |  |
| 24.26 | 20.34 | 31.43 |  |  | 23.63 | 12.46 | 26.17 |  |  |
| 24.27 | 20.32 | 31.39 |  |  | 23.64 | 12.43 | 26.13 |  |  |
| 24.28 | 20.29 | 31.35 |  |  | 23.65 | 12.41 | 26.09 |  |  |
| 24.30 | 20.21 | 31.28 |  |  | 23.66 | 12.38 | 26.05 |  |  |
| 24.31 | 20.19 | 31.24 |  |  | 23.67 | 12.37 | 26.02 |  |  |
| 24.32 | 20.16 | 31.20 |  |  | 23.68 | 12.34 | 25.98 |  |  |
| 24.33 | 20.14 | 31.16 |  |  | 23.69 | 12.32 | 25.94 |  |  |
| 24.34 | 20.12 | 31.12 |  |  | 23.70 | 12.29 | 25.90 |  |  |
| 24.35 | 20.10 | 31.08 |  |  | 23.71 | 12.26 | 25.86 |  |  |
| 24.36 | 20.09 | 31.04 |  |  | 23.72 | 12.24 | 25.83 |  |  |
| 24.37 | 20.07 | 31.01 |  |  | 23.73 | 12.22 | 25.79 |  |  |
| 24.38 | 20.04 | 30.97 |  |  | 23.74 | 12.20 | 25.75 |  |  |
| 24.39 | 20.00 | 30.93 |  |  | 23.75 | 12.20 | 25.71 |  |  |
| 24.41 | 19.96 | 30.85 |  |  | 23.76 | 12.16 | 25.67 |  |  |
| 24.42 | 19.93 | 30.81 |  |  | 23.77 | 12.14 | 25.64 |  |  |
| 24.43 | 19.90 | 30.78 |  |  | 23.78 | 12.13 | 25.60 |  |  |
| 24.45 | 19.85 | 30.70 |  |  | 23.79 | 12.10 | 25.56 |  |  |
| 24.46 | 19.83 | 30.66 |  |  | 23.80 | 12.10 | 25.52 |  |  |
| 24.47 | 19.80 | 30.62 |  |  | 23.81 | 12.09 | 25.48 |  |  |
| 24.48 | 19.77 | 30.58 |  |  | 23.82 | 12.07 | 25.45 |  |  |
| 24.49 | 19.75 | 30.54 |  |  | 23.83 | 12.05 | 25.41 |  |  |
| 24.50 | 19.73 | 30.51 |  |  | 23.84 | 12.03 | 25.37 |  |  |
| 24.51 | 19.72 | 30.47 |  |  | 23.85 | 12.02 | 25.33 |  |  |
| 24.52 | 19.70 | 30.43 |  |  | 23.86 | 12.01 | 25.29 |  |  |
| 24.54 | 19.65 | 30.35 |  |  | 23.87 | 12.00 | 25.26 |  |  |
| 24.55 | 19.61 | 30.31 |  |  | 23.88 | 11.99 | 25.22 |  |  |
| 24.56 | 19.59 | 30.28 |  |  | 23.90 | 11.96 | 25.14 |  |  |
| 24.57 | 19.57 | 30.24 |  |  | 23.91 | 11.95 | 25.10 |  |  |
| 24.58 | 19.53 | 30.20 |  |  | 23.92 | 11.93 | 25.06 |  |  |
| 24.61 | 19.46 | 30.08 |  |  | 23.94 | 11.91 | 24.99 |  |  |
| 24.62 | 19.44 | 30.04 |  |  | 23.95 | 11.89 | 24.95 |  |  |
| 24.63 | 19.41 | 30.01 |  |  | 23.97 | 11.86 | 24.87 |  |  |
| 24.64 | 19.39 | 29.97 |  |  | 23.98 | 11.85 | 24.84 |  |  |
| 24.65 | 19.37 | 29.93 |  |  | 23.99 | 11.84 | 24.80 |  |  |
| 24.66 | 19.34 | 29.89 |  |  | 24.00 | 11.83 | 24.76 |  |  |
| 24.67 | 19.32 | 29.85 |  |  | 24.01 | 11.81 | 24.72 |  |  |
| 24.68 | 19.29 | 29.81 |  |  | 24.02 | 11.80 | 24.68 |  |  |
| 24.70 | 19.25 | 29.74 |  |  | 24.03 | 11.78 | 24.65 |  |  |
| 24.71 | 19.22 | 29.70 |  |  | 24.04 | 11.77 | 24.61 |  |  |
| 24.72 | 19.20 | 29.66 |  |  | 24.05 | 11.76 | 24.57 |  |  |
| 24.73 | 19.17 | 29.62 |  |  | 24.06 | 11.75 | 24.53 |  |  |
| 24.74 | 19.15 | 29.58 |  |  | 24.07 | 11.74 | 24.49 |  |  |
| 24.75 | 19.14 | 29.54 |  |  | 24.08 | 11.73 | 24.46 |  |  |
| 24.77 | 19.10 | 29.47 |  |  | 24.09 | 11.71 | 24.42 |  |  |
| 24.78 | 19.07 | 29.43 |  |  | 24.10 | 11.70 | 24.38 |  |  |
| 24.79 | 19.06 | 29.39 |  |  | 24.11 | 11.68 | 24.34 |  |  |
| 24.80 | 19.04 | 29.35 |  |  | 24.12 | 11.67 | 24.30 |  |  |
| 24.82 | 19.01 | 29.28 |  |  | 24.13 | 11.66 | 24.27 |  |  |
| 24.83 | 18.99 | 29.24 |  |  | 24.14 | 11.64 | 24.23 |  |  |
| 24.84 | 18.97 | 29.20 |  |  | 24.15 | 11.62 | 24.19 |  |  |
| 24.85 | 18.96 | 29.16 |  |  | 24.16 | 11.60 | 24.15 |  |  |
| 24.86 | 18.93 | 29.12 |  |  | 24.17 | 11.59 | 24.11 |  |  |
| 24.87 | 18.92 | 29.08 |  |  | 24.18 | 11.58 | 24.08 |  |  |
| 24.88 | 18.89 | 29.04 |  |  | 24.19 | 11.57 | 24.04 |  |  |
| 24.90 | 18.86 | 28.97 |  |  | 24.20 | 11.55 | 24.00 |  |  |
| 24.91 | 18.84 | 28.93 |  |  | 24.21 | 11.54 | 23.96 |  |  |
| 24.92 | 18.82 | 28.89 |  |  | 24.22 | 11.54 | 23.92 |  |  |
| 24.93 | 18.79 | 28.85 |  |  | 24.23 | 11.52 | 23.89 |  |  |
| 24.94 | 18.78 | 28.81 |  |  | 24.24 | 11.51 | 23.85 |  |  |
| 24.95 | 18.77 | 28.78 |  |  | 24.25 | 11.51 | 23.81 |  |  |
| 24.96 | 18.76 | 28.74 |  |  | 24.26 | 11.49 | 23.77 |  |  |
| 24.97 | 18.74 | 28.70 |  |  | 24.27 | 11.48 | 23.73 |  |  |
| 24.98 | 18.71 | 28.66 |  |  | 24.28 | 11.47 | 23.69 |  |  |
| 24.99 | 18.68 | 28.62 |  |  | 24.29 | 11.45 | 23.66 |  |  |
| 25.01 | 18.64 | 28.54 |  |  | 24.31 | 11.43 | 23.58 |  |  |
| 25.02 | 18.62 | 28.51 |  |  | 24.32 | 11.42 | 23.54 |  |  |
| 25.03 | 18.60 | 28.47 |  |  | 24.33 | 11.40 | 23.50 |  |  |
| 25.04 | 18.59 | 28.43 |  |  | 24.34 | 11.39 | 23.47 |  |  |
| 25.05 | 18.55 | 28.39 |  |  | 24.35 | 11.37 | 23.43 |  |  |
| 25.06 | 18.52 | 28.35 |  |  | 24.36 | 11.36 | 23.39 |  |  |
| 25.07 | 18.50 | 28.31 |  |  | 24.37 | 11.35 | 23.35 |  |  |
| 25.08 | 18.46 | 28.28 |  |  | 24.38 | 11.33 | 23.31 |  |  |
| 25.09 | 18.44 | 28.24 |  |  | 24.39 | 11.32 | 23.28 |  |  |
| 25.11 | 18.40 | 28.16 |  |  | 24.40 | 11.30 | 23.24 |  |  |
| 25.12 | 18.38 | 28.12 |  |  | 24.42 | 11.27 | 23.16 |  |  |
| 25.13 | 18.36 | 28.08 |  |  | 24.43 | 11.26 | 23.12 |  |  |
| 25.14 | 18.34 | 28.04 |  |  | 24.44 | 11.24 | 23.09 |  |  |
| 25.15 | 18.33 | 28.01 |  |  | 24.45 | 11.23 | 23.05 |  |  |
| 25.17 | 18.27 | 27.93 |  |  | 24.46 | 11.21 | 23.01 |  |  |
| 25.18 | 18.25 | 27.89 |  |  | 24.47 | 11.19 | 22.97 |  |  |
| 25.19 | 18.23 | 27.85 |  |  | 24.48 | 11.18 | 22.93 |  |  |
| 25.20 | 18.22 | 27.81 |  |  | 24.49 | 11.16 | 22.90 |  |  |
| 25.21 | 18.20 | 27.77 |  |  | 24.52 | 11.12 | 22.78 |  |  |
| 25.22 | 18.19 | 27.74 |  |  | 24.54 | 11.10 | 22.71 |  |  |
| 25.23 | 18.17 | 27.70 |  |  | 24.55 | 11.10 | 22.67 |  |  |
| 25.24 | 18.14 | 27.66 |  |  | 24.56 | 11.08 | 22.63 |  |  |
| 25.25 | 18.11 | 27.62 |  |  | 24.58 | 11.04 | 22.55 |  |  |
| 25.26 | 18.09 | 27.58 |  |  | 24.59 | 11.02 | 22.51 |  |  |
| 25.27 | 18.07 | 27.54 |  |  | 24.60 | 11.01 | 22.48 |  |  |
| 25.29 | 18.05 | 27.47 |  |  | 24.61 | 11.00 | 22.44 |  |  |
| 25.30 | 18.03 | 27.43 |  |  | 24.62 | 10.99 | 22.40 |  |  |
| 25.31 | 18.01 | 27.39 |  |  | 24.63 | 10.97 | 22.36 |  |  |
| 25.32 | 17.98 | 27.35 |  |  | 24.64 | 10.96 | 22.32 |  |  |
| 25.33 | 17.94 | 27.31 |  |  | 24.65 | 10.93 | 22.29 |  |  |
| 25.34 | 17.91 | 27.27 |  |  | 24.66 | 10.91 | 22.25 |  |  |
| 25.36 | 17.86 | 27.20 |  |  | 24.67 | 10.89 | 22.21 |  |  |
| 25.37 | 17.83 | 27.16 |  |  | 24.68 | 10.88 | 22.17 |  |  |
| 25.38 | 17.80 | 27.12 |  |  | 24.69 | 10.86 | 22.13 |  |  |
| 25.39 | 17.78 | 27.08 |  |  | 24.70 | 10.84 | 22.10 |  |  |
| 25.40 | 17.76 | 27.04 |  |  | 24.71 | 10.83 | 22.06 |  |  |
| 25.41 | 17.73 | 27.01 |  |  | 24.72 | 10.82 | 22.02 |  |  |
| 25.42 | 17.71 | 26.97 |  |  | 24.73 | 10.81 | 21.98 |  |  |
| 25.43 | 17.69 | 26.93 |  |  | 24.74 | 10.80 | 21.94 |  |  |
| 25.44 | 17.68 | 26.89 |  |  | 24.75 | 10.77 | 21.91 |  |  |
| 25.45 | 17.66 | 26.85 |  |  | 24.76 | 10.75 | 21.87 |  |  |
| 25.46 | 17.64 | 26.81 |  |  | 24.77 | 10.73 | 21.83 |  |  |
| 25.47 | 17.62 | 26.77 |  |  | 24.78 | 10.72 | 21.79 |  |  |
| 25.48 | 17.59 | 26.74 |  |  | 24.79 | 10.70 | 21.75 |  |  |
| 25.49 | 17.57 | 26.70 |  |  | 24.80 | 10.69 | 21.72 |  |  |
| 25.50 | 17.55 | 26.66 |  |  | 24.82 | 10.65 | 21.64 |  |  |
| 25.51 | 17.53 | 26.62 |  |  | 24.83 | 10.63 | 21.60 |  |  |
| 25.52 | 17.52 | 26.58 |  |  | 24.85 | 10.61 | 21.53 |  |  |
| 25.53 | 17.50 | 26.54 |  |  | 24.86 | 10.61 | 21.49 |  |  |
| 25.54 | 17.47 | 26.51 |  |  | 24.88 | 10.57 | 21.41 |  |  |
| 25.55 | 17.45 | 26.47 |  |  | 24.89 | 10.55 | 21.37 |  |  |
| 25.56 | 17.43 | 26.43 |  |  | 24.91 | 10.51 | 21.30 |  |  |
| 25.57 | 17.40 | 26.39 |  |  | 24.92 | 10.48 | 21.26 |  |  |
| 25.58 | 17.39 | 26.35 |  |  | 24.93 | 10.46 | 21.22 |  |  |
| 25.60 | 17.34 | 26.27 |  |  | 24.94 | 10.44 | 21.18 |  |  |
| 25.61 | 17.31 | 26.24 |  |  | 24.95 | 10.42 | 21.14 |  |  |
| 25.62 | 17.30 | 26.20 |  |  | 24.96 | 10.42 | 21.11 |  |  |
| 25.63 | 17.28 | 26.16 |  |  | 24.97 | 10.41 | 21.07 |  |  |
| 25.64 | 17.26 | 26.12 |  |  | 24.98 | 10.40 | 21.03 |  |  |
| 25.65 | 17.24 | 26.08 |  |  | 24.99 | 10.38 | 20.99 |  |  |
| 25.66 | 17.21 | 26.04 |  |  | 25.00 | 10.36 | 20.95 |  |  |
| 25.67 | 17.20 | 26.01 |  |  | 25.01 | 10.35 | 20.92 |  |  |
| 25.68 | 17.19 | 25.97 |  |  | 25.02 | 10.33 | 20.88 |  |  |
| 25.70 | 17.14 | 25.89 |  |  | 25.03 | 10.31 | 20.84 |  |  |
| 25.71 | 17.10 | 25.85 |  |  | 25.04 | 10.30 | 20.80 |  |  |
| 25.72 | 17.09 | 25.81 |  |  | 25.05 | 10.28 | 20.76 |  |  |
| 25.74 | 17.05 | 25.74 |  |  | 25.06 | 10.27 | 20.73 |  |  |
| 25.76 | 17.01 | 25.66 |  |  | 25.08 | 10.25 | 20.65 |  |  |
| 25.77 | 17.00 | 25.62 |  |  | 25.09 | 10.22 | 20.61 |  |  |
| 25.78 | 17.00 | 25.58 |  |  | 25.10 | 10.21 | 20.57 |  |  |
| 25.79 | 16.97 | 25.54 |  |  | 25.11 | 10.19 | 20.54 |  |  |
| 25.80 | 16.93 | 25.51 |  |  | 25.12 | 10.18 | 20.50 |  |  |
| 25.82 | 16.91 | 25.43 |  |  | 25.13 | 10.15 | 20.46 |  |  |
| 25.83 | 16.88 | 25.39 |  |  | 25.16 | 10.10 | 20.35 |  |  |
| 25.84 | 16.87 | 25.35 |  |  | 25.17 | 10.09 | 20.31 |  |  |
| 25.85 | 16.84 | 25.31 |  |  | 25.18 | 10.07 | 20.27 |  |  |
| 25.86 | 16.83 | 25.27 |  |  | 25.19 | 10.05 | 20.23 |  |  |
| 25.87 | 16.80 | 25.24 |  |  | 25.20 | 10.04 | 20.19 |  |  |
| 25.88 | 16.78 | 25.20 |  |  | 25.21 | 10.02 | 20.15 |  |  |
| 25.89 | 16.76 | 25.16 |  |  | 25.22 | 10.00 | 20.12 |  |  |
| 25.90 | 16.74 | 25.12 |  |  | 25.23 | 9.99 | 20.08 |  |  |
| 25.91 | 16.72 | 25.08 |  |  | 25.24 | 9.97 | 20.04 |  |  |
| 25.92 | 16.71 | 25.04 |  |  | 25.25 | 9.95 | 20.00 |  |  |
| 25.93 | 16.69 | 25.01 |  |  | 25.26 | 9.95 | 19.96 |  |  |
| 25.94 | 16.68 | 24.97 |  |  | 25.28 | 9.93 | 19.89 |  |  |
| 25.95 | 16.67 | 24.93 |  |  | 25.29 | 9.91 | 19.85 |  |  |
| 25.96 | 16.65 | 24.89 |  |  | 25.31 | 9.89 | 19.77 |  |  |
| 25.97 | 16.64 | 24.85 |  |  | 25.33 | 9.86 | 19.70 |  |  |
| 25.99 | 16.60 | 24.77 |  |  | 25.34 | 9.85 | 19.66 |  |  |
| 26.00 | 16.58 | 24.74 |  |  | 25.35 | 9.84 | 19.62 |  |  |
| 26.01 | 16.56 | 24.70 |  |  | 25.37 | 9.82 | 19.55 |  |  |
| 26.02 | 16.53 | 24.66 |  |  | 25.38 | 9.81 | 19.51 |  |  |
| 26.03 | 16.51 | 24.62 |  |  | 25.39 | 9.80 | 19.47 |  |  |
| 26.04 | 16.49 | 24.58 |  |  | 25.40 | 9.79 | 19.43 |  |  |
| 26.05 | 16.47 | 24.54 |  |  | 25.41 | 9.76 | 19.39 |  |  |
| 26.06 | 16.44 | 24.51 |  |  | 25.42 | 9.75 | 19.36 |  |  |
| 26.07 | 16.43 | 24.47 |  |  | 25.43 | 9.73 | 19.32 |  |  |
| 26.08 | 16.41 | 24.43 |  |  | 25.44 | 9.71 | 19.28 |  |  |
| 26.09 | 16.39 | 24.39 |  |  | 25.45 | 9.69 | 19.24 |  |  |
| 26.10 | 16.37 | 24.35 |  |  | 25.46 | 9.68 | 19.20 |  |  |
| 26.11 | 16.35 | 24.31 |  |  | 25.47 | 9.67 | 19.17 |  |  |
| 26.13 | 16.29 | 24.24 |  |  | 25.48 | 9.67 | 19.13 |  |  |
| 26.14 | 16.25 | 24.20 |  |  | 25.49 | 9.66 | 19.09 |  |  |
| 26.16 | 16.22 | 24.12 |  |  | 25.50 | 9.65 | 19.05 |  |  |
| 26.18 | 16.18 | 24.04 |  |  | 25.51 | 9.65 | 19.01 |  |  |
| 26.19 | 16.16 | 24.00 |  |  | 25.52 | 9.63 | 18.98 |  |  |
| 26.20 | 16.13 | 23.97 |  |  | 25.54 | 9.62 | 18.90 |  |  |
| 26.21 | 16.12 | 23.93 |  |  | 25.55 | 9.61 | 18.86 |  |  |
| 26.22 | 16.09 | 23.89 |  |  | 25.56 | 9.59 | 18.82 |  |  |
| 26.23 | 16.07 | 23.85 |  |  | 25.57 | 9.59 | 18.78 |  |  |
| 26.24 | 16.05 | 23.81 |  |  | 25.58 | 9.58 | 18.75 |  |  |
| 26.25 | 16.02 | 23.77 |  |  | 25.59 | 9.57 | 18.71 |  |  |
| 26.26 | 16.00 | 23.74 |  |  | 25.60 | 9.56 | 18.67 |  |  |
| 26.27 | 15.98 | 23.70 |  |  | 25.61 | 9.55 | 18.63 |  |  |
| 26.28 | 15.94 | 23.66 |  |  | 25.62 | 9.54 | 18.59 |  |  |
| 26.29 | 15.93 | 23.62 |  |  | 25.63 | 9.53 | 18.56 |  |  |
| 26.30 | 15.90 | 23.58 |  |  | 25.65 | 9.52 | 18.48 |  |  |
| 26.31 | 15.88 | 23.54 |  |  | 25.66 | 9.52 | 18.44 |  |  |
| 26.32 | 15.86 | 23.50 |  |  | 25.67 | 9.51 | 18.40 |  |  |
| 26.33 | 15.85 | 23.47 |  |  | 25.68 | 9.50 | 18.37 |  |  |
| 26.34 | 15.82 | 23.43 |  |  | 25.69 | 9.50 | 18.33 |  |  |
| 26.35 | 15.80 | 23.39 |  |  | 25.70 | 9.49 | 18.29 |  |  |
| 26.36 | 15.78 | 23.35 |  |  | 25.71 | 9.49 | 18.25 |  |  |
| 26.37 | 15.76 | 23.31 |  |  | 25.72 | 9.48 | 18.21 |  |  |
| 26.38 | 15.74 | 23.27 |  |  | 25.73 | 9.47 | 18.18 |  |  |
| 26.39 | 15.73 | 23.24 |  |  | 25.74 | 9.47 | 18.14 |  |  |
| 26.40 | 15.70 | 23.20 |  |  | 25.75 | 9.46 | 18.10 |  |  |
| 26.41 | 15.69 | 23.16 |  |  | 25.76 | 9.46 | 18.06 |  |  |
| 26.42 | 15.67 | 23.12 |  |  | 25.77 | 9.45 | 18.02 |  |  |
| 26.43 | 15.65 | 23.08 |  |  | 25.79 | 9.43 | 17.95 |  |  |
| 26.44 | 15.62 | 23.04 |  |  | 25.80 | 9.42 | 17.91 |  |  |
| 26.45 | 15.59 | 23.00 |  |  | 25.81 | 9.42 | 17.87 |  |  |
| 26.46 | 15.57 | 22.97 |  |  | 25.82 | 9.42 | 17.83 |  |  |
| 26.48 | 15.53 | 22.89 |  |  | 25.83 | 9.41 | 17.80 |  |  |
| 26.49 | 15.51 | 22.85 |  |  | 25.84 | 9.41 | 17.76 |  |  |
| 26.50 | 15.48 | 22.81 |  |  | 25.85 | 9.40 | 17.72 |  |  |
| 26.51 | 15.48 | 22.77 |  |  | 25.86 | 9.39 | 17.68 |  |  |
| 26.52 | 15.46 | 22.74 |  |  | 25.87 | 9.38 | 17.64 |  |  |
| 26.53 | 15.42 | 22.70 |  |  | 25.88 | 9.36 | 17.60 |  |  |
| 26.54 | 15.39 | 22.66 |  |  | 25.89 | 9.35 | 17.57 |  |  |
| 26.55 | 15.37 | 22.62 |  |  | 25.90 | 9.35 | 17.53 |  |  |
| 26.56 | 15.36 | 22.58 |  |  | 25.91 | 9.34 | 17.49 |  |  |
| 26.57 | 15.33 | 22.54 |  |  | 25.92 | 9.33 | 17.45 |  |  |
| 26.58 | 15.31 | 22.50 |  |  | 25.93 | 9.32 | 17.41 |  |  |
| 26.59 | 15.29 | 22.47 |  |  | 25.95 | 9.30 | 17.34 |  |  |
| 26.60 | 15.26 | 22.43 |  |  | 25.96 | 9.29 | 17.30 |  |  |
| 26.61 | 15.23 | 22.39 |  |  | 25.97 | 9.28 | 17.26 |  |  |
| 26.62 | 15.22 | 22.35 |  |  | 25.98 | 9.28 | 17.22 |  |  |
| 26.63 | 15.20 | 22.31 |  |  | 25.99 | 9.27 | 17.19 |  |  |
| 26.64 | 15.16 | 22.27 |  |  | 26.00 | 9.26 | 17.15 |  |  |
| 26.65 | 15.13 | 22.24 |  |  | 26.02 | 9.25 | 17.07 |  |  |
| 26.66 | 15.12 | 22.20 |  |  | 26.03 | 9.25 | 17.03 |  |  |
| 26.67 | 15.09 | 22.16 |  |  | 26.04 | 9.24 | 17.00 |  |  |
| 26.68 | 15.07 | 22.12 |  |  | 26.05 | 9.23 | 16.96 |  |  |
| 26.69 | 15.05 | 22.08 |  |  | 26.06 | 9.22 | 16.92 |  |  |
| 26.70 | 15.03 | 22.04 |  |  | 26.09 | 9.19 | 16.81 |  |  |
| 26.71 | 15.02 | 22.00 |  |  | 26.10 | 9.19 | 16.77 |  |  |
| 26.72 | 15.00 | 21.97 |  |  | 26.11 | 9.17 | 16.73 |  |  |
| 26.73 | 14.97 | 21.93 |  |  | 26.12 | 9.16 | 16.69 |  |  |
| 26.74 | 14.97 | 21.89 |  |  | 26.13 | 9.13 | 16.65 |  |  |
| 26.75 | 14.94 | 21.85 |  |  | 26.14 | 9.12 | 16.62 |  |  |
| 26.77 | 14.89 | 21.77 |  |  | 26.15 | 9.11 | 16.58 |  |  |
| 26.78 | 14.86 | 21.74 |  |  | 26.18 | 9.08 | 16.46 |  |  |
| 26.80 | 14.82 | 21.66 |  |  | 26.19 | 9.07 | 16.42 |  |  |
| 26.81 | 14.80 | 21.62 |  |  | 26.20 | 9.06 | 16.39 |  |  |
| 26.82 | 14.78 | 21.58 |  |  | 26.24 | 9.02 | 16.23 |  |  |
| 26.83 | 14.75 | 21.54 |  |  | 26.25 | 9.01 | 16.20 |  |  |
| 26.84 | 14.73 | 21.50 |  |  | 26.26 | 9.01 | 16.16 |  |  |
| 26.85 | 14.71 | 21.47 |  |  | 26.27 | 8.99 | 16.12 |  |  |
| 26.86 | 14.68 | 21.43 |  |  | 26.28 | 8.99 | 16.08 |  |  |
| 26.87 | 14.67 | 21.39 |  |  | 26.29 | 8.97 | 16.04 |  |  |
| 26.88 | 14.66 | 21.35 |  |  | 26.30 | 8.95 | 16.01 |  |  |
| 26.89 | 14.64 | 21.31 |  |  | 26.31 | 8.94 | 15.97 |  |  |
| 26.90 | 14.61 | 21.27 |  |  | 26.33 | 8.92 | 15.89 |  |  |
| 26.91 | 14.59 | 21.24 |  |  | 26.35 | 8.88 | 15.82 |  |  |
| 26.92 | 14.58 | 21.20 |  |  | 26.36 | 8.87 | 15.78 |  |  |
| 26.93 | 14.57 | 21.16 |  |  | 26.38 | 8.84 | 15.70 |  |  |
| 26.94 | 14.55 | 21.12 |  |  | 26.41 | 8.81 | 15.59 |  |  |
| 26.95 | 14.53 | 21.08 |  |  | 26.42 | 8.79 | 15.55 |  |  |
| 26.96 | 14.51 | 21.04 |  |  | 26.43 | 8.77 | 15.51 |  |  |
| 26.97 | 14.49 | 21.00 |  |  | 26.44 | 8.77 | 15.47 |  |  |
| 26.99 | 14.44 | 20.93 |  |  | 26.45 | 8.76 | 15.44 |  |  |
| 27.00 | 14.43 | 20.89 |  |  | 26.47 | 8.74 | 15.36 |  |  |
| 27.01 | 14.40 | 20.85 |  |  | 26.48 | 8.72 | 15.32 |  |  |
| 27.02 | 14.39 | 20.81 |  |  | 26.49 | 8.71 | 15.28 |  |  |
| 27.03 | 14.36 | 20.77 |  |  | 26.50 | 8.69 | 15.25 |  |  |
| 27.04 | 14.34 | 20.73 |  |  | 26.51 | 8.69 | 15.21 |  |  |
| 27.05 | 14.32 | 20.70 |  |  | 26.52 | 8.67 | 15.17 |  |  |
| 27.08 | 14.26 | 20.58 |  |  | 26.53 | 8.66 | 15.13 |  |  |
| 27.09 | 14.22 | 20.54 |  |  | 26.54 | 8.65 | 15.09 |  |  |
| 27.10 | 14.21 | 20.50 |  |  | 26.55 | 8.64 | 15.05 |  |  |
| 27.11 | 14.19 | 20.47 |  |  | 26.56 | 8.63 | 15.02 |  |  |
| 27.12 | 14.17 | 20.43 |  |  | 26.57 | 8.62 | 14.98 |  |  |
| 27.13 | 14.15 | 20.39 |  |  | 26.58 | 8.60 | 14.94 |  |  |
| 27.14 | 14.15 | 20.35 |  |  | 26.59 | 8.59 | 14.90 |  |  |
| 27.15 | 14.12 | 20.31 |  |  | 26.60 | 8.57 | 14.86 |  |  |
| 27.16 | 14.10 | 20.27 |  |  | 26.61 | 8.57 | 14.83 |  |  |
| 27.17 | 14.08 | 20.23 |  |  | 26.62 | 8.55 | 14.79 |  |  |
| 27.18 | 14.06 | 20.20 |  |  | 26.64 | 8.53 | 14.71 |  |  |
| 27.19 | 14.03 | 20.16 |  |  | 26.65 | 8.52 | 14.67 |  |  |
| 27.21 | 13.98 | 20.08 |  |  | 26.67 | 8.51 | 14.60 |  |  |
| 27.22 | 13.97 | 20.04 |  |  | 26.68 | 8.49 | 14.56 |  |  |
| 27.24 | 13.95 | 19.97 |  |  | 26.70 | 8.47 | 14.48 |  |  |
| 27.25 | 13.93 | 19.93 |  |  | 26.71 | 8.45 | 14.45 |  |  |
| 27.26 | 13.91 | 19.89 |  |  | 26.72 | 8.44 | 14.41 |  |  |
| 27.27 | 13.90 | 19.85 |  |  | 26.73 | 8.43 | 14.37 |  |  |
| 27.28 | 13.88 | 19.81 |  |  | 26.75 | 8.41 | 14.29 |  |  |
| 27.29 | 13.87 | 19.77 |  |  | 26.76 | 8.41 | 14.26 |  |  |
| 27.30 | 13.85 | 19.73 |  |  | 26.78 | 8.39 | 14.18 |  |  |
| 27.31 | 13.83 | 19.70 |  |  | 26.79 | 8.39 | 14.14 |  |  |
| 27.32 | 13.80 | 19.66 |  |  | 26.80 | 8.37 | 14.10 |  |  |
| 27.34 | 13.78 | 19.58 |  |  | 26.82 | 8.35 | 14.03 |  |  |
| 27.35 | 13.76 | 19.54 |  |  | 26.83 | 8.33 | 13.99 |  |  |
| 27.36 | 13.74 | 19.50 |  |  | 26.85 | 8.31 | 13.91 |  |  |
| 27.37 | 13.73 | 19.47 |  |  | 26.87 | 8.30 | 13.84 |  |  |
| 27.38 | 13.70 | 19.43 |  |  | 26.88 | 8.28 | 13.80 |  |  |
| 27.39 | 13.68 | 19.39 |  |  | 26.90 | 8.26 | 13.72 |  |  |
| 27.40 | 13.67 | 19.35 |  |  | 26.91 | 8.25 | 13.68 |  |  |
| 27.41 | 13.66 | 19.31 |  |  | 26.92 | 8.24 | 13.65 |  |  |
| 27.42 | 13.65 | 19.27 |  |  | 26.93 | 8.23 | 13.61 |  |  |
| 27.44 | 13.61 | 19.20 |  |  | 26.94 | 8.23 | 13.57 |  |  |
| 27.45 | 13.60 | 19.16 |  |  | 26.97 | 8.20 | 13.46 |  |  |
| 27.47 | 13.56 | 19.08 |  |  | 26.98 | 8.20 | 13.42 |  |  |
| 27.48 | 13.54 | 19.04 |  |  | 27.00 | 8.18 | 13.34 |  |  |
| 27.49 | 13.52 | 19.00 |  |  | 27.02 | 8.17 | 13.27 |  |  |
| 27.50 | 13.51 | 18.97 |  |  | 27.03 | 8.16 | 13.23 |  |  |
| 27.51 | 13.48 | 18.93 |  |  | 27.04 | 8.15 | 13.19 |  |  |
| 27.52 | 13.47 | 18.89 |  |  | 27.05 | 8.13 | 13.15 |  |  |
| 27.53 | 13.46 | 18.85 |  |  | 27.06 | 8.11 | 13.11 |  |  |
| 27.54 | 13.44 | 18.81 |  |  | 27.07 | 8.10 | 13.08 |  |  |
| 27.55 | 13.43 | 18.77 |  |  | 27.08 | 8.09 | 13.04 |  |  |
| 27.56 | 13.41 | 18.73 |  |  | 27.09 | 8.09 | 13.00 |  |  |
| 27.57 | 13.39 | 18.70 |  |  | 27.10 | 8.08 | 12.96 |  |  |
| 27.58 | 13.38 | 18.66 |  |  | 27.11 | 8.08 | 12.92 |  |  |
| 27.59 | 13.36 | 18.62 |  |  | 27.13 | 8.06 | 12.85 |  |  |
| 27.61 | 13.32 | 18.54 |  |  | 27.14 | 8.06 | 12.81 |  |  |
| 27.62 | 13.30 | 18.50 |  |  | 27.15 | 8.04 | 12.77 |  |  |
| 27.63 | 13.28 | 18.47 |  |  | 27.16 | 8.03 | 12.73 |  |  |
| 27.64 | 13.26 | 18.43 |  |  | 27.17 | 8.02 | 12.69 |  |  |
| 27.65 | 13.24 | 18.39 |  |  | 27.18 | 8.01 | 12.66 |  |  |
| 27.66 | 13.21 | 18.35 |  |  | 27.19 | 8.00 | 12.62 |  |  |
| 27.67 | 13.20 | 18.31 |  |  | 27.20 | 7.98 | 12.58 |  |  |
| 27.68 | 13.19 | 18.27 |  |  | 27.21 | 7.98 | 12.54 |  |  |
| 27.69 | 13.17 | 18.23 |  |  | 27.22 | 7.97 | 12.50 |  |  |
| 27.70 | 13.16 | 18.20 |  |  | 27.23 | 7.96 | 12.47 |  |  |
| 27.71 | 13.12 | 18.16 |  |  | 27.24 | 7.95 | 12.43 |  |  |
| 27.72 | 13.11 | 18.12 |  |  | 27.26 | 7.94 | 12.35 |  |  |
| 27.73 | 13.09 | 18.08 |  |  | 27.27 | 7.93 | 12.31 |  |  |
| 27.74 | 13.08 | 18.04 |  |  | 27.28 | 7.92 | 12.28 |  |  |
| 27.76 | 13.05 | 17.97 |  |  | 27.30 | 7.90 | 12.20 |  |  |
| 27.77 | 13.04 | 17.93 |  |  | 27.32 | 7.88 | 12.12 |  |  |
| 27.79 | 12.99 | 17.85 |  |  | 27.33 | 7.88 | 12.09 |  |  |
| 27.82 | 12.95 | 17.73 |  |  | 27.34 | 7.87 | 12.05 |  |  |
| 27.83 | 12.93 | 17.70 |  |  | 27.35 | 7.85 | 12.01 |  |  |
| 27.84 | 12.90 | 17.66 |  |  | 27.37 | 7.84 | 11.93 |  |  |
| 27.85 | 12.89 | 17.62 |  |  | 27.38 | 7.83 | 11.90 |  |  |
| 27.86 | 12.86 | 17.58 |  |  | 27.39 | 7.82 | 11.86 |  |  |
| 27.87 | 12.84 | 17.54 |  |  | 27.40 | 7.82 | 11.82 |  |  |
| 27.88 | 12.82 | 17.50 |  |  | 27.41 | 7.80 | 11.78 |  |  |
| 27.89 | 12.80 | 17.46 |  |  | 27.42 | 7.78 | 11.74 |  |  |
| 27.90 | 12.78 | 17.43 |  |  | 27.44 | 7.76 | 11.67 |  |  |
| 27.91 | 12.74 | 17.39 |  |  | 27.45 | 7.75 | 11.63 |  |  |
| 27.92 | 12.71 | 17.35 |  |  | 27.46 | 7.74 | 11.59 |  |  |
| 27.93 | 12.69 | 17.31 |  |  | 27.47 | 7.72 | 11.55 |  |  |
| 27.94 | 12.67 | 17.27 |  |  | 27.48 | 7.72 | 11.52 |  |  |
| 27.95 | 12.67 | 17.23 |  |  | 27.49 | 7.71 | 11.48 |  |  |
| 27.96 | 12.64 | 17.20 |  |  | 27.50 | 7.70 | 11.44 |  |  |
| 27.97 | 12.61 | 17.16 |  |  | 27.51 | 7.70 | 11.40 |  |  |
| 27.98 | 12.57 | 17.12 |  |  | 27.53 | 7.66 | 11.32 |  |  |
| 27.99 | 12.56 | 17.08 |  |  | 27.54 | 7.65 | 11.29 |  |  |
| 28.00 | 12.54 | 17.04 |  |  | 27.55 | 7.63 | 11.25 |  |  |
| 28.01 | 12.51 | 17.00 |  |  | 27.56 | 7.63 | 11.21 |  |  |
| 28.02 | 12.48 | 16.96 |  |  | 27.57 | 7.62 | 11.17 |  |  |
| 28.03 | 12.47 | 16.93 |  |  | 27.58 | 7.61 | 11.13 |  |  |
| 28.04 | 12.44 | 16.89 |  |  | 27.59 | 7.60 | 11.10 |  |  |
| 28.05 | 12.41 | 16.85 |  |  | 27.60 | 7.59 | 11.06 |  |  |
| 28.06 | 12.39 | 16.81 |  |  | 27.61 | 7.59 | 11.02 |  |  |
| 28.07 | 12.37 | 16.77 |  |  | 27.62 | 7.58 | 10.98 |  |  |
| 28.08 | 12.35 | 16.73 |  |  | 27.63 | 7.57 | 10.94 |  |  |
| 28.09 | 12.34 | 16.70 |  |  | 27.64 | 7.57 | 10.91 |  |  |
| 28.10 | 12.32 | 16.66 |  |  | 27.65 | 7.56 | 10.87 |  |  |
| 28.11 | 12.29 | 16.62 |  |  | 27.66 | 7.55 | 10.83 |  |  |
| 28.12 | 12.27 | 16.58 |  |  | 27.67 | 7.54 | 10.79 |  |  |
| 28.13 | 12.26 | 16.54 |  |  | 27.69 | 7.53 | 10.72 |  |  |
| 28.15 | 12.22 | 16.46 |  |  | 27.70 | 7.51 | 10.68 |  |  |
| 28.16 | 12.20 | 16.43 |  |  | 27.71 | 7.50 | 10.64 |  |  |
| 28.17 | 12.18 | 16.39 |  |  | 27.72 | 7.48 | 10.60 |  |  |
| 28.18 | 12.17 | 16.35 |  |  | 27.73 | 7.47 | 10.56 |  |  |
| 28.19 | 12.15 | 16.31 |  |  | 27.74 | 7.47 | 10.53 |  |  |
| 28.20 | 12.14 | 16.27 |  |  | 27.75 | 7.46 | 10.49 |  |  |
| 28.21 | 12.13 | 16.23 |  |  | 27.76 | 7.46 | 10.45 |  |  |
| 28.22 | 12.11 | 16.20 |  |  | 27.77 | 7.45 | 10.41 |  |  |
| 28.23 | 12.09 | 16.16 |  |  | 27.78 | 7.45 | 10.37 |  |  |
| 28.24 | 12.08 | 16.12 |  |  | 27.79 | 7.44 | 10.34 |  |  |
| 28.25 | 12.07 | 16.08 |  |  | 27.80 | 7.43 | 10.30 |  |  |
| 28.26 | 12.06 | 16.04 |  |  | 27.81 | 7.43 | 10.26 |  |  |
| 28.27 | 12.02 | 16.00 |  |  | 27.82 | 7.43 | 10.22 |  |  |
| 28.28 | 11.99 | 15.96 |  |  | 27.83 | 7.42 | 10.18 |  |  |
| 28.29 | 11.98 | 15.93 |  |  | 27.84 | 7.40 | 10.14 |  |  |
| 28.30 | 11.97 | 15.89 |  |  | 27.85 | 7.38 | 10.11 |  |  |
| 28.32 | 11.92 | 15.81 |  |  | 27.86 | 7.37 | 10.07 |  |  |
| 28.33 | 11.89 | 15.77 |  |  | 27.87 | 7.36 | 10.03 |  |  |
| 28.34 | 11.88 | 15.73 |  |  | 27.88 | 7.34 | 9.99 |  |  |
| 28.35 | 11.86 | 15.70 |  |  | 27.89 | 7.33 | 9.95 |  |  |
| 28.36 | 11.83 | 15.66 |  |  | 27.90 | 7.31 | 9.92 |  |  |
| 28.37 | 11.82 | 15.62 |  |  | 27.92 | 7.30 | 9.84 |  |  |
| 28.38 | 11.81 | 15.58 |  |  | 27.93 | 7.29 | 9.80 |  |  |
| 28.39 | 11.80 | 15.54 |  |  | 27.94 | 7.28 | 9.76 |  |  |
| 28.40 | 11.79 | 15.50 |  |  | 27.95 | 7.28 | 9.73 |  |  |
| 28.41 | 11.77 | 15.46 |  |  | 27.98 | 7.25 | 9.61 |  |  |
| 28.42 | 11.76 | 15.43 |  |  | 27.99 | 7.24 | 9.57 |  |  |
| 28.43 | 11.75 | 15.39 |  |  | 28.00 | 7.23 | 9.54 |  |  |
| 28.44 | 11.72 | 15.35 |  |  | 28.01 | 7.22 | 9.50 |  |  |
| 28.45 | 11.71 | 15.31 |  |  | 28.02 | 7.21 | 9.46 |  |  |
| 28.46 | 11.69 | 15.27 |  |  | 28.03 | 7.21 | 9.42 |  |  |
| 28.47 | 11.67 | 15.23 |  |  | 28.04 | 7.20 | 9.38 |  |  |
| 28.48 | 11.65 | 15.20 |  |  | 28.05 | 7.19 | 9.35 |  |  |
| 28.49 | 11.63 | 15.16 |  |  | 28.07 | 7.17 | 9.27 |  |  |
| 28.50 | 11.60 | 15.12 |  |  | 28.08 | 7.16 | 9.23 |  |  |
| 28.51 | 11.59 | 15.08 |  |  | 28.10 | 7.14 | 9.16 |  |  |
| 28.52 | 11.55 | 15.04 |  |  | 28.11 | 7.13 | 9.12 |  |  |
| 28.53 | 11.53 | 15.00 |  |  | 28.12 | 7.11 | 9.08 |  |  |
| 28.54 | 11.52 | 14.96 |  |  | 28.13 | 7.10 | 9.04 |  |  |
| 28.55 | 11.49 | 14.93 |  |  | 28.15 | 7.10 | 8.96 |  |  |
| 28.56 | 11.47 | 14.89 |  |  | 28.16 | 7.08 | 8.93 |  |  |
| 28.57 | 11.45 | 14.85 |  |  | 28.18 | 7.07 | 8.85 |  |  |
| 28.58 | 11.41 | 14.81 |  |  | 28.19 | 7.05 | 8.81 |  |  |
| 28.59 | 11.40 | 14.77 |  |  | 28.20 | 7.03 | 8.77 |  |  |
| 28.60 | 11.39 | 14.73 |  |  | 28.21 | 7.02 | 8.74 |  |  |
| 28.61 | 11.37 | 14.70 |  |  | 28.23 | 7.00 | 8.66 |  |  |
| 28.62 | 11.35 | 14.66 |  |  | 28.24 | 7.00 | 8.62 |  |  |
| 28.63 | 11.33 | 14.62 |  |  | 28.25 | 6.99 | 8.58 |  |  |
| 28.64 | 11.31 | 14.58 |  |  | 28.26 | 6.98 | 8.55 |  |  |
| 28.65 | 11.28 | 14.54 |  |  | 28.27 | 6.97 | 8.51 |  |  |
| 28.66 | 11.27 | 14.50 |  |  | 28.29 | 6.94 | 8.43 |  |  |
| 28.67 | 11.25 | 14.46 |  |  | 28.30 | 6.94 | 8.39 |  |  |
| 28.68 | 11.23 | 14.43 |  |  | 28.31 | 6.93 | 8.36 |  |  |
| 28.69 | 11.21 | 14.39 |  |  | 28.32 | 6.92 | 8.32 |  |  |
| 28.70 | 11.20 | 14.35 |  |  | 28.33 | 6.91 | 8.28 |  |  |
| 28.71 | 11.18 | 14.31 |  |  | 28.34 | 6.89 | 8.24 |  |  |
| 28.72 | 11.16 | 14.27 |  |  | 28.35 | 6.88 | 8.20 |  |  |
| 28.73 | 11.15 | 14.23 |  |  | 28.36 | 6.86 | 8.17 |  |  |
| 28.74 | 11.13 | 14.19 |  |  | 28.37 | 6.84 | 8.13 |  |  |
| 28.75 | 11.11 | 14.16 |  |  | 28.38 | 6.83 | 8.09 |  |  |
| 28.78 | 11.07 | 14.04 |  |  | 28.40 | 6.81 | 8.01 |  |  |
| 28.79 | 11.04 | 14.00 |  |  | 28.41 | 6.80 | 7.98 |  |  |
| 28.80 | 11.04 | 13.96 |  |  | 28.42 | 6.79 | 7.94 |  |  |
| 28.81 | 11.02 | 13.93 |  |  | 28.43 | 6.79 | 7.90 |  |  |
| 28.82 | 11.00 | 13.89 |  |  | 28.44 | 6.78 | 7.86 |  |  |
| 28.83 | 10.98 | 13.85 |  |  | 28.45 | 6.76 | 7.82 |  |  |
| 28.84 | 10.96 | 13.81 |  |  | 28.46 | 6.75 | 7.79 |  |  |
| 28.85 | 10.94 | 13.77 |  |  | 28.49 | 6.72 | 7.67 |  |  |
| 28.86 | 10.93 | 13.73 |  |  | 28.50 | 6.70 | 7.63 |  |  |
| 28.87 | 10.91 | 13.69 |  |  | 28.51 | 6.69 | 7.59 |  |  |
| 28.88 | 10.89 | 13.66 |  |  | 28.53 | 6.67 | 7.52 |  |  |
| 28.89 | 10.89 | 13.62 |  |  | 28.54 | 6.65 | 7.48 |  |  |
| 28.90 | 10.86 | 13.58 |  |  | 28.55 | 6.63 | 7.44 |  |  |
| 28.91 | 10.84 | 13.54 |  |  | 28.56 | 6.62 | 7.40 |  |  |
| 28.92 | 10.82 | 13.50 |  |  | 28.57 | 6.61 | 7.37 |  |  |
| 28.93 | 10.80 | 13.46 |  |  | 28.58 | 6.59 | 7.33 |  |  |
| 28.94 | 10.79 | 13.43 |  |  | 28.62 | 6.56 | 7.18 |  |  |
| 28.95 | 10.76 | 13.39 |  |  | 28.63 | 6.55 | 7.14 |  |  |
| 28.96 | 10.75 | 13.35 |  |  | 28.64 | 6.55 | 7.10 |  |  |
| 28.97 | 10.73 | 13.31 |  |  | 28.66 | 6.51 | 7.02 |  |  |
| 28.98 | 10.71 | 13.27 |  |  | 28.67 | 6.50 | 6.99 |  |  |
| 28.99 | 10.68 | 13.23 |  |  | 28.68 | 6.49 | 6.95 |  |  |
| 29.00 | 10.67 | 13.19 |  |  | 28.69 | 6.48 | 6.91 |  |  |
| 29.01 | 10.66 | 13.16 |  |  | 28.70 | 6.46 | 6.87 |  |  |
| 29.03 | 10.62 | 13.08 |  |  | 28.71 | 6.45 | 6.83 |  |  |
| 29.04 | 10.60 | 13.04 |  |  | 28.72 | 6.43 | 6.80 |  |  |
| 29.05 | 10.60 | 13.00 |  |  | 28.73 | 6.42 | 6.76 |  |  |
| 29.06 | 10.57 | 12.96 |  |  | 28.75 | 6.40 | 6.68 |  |  |
| 29.09 | 10.53 | 12.85 |  |  | 28.76 | 6.39 | 6.64 |  |  |
| 29.10 | 10.51 | 12.81 |  |  | 28.77 | 6.39 | 6.61 |  |  |
| 29.11 | 10.49 | 12.77 |  |  | 28.78 | 6.38 | 6.57 |  |  |
| 29.15 | 10.43 | 12.62 |  |  | 28.79 | 6.36 | 6.53 |  |  |
| 29.16 | 10.42 | 12.58 |  |  | 28.80 | 6.35 | 6.49 |  |  |
| 29.17 | 10.40 | 12.54 |  |  | 28.81 | 6.35 | 6.45 |  |  |
| 29.18 | 10.37 | 12.50 |  |  | 28.82 | 6.33 | 6.41 |  |  |
| 29.19 | 10.34 | 12.46 |  |  | 28.83 | 6.33 | 6.38 |  |  |
| 29.20 | 10.31 | 12.43 |  |  | 28.84 | 6.31 | 6.34 |  |  |
| 29.21 | 10.29 | 12.39 |  |  | 28.85 | 6.30 | 6.30 |  |  |
| 29.22 | 10.27 | 12.35 |  |  | 28.86 | 6.28 | 6.26 |  |  |
| 29.23 | 10.26 | 12.31 |  |  | 28.87 | 6.27 | 6.22 |  |  |
| 29.24 | 10.23 | 12.27 |  |  | 28.88 | 6.26 | 6.19 |  |  |
| 29.26 | 10.21 | 12.19 |  |  | 28.89 | 6.25 | 6.15 |  |  |
| 29.27 | 10.19 | 12.16 |  |  | 28.90 | 6.24 | 6.11 |  |  |
| 29.28 | 10.18 | 12.12 |  |  | 28.91 | 6.22 | 6.07 |  |  |
| 29.29 | 10.15 | 12.08 |  |  | 28.92 | 6.21 | 6.03 |  |  |
| 29.30 | 10.14 | 12.04 |  |  | 28.93 | 6.20 | 6.00 |  |  |
| 29.31 | 10.12 | 12.00 |  |  | 28.94 | 6.19 | 5.96 |  |  |
| 29.32 | 10.10 | 11.96 |  |  | 28.95 | 6.18 | 5.92 |  |  |
| 29.33 | 10.08 | 11.93 |  |  | 28.96 | 6.17 | 5.88 |  |  |
| 29.35 | 10.05 | 11.85 |  |  | 28.97 | 6.16 | 5.84 |  |  |
| 29.36 | 10.02 | 11.81 |  |  | 28.98 | 6.16 | 5.81 |  |  |
| 29.37 | 10.00 | 11.77 |  |  | 28.99 | 6.14 | 5.77 |  |  |
| 29.38 | 9.99 | 11.73 |  |  | 29.01 | 6.13 | 5.69 |  |  |
| 29.39 | 9.96 | 11.69 |  |  | 29.02 | 6.11 | 5.65 |  |  |
| 29.40 | 9.95 | 11.66 |  |  | 29.04 | 6.10 | 5.58 |  |  |
| 29.41 | 9.93 | 11.62 |  |  | 29.07 | 6.09 | 5.46 |  |  |
| 29.42 | 9.92 | 11.58 |  |  | 29.09 | 6.07 | 5.39 |  |  |
| 29.44 | 9.89 | 11.50 |  |  | 29.10 | 6.06 | 5.35 |  |  |
| 29.45 | 9.86 | 11.46 |  |  | 29.11 | 6.05 | 5.31 |  |  |
| 29.46 | 9.84 | 11.43 |  |  | 29.12 | 6.04 | 5.27 |  |  |
| 29.47 | 9.81 | 11.39 |  |  | 29.13 | 6.03 | 5.23 |  |  |
| 29.48 | 9.80 | 11.35 |  |  | 29.14 | 6.03 | 5.20 |  |  |
| 29.49 | 9.77 | 11.31 |  |  | 29.15 | 6.02 | 5.16 |  |  |
| 29.50 | 9.75 | 11.27 |  |  | 29.17 | 6.01 | 5.08 |  |  |
| 29.51 | 9.72 | 11.23 |  |  | 29.18 | 5.99 | 5.04 |  |  |
| 29.52 | 9.70 | 11.19 |  |  | 29.19 | 5.99 | 5.01 |  |  |
| 29.53 | 9.69 | 11.16 |  |  | 29.20 | 5.98 | 4.97 |  |  |
| 29.54 | 9.68 | 11.12 |  |  | 29.21 | 5.98 | 4.93 |  |  |
| 29.55 | 9.66 | 11.08 |  |  | 29.22 | 5.97 | 4.89 |  |  |
| 29.56 | 9.63 | 11.04 |  |  | 29.23 | 5.97 | 4.85 |  |  |
| 29.57 | 9.60 | 11.00 |  |  | 29.24 | 5.97 | 4.82 |  |  |
| 29.59 | 9.58 | 10.93 |  |  | 29.25 | 5.96 | 4.78 |  |  |
| 29.60 | 9.56 | 10.89 |  |  | 29.26 | 5.94 | 4.74 |  |  |
| 29.61 | 9.55 | 10.85 |  |  | 29.27 | 5.93 | 4.70 |  |  |
| 29.62 | 9.52 | 10.81 |  |  | 29.28 | 5.92 | 4.66 |  |  |
| 29.63 | 9.51 | 10.77 |  |  | 29.29 | 5.92 | 4.63 |  |  |
| 29.64 | 9.49 | 10.73 |  |  | 29.30 | 5.91 | 4.59 |  |  |
| 29.65 | 9.48 | 10.69 |  |  | 29.31 | 5.89 | 4.55 |  |  |
| 29.66 | 9.46 | 10.66 |  |  | 29.32 | 5.89 | 4.51 |  |  |
| 29.67 | 9.44 | 10.62 |  |  | 29.33 | 5.89 | 4.47 |  |  |
| 29.68 | 9.41 | 10.58 |  |  | 29.34 | 5.88 | 4.44 |  |  |
| 29.69 | 9.38 | 10.54 |  |  | 29.36 | 5.87 | 4.36 |  |  |
| 29.70 | 9.37 | 10.50 |  |  | 29.37 | 5.86 | 4.32 |  |  |
| 29.71 | 9.36 | 10.46 |  |  | 29.38 | 5.84 | 4.28 |  |  |
| 29.72 | 9.33 | 10.42 |  |  | 29.39 | 5.83 | 4.25 |  |  |
| 29.73 | 9.31 | 10.39 |  |  | 29.41 | 5.83 | 4.17 |  |  |
| 29.74 | 9.28 | 10.35 |  |  | 29.42 | 5.82 | 4.13 |  |  |
| 29.75 | 9.26 | 10.31 |  |  | 29.43 | 5.80 | 4.09 |  |  |
| 29.78 | 9.24 | 10.19 |  |  | 29.44 | 5.80 | 4.05 |  |  |
| 29.79 | 9.23 | 10.16 |  |  | 29.45 | 5.79 | 4.02 |  |  |
| 29.80 | 9.20 | 10.12 |  |  | 29.48 | 5.77 | 3.90 |  |  |
| 29.81 | 9.18 | 10.08 |  |  | 29.49 | 5.76 | 3.86 |  |  |
| 29.82 | 9.14 | 10.04 |  |  | 29.50 | 5.76 | 3.83 |  |  |
| 29.83 | 9.12 | 10.00 |  |  | 29.52 | 5.75 | 3.75 |  |  |
| 29.84 | 9.11 | 9.96 |  |  | 29.53 | 5.74 | 3.71 |  |  |
| 29.85 | 9.10 | 9.92 |  |  | 29.54 | 5.74 | 3.67 |  |  |
| 29.86 | 9.09 | 9.89 |  |  | 29.55 | 5.72 | 3.64 |  |  |
| 29.87 | 9.07 | 9.85 |  |  | 29.57 | 5.71 | 3.56 |  |  |
| 29.88 | 9.06 | 9.81 |  |  | 29.58 | 5.70 | 3.52 |  |  |
| 29.89 | 9.04 | 9.77 |  |  | 29.59 | 5.69 | 3.48 |  |  |
| 29.91 | 9.01 | 9.69 |  |  | 29.60 | 5.68 | 3.45 |  |  |
| 29.92 | 8.99 | 9.66 |  |  | 29.61 | 5.67 | 3.41 |  |  |
| 29.93 | 8.96 | 9.62 |  |  | 29.62 | 5.66 | 3.37 |  |  |
| 29.94 | 8.94 | 9.58 |  |  | 29.63 | 5.64 | 3.33 |  |  |
| 29.95 | 8.93 | 9.54 |  |  | 29.65 | 5.63 | 3.26 |  |  |
| 29.96 | 8.91 | 9.50 |  |  | 29.66 | 5.62 | 3.22 |  |  |
| 29.97 | 8.90 | 9.46 |  |  | 29.67 | 5.61 | 3.18 |  |  |
| 29.98 | 8.89 | 9.42 |  |  | 29.68 | 5.61 | 3.14 |  |  |
| 30.00 | 8.86 | 9.35 |  |  | 29.69 | 5.60 | 3.10 |  |  |
| 30.02 | 8.82 | 9.27 |  |  | 29.70 | 5.59 | 3.07 |  |  |
| 30.03 | 8.79 | 9.23 |  |  | 29.71 | 5.58 | 3.03 |  |  |
| 30.04 | 8.77 | 9.19 |  |  | 29.72 | 5.57 | 2.99 |  |  |
| 30.06 | 8.74 | 9.12 |  |  | 29.73 | 5.57 | 2.95 |  |  |
| 30.08 | 8.72 | 9.04 |  |  | 29.74 | 5.55 | 2.91 |  |  |
| 30.09 | 8.70 | 9.00 |  |  | 29.77 | 5.52 | 2.80 |  |  |
| 30.10 | 8.66 | 8.96 |  |  | 29.78 | 5.51 | 2.76 |  |  |
| 30.11 | 8.65 | 8.92 |  |  | 29.79 | 5.50 | 2.72 |  |  |
| 30.12 | 8.63 | 8.89 |  |  | 29.80 | 5.50 | 2.68 |  |  |
| 30.13 | 8.60 | 8.85 |  |  | 29.81 | 5.49 | 2.65 |  |  |
| 30.14 | 8.58 | 8.81 |  |  | 29.82 | 5.48 | 2.61 |  |  |
| 30.15 | 8.55 | 8.77 |  |  | 29.83 | 5.48 | 2.57 |  |  |
| 30.16 | 8.53 | 8.73 |  |  | 29.85 | 5.46 | 2.49 |  |  |
| 30.17 | 8.51 | 8.69 |  |  | 29.86 | 5.45 | 2.46 |  |  |
| 30.18 | 8.48 | 8.66 |  |  | 29.87 | 5.44 | 2.42 |  |  |
| 30.19 | 8.46 | 8.62 |  |  | 29.88 | 5.43 | 2.38 |  |  |
| 30.20 | 8.45 | 8.58 |  |  | 29.89 | 5.41 | 2.34 |  |  |
| 30.21 | 8.43 | 8.54 |  |  | 29.90 | 5.41 | 2.30 |  |  |
| 30.23 | 8.38 | 8.46 |  |  | 29.91 | 5.40 | 2.27 |  |  |
| 30.24 | 8.35 | 8.42 |  |  | 29.92 | 5.39 | 2.23 |  |  |
| 30.25 | 8.32 | 8.39 |  |  | 29.93 | 5.38 | 2.19 |  |  |
| 30.26 | 8.30 | 8.35 |  |  | 29.94 | 5.36 | 2.15 |  |  |
| 30.27 | 8.29 | 8.31 |  |  | 29.96 | 5.34 | 2.08 |  |  |
| 30.28 | 8.27 | 8.27 |  |  | 29.97 | 5.33 | 2.04 |  |  |
| 30.29 | 8.25 | 8.23 |  |  | 29.98 | 5.32 | 2.00 |  |  |
| 30.30 | 8.23 | 8.19 |  |  | 29.99 | 5.31 | 1.96 |  |  |
| 30.31 | 8.21 | 8.16 |  |  | 30.00 | 5.29 | 1.92 |  |  |
| 30.32 | 8.20 | 8.12 |  |  | 30.01 | 5.28 | 1.89 |  |  |
| 30.33 | 8.17 | 8.08 |  |  | 30.02 | 5.27 | 1.85 |  |  |
| 30.34 | 8.15 | 8.04 |  |  | 30.03 | 5.26 | 1.81 |  |  |
| 30.35 | 8.13 | 8.00 |  |  | 30.04 | 5.26 | 1.77 |  |  |
| 30.36 | 8.11 | 7.96 |  |  | 30.05 | 5.25 | 1.73 |  |  |
| 30.37 | 8.09 | 7.92 |  |  | 30.06 | 5.23 | 1.70 |  |  |
| 30.38 | 8.06 | 7.89 |  |  | 30.07 | 5.22 | 1.66 |  |  |
| 30.39 | 8.04 | 7.85 |  |  | 30.10 | 5.20 | 1.54 |  |  |
| 30.40 | 8.03 | 7.81 |  |  | 30.12 | 5.18 | 1.47 |  |  |
| 30.41 | 8.02 | 7.77 |  |  | 30.13 | 5.17 | 1.43 |  |  |
| 30.42 | 7.99 | 7.73 |  |  | 30.14 | 5.15 | 1.39 |  |  |
| 30.43 | 7.95 | 7.69 |  |  | 30.15 | 5.15 | 1.35 |  |  |
| 30.44 | 7.93 | 7.66 |  |  | 30.17 | 5.13 | 1.28 |  |  |
| 30.45 | 7.90 | 7.62 |  |  | 30.19 | 5.12 | 1.20 |  |  |
| 30.46 | 7.88 | 7.58 |  |  | 30.20 | 5.11 | 1.16 |  |  |
| 30.47 | 7.86 | 7.54 |  |  | 30.22 | 5.08 | 1.09 |  |  |
| 30.48 | 7.83 | 7.50 |  |  | 30.23 | 5.07 | 1.05 |  |  |
| 30.49 | 7.81 | 7.46 |  |  | 30.24 | 5.05 | 1.01 |  |  |
| 30.50 | 7.79 | 7.42 |  |  | 30.25 | 5.03 | 0.97 |  |  |
| 30.52 | 7.75 | 7.35 |  |  | 30.26 | 5.03 | 0.93 |  |  |
| 30.53 | 7.73 | 7.31 |  |  | 30.27 | 5.01 | 0.90 |  |  |
| 30.54 | 7.71 | 7.27 |  |  | 30.28 | 5.00 | 0.86 |  |  |
| 30.55 | 7.69 | 7.23 |  |  | 30.29 | 4.98 | 0.82 |  |  |
| 30.56 | 7.67 | 7.19 |  |  | 30.30 | 4.97 | 0.78 |  |  |
| 30.57 | 7.64 | 7.15 |  |  | 30.31 | 4.96 | 0.74 |  |  |
| 30.58 | 7.62 | 7.12 |  |  | 30.32 | 4.94 | 0.71 |  |  |
| 30.59 | 7.60 | 7.08 |  |  | 30.33 | 4.93 | 0.67 |  |  |
| 30.62 | 7.55 | 6.96 |  |  | 30.34 | 4.92 | 0.63 |  |  |
| 30.64 | 7.50 | 6.89 |  |  | 30.35 | 4.91 | 0.59 |  |  |
| 30.65 | 7.48 | 6.85 |  |  | 30.36 | 4.89 | 0.55 |  |  |
| 30.66 | 7.47 | 6.81 |  |  | 30.37 | 4.88 | 0.52 |  |  |
| 30.67 | 7.45 | 6.77 |  |  | 30.38 | 4.86 | 0.48 |  |  |
| 30.68 | 7.43 | 6.73 |  |  | 30.39 | 4.85 | 0.44 |  |  |
| 30.69 | 7.40 | 6.69 |  |  | 30.42 | 4.84 | 0.32 |  |  |
| 30.72 | 7.35 | 6.58 |  |  | 30.43 | 4.83 | 0.29 |  |  |
| 30.73 | 7.34 | 6.54 |  |  | 30.44 | 4.80 | 0.25 |  |  |
| 30.74 | 7.33 | 6.50 |  |  | 30.45 | 4.79 | 0.21 |  |  |
| 30.75 | 7.32 | 6.46 |  |  | 30.46 | 4.77 | 0.17 |  |  |
| 30.76 | 7.31 | 6.42 |  |  | 30.47 | 4.76 | 0.13 |  |  |
| 30.77 | 7.30 | 6.39 |  |  | 30.49 | 4.73 | 0.06 |  |  |
| 30.78 | 7.28 | 6.35 |  |  | 30.50 | 4.72 | 0.02 |  |  |
| 30.79 | 7.26 | 6.31 |  |  | 30.51 | 4.70 | -0.02 |  |  |
| 30.81 | 7.22 | 6.23 |  |  | 30.52 | 4.69 | -0.06 |  |  |
| 30.82 | 7.21 | 6.19 |  |  | 30.53 | 4.67 | -0.09 |  |  |
| 30.83 | 7.19 | 6.15 |  |  | 30.54 | 4.65 | -0.13 |  |  |
| 30.84 | 7.18 | 6.12 |  |  | 30.55 | 4.64 | -0.17 |  |  |
| 30.85 | 7.15 | 6.08 |  |  | 30.57 | 4.61 | -0.25 |  |  |
| 30.86 | 7.13 | 6.04 |  |  | 30.58 | 4.60 | -0.28 |  |  |
| 30.87 | 7.11 | 6.00 |  |  | 30.59 | 4.59 | -0.32 |  |  |
| 30.88 | 7.09 | 5.96 |  |  | 30.61 | 4.58 | -0.40 |  |  |
| 30.90 | 7.06 | 5.89 |  |  | 30.62 | 4.57 | -0.44 |  |  |
| 30.91 | 7.03 | 5.85 |  |  | 30.63 | 4.56 | -0.47 |  |  |
| 30.92 | 7.02 | 5.81 |  |  | 30.64 | 4.55 | -0.51 |  |  |
| 30.93 | 7.00 | 5.77 |  |  | 30.65 | 4.53 | -0.55 |  |  |
| 30.94 | 6.98 | 5.73 |  |  | 30.66 | 4.52 | -0.59 |  |  |
| 30.95 | 6.97 | 5.69 |  |  | 30.67 | 4.51 | -0.63 |  |  |
| 30.96 | 6.95 | 5.65 |  |  | 30.68 | 4.48 | -0.66 |  |  |
| 30.97 | 6.94 | 5.62 |  |  | 30.69 | 4.47 | -0.70 |  |  |
| 30.98 | 6.92 | 5.58 |  |  | 30.71 | 4.44 | -0.78 |  |  |
| 30.99 | 6.90 | 5.54 |  |  | 30.72 | 4.44 | -0.82 |  |  |
| 31.01 | 6.86 | 5.46 |  |  | 30.73 | 4.43 | -0.85 |  |  |
| 31.02 | 6.84 | 5.42 |  |  | 30.74 | 4.42 | -0.89 |  |  |
| 31.03 | 6.82 | 5.39 |  |  | 30.75 | 4.41 | -0.93 |  |  |
| 31.04 | 6.78 | 5.35 |  |  | 30.76 | 4.40 | -0.97 |  |  |
| 31.06 | 6.74 | 5.27 |  |  | 30.77 | 4.40 | -1.01 |  |  |
| 31.07 | 6.72 | 5.23 |  |  | 30.79 | 4.36 | -1.08 |  |  |
| 31.08 | 6.70 | 5.19 |  |  | 30.81 | 4.33 | -1.16 |  |  |
| 31.09 | 6.68 | 5.15 |  |  | 30.82 | 4.31 | -1.20 |  |  |
| 31.10 | 6.66 | 5.12 |  |  | 30.83 | 4.29 | -1.24 |  |  |
| 31.11 | 6.64 | 5.08 |  |  | 30.84 | 4.28 | -1.27 |  |  |
| 31.12 | 6.62 | 5.04 |  |  | 30.86 | 4.25 | -1.35 |  |  |
| 31.13 | 6.60 | 5.00 |  |  | 30.87 | 4.24 | -1.39 |  |  |
| 31.14 | 6.59 | 4.96 |  |  | 30.88 | 4.23 | -1.43 |  |  |
| 31.15 | 6.57 | 4.92 |  |  | 30.89 | 4.21 | -1.46 |  |  |
| 31.18 | 6.51 | 4.81 |  |  | 30.90 | 4.21 | -1.50 |  |  |
| 31.19 | 6.50 | 4.77 |  |  | 30.91 | 4.20 | -1.54 |  |  |
| 31.20 | 6.48 | 4.73 |  |  | 30.92 | 4.19 | -1.58 |  |  |
| 31.21 | 6.45 | 4.69 |  |  | 30.93 | 4.19 | -1.62 |  |  |
| 31.22 | 6.43 | 4.65 |  |  | 30.94 | 4.16 | -1.65 |  |  |
| 31.23 | 6.41 | 4.62 |  |  | 30.95 | 4.14 | -1.69 |  |  |
| 31.24 | 6.38 | 4.58 |  |  | 30.96 | 4.13 | -1.73 |  |  |
| 31.25 | 6.35 | 4.54 |  |  | 30.98 | 4.11 | -1.81 |  |  |
| 31.26 | 6.33 | 4.50 |  |  | 30.99 | 4.10 | -1.84 |  |  |
| 31.27 | 6.32 | 4.46 |  |  | 31.03 | 4.05 | -2.00 |  |  |
| 31.28 | 6.29 | 4.42 |  |  | 31.04 | 4.04 | -2.03 |  |  |
| 31.29 | 6.28 | 4.39 |  |  | 31.05 | 4.02 | -2.07 |  |  |
| 31.30 | 6.25 | 4.35 |  |  | 31.06 | 4.00 | -2.11 |  |  |
| 31.31 | 6.22 | 4.31 |  |  | 31.07 | 3.98 | -2.15 |  |  |
| 31.32 | 6.21 | 4.27 |  |  | 31.08 | 3.97 | -2.19 |  |  |
| 31.33 | 6.18 | 4.23 |  |  | 31.10 | 3.95 | -2.26 |  |  |
| 31.34 | 6.15 | 4.19 |  |  | 31.11 | 3.94 | -2.30 |  |  |
| 31.35 | 6.14 | 4.15 |  |  | 31.12 | 3.93 | -2.34 |  |  |
| 31.36 | 6.13 | 4.12 |  |  | 31.13 | 3.92 | -2.38 |  |  |
| 31.37 | 6.09 | 4.08 |  |  | 31.14 | 3.91 | -2.42 |  |  |
| 31.38 | 6.07 | 4.04 |  |  | 31.15 | 3.91 | -2.45 |  |  |
| 31.40 | 6.03 | 3.96 |  |  | 31.16 | 3.89 | -2.49 |  |  |
| 31.42 | 5.98 | 3.88 |  |  | 31.17 | 3.87 | -2.53 |  |  |
| 31.43 | 5.97 | 3.85 |  |  | 31.18 | 3.86 | -2.57 |  |  |
| 31.44 | 5.94 | 3.81 |  |  | 31.19 | 3.85 | -2.61 |  |  |
| 31.45 | 5.92 | 3.77 |  |  | 31.20 | 3.84 | -2.64 |  |  |
| 31.46 | 5.89 | 3.73 |  |  | 31.21 | 3.83 | -2.68 |  |  |
| 31.47 | 5.87 | 3.69 |  |  | 31.23 | 3.81 | -2.76 |  |  |
| 31.48 | 5.85 | 3.65 |  |  | 31.24 | 3.80 | -2.80 |  |  |
| 31.49 | 5.83 | 3.62 |  |  | 31.25 | 3.80 | -2.83 |  |  |
| 31.50 | 5.81 | 3.58 |  |  | 31.26 | 3.78 | -2.87 |  |  |
| 31.51 | 5.78 | 3.54 |  |  | 31.27 | 3.78 | -2.91 |  |  |
| 31.52 | 5.76 | 3.50 |  |  | 31.28 | 3.77 | -2.95 |  |  |
| 31.53 | 5.73 | 3.46 |  |  | 31.29 | 3.75 | -2.99 |  |  |
| 31.54 | 5.70 | 3.42 |  |  | 31.30 | 3.75 | -3.02 |  |  |
| 31.55 | 5.68 | 3.38 |  |  | 31.32 | 3.72 | -3.10 |  |  |
| 31.56 | 5.65 | 3.35 |  |  | 31.33 | 3.71 | -3.14 |  |  |
| 31.58 | 5.62 | 3.27 |  |  | 31.34 | 3.70 | -3.18 |  |  |
| 31.59 | 5.59 | 3.23 |  |  | 31.35 | 3.70 | -3.21 |  |  |
| 31.60 | 5.57 | 3.19 |  |  | 31.38 | 3.67 | -3.33 |  |  |
| 31.61 | 5.55 | 3.15 |  |  | 31.39 | 3.65 | -3.37 |  |  |
| 31.62 | 5.54 | 3.12 |  |  | 31.40 | 3.65 | -3.41 |  |  |
| 31.63 | 5.52 | 3.08 |  |  | 31.41 | 3.65 | -3.44 |  |  |
| 31.64 | 5.50 | 3.04 |  |  | 31.42 | 3.63 | -3.48 |  |  |
| 31.65 | 5.49 | 3.00 |  |  | 31.43 | 3.63 | -3.52 |  |  |
| 31.66 | 5.47 | 2.96 |  |  | 31.45 | 3.61 | -3.60 |  |  |
| 31.67 | 5.45 | 2.92 |  |  | 31.46 | 3.59 | -3.63 |  |  |
| 31.68 | 5.44 | 2.88 |  |  | 31.47 | 3.59 | -3.67 |  |  |
| 31.69 | 5.42 | 2.85 |  |  | 31.48 | 3.57 | -3.71 |  |  |
| 31.70 | 5.39 | 2.81 |  |  | 31.50 | 3.55 | -3.79 |  |  |
| 31.71 | 5.36 | 2.77 |  |  | 31.51 | 3.54 | -3.82 |  |  |
| 31.73 | 5.32 | 2.69 |  |  | 31.52 | 3.54 | -3.86 |  |  |
| 31.74 | 5.29 | 2.65 |  |  | 31.53 | 3.54 | -3.90 |  |  |
| 31.76 | 5.23 | 2.58 |  |  | 31.54 | 3.52 | -3.94 |  |  |
| 31.77 | 5.20 | 2.54 |  |  | 31.56 | 3.51 | -4.01 |  |  |
| 31.78 | 5.19 | 2.50 |  |  | 31.57 | 3.49 | -4.05 |  |  |
| 31.79 | 5.18 | 2.46 |  |  | 31.58 | 3.48 | -4.09 |  |  |
| 31.80 | 5.15 | 2.42 |  |  | 31.59 | 3.47 | -4.13 |  |  |
| 31.81 | 5.12 | 2.38 |  |  | 31.60 | 3.45 | -4.17 |  |  |
| 31.83 | 5.06 | 2.31 |  |  | 31.61 | 3.45 | -4.20 |  |  |
| 31.84 | 5.04 | 2.27 |  |  | 31.63 | 3.42 | -4.28 |  |  |
| 31.85 | 5.02 | 2.23 |  |  | 31.66 | 3.41 | -4.39 |  |  |
| 31.86 | 4.99 | 2.19 |  |  | 31.67 | 3.40 | -4.43 |  |  |
| 31.87 | 4.97 | 2.15 |  |  | 31.68 | 3.39 | -4.47 |  |  |
| 31.88 | 4.94 | 2.12 |  |  | 31.69 | 3.38 | -4.51 |  |  |
| 31.89 | 4.92 | 2.08 |  |  | 31.70 | 3.38 | -4.55 |  |  |
| 31.90 | 4.90 | 2.04 |  |  | 31.72 | 3.35 | -4.62 |  |  |
| 31.91 | 4.87 | 2.00 |  |  | 31.73 | 3.34 | -4.66 |  |  |
| 31.92 | 4.85 | 1.96 |  |  | 31.74 | 3.33 | -4.70 |  |  |
| 31.93 | 4.84 | 1.92 |  |  | 31.75 | 3.32 | -4.74 |  |  |
| 31.94 | 4.83 | 1.88 |  |  | 31.76 | 3.31 | -4.78 |  |  |
| 31.95 | 4.81 | 1.85 |  |  | 31.77 | 3.31 | -4.81 |  |  |
| 31.96 | 4.77 | 1.81 |  |  | 31.78 | 3.30 | -4.85 |  |  |
| 31.97 | 4.75 | 1.77 |  |  | 31.79 | 3.30 | -4.89 |  |  |
| 31.98 | 4.74 | 1.73 |  |  | 31.80 | 3.28 | -4.93 |  |  |
| 31.99 | 4.71 | 1.69 |  |  | 31.81 | 3.27 | -4.97 |  |  |
| 32.00 | 4.69 | 1.65 |  |  | 31.82 | 3.26 | -5.00 |  |  |
| 32.01 | 4.67 | 1.62 |  |  | 31.83 | 3.25 | -5.04 |  |  |
| 32.02 | 4.66 | 1.58 |  |  | 31.84 | 3.24 | -5.08 |  |  |
| 32.03 | 4.63 | 1.54 |  |  | 31.85 | 3.23 | -5.12 |  |  |
| 32.04 | 4.61 | 1.50 |  |  | 31.86 | 3.23 | -5.16 |  |  |
| 32.05 | 4.60 | 1.46 |  |  | 31.87 | 3.22 | -5.19 |  |  |
| 32.06 | 4.56 | 1.42 |  |  | 31.88 | 3.21 | -5.23 |  |  |
| 32.07 | 4.52 | 1.38 |  |  | 31.90 | 3.20 | -5.31 |  |  |
| 32.08 | 4.49 | 1.35 |  |  | 31.91 | 3.19 | -5.35 |  |  |
| 32.09 | 4.46 | 1.31 |  |  | 31.92 | 3.18 | -5.38 |  |  |
| 32.10 | 4.43 | 1.27 |  |  | 31.93 | 3.17 | -5.42 |  |  |
| 32.11 | 4.40 | 1.23 |  |  | 31.94 | 3.16 | -5.46 |  |  |
| 32.13 | 4.36 | 1.15 |  |  | 31.95 | 3.15 | -5.50 |  |  |
| 32.14 | 4.33 | 1.12 |  |  | 31.96 | 3.14 | -5.54 |  |  |
| 32.15 | 4.30 | 1.08 |  |  | 31.97 | 3.13 | -5.57 |  |  |
| 32.16 | 4.29 | 1.04 |  |  | 31.99 | 3.09 | -5.65 |  |  |
| 32.17 | 4.25 | 1.00 |  |  | 32.00 | 3.09 | -5.69 |  |  |
| 32.18 | 4.23 | 0.96 |  |  | 32.01 | 3.08 | -5.73 |  |  |
| 32.20 | 4.20 | 0.88 |  |  | 32.03 | 3.06 | -5.80 |  |  |
| 32.21 | 4.18 | 0.85 |  |  | 32.04 | 3.05 | -5.84 |  |  |
| 32.22 | 4.14 | 0.81 |  |  | 32.05 | 3.04 | -5.88 |  |  |
| 32.23 | 4.13 | 0.77 |  |  | 32.07 | 3.02 | -5.96 |  |  |
| 32.24 | 4.11 | 0.73 |  |  | 32.08 | 3.02 | -5.99 |  |  |
| 32.25 | 4.08 | 0.69 |  |  | 32.09 | 3.02 | -6.03 |  |  |
| 32.26 | 4.06 | 0.65 |  |  | 32.10 | 3.00 | -6.07 |  |  |
| 32.27 | 4.04 | 0.62 |  |  | 32.11 | 2.99 | -6.11 |  |  |
| 32.28 | 4.01 | 0.58 |  |  | 32.12 | 2.97 | -6.15 |  |  |
| 32.29 | 3.98 | 0.54 |  |  | 32.13 | 2.97 | -6.18 |  |  |
| 32.30 | 3.95 | 0.50 |  |  | 32.14 | 2.96 | -6.22 |  |  |
| 32.31 | 3.93 | 0.46 |  |  | 32.16 | 2.94 | -6.30 |  |  |
| 32.32 | 3.90 | 0.42 |  |  | 32.17 | 2.93 | -6.34 |  |  |
| 32.33 | 3.88 | 0.38 |  |  | 32.18 | 2.92 | -6.37 |  |  |
| 32.34 | 3.86 | 0.35 |  |  | 32.19 | 2.92 | -6.41 |  |  |
| 32.35 | 3.84 | 0.31 |  |  | 32.20 | 2.91 | -6.45 |  |  |
| 32.36 | 3.81 | 0.27 |  |  | 32.21 | 2.90 | -6.49 |  |  |
| 32.38 | 3.77 | 0.19 |  |  | 32.22 | 2.90 | -6.53 |  |  |
| 32.39 | 3.75 | 0.15 |  |  | 32.23 | 2.89 | -6.56 |  |  |
| 32.40 | 3.73 | 0.11 |  |  | 32.26 | 2.84 | -6.68 |  |  |
| 32.41 | 3.72 | 0.08 |  |  | 32.27 | 2.82 | -6.72 |  |  |
| 32.42 | 3.70 | 0.04 |  |  | 32.28 | 2.81 | -6.75 |  |  |
| 32.43 | 3.70 | 0.00 |  |  | 32.29 | 2.80 | -6.79 |  |  |
| 32.44 | 3.68 | -0.04 |  |  | 32.30 | 2.78 | -6.83 |  |  |
| 32.45 | 3.64 | -0.08 |  |  | 32.31 | 2.77 | -6.87 |  |  |
| 32.46 | 3.62 | -0.12 |  |  | 32.32 | 2.76 | -6.91 |  |  |
| 32.47 | 3.59 | -0.15 |  |  | 32.33 | 2.74 | -6.94 |  |  |
| 32.48 | 3.57 | -0.19 |  |  | 32.34 | 2.74 | -6.98 |  |  |
| 32.49 | 3.54 | -0.23 |  |  | 32.35 | 2.73 | -7.02 |  |  |
| 32.51 | 3.49 | -0.31 |  |  | 32.36 | 2.72 | -7.06 |  |  |
| 32.52 | 3.48 | -0.35 |  |  | 32.37 | 2.71 | -7.10 |  |  |
| 32.53 | 3.47 | -0.39 |  |  | 32.38 | 2.70 | -7.14 |  |  |
| 32.54 | 3.45 | -0.42 |  |  | 32.39 | 2.70 | -7.17 |  |  |
| 32.55 | 3.43 | -0.46 |  |  | 32.40 | 2.69 | -7.21 |  |  |
| 32.56 | 3.41 | -0.50 |  |  | 32.41 | 2.68 | -7.25 |  |  |
| 32.57 | 3.38 | -0.54 |  |  | 32.42 | 2.68 | -7.29 |  |  |
| 32.59 | 3.32 | -0.62 |  |  | 32.43 | 2.67 | -7.33 |  |  |
| 32.60 | 3.31 | -0.65 |  |  | 32.44 | 2.66 | -7.36 |  |  |
| 32.61 | 3.28 | -0.69 |  |  | 32.45 | 2.64 | -7.40 |  |  |
| 32.62 | 3.27 | -0.73 |  |  | 32.46 | 2.64 | -7.44 |  |  |
| 32.63 | 3.24 | -0.77 |  |  | 32.47 | 2.63 | -7.48 |  |  |
| 32.64 | 3.21 | -0.81 |  |  | 32.48 | 2.61 | -7.52 |  |  |
| 32.65 | 3.19 | -0.85 |  |  | 32.49 | 2.59 | -7.55 |  |  |
| 32.66 | 3.16 | -0.89 |  |  | 32.50 | 2.57 | -7.59 |  |  |
| 32.69 | 3.08 | -1.00 |  |  | 32.51 | 2.56 | -7.63 |  |  |
| 32.70 | 3.04 | -1.04 |  |  | 32.52 | 2.55 | -7.67 |  |  |
| 32.71 | 3.02 | -1.08 |  |  | 32.53 | 2.54 | -7.71 |  |  |
| 32.72 | 3.01 | -1.12 |  |  | 32.54 | 2.52 | -7.74 |  |  |
| 32.73 | 2.98 | -1.15 |  |  | 32.56 | 2.49 | -7.82 |  |  |
| 32.74 | 2.95 | -1.19 |  |  | 32.58 | 2.44 | -7.90 |  |  |
| 32.76 | 2.91 | -1.27 |  |  | 32.59 | 2.43 | -7.93 |  |  |
| 32.77 | 2.89 | -1.31 |  |  | 32.60 | 2.42 | -7.97 |  |  |
| 32.78 | 2.88 | -1.35 |  |  | 32.61 | 2.41 | -8.01 |  |  |
| 32.79 | 2.85 | -1.39 |  |  | 32.62 | 2.39 | -8.05 |  |  |
| 32.80 | 2.84 | -1.42 |  |  | 32.63 | 2.38 | -8.09 |  |  |
| 32.81 | 2.79 | -1.46 |  |  | 32.64 | 2.36 | -8.12 |  |  |
| 32.82 | 2.76 | -1.50 |  |  | 32.65 | 2.34 | -8.16 |  |  |
| 32.83 | 2.75 | -1.54 |  |  | 32.66 | 2.33 | -8.20 |  |  |
| 32.84 | 2.72 | -1.58 |  |  | 32.67 | 2.32 | -8.24 |  |  |
| 32.85 | 2.71 | -1.62 |  |  | 32.68 | 2.31 | -8.28 |  |  |
| 32.86 | 2.69 | -1.65 |  |  | 32.69 | 2.29 | -8.31 |  |  |
| 32.87 | 2.66 | -1.69 |  |  | 32.70 | 2.27 | -8.35 |  |  |
| 32.88 | 2.65 | -1.73 |  |  | 32.71 | 2.26 | -8.39 |  |  |
| 32.89 | 2.62 | -1.77 |  |  | 32.72 | 2.24 | -8.43 |  |  |
| 32.91 | 2.58 | -1.85 |  |  | 32.73 | 2.22 | -8.47 |  |  |
| 32.92 | 2.56 | -1.89 |  |  | 32.74 | 2.20 | -8.51 |  |  |
| 32.95 | 2.51 | -2.00 |  |  | 32.75 | 2.17 | -8.54 |  |  |
| 32.96 | 2.48 | -2.04 |  |  | 32.76 | 2.16 | -8.58 |  |  |
| 32.97 | 2.45 | -2.08 |  |  | 32.77 | 2.14 | -8.62 |  |  |
| 32.99 | 2.42 | -2.15 |  |  | 32.78 | 2.13 | -8.66 |  |  |
| 33.00 | 2.41 | -2.19 |  |  | 32.79 | 2.09 | -8.70 |  |  |
| 33.02 | 2.36 | -2.27 |  |  | 32.80 | 2.07 | -8.73 |  |  |
| 33.03 | 2.34 | -2.31 |  |  | 32.81 | 2.06 | -8.77 |  |  |
| 33.04 | 2.32 | -2.35 |  |  | 32.83 | 2.03 | -8.85 |  |  |
| 33.05 | 2.29 | -2.39 |  |  | 32.84 | 2.02 | -8.89 |  |  |
| 33.06 | 2.28 | -2.42 |  |  | 32.85 | 2.00 | -8.92 |  |  |
| 33.08 | 2.24 | -2.50 |  |  | 32.86 | 1.98 | -8.96 |  |  |
| 33.09 | 2.21 | -2.54 |  |  | 32.87 | 1.98 | -9.00 |  |  |
| 33.10 | 2.19 | -2.58 |  |  | 32.88 | 1.96 | -9.04 |  |  |
| 33.11 | 2.17 | -2.62 |  |  | 32.89 | 1.93 | -9.08 |  |  |
| 33.12 | 2.16 | -2.65 |  |  | 32.90 | 1.90 | -9.11 |  |  |
| 33.13 | 2.13 | -2.69 |  |  | 32.91 | 1.88 | -9.15 |  |  |
| 33.14 | 2.11 | -2.73 |  |  | 32.92 | 1.86 | -9.19 |  |  |
| 33.15 | 2.08 | -2.77 |  |  | 32.93 | 1.86 | -9.23 |  |  |
| 33.16 | 2.07 | -2.81 |  |  | 32.94 | 1.83 | -9.27 |  |  |
| 33.17 | 2.06 | -2.85 |  |  | 32.95 | 1.81 | -9.30 |  |  |
| 33.18 | 2.03 | -2.89 |  |  | 32.96 | 1.79 | -9.34 |  |  |
| 33.19 | 2.02 | -2.92 |  |  | 32.97 | 1.78 | -9.38 |  |  |
| 33.20 | 2.00 | -2.96 |  |  | 32.98 | 1.75 | -9.42 |  |  |
| 33.21 | 1.98 | -3.00 |  |  | 32.99 | 1.74 | -9.46 |  |  |
| 33.22 | 1.95 | -3.04 |  |  | 33.00 | 1.71 | -9.49 |  |  |
| 33.23 | 1.93 | -3.08 |  |  | 33.01 | 1.69 | -9.53 |  |  |
| 33.25 | 1.92 | -3.16 |  |  | 33.02 | 1.66 | -9.57 |  |  |
| 33.26 | 1.89 | -3.19 |  |  | 33.03 | 1.63 | -9.61 |  |  |
| 33.27 | 1.88 | -3.23 |  |  | 33.04 | 1.62 | -9.65 |  |  |
| 33.28 | 1.86 | -3.27 |  |  | 33.05 | 1.61 | -9.69 |  |  |
| 33.29 | 1.83 | -3.31 |  |  | 33.06 | 1.60 | -9.72 |  |  |
| 33.30 | 1.82 | -3.35 |  |  | 33.07 | 1.59 | -9.76 |  |  |
| 33.31 | 1.81 | -3.39 |  |  | 33.08 | 1.57 | -9.80 |  |  |
| 33.33 | 1.78 | -3.46 |  |  | 33.09 | 1.55 | -9.84 |  |  |
| 33.34 | 1.76 | -3.50 |  |  | 33.10 | 1.54 | -9.88 |  |  |
| 33.35 | 1.73 | -3.54 |  |  | 33.11 | 1.52 | -9.91 |  |  |
| 33.36 | 1.71 | -3.58 |  |  | 33.12 | 1.51 | -9.95 |  |  |
| 33.37 | 1.68 | -3.62 |  |  | 33.13 | 1.48 | -9.99 |  |  |
| 33.38 | 1.66 | -3.66 |  |  | 33.14 | 1.48 | -10.03 |  |  |
| 33.39 | 1.64 | -3.69 |  |  | 33.15 | 1.46 | -10.07 |  |  |
| 33.40 | 1.62 | -3.73 |  |  | 33.16 | 1.44 | -10.10 |  |  |
| 33.41 | 1.60 | -3.77 |  |  | 33.17 | 1.43 | -10.14 |  |  |
| 33.42 | 1.58 | -3.81 |  |  | 33.18 | 1.41 | -10.18 |  |  |
| 33.43 | 1.57 | -3.85 |  |  | 33.19 | 1.39 | -10.22 |  |  |
| 33.44 | 1.57 | -3.89 |  |  | 33.20 | 1.38 | -10.26 |  |  |
| 33.45 | 1.54 | -3.92 |  |  | 33.21 | 1.37 | -10.29 |  |  |
| 33.46 | 1.53 | -3.96 |  |  | 33.22 | 1.35 | -10.33 |  |  |
| 33.47 | 1.51 | -4.00 |  |  | 33.23 | 1.34 | -10.37 |  |  |
| 33.48 | 1.50 | -4.04 |  |  | 33.24 | 1.31 | -10.41 |  |  |
| 33.50 | 1.47 | -4.12 |  |  | 33.26 | 1.28 | -10.48 |  |  |
| 33.51 | 1.46 | -4.16 |  |  | 33.27 | 1.27 | -10.52 |  |  |
| 33.52 | 1.45 | -4.19 |  |  | 33.28 | 1.25 | -10.56 |  |  |
| 33.53 | 1.42 | -4.23 |  |  | 33.30 | 1.22 | -10.64 |  |  |
| 33.54 | 1.40 | -4.27 |  |  | 33.31 | 1.19 | -10.67 |  |  |
| 33.55 | 1.39 | -4.31 |  |  | 33.32 | 1.18 | -10.71 |  |  |
| 33.57 | 1.36 | -4.39 |  |  | 33.33 | 1.16 | -10.75 |  |  |
| 33.58 | 1.35 | -4.42 |  |  | 33.34 | 1.15 | -10.79 |  |  |
| 33.59 | 1.33 | -4.46 |  |  | 33.35 | 1.13 | -10.83 |  |  |
| 33.60 | 1.32 | -4.50 |  |  | 33.37 | 1.10 | -10.90 |  |  |
| 33.61 | 1.29 | -4.54 |  |  | 33.38 | 1.09 | -10.94 |  |  |
| 33.62 | 1.28 | -4.58 |  |  | 33.40 | 1.06 | -11.02 |  |  |
| 33.63 | 1.25 | -4.62 |  |  | 33.41 | 1.03 | -11.06 |  |  |
| 33.64 | 1.24 | -4.66 |  |  | 33.42 | 1.01 | -11.09 |  |  |
| 33.65 | 1.23 | -4.69 |  |  | 33.43 | 0.99 | -11.13 |  |  |
| 33.66 | 1.21 | -4.73 |  |  | 33.44 | 0.97 | -11.17 |  |  |
| 33.68 | 1.18 | -4.81 |  |  | 33.45 | 0.95 | -11.21 |  |  |
| 33.69 | 1.16 | -4.85 |  |  | 33.46 | 0.95 | -11.25 |  |  |
| 33.70 | 1.13 | -4.89 |  |  | 33.47 | 0.93 | -11.28 |  |  |
| 33.71 | 1.11 | -4.92 |  |  | 33.48 | 0.91 | -11.32 |  |  |
| 33.72 | 1.09 | -4.96 |  |  | 33.49 | 0.90 | -11.36 |  |  |
| 33.73 | 1.07 | -5.00 |  |  | 33.50 | 0.87 | -11.40 |  |  |
| 33.74 | 1.06 | -5.04 |  |  | 33.51 | 0.86 | -11.44 |  |  |
| 33.75 | 1.04 | -5.08 |  |  | 33.52 | 0.84 | -11.47 |  |  |
| 33.76 | 1.03 | -5.12 |  |  | 33.53 | 0.83 | -11.51 |  |  |
| 33.77 | 1.02 | -5.16 |  |  | 33.54 | 0.81 | -11.55 |  |  |
| 33.78 | 0.99 | -5.19 |  |  | 33.55 | 0.80 | -11.59 |  |  |
| 33.79 | 0.97 | -5.23 |  |  | 33.56 | 0.79 | -11.63 |  |  |
| 33.83 | 0.90 | -5.39 |  |  | 33.57 | 0.77 | -11.66 |  |  |
| 33.84 | 0.88 | -5.42 |  |  | 33.58 | 0.77 | -11.70 |  |  |
| 33.85 | 0.86 | -5.46 |  |  | 33.59 | 0.76 | -11.74 |  |  |
| 33.86 | 0.84 | -5.50 |  |  | 33.60 | 0.74 | -11.78 |  |  |
| 33.87 | 0.82 | -5.54 |  |  | 33.61 | 0.73 | -11.82 |  |  |
| 33.88 | 0.81 | -5.58 |  |  | 33.62 | 0.72 | -11.85 |  |  |
| 33.89 | 0.79 | -5.62 |  |  | 33.64 | 0.69 | -11.93 |  |  |
| 33.90 | 0.78 | -5.66 |  |  | 33.66 | 0.66 | -12.01 |  |  |
| 33.92 | 0.75 | -5.73 |  |  | 33.68 | 0.63 | -12.08 |  |  |
| 33.93 | 0.73 | -5.77 |  |  | 33.69 | 0.62 | -12.12 |  |  |
| 33.94 | 0.72 | -5.81 |  |  | 33.70 | 0.61 | -12.16 |  |  |
| 33.95 | 0.70 | -5.85 |  |  | 33.71 | 0.59 | -12.20 |  |  |
| 33.97 | 0.67 | -5.92 |  |  | 33.72 | 0.57 | -12.24 |  |  |
| 33.98 | 0.66 | -5.96 |  |  | 33.73 | 0.55 | -12.27 |  |  |
| 33.99 | 0.65 | -6.00 |  |  | 33.74 | 0.53 | -12.31 |  |  |
| 34.00 | 0.63 | -6.04 |  |  | 33.76 | 0.50 | -12.39 |  |  |
| 34.01 | 0.62 | -6.08 |  |  | 33.77 | 0.48 | -12.43 |  |  |
| 34.02 | 0.61 | -6.12 |  |  | 33.78 | 0.46 | -12.46 |  |  |
| 34.03 | 0.60 | -6.16 |  |  | 33.79 | 0.44 | -12.50 |  |  |
| 34.05 | 0.58 | -6.23 |  |  | 33.80 | 0.43 | -12.54 |  |  |
| 34.06 | 0.57 | -6.27 |  |  | 33.81 | 0.42 | -12.58 |  |  |
| 34.07 | 0.56 | -6.31 |  |  | 33.82 | 0.41 | -12.62 |  |  |
| 34.08 | 0.54 | -6.35 |  |  | 33.83 | 0.41 | -12.65 |  |  |
| 34.09 | 0.53 | -6.39 |  |  | 33.84 | 0.40 | -12.69 |  |  |
| 34.10 | 0.51 | -6.43 |  |  | 33.85 | 0.39 | -12.73 |  |  |
| 34.11 | 0.50 | -6.46 |  |  | 33.86 | 0.39 | -12.77 |  |  |
| 34.12 | 0.50 | -6.50 |  |  | 33.87 | 0.38 | -12.81 |  |  |
| 34.13 | 0.48 | -6.54 |  |  | 33.88 | 0.37 | -12.84 |  |  |
| 34.15 | 0.46 | -6.62 |  |  | 33.89 | 0.36 | -12.88 |  |  |
| 34.16 | 0.45 | -6.66 |  |  | 33.92 | 0.32 | -13.00 |  |  |
| 34.18 | 0.42 | -6.73 |  |  | 33.93 | 0.31 | -13.03 |  |  |
| 34.22 | 0.38 | -6.89 |  |  | 33.94 | 0.30 | -13.07 |  |  |
| 34.23 | 0.37 | -6.93 |  |  | 33.95 | 0.29 | -13.11 |  |  |
| 34.24 | 0.36 | -6.96 |  |  | 33.96 | 0.27 | -13.15 |  |  |
| 34.25 | 0.34 | -7.00 |  |  | 33.97 | 0.27 | -13.19 |  |  |
| 34.26 | 0.33 | -7.04 |  |  | 33.98 | 0.26 | -13.22 |  |  |
| 34.27 | 0.33 | -7.08 |  |  | 33.99 | 0.26 | -13.26 |  |  |
| 34.28 | 0.32 | -7.12 |  |  | 34.00 | 0.26 | -13.30 |  |  |
| 34.29 | 0.32 | -7.16 |  |  | 34.01 | 0.25 | -13.34 |  |  |
| 34.31 | 0.30 | -7.23 |  |  | 34.02 | 0.24 | -13.38 |  |  |
| 34.32 | 0.30 | -7.27 |  |  | 34.03 | 0.24 | -13.42 |  |  |
| 34.34 | 0.28 | -7.35 |  |  | 34.04 | 0.24 | -13.45 |  |  |
| 34.36 | 0.27 | -7.43 |  |  | 34.05 | 0.22 | -13.49 |  |  |
| 34.37 | 0.25 | -7.46 |  |  | 34.06 | 0.21 | -13.53 |  |  |
| 34.38 | 0.24 | -7.50 |  |  | 34.07 | 0.21 | -13.57 |  |  |
| 34.39 | 0.24 | -7.54 |  |  | 34.08 | 0.20 | -13.61 |  |  |
| 34.40 | 0.24 | -7.58 |  |  | 34.10 | 0.19 | -13.68 |  |  |
| 34.41 | 0.22 | -7.62 |  |  | 34.11 | 0.19 | -13.72 |  |  |
| 34.42 | 0.22 | -7.66 |  |  | 34.12 | 0.18 | -13.76 |  |  |
| 34.43 | 0.21 | -7.69 |  |  | 34.15 | 0.15 | -13.87 |  |  |
| 34.45 | 0.19 | -7.77 |  |  | 34.16 | 0.15 | -13.91 |  |  |
| 34.46 | 0.19 | -7.81 |  |  | 34.17 | 0.15 | -13.95 |  |  |
| 34.47 | 0.19 | -7.85 |  |  | 34.18 | 0.15 | -13.99 |  |  |
| 34.48 | 0.19 | -7.89 |  |  | 34.19 | 0.14 | -14.02 |  |  |
| 34.49 | 0.18 | -7.93 |  |  | 34.20 | 0.14 | -14.06 |  |  |
| 34.50 | 0.18 | -7.96 |  |  | 34.21 | 0.14 | -14.10 |  |  |
| 34.52 | 0.16 | -8.04 |  |  | 34.22 | 0.13 | -14.14 |  |  |
| 34.53 | 0.15 | -8.08 |  |  | 34.23 | 0.13 | -14.18 |  |  |
| 34.54 | 0.15 | -8.12 |  |  | 34.24 | 0.12 | -14.21 |  |  |
| 34.55 | 0.14 | -8.16 |  |  | 34.27 | 0.12 | -14.33 |  |  |
| 34.56 | 0.13 | -8.19 |  |  | 34.28 | 0.12 | -14.37 |  |  |
| 34.57 | 0.12 | -8.23 |  |  | 34.29 | 0.11 | -14.40 |  |  |
| 34.58 | 0.12 | -8.27 |  |  | 34.30 | 0.11 | -14.44 |  |  |
| 34.59 | 0.10 | -8.31 |  |  | 34.32 | 0.10 | -14.52 |  |  |
| 34.60 | 0.08 | -8.35 |  |  | 34.33 | 0.10 | -14.56 |  |  |
| 34.62 | 0.08 | -8.43 |  |  | 34.34 | 0.09 | -14.60 |  |  |
| 34.63 | 0.07 | -8.46 |  |  | 34.35 | 0.09 | -14.63 |  |  |
| 34.64 | 0.06 | -8.50 |  |  | 34.36 | 0.09 | -14.67 |  |  |
| 34.65 | 0.06 | -8.54 |  |  | 34.37 | 0.09 | -14.71 |  |  |
| 34.66 | 0.05 | -8.58 |  |  | 34.38 | 0.08 | -14.75 |  |  |
| 34.67 | 0.04 | -8.62 |  |  | 34.40 | 0.08 | -14.82 |  |  |
| 34.68 | 0.04 | -8.66 |  |  | 34.42 | 0.07 | -14.90 |  |  |
| 34.69 | 0.04 | -8.69 |  |  | 34.43 | 0.06 | -14.94 |  |  |
| 34.72 | 0.04 | -8.81 |  |  | 34.44 | 0.06 | -14.98 |  |  |
| 34.73 | 0.04 | -8.85 |  |  | 34.45 | 0.06 | -15.01 |  |  |
| 34.74 | 0.04 | -8.89 |  |  | 34.47 | 0.06 | -15.09 |  |  |
| 34.75 | 0.03 | -8.93 |  |  | 34.48 | 0.06 | -15.13 |  |  |
| 34.77 | 0.03 | -9.00 |  |  | 34.50 | 0.06 | -15.20 |  |  |
| 34.78 | 0.02 | -9.04 |  |  | 34.51 | 0.05 | -15.24 |  |  |
| 34.79 | 0.02 | -9.08 |  |  | 34.52 | 0.05 | -15.28 |  |  |
| 34.81 | 0.02 | -9.16 |  |  | 34.53 | 0.05 | -15.32 |  |  |
| 34.85 | 0.02 | -9.31 |  |  | 34.55 | 0.04 | -15.39 |  |  |
| 34.87 | 0.01 | -9.39 |  |  | 34.58 | 0.04 | -15.51 |  |  |
| 34.94 | 0.01 | -9.66 |  |  | 34.59 | 0.03 | -15.55 |  |  |
| 34.96 | 0.01 | -9.73 |  |  | 34.60 | 0.03 | -15.58 |  |  |
| 34.97 | 0.01 | -9.77 |  |  | 34.61 | 0.03 | -15.62 |  |  |
| 34.98 | 0.01 | -9.81 |  |  | 34.62 | 0.03 | -15.66 |  |  |
| 35.05 | 0.01 | -10.08 |  |  | 34.63 | 0.03 | -15.70 |  |  |
| 35.06 | 0.01 | -10.12 |  |  | 34.64 | 0.03 | -15.74 |  |  |
| 35.12 | 0.01 | -10.35 |  |  | 34.65 | 0.02 | -15.78 |  |  |
| 35.13 | 0.00 | -10.39 |  |  | 34.67 | 0.02 | -15.85 |  |  |
| 35.14 | 0.00 | -10.43 |  |  | 34.68 | 0.01 | -15.89 |  |  |
| 35.15 | 0.00 | -10.46 |  |  | 34.69 | 0.01 | -15.93 |  |  |
| 41.31 | 0.00 | -34.16 |  |  | 34.70 | 0.01 | -15.97 |  |  |
| 41.31 | 0.00 | -34.18 |  |  | 34.71 | 0.01 | -16.00 |  |  |
|  |  |  |  |  | 34.72 | 0.01 | -16.04 |  |  |
|  |  |  |  |  | 34.74 | 0.01 | -16.12 |  |  |
|  |  |  |  |  | 34.75 | 0.01 | -16.16 |  |  |
|  |  |  |  |  | 34.78 | 0.01 | -16.27 |  |  |
|  |  |  |  |  | 34.79 | 0.00 | -16.31 |  |  |
|  |  |  |  |  | 34.81 | 0.00 | -16.38 |  |  |
|  |  |  |  |  | 34.82 | 0.00 | -16.42 |  |  |
|  |  |  |  |  | 38.91 | 0.00 | -31.99 |  |  |
|  |  |  |  |  | 38.91 | 0.00 | -32.01 |  |  |

### Data used to calculate results in Fig 4 and Table 3.

**S2 Table**

|  | UD | | | |  | MD | | | |
| --- | --- | --- | --- | --- | --- | --- | --- | --- | --- |
|  | Percentage Volume (%) | | Histogram Data | |  | Percentage Volume (%) | | Histogram Data | |
| Dose (Gy) | DVH data | Linear Fit Data | dV/dD | Relative Frequency | Dose (Gy) | DVH data | Linear Fit Data | dV/dD | Relative Frequency |
| 0.00 | 100.00 | 106.26 | -10.20 | 0.00 | 0.00 | 100.00 | 93.85 | -14.20 | 0.00 |
| 1.40 | 100.00 | 102.39 | -9.80 | 0.00 | 1.89 | 100.00 | 90.81 | -13.80 | 0.07 |
| 1.41 | 100.00 | 102.36 | -9.40 | 0.03 | 1.90 | 100.00 | 90.78 | -13.40 | 0.14 |
| 1.43 | 100.00 | 102.30 | -9.00 | 0.09 | 1.98 | 100.00 | 90.75 | -13.00 | 0.07 |
| 1.44 | 100.00 | 102.28 | -8.60 | 0.03 | 1.99 | 99.99 | 90.70 | -12.60 | 0.20 |
| 1.45 | 100.00 | 102.25 | -8.20 | 0.09 | 2.00 | 99.99 | 90.67 | -12.20 | 0.38 |
| 1.46 | 99.99 | 102.22 | -7.80 | 0.12 | 2.04 | 99.98 | 90.65 | -11.80 | 0.51 |
| 1.47 | 99.99 | 102.19 | -7.40 | 0.25 | 2.05 | 99.98 | 90.59 | -11.40 | 0.68 |
| 1.48 | 99.99 | 102.17 | -7.00 | 0.34 | 2.06 | 99.98 | 90.57 | -11.00 | 1.02 |
| 1.49 | 99.98 | 102.14 | -6.60 | 0.50 | 2.07 | 99.98 | 90.54 | -10.60 | 0.99 |
| 1.50 | 99.98 | 102.11 | -6.20 | 1.22 | 2.08 | 99.97 | 90.51 | -10.20 | 0.82 |
| 1.51 | 99.97 | 102.08 | -5.80 | 1.44 | 2.10 | 99.97 | 90.48 | -9.80 | 1.40 |
| 1.52 | 99.96 | 102.05 | -5.40 | 2.09 | 2.11 | 99.96 | 90.46 | -9.40 | 1.19 |
| 1.53 | 99.95 | 102.03 | -5.00 | 2.81 | 2.12 | 99.96 | 90.43 | -9.00 | 1.71 |
| 1.54 | 99.95 | 102.00 | -4.60 | 4.43 | 2.16 | 99.94 | 90.40 | -8.60 | 1.30 |
| 1.55 | 99.94 | 101.97 | -4.20 | 6.21 | 2.17 | 99.93 | 90.38 | -8.20 | 1.33 |
| 1.56 | 99.93 | 101.94 | -3.80 | 6.46 | 2.18 | 99.92 | 90.35 | -7.80 | 1.13 |
| 1.57 | 99.92 | 101.92 | -3.40 | 9.12 | 2.20 | 99.90 | 90.32 | -7.40 | 1.06 |
| 1.58 | 99.91 | 101.89 | -3.00 | 11.87 | 2.22 | 99.87 | 90.27 | -7.00 | 0.85 |
| 1.59 | 99.89 | 101.86 | -2.60 | 11.46 | 2.23 | 99.86 | 90.24 | -6.60 | 0.89 |
| 1.61 | 99.87 | 101.81 | -2.20 | 13.52 | 2.25 | 99.85 | 90.21 | -6.20 | 0.79 |
| 1.62 | 99.84 | 101.78 | -1.80 | 11.56 | 2.28 | 99.81 | 90.19 | -5.80 | 0.85 |
| 1.64 | 99.79 | 101.72 | -1.40 | 8.09 | 2.29 | 99.79 | 90.16 | -5.40 | 1.50 |
| 1.65 | 99.77 | 101.69 | -1.00 | 4.65 | 2.30 | 99.77 | 90.13 | -5.00 | 1.50 |
| 1.66 | 99.72 | 101.67 | -0.60 | 2.09 | 2.31 | 99.76 | 90.11 | -4.60 | 1.43 |
| 1.69 | 99.63 | 101.58 | -0.20 | 1.50 | 2.32 | 99.75 | 90.08 | -4.20 | 2.56 |
| 1.70 | 99.62 | 101.56 | 0.20 | 0.00 | 2.34 | 99.72 | 90.05 | -3.80 | 2.08 |
| 1.72 | 99.56 | 101.50 |  |  | 2.36 | 99.71 | 90.02 | -3.40 | 3.58 |
| 1.73 | 99.54 | 101.47 |  |  | 2.37 | 99.69 | 90.00 | -3.00 | 4.40 |
| 1.74 | 99.51 | 101.44 |  |  | 2.38 | 99.69 | 89.97 | -2.60 | 6.38 |
| 1.75 | 99.48 | 101.42 |  |  | 2.39 | 99.69 | 89.94 | -2.20 | 9.49 |
| 1.76 | 99.45 | 101.39 |  |  | 2.40 | 99.67 | 89.92 | -1.80 | 11.81 |
| 1.77 | 99.43 | 101.36 |  |  | 2.41 | 99.66 | 89.89 | -1.40 | 13.08 |
| 1.78 | 99.41 | 101.33 |  |  | 2.42 | 99.65 | 89.86 | -1.00 | 11.20 |
| 1.79 | 99.38 | 101.31 |  |  | 2.43 | 99.63 | 89.83 | -0.60 | 9.32 |
| 1.80 | 99.36 | 101.28 |  |  | 2.44 | 99.62 | 89.81 | -0.20 | 4.27 |
| 1.81 | 99.33 | 101.25 |  |  | 2.45 | 99.61 | 89.78 | 0.20 | 0.00 |
| 1.82 | 99.29 | 101.22 |  |  | 2.46 | 99.60 | 89.75 |  |  |
| 1.83 | 99.27 | 101.19 |  |  | 2.48 | 99.58 | 89.73 |  |  |
| 1.84 | 99.26 | 101.17 |  |  | 2.49 | 99.57 | 89.70 |  |  |
| 1.85 | 99.23 | 101.14 |  |  | 2.52 | 99.52 | 89.67 |  |  |
| 1.86 | 99.20 | 101.11 |  |  | 2.53 | 99.51 | 89.65 |  |  |
| 1.87 | 99.17 | 101.08 |  |  | 2.54 | 99.50 | 89.62 |  |  |
| 1.88 | 99.13 | 101.06 |  |  | 2.55 | 99.47 | 89.59 |  |  |
| 1.89 | 99.10 | 101.03 |  |  | 2.56 | 99.45 | 89.56 |  |  |
| 1.90 | 99.07 | 101.00 |  |  | 2.57 | 99.43 | 89.54 |  |  |
| 1.91 | 99.03 | 100.97 |  |  | 2.59 | 99.40 | 89.51 |  |  |
| 1.92 | 98.99 | 100.94 |  |  | 2.60 | 99.36 | 89.48 |  |  |
| 1.93 | 98.97 | 100.92 |  |  | 2.61 | 99.34 | 89.46 |  |  |
| 1.94 | 98.93 | 100.89 |  |  | 2.62 | 99.33 | 89.43 |  |  |
| 1.95 | 98.91 | 100.86 |  |  | 2.63 | 99.31 | 89.40 |  |  |
| 1.96 | 98.88 | 100.83 |  |  | 2.64 | 99.30 | 89.37 |  |  |
| 1.97 | 98.83 | 100.81 |  |  | 2.65 | 99.29 | 89.35 |  |  |
| 1.98 | 98.80 | 100.78 |  |  | 2.66 | 99.26 | 89.29 |  |  |
| 1.99 | 98.76 | 100.75 |  |  | 2.67 | 99.23 | 89.27 |  |  |
| 2.00 | 98.72 | 100.72 |  |  | 2.68 | 99.20 | 89.24 |  |  |
| 2.01 | 98.67 | 100.69 |  |  | 2.69 | 99.18 | 89.19 |  |  |
| 2.02 | 98.61 | 100.67 |  |  | 2.70 | 99.17 | 89.16 |  |  |
| 2.03 | 98.56 | 100.64 |  |  | 2.71 | 99.15 | 89.13 |  |  |
| 2.04 | 98.52 | 100.61 |  |  | 2.72 | 99.13 | 89.10 |  |  |
| 2.05 | 98.49 | 100.58 |  |  | 2.73 | 99.11 | 89.08 |  |  |
| 2.07 | 98.41 | 100.53 |  |  | 2.74 | 99.09 | 89.05 |  |  |
| 2.08 | 98.37 | 100.50 |  |  | 2.75 | 99.09 | 89.02 |  |  |
| 2.09 | 98.34 | 100.47 |  |  | 2.76 | 99.06 | 89.00 |  |  |
| 2.10 | 98.30 | 100.44 |  |  | 2.77 | 99.05 | 88.97 |  |  |
| 2.11 | 98.25 | 100.42 |  |  | 2.78 | 99.03 | 88.94 |  |  |
| 2.12 | 98.19 | 100.39 |  |  | 2.79 | 99.02 | 88.91 |  |  |
| 2.13 | 98.14 | 100.36 |  |  | 2.80 | 98.99 | 88.89 |  |  |
| 2.14 | 98.12 | 100.33 |  |  | 2.81 | 98.98 | 88.86 |  |  |
| 2.15 | 98.06 | 100.31 |  |  | 2.82 | 98.97 | 88.83 |  |  |
| 2.16 | 98.02 | 100.28 |  |  | 2.83 | 98.95 | 88.81 |  |  |
| 2.17 | 97.98 | 100.25 |  |  | 2.85 | 98.94 | 88.78 |  |  |
| 2.18 | 97.92 | 100.22 |  |  | 2.86 | 98.93 | 88.75 |  |  |
| 2.19 | 97.87 | 100.20 |  |  | 2.87 | 98.91 | 88.73 |  |  |
| 2.20 | 97.84 | 100.17 |  |  | 2.88 | 98.90 | 88.70 |  |  |
| 2.21 | 97.81 | 100.14 |  |  | 2.89 | 98.88 | 88.67 |  |  |
| 2.22 | 97.77 | 100.11 |  |  | 2.90 | 98.85 | 88.64 |  |  |
| 2.23 | 97.72 | 100.08 |  |  | 2.91 | 98.83 | 88.62 |  |  |
| 2.25 | 97.64 | 100.03 |  |  | 2.92 | 98.82 | 88.59 |  |  |
| 2.26 | 97.60 | 100.00 |  |  | 2.93 | 98.80 | 88.56 |  |  |
| 2.27 | 97.57 | 99.97 |  |  | 2.94 | 98.79 | 88.54 |  |  |
| 2.28 | 97.53 | 99.95 |  |  | 2.95 | 98.77 | 88.51 |  |  |
| 2.29 | 97.48 | 99.92 |  |  | 2.97 | 98.73 | 88.45 |  |  |
| 2.30 | 97.45 | 99.89 |  |  | 2.98 | 98.72 | 88.43 |  |  |
| 2.31 | 97.40 | 99.86 |  |  | 2.99 | 98.70 | 88.40 |  |  |
| 2.32 | 97.35 | 99.83 |  |  | 3.00 | 98.69 | 88.37 |  |  |
| 2.33 | 97.31 | 99.81 |  |  | 3.01 | 98.68 | 88.35 |  |  |
| 2.34 | 97.27 | 99.78 |  |  | 3.02 | 98.66 | 88.32 |  |  |
| 2.35 | 97.22 | 99.75 |  |  | 3.03 | 98.64 | 88.29 |  |  |
| 2.36 | 97.17 | 99.72 |  |  | 3.04 | 98.62 | 88.27 |  |  |
| 2.37 | 97.11 | 99.70 |  |  | 3.05 | 98.59 | 88.24 |  |  |
| 2.39 | 97.00 | 99.64 |  |  | 3.06 | 98.57 | 88.21 |  |  |
| 2.40 | 96.96 | 99.61 |  |  | 3.07 | 98.54 | 88.18 |  |  |
| 2.41 | 96.90 | 99.58 |  |  | 3.08 | 98.51 | 88.16 |  |  |
| 2.42 | 96.84 | 99.56 |  |  | 3.09 | 98.48 | 88.13 |  |  |
| 2.43 | 96.80 | 99.53 |  |  | 3.10 | 98.45 | 88.10 |  |  |
| 2.44 | 96.74 | 99.50 |  |  | 3.11 | 98.44 | 88.08 |  |  |
| 2.45 | 96.71 | 99.47 |  |  | 3.12 | 98.42 | 88.05 |  |  |
| 2.46 | 96.67 | 99.45 |  |  | 3.14 | 98.38 | 88.02 |  |  |
| 2.47 | 96.62 | 99.42 |  |  | 3.15 | 98.37 | 88.00 |  |  |
| 2.48 | 96.58 | 99.39 |  |  | 3.17 | 98.32 | 87.97 |  |  |
| 2.49 | 96.55 | 99.36 |  |  | 3.18 | 98.31 | 87.94 |  |  |
| 2.50 | 96.53 | 99.33 |  |  | 3.20 | 98.28 | 87.91 |  |  |
| 2.51 | 96.50 | 99.31 |  |  | 3.21 | 98.26 | 87.89 |  |  |
| 2.52 | 96.46 | 99.28 |  |  | 3.22 | 98.23 | 87.86 |  |  |
| 2.53 | 96.42 | 99.25 |  |  | 3.23 | 98.22 | 87.83 |  |  |
| 2.54 | 96.39 | 99.22 |  |  | 3.24 | 98.20 | 87.81 |  |  |
| 2.55 | 96.36 | 99.20 |  |  | 3.25 | 98.18 | 87.78 |  |  |
| 2.57 | 96.32 | 99.14 |  |  | 3.26 | 98.16 | 87.75 |  |  |
| 2.58 | 96.26 | 99.11 |  |  | 3.27 | 98.15 | 87.72 |  |  |
| 2.59 | 96.23 | 99.08 |  |  | 3.28 | 98.13 | 87.70 |  |  |
| 2.60 | 96.20 | 99.06 |  |  | 3.29 | 98.12 | 87.67 |  |  |
| 2.61 | 96.16 | 99.03 |  |  | 3.32 | 98.06 | 87.64 |  |  |
| 2.62 | 96.14 | 99.00 |  |  | 3.34 | 98.03 | 87.62 |  |  |
| 2.63 | 96.12 | 98.97 |  |  | 3.35 | 98.01 | 87.59 |  |  |
| 2.64 | 96.09 | 98.95 |  |  | 3.36 | 97.99 | 87.56 |  |  |
| 2.65 | 96.05 | 98.92 |  |  | 3.37 | 97.99 | 87.54 |  |  |
| 2.66 | 96.02 | 98.89 |  |  | 3.38 | 97.97 | 87.51 |  |  |
| 2.67 | 96.00 | 98.86 |  |  | 3.39 | 97.94 | 87.48 |  |  |
| 2.68 | 95.98 | 98.83 |  |  | 3.40 | 97.93 | 87.45 |  |  |
| 2.70 | 95.90 | 98.78 |  |  | 3.41 | 97.90 | 87.43 |  |  |
| 2.71 | 95.88 | 98.75 |  |  | 3.42 | 97.89 | 87.37 |  |  |
| 2.73 | 95.83 | 98.70 |  |  | 3.43 | 97.87 | 87.35 |  |  |
| 2.74 | 95.79 | 98.67 |  |  | 3.44 | 97.85 | 87.32 |  |  |
| 2.75 | 95.76 | 98.64 |  |  | 3.45 | 97.83 | 87.29 |  |  |
| 2.76 | 95.74 | 98.61 |  |  | 3.46 | 97.81 | 87.26 |  |  |
| 2.77 | 95.72 | 98.58 |  |  | 3.48 | 97.78 | 87.24 |  |  |
| 2.78 | 95.68 | 98.56 |  |  | 3.49 | 97.76 | 87.21 |  |  |
| 2.79 | 95.66 | 98.53 |  |  | 3.50 | 97.73 | 87.16 |  |  |
| 2.80 | 95.65 | 98.50 |  |  | 3.51 | 97.71 | 87.13 |  |  |
| 2.81 | 95.62 | 98.47 |  |  | 3.52 | 97.69 | 87.10 |  |  |
| 2.82 | 95.59 | 98.45 |  |  | 3.53 | 97.66 | 87.08 |  |  |
| 2.83 | 95.57 | 98.42 |  |  | 3.54 | 97.63 | 87.05 |  |  |
| 2.84 | 95.54 | 98.39 |  |  | 3.56 | 97.57 | 87.02 |  |  |
| 2.85 | 95.51 | 98.36 |  |  | 3.57 | 97.54 | 86.99 |  |  |
| 2.86 | 95.49 | 98.34 |  |  | 3.60 | 97.47 | 86.97 |  |  |
| 2.87 | 95.44 | 98.31 |  |  | 3.61 | 97.44 | 86.94 |  |  |
| 2.88 | 95.41 | 98.28 |  |  | 3.62 | 97.41 | 86.91 |  |  |
| 2.89 | 95.39 | 98.25 |  |  | 3.63 | 97.38 | 86.89 |  |  |
| 2.91 | 95.33 | 98.20 |  |  | 3.64 | 97.36 | 86.86 |  |  |
| 2.92 | 95.30 | 98.17 |  |  | 3.65 | 97.34 | 86.83 |  |  |
| 2.93 | 95.27 | 98.14 |  |  | 3.66 | 97.32 | 86.80 |  |  |
| 2.94 | 95.23 | 98.11 |  |  | 3.67 | 97.30 | 86.78 |  |  |
| 2.96 | 95.18 | 98.06 |  |  | 3.68 | 97.28 | 86.75 |  |  |
| 2.97 | 95.15 | 98.03 |  |  | 3.70 | 97.24 | 86.72 |  |  |
| 2.98 | 95.13 | 98.00 |  |  | 3.71 | 97.20 | 86.70 |  |  |
| 2.99 | 95.09 | 97.97 |  |  | 3.72 | 97.19 | 86.67 |  |  |
| 3.00 | 95.06 | 97.95 |  |  | 3.73 | 97.14 | 86.64 |  |  |
| 3.01 | 95.04 | 97.92 |  |  | 3.74 | 97.10 | 86.62 |  |  |
| 3.02 | 95.02 | 97.89 |  |  | 3.75 | 97.08 | 86.56 |  |  |
| 3.03 | 94.98 | 97.86 |  |  | 3.76 | 97.06 | 86.53 |  |  |
| 3.04 | 94.95 | 97.84 |  |  | 3.79 | 96.97 | 86.51 |  |  |
| 3.05 | 94.92 | 97.81 |  |  | 3.80 | 96.95 | 86.48 |  |  |
| 3.06 | 94.90 | 97.78 |  |  | 3.81 | 96.91 | 86.43 |  |  |
| 3.07 | 94.88 | 97.75 |  |  | 3.83 | 96.86 | 86.40 |  |  |
| 3.08 | 94.86 | 97.72 |  |  | 3.84 | 96.85 | 86.37 |  |  |
| 3.09 | 94.84 | 97.70 |  |  | 3.85 | 96.82 | 86.34 |  |  |
| 3.10 | 94.80 | 97.67 |  |  | 3.86 | 96.78 | 86.32 |  |  |
| 3.11 | 94.79 | 97.64 |  |  | 3.87 | 96.75 | 86.29 |  |  |
| 3.12 | 94.76 | 97.61 |  |  | 3.88 | 96.73 | 86.26 |  |  |
| 3.13 | 94.74 | 97.59 |  |  | 3.89 | 96.71 | 86.24 |  |  |
| 3.14 | 94.71 | 97.56 |  |  | 3.90 | 96.69 | 86.21 |  |  |
| 3.15 | 94.68 | 97.53 |  |  | 3.91 | 96.67 | 86.16 |  |  |
| 3.16 | 94.66 | 97.50 |  |  | 3.92 | 96.66 | 86.13 |  |  |
| 3.17 | 94.63 | 97.47 |  |  | 3.93 | 96.63 | 86.10 |  |  |
| 3.18 | 94.61 | 97.45 |  |  | 3.94 | 96.61 | 86.07 |  |  |
| 3.19 | 94.59 | 97.42 |  |  | 3.96 | 96.57 | 86.05 |  |  |
| 3.20 | 94.58 | 97.39 |  |  | 3.97 | 96.56 | 86.02 |  |  |
| 3.21 | 94.54 | 97.36 |  |  | 3.98 | 96.53 | 85.99 |  |  |
| 3.22 | 94.51 | 97.34 |  |  | 3.99 | 96.51 | 85.97 |  |  |
| 3.23 | 94.48 | 97.31 |  |  | 4.00 | 96.49 | 85.94 |  |  |
| 3.24 | 94.46 | 97.28 |  |  | 4.01 | 96.48 | 85.91 |  |  |
| 3.25 | 94.44 | 97.25 |  |  | 4.02 | 96.44 | 85.88 |  |  |
| 3.26 | 94.40 | 97.22 |  |  | 4.03 | 96.43 | 85.86 |  |  |
| 3.27 | 94.37 | 97.20 |  |  | 4.04 | 96.40 | 85.80 |  |  |
| 3.28 | 94.35 | 97.17 |  |  | 4.06 | 96.36 | 85.78 |  |  |
| 3.29 | 94.33 | 97.14 |  |  | 4.07 | 96.34 | 85.75 |  |  |
| 3.30 | 94.31 | 97.11 |  |  | 4.08 | 96.32 | 85.72 |  |  |
| 3.31 | 94.28 | 97.09 |  |  | 4.09 | 96.30 | 85.70 |  |  |
| 3.32 | 94.26 | 97.06 |  |  | 4.10 | 96.28 | 85.67 |  |  |
| 3.33 | 94.23 | 97.03 |  |  | 4.11 | 96.26 | 85.64 |  |  |
| 3.34 | 94.20 | 97.00 |  |  | 4.12 | 96.24 | 85.61 |  |  |
| 3.35 | 94.17 | 96.97 |  |  | 4.13 | 96.22 | 85.59 |  |  |
| 3.38 | 94.11 | 96.89 |  |  | 4.14 | 96.20 | 85.56 |  |  |
| 3.39 | 94.07 | 96.86 |  |  | 4.16 | 96.16 | 85.53 |  |  |
| 3.41 | 94.03 | 96.81 |  |  | 4.17 | 96.14 | 85.51 |  |  |
| 3.42 | 94.00 | 96.78 |  |  | 4.18 | 96.11 | 85.48 |  |  |
| 3.43 | 93.98 | 96.75 |  |  | 4.19 | 96.08 | 85.45 |  |  |
| 3.44 | 93.95 | 96.73 |  |  | 4.20 | 96.07 | 85.40 |  |  |
| 3.45 | 93.93 | 96.70 |  |  | 4.21 | 96.04 | 85.37 |  |  |
| 3.47 | 93.91 | 96.64 |  |  | 4.22 | 96.02 | 85.34 |  |  |
| 3.48 | 93.88 | 96.61 |  |  | 4.23 | 96.01 | 85.32 |  |  |
| 3.49 | 93.85 | 96.59 |  |  | 4.24 | 95.99 | 85.29 |  |  |
| 3.50 | 93.84 | 96.56 |  |  | 4.25 | 95.97 | 85.26 |  |  |
| 3.51 | 93.80 | 96.53 |  |  | 4.26 | 95.94 | 85.21 |  |  |
| 3.52 | 93.79 | 96.50 |  |  | 4.27 | 95.93 | 85.18 |  |  |
| 3.53 | 93.77 | 96.48 |  |  | 4.28 | 95.91 | 85.15 |  |  |
| 3.54 | 93.74 | 96.45 |  |  | 4.29 | 95.88 | 85.13 |  |  |
| 3.56 | 93.71 | 96.39 |  |  | 4.30 | 95.85 | 85.10 |  |  |
| 3.57 | 93.67 | 96.36 |  |  | 4.31 | 95.84 | 85.07 |  |  |
| 3.58 | 93.66 | 96.34 |  |  | 4.32 | 95.82 | 85.05 |  |  |
| 3.59 | 93.63 | 96.31 |  |  | 4.33 | 95.81 | 85.02 |  |  |
| 3.60 | 93.60 | 96.28 |  |  | 4.34 | 95.78 | 84.99 |  |  |
| 3.61 | 93.58 | 96.25 |  |  | 4.36 | 95.75 | 84.96 |  |  |
| 3.63 | 93.53 | 96.20 |  |  | 4.37 | 95.72 | 84.94 |  |  |
| 3.64 | 93.50 | 96.17 |  |  | 4.38 | 95.71 | 84.91 |  |  |
| 3.65 | 93.48 | 96.14 |  |  | 4.39 | 95.69 | 84.88 |  |  |
| 3.66 | 93.47 | 96.11 |  |  | 4.40 | 95.66 | 84.86 |  |  |
| 3.67 | 93.44 | 96.09 |  |  | 4.41 | 95.65 | 84.80 |  |  |
| 3.68 | 93.41 | 96.06 |  |  | 4.42 | 95.64 | 84.78 |  |  |
| 3.69 | 93.38 | 96.03 |  |  | 4.43 | 95.61 | 84.75 |  |  |
| 3.70 | 93.36 | 96.00 |  |  | 4.44 | 95.60 | 84.72 |  |  |
| 3.71 | 93.33 | 95.98 |  |  | 4.46 | 95.55 | 84.69 |  |  |
| 3.72 | 93.31 | 95.95 |  |  | 4.47 | 95.54 | 84.67 |  |  |
| 3.73 | 93.27 | 95.92 |  |  | 4.48 | 95.52 | 84.64 |  |  |
| 3.74 | 93.26 | 95.89 |  |  | 4.49 | 95.50 | 84.61 |  |  |
| 3.75 | 93.23 | 95.86 |  |  | 4.50 | 95.49 | 84.59 |  |  |
| 3.76 | 93.22 | 95.84 |  |  | 4.51 | 95.47 | 84.56 |  |  |
| 3.77 | 93.20 | 95.81 |  |  | 4.53 | 95.42 | 84.53 |  |  |
| 3.78 | 93.18 | 95.78 |  |  | 4.54 | 95.40 | 84.48 |  |  |
| 3.79 | 93.16 | 95.75 |  |  | 4.55 | 95.38 | 84.45 |  |  |
| 3.80 | 93.15 | 95.73 |  |  | 4.56 | 95.36 | 84.42 |  |  |
| 3.82 | 93.09 | 95.67 |  |  | 4.57 | 95.33 | 84.40 |  |  |
| 3.83 | 93.07 | 95.64 |  |  | 4.58 | 95.31 | 84.32 |  |  |
| 3.84 | 93.05 | 95.61 |  |  | 4.60 | 95.28 | 84.29 |  |  |
| 3.85 | 93.02 | 95.59 |  |  | 4.62 | 95.22 | 84.26 |  |  |
| 3.87 | 92.99 | 95.53 |  |  | 4.63 | 95.21 | 84.23 |  |  |
| 3.88 | 92.97 | 95.50 |  |  | 4.64 | 95.18 | 84.21 |  |  |
| 3.89 | 92.94 | 95.48 |  |  | 4.65 | 95.15 | 84.18 |  |  |
| 3.90 | 92.92 | 95.45 |  |  | 4.66 | 95.11 | 84.15 |  |  |
| 3.91 | 92.90 | 95.42 |  |  | 4.67 | 95.07 | 84.13 |  |  |
| 3.92 | 92.87 | 95.39 |  |  | 4.68 | 95.04 | 84.07 |  |  |
| 3.93 | 92.82 | 95.36 |  |  | 4.69 | 95.01 | 84.04 |  |  |
| 3.94 | 92.80 | 95.34 |  |  | 4.70 | 94.98 | 84.02 |  |  |
| 3.95 | 92.78 | 95.31 |  |  | 4.71 | 94.96 | 83.99 |  |  |
| 3.96 | 92.76 | 95.28 |  |  | 4.72 | 94.94 | 83.96 |  |  |
| 3.97 | 92.74 | 95.25 |  |  | 4.73 | 94.92 | 83.94 |  |  |
| 3.98 | 92.70 | 95.23 |  |  | 4.74 | 94.89 | 83.91 |  |  |
| 3.99 | 92.67 | 95.20 |  |  | 4.75 | 94.85 | 83.88 |  |  |
| 4.00 | 92.64 | 95.17 |  |  | 4.77 | 94.81 | 83.86 |  |  |
| 4.01 | 92.62 | 95.14 |  |  | 4.78 | 94.77 | 83.83 |  |  |
| 4.02 | 92.59 | 95.11 |  |  | 4.79 | 94.75 | 83.80 |  |  |
| 4.03 | 92.57 | 95.09 |  |  | 4.80 | 94.74 | 83.77 |  |  |
| 4.04 | 92.56 | 95.06 |  |  | 4.81 | 94.70 | 83.75 |  |  |
| 4.05 | 92.53 | 95.03 |  |  | 4.82 | 94.68 | 83.72 |  |  |
| 4.06 | 92.49 | 95.00 |  |  | 4.83 | 94.65 | 83.69 |  |  |
| 4.07 | 92.46 | 94.98 |  |  | 4.84 | 94.63 | 83.67 |  |  |
| 4.08 | 92.43 | 94.95 |  |  | 4.85 | 94.62 | 83.64 |  |  |
| 4.09 | 92.39 | 94.92 |  |  | 4.86 | 94.58 | 83.61 |  |  |
| 4.10 | 92.37 | 94.89 |  |  | 4.88 | 94.53 | 83.58 |  |  |
| 4.11 | 92.35 | 94.87 |  |  | 4.89 | 94.51 | 83.56 |  |  |
| 4.12 | 92.33 | 94.84 |  |  | 4.90 | 94.50 | 83.53 |  |  |
| 4.13 | 92.30 | 94.81 |  |  | 4.91 | 94.48 | 83.50 |  |  |
| 4.14 | 92.28 | 94.78 |  |  | 4.92 | 94.47 | 83.48 |  |  |
| 4.15 | 92.26 | 94.75 |  |  | 4.93 | 94.44 | 83.45 |  |  |
| 4.17 | 92.21 | 94.70 |  |  | 4.94 | 94.43 | 83.42 |  |  |
| 4.18 | 92.19 | 94.67 |  |  | 4.95 | 94.40 | 83.40 |  |  |
| 4.19 | 92.16 | 94.64 |  |  | 4.96 | 94.38 | 83.34 |  |  |
| 4.20 | 92.14 | 94.62 |  |  | 4.97 | 94.37 | 83.31 |  |  |
| 4.22 | 92.10 | 94.56 |  |  | 4.98 | 94.36 | 83.29 |  |  |
| 4.24 | 92.05 | 94.50 |  |  | 4.99 | 94.33 | 83.26 |  |  |
| 4.25 | 92.03 | 94.48 |  |  | 5.00 | 94.30 | 83.23 |  |  |
| 4.26 | 92.01 | 94.45 |  |  | 5.01 | 94.29 | 83.21 |  |  |
| 4.27 | 91.99 | 94.42 |  |  | 5.02 | 94.27 | 83.18 |  |  |
| 4.28 | 91.97 | 94.39 |  |  | 5.03 | 94.25 | 83.15 |  |  |
| 4.29 | 91.94 | 94.37 |  |  | 5.04 | 94.24 | 83.12 |  |  |
| 4.30 | 91.93 | 94.34 |  |  | 5.05 | 94.22 | 83.10 |  |  |
| 4.31 | 91.91 | 94.31 |  |  | 5.06 | 94.20 | 83.07 |  |  |
| 4.32 | 91.87 | 94.28 |  |  | 5.07 | 94.19 | 83.04 |  |  |
| 4.35 | 91.82 | 94.20 |  |  | 5.08 | 94.17 | 83.02 |  |  |
| 4.36 | 91.80 | 94.17 |  |  | 5.10 | 94.13 | 82.99 |  |  |
| 4.37 | 91.77 | 94.14 |  |  | 5.11 | 94.11 | 82.96 |  |  |
| 4.38 | 91.75 | 94.12 |  |  | 5.12 | 94.09 | 82.94 |  |  |
| 4.39 | 91.74 | 94.09 |  |  | 5.13 | 94.07 | 82.91 |  |  |
| 4.41 | 91.69 | 94.03 |  |  | 5.14 | 94.06 | 82.88 |  |  |
| 4.42 | 91.66 | 94.00 |  |  | 5.15 | 94.02 | 82.85 |  |  |
| 4.43 | 91.64 | 93.98 |  |  | 5.16 | 94.01 | 82.83 |  |  |
| 4.44 | 91.61 | 93.95 |  |  | 5.17 | 93.99 | 82.80 |  |  |
| 4.45 | 91.59 | 93.92 |  |  | 5.18 | 93.98 | 82.77 |  |  |
| 4.46 | 91.57 | 93.89 |  |  | 5.20 | 93.95 | 82.75 |  |  |
| 4.47 | 91.56 | 93.87 |  |  | 5.23 | 93.90 | 82.72 |  |  |
| 4.48 | 91.53 | 93.84 |  |  | 5.25 | 93.87 | 82.69 |  |  |
| 4.49 | 91.51 | 93.81 |  |  | 5.26 | 93.85 | 82.66 |  |  |
| 4.50 | 91.48 | 93.78 |  |  | 5.27 | 93.84 | 82.64 |  |  |
| 4.51 | 91.47 | 93.75 |  |  | 5.28 | 93.83 | 82.61 |  |  |
| 4.52 | 91.44 | 93.73 |  |  | 5.30 | 93.78 | 82.58 |  |  |
| 4.53 | 91.42 | 93.70 |  |  | 5.31 | 93.77 | 82.56 |  |  |
| 4.54 | 91.40 | 93.67 |  |  | 5.32 | 93.75 | 82.53 |  |  |
| 4.55 | 91.38 | 93.64 |  |  | 5.33 | 93.73 | 82.50 |  |  |
| 4.56 | 91.36 | 93.62 |  |  | 5.34 | 93.70 | 82.48 |  |  |
| 4.57 | 91.34 | 93.59 |  |  | 5.36 | 93.68 | 82.45 |  |  |
| 4.58 | 91.32 | 93.56 |  |  | 5.37 | 93.66 | 82.42 |  |  |
| 4.59 | 91.30 | 93.53 |  |  | 5.38 | 93.65 | 82.39 |  |  |
| 4.60 | 91.27 | 93.50 |  |  | 5.39 | 93.64 | 82.37 |  |  |
| 4.64 | 91.20 | 93.39 |  |  | 5.40 | 93.62 | 82.34 |  |  |
| 4.65 | 91.18 | 93.37 |  |  | 5.41 | 93.61 | 82.31 |  |  |
| 4.66 | 91.16 | 93.34 |  |  | 5.43 | 93.57 | 82.29 |  |  |
| 4.67 | 91.15 | 93.31 |  |  | 5.44 | 93.55 | 82.26 |  |  |
| 4.68 | 91.12 | 93.28 |  |  | 5.46 | 93.52 | 82.23 |  |  |
| 4.69 | 91.10 | 93.26 |  |  | 5.47 | 93.52 | 82.20 |  |  |
| 4.70 | 91.08 | 93.23 |  |  | 5.48 | 93.50 | 82.18 |  |  |
| 4.71 | 91.05 | 93.20 |  |  | 5.49 | 93.49 | 82.15 |  |  |
| 4.72 | 91.02 | 93.17 |  |  | 5.50 | 93.47 | 82.12 |  |  |
| 4.74 | 90.99 | 93.12 |  |  | 5.52 | 93.44 | 82.10 |  |  |
| 4.76 | 90.94 | 93.06 |  |  | 5.54 | 93.38 | 82.07 |  |  |
| 4.77 | 90.94 | 93.03 |  |  | 5.55 | 93.38 | 82.04 |  |  |
| 4.78 | 90.92 | 93.01 |  |  | 5.56 | 93.36 | 82.02 |  |  |
| 4.79 | 90.90 | 92.98 |  |  | 5.57 | 93.34 | 81.99 |  |  |
| 4.80 | 90.88 | 92.95 |  |  | 5.58 | 93.33 | 81.96 |  |  |
| 4.81 | 90.86 | 92.92 |  |  | 5.59 | 93.30 | 81.93 |  |  |
| 4.82 | 90.85 | 92.89 |  |  | 5.60 | 93.27 | 81.91 |  |  |
| 4.83 | 90.83 | 92.87 |  |  | 5.61 | 93.27 | 81.85 |  |  |
| 4.84 | 90.81 | 92.84 |  |  | 5.62 | 93.25 | 81.83 |  |  |
| 4.85 | 90.79 | 92.81 |  |  | 5.63 | 93.24 | 81.80 |  |  |
| 4.87 | 90.76 | 92.76 |  |  | 5.64 | 93.21 | 81.77 |  |  |
| 4.88 | 90.73 | 92.73 |  |  | 5.65 | 93.20 | 81.72 |  |  |
| 4.89 | 90.71 | 92.70 |  |  | 5.66 | 93.17 | 81.69 |  |  |
| 4.90 | 90.70 | 92.67 |  |  | 5.67 | 93.16 | 81.66 |  |  |
| 4.91 | 90.68 | 92.64 |  |  | 5.68 | 93.14 | 81.64 |  |  |
| 4.93 | 90.64 | 92.59 |  |  | 5.69 | 93.12 | 81.61 |  |  |
| 4.94 | 90.60 | 92.56 |  |  | 5.71 | 93.07 | 81.58 |  |  |
| 4.95 | 90.57 | 92.53 |  |  | 5.73 | 93.04 | 81.56 |  |  |
| 4.96 | 90.54 | 92.51 |  |  | 5.74 | 93.02 | 81.53 |  |  |
| 4.97 | 90.53 | 92.48 |  |  | 5.75 | 93.00 | 81.50 |  |  |
| 4.98 | 90.50 | 92.45 |  |  | 5.76 | 92.98 | 81.47 |  |  |
| 4.99 | 90.48 | 92.42 |  |  | 5.77 | 92.95 | 81.45 |  |  |
| 5.00 | 90.46 | 92.39 |  |  | 5.80 | 92.89 | 81.42 |  |  |
| 5.01 | 90.44 | 92.37 |  |  | 5.81 | 92.87 | 81.39 |  |  |
| 5.02 | 90.41 | 92.34 |  |  | 5.82 | 92.85 | 81.37 |  |  |
| 5.03 | 90.39 | 92.31 |  |  | 5.83 | 92.83 | 81.34 |  |  |
| 5.05 | 90.36 | 92.26 |  |  | 5.84 | 92.80 | 81.31 |  |  |
| 5.06 | 90.33 | 92.23 |  |  | 5.85 | 92.77 | 81.28 |  |  |
| 5.07 | 90.31 | 92.20 |  |  | 5.87 | 92.73 | 81.26 |  |  |
| 5.09 | 90.25 | 92.14 |  |  | 5.88 | 92.69 | 81.23 |  |  |
| 5.10 | 90.23 | 92.12 |  |  | 5.89 | 92.66 | 81.20 |  |  |
| 5.11 | 90.21 | 92.09 |  |  | 5.90 | 92.63 | 81.18 |  |  |
| 5.12 | 90.19 | 92.06 |  |  | 5.92 | 92.57 | 81.15 |  |  |
| 5.13 | 90.18 | 92.03 |  |  | 5.93 | 92.56 | 81.12 |  |  |
| 5.14 | 90.17 | 92.01 |  |  | 5.94 | 92.53 | 81.10 |  |  |
| 5.15 | 90.15 | 91.98 |  |  | 5.95 | 92.52 | 81.07 |  |  |
| 5.16 | 90.14 | 91.95 |  |  | 5.96 | 92.50 | 81.04 |  |  |
| 5.17 | 90.11 | 91.92 |  |  | 5.97 | 92.49 | 81.01 |  |  |
| 5.18 | 90.09 | 91.89 |  |  | 5.98 | 92.47 | 80.99 |  |  |
| 5.19 | 90.08 | 91.87 |  |  | 5.99 | 92.45 | 80.96 |  |  |
| 5.21 | 90.02 | 91.81 |  |  | 6.00 | 92.44 | 80.93 |  |  |
| 5.22 | 90.00 | 91.78 |  |  | 6.01 | 92.41 | 80.85 |  |  |
| 5.23 | 89.99 | 91.76 |  |  | 6.02 | 92.39 | 80.82 |  |  |
| 5.25 | 89.96 | 91.70 |  |  | 6.03 | 92.37 | 80.80 |  |  |
| 5.26 | 89.94 | 91.67 |  |  | 6.04 | 92.35 | 80.77 |  |  |
| 5.27 | 89.92 | 91.64 |  |  | 6.05 | 92.31 | 80.74 |  |  |
| 5.28 | 89.89 | 91.62 |  |  | 6.06 | 92.31 | 80.72 |  |  |
| 5.29 | 89.87 | 91.59 |  |  | 6.07 | 92.28 | 80.69 |  |  |
| 5.30 | 89.85 | 91.56 |  |  | 6.08 | 92.26 | 80.66 |  |  |
| 5.31 | 89.83 | 91.53 |  |  | 6.09 | 92.25 | 80.64 |  |  |
| 5.32 | 89.80 | 91.51 |  |  | 6.10 | 92.23 | 80.61 |  |  |
| 5.33 | 89.78 | 91.48 |  |  | 6.11 | 92.21 | 80.58 |  |  |
| 5.34 | 89.73 | 91.45 |  |  | 6.13 | 92.16 | 80.55 |  |  |
| 5.35 | 89.70 | 91.42 |  |  | 6.15 | 92.14 | 80.53 |  |  |
| 5.36 | 89.68 | 91.40 |  |  | 6.16 | 92.10 | 80.50 |  |  |
| 5.37 | 89.66 | 91.37 |  |  | 6.17 | 92.09 | 80.47 |  |  |
| 5.38 | 89.64 | 91.34 |  |  | 6.19 | 92.05 | 80.45 |  |  |
| 5.40 | 89.61 | 91.28 |  |  | 6.20 | 92.03 | 80.42 |  |  |
| 5.41 | 89.59 | 91.26 |  |  | 6.21 | 92.01 | 80.39 |  |  |
| 5.42 | 89.58 | 91.23 |  |  | 6.22 | 92.00 | 80.36 |  |  |
| 5.43 | 89.56 | 91.20 |  |  | 6.23 | 91.99 | 80.34 |  |  |
| 5.44 | 89.53 | 91.17 |  |  | 6.24 | 91.96 | 80.28 |  |  |
| 5.45 | 89.51 | 91.15 |  |  | 6.25 | 91.94 | 80.26 |  |  |
| 5.47 | 89.46 | 91.09 |  |  | 6.26 | 91.92 | 80.23 |  |  |
| 5.49 | 89.42 | 91.03 |  |  | 6.27 | 91.89 | 80.20 |  |  |
| 5.51 | 89.36 | 90.98 |  |  | 6.28 | 91.87 | 80.18 |  |  |
| 5.52 | 89.33 | 90.95 |  |  | 6.29 | 91.86 | 80.15 |  |  |
| 5.53 | 89.32 | 90.92 |  |  | 6.30 | 91.84 | 80.09 |  |  |
| 5.54 | 89.31 | 90.90 |  |  | 6.31 | 91.82 | 80.07 |  |  |
| 5.55 | 89.28 | 90.87 |  |  | 6.32 | 91.80 | 80.04 |  |  |
| 5.56 | 89.26 | 90.84 |  |  | 6.33 | 91.79 | 80.01 |  |  |
| 5.57 | 89.25 | 90.81 |  |  | 6.34 | 91.77 | 79.99 |  |  |
| 5.58 | 89.22 | 90.78 |  |  | 6.35 | 91.75 | 79.96 |  |  |
| 5.59 | 89.21 | 90.76 |  |  | 6.36 | 91.73 | 79.93 |  |  |
| 5.60 | 89.18 | 90.73 |  |  | 6.37 | 91.70 | 79.90 |  |  |
| 5.61 | 89.16 | 90.70 |  |  | 6.38 | 91.68 | 79.88 |  |  |
| 5.62 | 89.16 | 90.67 |  |  | 6.39 | 91.67 | 79.85 |  |  |
| 5.63 | 89.14 | 90.65 |  |  | 6.40 | 91.65 | 79.82 |  |  |
| 5.64 | 89.12 | 90.62 |  |  | 6.41 | 91.63 | 79.80 |  |  |
| 5.65 | 89.10 | 90.59 |  |  | 6.43 | 91.60 | 79.77 |  |  |
| 5.67 | 89.07 | 90.53 |  |  | 6.44 | 91.57 | 79.72 |  |  |
| 5.68 | 89.04 | 90.51 |  |  | 6.45 | 91.56 | 79.69 |  |  |
| 5.69 | 89.02 | 90.48 |  |  | 6.46 | 91.54 | 79.66 |  |  |
| 5.70 | 89.00 | 90.45 |  |  | 6.47 | 91.52 | 79.63 |  |  |
| 5.71 | 88.99 | 90.42 |  |  | 6.48 | 91.49 | 79.61 |  |  |
| 5.72 | 88.97 | 90.40 |  |  | 6.49 | 91.49 | 79.55 |  |  |
| 5.73 | 88.96 | 90.37 |  |  | 6.50 | 91.47 | 79.53 |  |  |
| 5.75 | 88.91 | 90.31 |  |  | 6.51 | 91.44 | 79.50 |  |  |
| 5.76 | 88.89 | 90.28 |  |  | 6.52 | 91.44 | 79.47 |  |  |
| 5.77 | 88.88 | 90.26 |  |  | 6.53 | 91.42 | 79.44 |  |  |
| 5.78 | 88.87 | 90.23 |  |  | 6.54 | 91.40 | 79.42 |  |  |
| 5.79 | 88.86 | 90.20 |  |  | 6.55 | 91.38 | 79.39 |  |  |
| 5.80 | 88.83 | 90.17 |  |  | 6.56 | 91.36 | 79.36 |  |  |
| 5.81 | 88.81 | 90.15 |  |  | 6.57 | 91.34 | 79.34 |  |  |
| 5.82 | 88.78 | 90.12 |  |  | 6.58 | 91.32 | 79.31 |  |  |
| 5.83 | 88.77 | 90.09 |  |  | 6.60 | 91.29 | 79.28 |  |  |
| 5.84 | 88.74 | 90.06 |  |  | 6.61 | 91.27 | 79.26 |  |  |
| 5.85 | 88.71 | 90.03 |  |  | 6.62 | 91.26 | 79.23 |  |  |
| 5.87 | 88.69 | 89.98 |  |  | 6.63 | 91.25 | 79.20 |  |  |
| 5.88 | 88.67 | 89.95 |  |  | 6.64 | 91.23 | 79.17 |  |  |
| 5.89 | 88.64 | 89.92 |  |  | 6.65 | 91.22 | 79.15 |  |  |
| 5.90 | 88.61 | 89.90 |  |  | 6.67 | 91.19 | 79.12 |  |  |
| 5.91 | 88.58 | 89.87 |  |  | 6.68 | 91.18 | 79.09 |  |  |
| 5.92 | 88.57 | 89.84 |  |  | 6.69 | 91.16 | 79.07 |  |  |
| 5.93 | 88.54 | 89.81 |  |  | 6.70 | 91.15 | 79.04 |  |  |
| 5.94 | 88.52 | 89.79 |  |  | 6.71 | 91.13 | 79.01 |  |  |
| 5.95 | 88.50 | 89.76 |  |  | 6.73 | 91.11 | 78.98 |  |  |
| 5.96 | 88.49 | 89.73 |  |  | 6.75 | 91.08 | 78.96 |  |  |
| 5.97 | 88.47 | 89.70 |  |  | 6.76 | 91.06 | 78.93 |  |  |
| 5.98 | 88.45 | 89.67 |  |  | 6.77 | 91.05 | 78.90 |  |  |
| 5.99 | 88.41 | 89.65 |  |  | 6.78 | 91.04 | 78.88 |  |  |
| 6.01 | 88.37 | 89.59 |  |  | 6.79 | 91.03 | 78.85 |  |  |
| 6.02 | 88.34 | 89.56 |  |  | 6.80 | 91.02 | 78.82 |  |  |
| 6.03 | 88.31 | 89.54 |  |  | 6.81 | 91.01 | 78.80 |  |  |
| 6.04 | 88.30 | 89.51 |  |  | 6.82 | 91.00 | 78.77 |  |  |
| 6.05 | 88.27 | 89.48 |  |  | 6.83 | 90.97 | 78.74 |  |  |
| 6.06 | 88.25 | 89.45 |  |  | 6.84 | 90.96 | 78.69 |  |  |
| 6.07 | 88.24 | 89.42 |  |  | 6.85 | 90.95 | 78.66 |  |  |
| 6.08 | 88.22 | 89.40 |  |  | 6.86 | 90.94 | 78.63 |  |  |
| 6.09 | 88.20 | 89.37 |  |  | 6.87 | 90.91 | 78.61 |  |  |
| 6.10 | 88.18 | 89.34 |  |  | 6.88 | 90.90 | 78.55 |  |  |
| 6.12 | 88.12 | 89.29 |  |  | 6.89 | 90.88 | 78.52 |  |  |
| 6.14 | 88.09 | 89.23 |  |  | 6.90 | 90.87 | 78.50 |  |  |
| 6.15 | 88.07 | 89.20 |  |  | 6.91 | 90.86 | 78.47 |  |  |
| 6.16 | 88.05 | 89.17 |  |  | 6.92 | 90.85 | 78.44 |  |  |
| 6.17 | 88.02 | 89.15 |  |  | 6.93 | 90.83 | 78.42 |  |  |
| 6.18 | 88.00 | 89.12 |  |  | 6.94 | 90.82 | 78.39 |  |  |
| 6.19 | 87.98 | 89.09 |  |  | 6.95 | 90.79 | 78.36 |  |  |
| 6.20 | 87.95 | 89.06 |  |  | 6.96 | 90.77 | 78.34 |  |  |
| 6.21 | 87.93 | 89.04 |  |  | 6.97 | 90.77 | 78.31 |  |  |
| 6.22 | 87.90 | 89.01 |  |  | 6.99 | 90.74 | 78.28 |  |  |
| 6.23 | 87.88 | 88.98 |  |  | 7.00 | 90.73 | 78.25 |  |  |
| 6.24 | 87.85 | 88.95 |  |  | 7.01 | 90.72 | 78.23 |  |  |
| 6.25 | 87.83 | 88.92 |  |  | 7.02 | 90.70 | 78.20 |  |  |
| 6.26 | 87.81 | 88.90 |  |  | 7.03 | 90.67 | 78.17 |  |  |
| 6.27 | 87.78 | 88.87 |  |  | 7.04 | 90.65 | 78.15 |  |  |
| 6.28 | 87.77 | 88.84 |  |  | 7.05 | 90.64 | 78.12 |  |  |
| 6.29 | 87.74 | 88.81 |  |  | 7.06 | 90.62 | 78.09 |  |  |
| 6.30 | 87.72 | 88.79 |  |  | 7.07 | 90.61 | 78.06 |  |  |
| 6.31 | 87.70 | 88.76 |  |  | 7.08 | 90.60 | 78.04 |  |  |
| 6.32 | 87.68 | 88.73 |  |  | 7.09 | 90.57 | 78.01 |  |  |
| 6.33 | 87.64 | 88.70 |  |  | 7.10 | 90.56 | 77.98 |  |  |
| 6.34 | 87.62 | 88.67 |  |  | 7.11 | 90.54 | 77.96 |  |  |
| 6.35 | 87.59 | 88.65 |  |  | 7.12 | 90.52 | 77.93 |  |  |
| 6.36 | 87.56 | 88.62 |  |  | 7.13 | 90.51 | 77.90 |  |  |
| 6.37 | 87.53 | 88.59 |  |  | 7.14 | 90.48 | 77.88 |  |  |
| 6.39 | 87.47 | 88.54 |  |  | 7.15 | 90.48 | 77.85 |  |  |
| 6.40 | 87.46 | 88.51 |  |  | 7.16 | 90.46 | 77.82 |  |  |
| 6.41 | 87.43 | 88.48 |  |  | 7.18 | 90.44 | 77.79 |  |  |
| 6.42 | 87.41 | 88.45 |  |  | 7.19 | 90.41 | 77.77 |  |  |
| 6.44 | 87.35 | 88.40 |  |  | 7.20 | 90.40 | 77.74 |  |  |
| 6.45 | 87.31 | 88.37 |  |  | 7.21 | 90.38 | 77.71 |  |  |
| 6.46 | 87.29 | 88.34 |  |  | 7.22 | 90.36 | 77.69 |  |  |
| 6.47 | 87.26 | 88.31 |  |  | 7.23 | 90.36 | 77.66 |  |  |
| 6.48 | 87.24 | 88.29 |  |  | 7.24 | 90.34 | 77.63 |  |  |
| 6.49 | 87.19 | 88.26 |  |  | 7.25 | 90.33 | 77.60 |  |  |
| 6.50 | 87.16 | 88.23 |  |  | 7.26 | 90.31 | 77.58 |  |  |
| 6.52 | 87.11 | 88.17 |  |  | 7.27 | 90.29 | 77.55 |  |  |
| 6.53 | 87.08 | 88.15 |  |  | 7.28 | 90.27 | 77.52 |  |  |
| 6.55 | 87.04 | 88.09 |  |  | 7.29 | 90.25 | 77.50 |  |  |
| 6.56 | 87.02 | 88.06 |  |  | 7.30 | 90.23 | 77.47 |  |  |
| 6.57 | 86.99 | 88.04 |  |  | 7.31 | 90.21 | 77.44 |  |  |
| 6.58 | 86.96 | 88.01 |  |  | 7.32 | 90.19 | 77.42 |  |  |
| 6.59 | 86.94 | 87.98 |  |  | 7.33 | 90.18 | 77.39 |  |  |
| 6.60 | 86.92 | 87.95 |  |  | 7.34 | 90.17 | 77.36 |  |  |
| 6.61 | 86.87 | 87.93 |  |  | 7.35 | 90.14 | 77.33 |  |  |
| 6.62 | 86.86 | 87.90 |  |  | 7.36 | 90.11 | 77.31 |  |  |
| 6.63 | 86.84 | 87.87 |  |  | 7.37 | 90.10 | 77.28 |  |  |
| 6.64 | 86.81 | 87.84 |  |  | 7.38 | 90.08 | 77.25 |  |  |
| 6.65 | 86.79 | 87.81 |  |  | 7.39 | 90.07 | 77.23 |  |  |
| 6.66 | 86.78 | 87.79 |  |  | 7.40 | 90.04 | 77.20 |  |  |
| 6.67 | 86.75 | 87.76 |  |  | 7.41 | 90.03 | 77.17 |  |  |
| 6.68 | 86.72 | 87.73 |  |  | 7.42 | 90.01 | 77.14 |  |  |
| 6.69 | 86.67 | 87.70 |  |  | 7.43 | 90.00 | 77.12 |  |  |
| 6.70 | 86.64 | 87.68 |  |  | 7.44 | 89.98 | 77.09 |  |  |
| 6.71 | 86.63 | 87.65 |  |  | 7.45 | 89.97 | 77.06 |  |  |
| 6.72 | 86.60 | 87.62 |  |  | 7.46 | 89.96 | 77.04 |  |  |
| 6.73 | 86.56 | 87.59 |  |  | 7.47 | 89.93 | 77.01 |  |  |
| 6.74 | 86.55 | 87.56 |  |  | 7.48 | 89.91 | 76.98 |  |  |
| 6.75 | 86.52 | 87.54 |  |  | 7.49 | 89.90 | 76.96 |  |  |
| 6.76 | 86.50 | 87.51 |  |  | 7.50 | 89.87 | 76.90 |  |  |
| 6.77 | 86.49 | 87.48 |  |  | 7.51 | 89.85 | 76.87 |  |  |
| 6.78 | 86.47 | 87.45 |  |  | 7.52 | 89.83 | 76.85 |  |  |
| 6.79 | 86.43 | 87.43 |  |  | 7.53 | 89.81 | 76.82 |  |  |
| 6.80 | 86.42 | 87.40 |  |  | 7.54 | 89.79 | 76.79 |  |  |
| 6.81 | 86.40 | 87.37 |  |  | 7.55 | 89.78 | 76.77 |  |  |
| 6.82 | 86.38 | 87.34 |  |  | 7.56 | 89.76 | 76.71 |  |  |
| 6.83 | 86.36 | 87.31 |  |  | 7.58 | 89.72 | 76.68 |  |  |
| 6.84 | 86.34 | 87.29 |  |  | 7.59 | 89.69 | 76.66 |  |  |
| 6.85 | 86.32 | 87.26 |  |  | 7.60 | 89.68 | 76.63 |  |  |
| 6.86 | 86.30 | 87.23 |  |  | 7.61 | 89.67 | 76.60 |  |  |
| 6.87 | 86.28 | 87.20 |  |  | 7.62 | 89.64 | 76.58 |  |  |
| 6.88 | 86.26 | 87.18 |  |  | 7.63 | 89.62 | 76.55 |  |  |
| 6.89 | 86.25 | 87.15 |  |  | 7.64 | 89.60 | 76.52 |  |  |
| 6.90 | 86.24 | 87.12 |  |  | 7.65 | 89.59 | 76.50 |  |  |
| 6.91 | 86.22 | 87.09 |  |  | 7.66 | 89.56 | 76.47 |  |  |
| 6.92 | 86.21 | 87.06 |  |  | 7.67 | 89.55 | 76.44 |  |  |
| 6.94 | 86.16 | 87.01 |  |  | 7.69 | 89.51 | 76.41 |  |  |
| 6.96 | 86.12 | 86.95 |  |  | 7.70 | 89.48 | 76.39 |  |  |
| 6.98 | 86.09 | 86.90 |  |  | 7.71 | 89.45 | 76.36 |  |  |
| 6.99 | 86.08 | 86.87 |  |  | 7.73 | 89.42 | 76.33 |  |  |
| 7.00 | 86.04 | 86.84 |  |  | 7.74 | 89.40 | 76.28 |  |  |
| 7.01 | 86.03 | 86.81 |  |  | 7.75 | 89.38 | 76.25 |  |  |
| 7.02 | 86.01 | 86.79 |  |  | 7.76 | 89.37 | 76.23 |  |  |
| 7.03 | 85.98 | 86.76 |  |  | 7.77 | 89.33 | 76.20 |  |  |
| 7.04 | 85.97 | 86.73 |  |  | 7.78 | 89.31 | 76.17 |  |  |
| 7.05 | 85.95 | 86.70 |  |  | 7.79 | 89.30 | 76.14 |  |  |
| 7.06 | 85.92 | 86.68 |  |  | 7.80 | 89.28 | 76.12 |  |  |
| 7.07 | 85.89 | 86.65 |  |  | 7.81 | 89.27 | 76.09 |  |  |
| 7.08 | 85.87 | 86.62 |  |  | 7.83 | 89.25 | 76.06 |  |  |
| 7.09 | 85.85 | 86.59 |  |  | 7.84 | 89.22 | 76.04 |  |  |
| 7.10 | 85.83 | 86.56 |  |  | 7.85 | 89.20 | 75.98 |  |  |
| 7.11 | 85.80 | 86.54 |  |  | 7.86 | 89.18 | 75.93 |  |  |
| 7.12 | 85.77 | 86.51 |  |  | 7.87 | 89.17 | 75.87 |  |  |
| 7.13 | 85.74 | 86.48 |  |  | 7.88 | 89.14 | 75.82 |  |  |
| 7.14 | 85.72 | 86.45 |  |  | 7.89 | 89.11 | 75.79 |  |  |
| 7.15 | 85.70 | 86.43 |  |  | 7.90 | 89.09 | 75.77 |  |  |
| 7.16 | 85.67 | 86.40 |  |  | 7.91 | 89.06 | 75.74 |  |  |
| 7.17 | 85.63 | 86.37 |  |  | 7.92 | 89.05 | 75.71 |  |  |
| 7.18 | 85.61 | 86.34 |  |  | 7.93 | 89.04 | 75.68 |  |  |
| 7.19 | 85.57 | 86.32 |  |  | 7.94 | 89.02 | 75.66 |  |  |
| 7.20 | 85.54 | 86.29 |  |  | 7.95 | 89.01 | 75.63 |  |  |
| 7.21 | 85.52 | 86.26 |  |  | 7.96 | 89.00 | 75.58 |  |  |
| 7.22 | 85.50 | 86.23 |  |  | 7.97 | 88.98 | 75.55 |  |  |
| 7.24 | 85.44 | 86.18 |  |  | 7.98 | 88.95 | 75.49 |  |  |
| 7.25 | 85.40 | 86.15 |  |  | 7.99 | 88.95 | 75.47 |  |  |
| 7.26 | 85.36 | 86.12 |  |  | 8.01 | 88.90 | 75.44 |  |  |
| 7.27 | 85.33 | 86.09 |  |  | 8.02 | 88.89 | 75.41 |  |  |
| 7.28 | 85.31 | 86.07 |  |  | 8.03 | 88.87 | 75.39 |  |  |
| 7.29 | 85.26 | 86.04 |  |  | 8.04 | 88.86 | 75.36 |  |  |
| 7.30 | 85.25 | 86.01 |  |  | 8.05 | 88.84 | 75.33 |  |  |
| 7.31 | 85.22 | 85.98 |  |  | 8.06 | 88.82 | 75.31 |  |  |
| 7.32 | 85.18 | 85.95 |  |  | 8.07 | 88.80 | 75.28 |  |  |
| 7.33 | 85.15 | 85.93 |  |  | 8.08 | 88.79 | 75.25 |  |  |
| 7.34 | 85.12 | 85.90 |  |  | 8.09 | 88.77 | 75.22 |  |  |
| 7.35 | 85.09 | 85.87 |  |  | 8.10 | 88.75 | 75.17 |  |  |
| 7.37 | 85.00 | 85.82 |  |  | 8.11 | 88.73 | 75.14 |  |  |
| 7.39 | 84.95 | 85.76 |  |  | 8.12 | 88.71 | 75.12 |  |  |
| 7.40 | 84.92 | 85.73 |  |  | 8.13 | 88.69 | 75.09 |  |  |
| 7.41 | 84.89 | 85.70 |  |  | 8.14 | 88.67 | 75.06 |  |  |
| 7.42 | 84.86 | 85.68 |  |  | 8.15 | 88.65 | 75.03 |  |  |
| 7.43 | 84.83 | 85.65 |  |  | 8.16 | 88.63 | 75.01 |  |  |
| 7.44 | 84.80 | 85.62 |  |  | 8.17 | 88.61 | 74.95 |  |  |
| 7.45 | 84.76 | 85.59 |  |  | 8.19 | 88.59 | 74.90 |  |  |
| 7.46 | 84.73 | 85.57 |  |  | 8.20 | 88.58 | 74.87 |  |  |
| 7.47 | 84.71 | 85.54 |  |  | 8.21 | 88.55 | 74.85 |  |  |
| 7.49 | 84.65 | 85.48 |  |  | 8.23 | 88.53 | 74.82 |  |  |
| 7.50 | 84.61 | 85.45 |  |  | 8.24 | 88.52 | 74.79 |  |  |
| 7.52 | 84.56 | 85.40 |  |  | 8.27 | 88.46 | 74.71 |  |  |
| 7.53 | 84.53 | 85.37 |  |  | 8.28 | 88.45 | 74.68 |  |  |
| 7.54 | 84.49 | 85.34 |  |  | 8.29 | 88.43 | 74.66 |  |  |
| 7.55 | 84.45 | 85.32 |  |  | 8.30 | 88.43 | 74.63 |  |  |
| 7.56 | 84.41 | 85.29 |  |  | 8.31 | 88.41 | 74.60 |  |  |
| 7.58 | 84.35 | 85.23 |  |  | 8.32 | 88.39 | 74.57 |  |  |
| 7.59 | 84.32 | 85.20 |  |  | 8.33 | 88.38 | 74.52 |  |  |
| 7.60 | 84.29 | 85.18 |  |  | 8.34 | 88.38 | 74.49 |  |  |
| 7.62 | 84.21 | 85.12 |  |  | 8.35 | 88.35 | 74.47 |  |  |
| 7.63 | 84.18 | 85.09 |  |  | 8.36 | 88.33 | 74.44 |  |  |
| 7.64 | 84.12 | 85.07 |  |  | 8.37 | 88.31 | 74.41 |  |  |
| 7.65 | 84.08 | 85.04 |  |  | 8.38 | 88.29 | 74.39 |  |  |
| 7.66 | 84.05 | 85.01 |  |  | 8.39 | 88.27 | 74.36 |  |  |
| 7.67 | 84.00 | 84.98 |  |  | 8.40 | 88.26 | 74.33 |  |  |
| 7.68 | 83.98 | 84.95 |  |  | 8.41 | 88.24 | 74.30 |  |  |
| 7.69 | 83.96 | 84.93 |  |  | 8.42 | 88.23 | 74.28 |  |  |
| 7.71 | 83.87 | 84.87 |  |  | 8.45 | 88.17 | 74.25 |  |  |
| 7.72 | 83.84 | 84.84 |  |  | 8.46 | 88.16 | 74.20 |  |  |
| 7.73 | 83.80 | 84.82 |  |  | 8.47 | 88.13 | 74.17 |  |  |
| 7.74 | 83.77 | 84.79 |  |  | 8.48 | 88.11 | 74.14 |  |  |
| 7.75 | 83.72 | 84.76 |  |  | 8.49 | 88.09 | 74.11 |  |  |
| 7.76 | 83.68 | 84.73 |  |  | 8.50 | 88.08 | 74.09 |  |  |
| 7.77 | 83.65 | 84.70 |  |  | 8.51 | 88.05 | 74.06 |  |  |
| 7.78 | 83.60 | 84.68 |  |  | 8.52 | 88.05 | 74.01 |  |  |
| 7.79 | 83.55 | 84.65 |  |  | 8.53 | 88.03 | 73.98 |  |  |
| 7.80 | 83.50 | 84.62 |  |  | 8.54 | 88.01 | 73.95 |  |  |
| 7.81 | 83.46 | 84.59 |  |  | 8.55 | 87.99 | 73.93 |  |  |
| 7.82 | 83.41 | 84.57 |  |  | 8.56 | 87.98 | 73.90 |  |  |
| 7.83 | 83.39 | 84.54 |  |  | 8.57 | 87.97 | 73.84 |  |  |
| 7.84 | 83.36 | 84.51 |  |  | 8.58 | 87.95 | 73.82 |  |  |
| 7.85 | 83.32 | 84.48 |  |  | 8.59 | 87.93 | 73.79 |  |  |
| 7.86 | 83.28 | 84.46 |  |  | 8.60 | 87.91 | 73.76 |  |  |
| 7.87 | 83.25 | 84.43 |  |  | 8.61 | 87.89 | 73.74 |  |  |
| 7.88 | 83.21 | 84.40 |  |  | 8.62 | 87.88 | 73.71 |  |  |
| 7.89 | 83.18 | 84.37 |  |  | 8.63 | 87.86 | 73.68 |  |  |
| 7.90 | 83.14 | 84.34 |  |  | 8.64 | 87.85 | 73.65 |  |  |
| 7.91 | 83.11 | 84.32 |  |  | 8.65 | 87.83 | 73.63 |  |  |
| 7.92 | 83.09 | 84.29 |  |  | 8.66 | 87.81 | 73.60 |  |  |
| 7.93 | 83.04 | 84.26 |  |  | 8.67 | 87.79 | 73.57 |  |  |
| 7.94 | 83.01 | 84.23 |  |  | 8.68 | 87.76 | 73.55 |  |  |
| 7.95 | 82.97 | 84.21 |  |  | 8.69 | 87.74 | 73.49 |  |  |
| 7.97 | 82.92 | 84.15 |  |  | 8.70 | 87.74 | 73.47 |  |  |
| 8.00 | 82.81 | 84.07 |  |  | 8.71 | 87.71 | 73.44 |  |  |
| 8.01 | 82.76 | 84.04 |  |  | 8.72 | 87.69 | 73.41 |  |  |
| 8.02 | 82.73 | 84.01 |  |  | 8.73 | 87.67 | 73.36 |  |  |
| 8.03 | 82.70 | 83.98 |  |  | 8.74 | 87.66 | 73.33 |  |  |
| 8.04 | 82.67 | 83.96 |  |  | 8.75 | 87.63 | 73.30 |  |  |
| 8.05 | 82.63 | 83.93 |  |  | 8.77 | 87.60 | 73.28 |  |  |
| 8.06 | 82.60 | 83.90 |  |  | 8.78 | 87.57 | 73.25 |  |  |
| 8.08 | 82.54 | 83.84 |  |  | 8.79 | 87.55 | 73.22 |  |  |
| 8.09 | 82.49 | 83.82 |  |  | 8.80 | 87.54 | 73.19 |  |  |
| 8.10 | 82.45 | 83.79 |  |  | 8.81 | 87.51 | 73.14 |  |  |
| 8.11 | 82.42 | 83.76 |  |  | 8.82 | 87.49 | 73.11 |  |  |
| 8.12 | 82.37 | 83.73 |  |  | 8.83 | 87.46 | 73.06 |  |  |
| 8.13 | 82.35 | 83.71 |  |  | 8.85 | 87.41 | 73.03 |  |  |
| 8.14 | 82.32 | 83.68 |  |  | 8.86 | 87.39 | 73.01 |  |  |
| 8.15 | 82.29 | 83.65 |  |  | 8.87 | 87.37 | 72.98 |  |  |
| 8.16 | 82.26 | 83.62 |  |  | 8.88 | 87.36 | 72.95 |  |  |
| 8.18 | 82.21 | 83.57 |  |  | 8.89 | 87.34 | 72.92 |  |  |
| 8.19 | 82.18 | 83.54 |  |  | 8.90 | 87.33 | 72.90 |  |  |
| 8.20 | 82.15 | 83.51 |  |  | 8.91 | 87.31 | 72.87 |  |  |
| 8.21 | 82.11 | 83.48 |  |  | 8.92 | 87.31 | 72.84 |  |  |
| 8.22 | 82.08 | 83.46 |  |  | 8.93 | 87.30 | 72.82 |  |  |
| 8.23 | 82.04 | 83.43 |  |  | 8.94 | 87.29 | 72.79 |  |  |
| 8.24 | 82.01 | 83.40 |  |  | 8.95 | 87.28 | 72.76 |  |  |
| 8.25 | 81.99 | 83.37 |  |  | 8.97 | 87.24 | 72.73 |  |  |
| 8.26 | 81.96 | 83.34 |  |  | 8.98 | 87.23 | 72.71 |  |  |
| 8.27 | 81.92 | 83.32 |  |  | 8.99 | 87.20 | 72.65 |  |  |
| 8.28 | 81.88 | 83.29 |  |  | 9.00 | 87.19 | 72.63 |  |  |
| 8.29 | 81.85 | 83.26 |  |  | 9.01 | 87.18 | 72.60 |  |  |
| 8.31 | 81.79 | 83.21 |  |  | 9.02 | 87.17 | 72.57 |  |  |
| 8.32 | 81.76 | 83.18 |  |  | 9.03 | 87.16 | 72.55 |  |  |
| 8.33 | 81.73 | 83.15 |  |  | 9.04 | 87.14 | 72.52 |  |  |
| 8.34 | 81.69 | 83.12 |  |  | 9.05 | 87.13 | 72.49 |  |  |
| 8.35 | 81.66 | 83.09 |  |  | 9.06 | 87.11 | 72.46 |  |  |
| 8.36 | 81.64 | 83.07 |  |  | 9.08 | 87.06 | 72.44 |  |  |
| 8.37 | 81.60 | 83.04 |  |  | 9.09 | 87.03 | 72.41 |  |  |
| 8.38 | 81.57 | 83.01 |  |  | 9.10 | 87.01 | 72.38 |  |  |
| 8.39 | 81.53 | 82.98 |  |  | 9.11 | 86.99 | 72.36 |  |  |
| 8.40 | 81.50 | 82.96 |  |  | 9.12 | 86.98 | 72.33 |  |  |
| 8.41 | 81.46 | 82.93 |  |  | 9.13 | 86.96 | 72.30 |  |  |
| 8.42 | 81.43 | 82.90 |  |  | 9.14 | 86.95 | 72.27 |  |  |
| 8.43 | 81.39 | 82.87 |  |  | 9.15 | 86.93 | 72.25 |  |  |
| 8.44 | 81.34 | 82.84 |  |  | 9.16 | 86.92 | 72.22 |  |  |
| 8.45 | 81.31 | 82.82 |  |  | 9.18 | 86.90 | 72.17 |  |  |
| 8.46 | 81.26 | 82.79 |  |  | 9.19 | 86.89 | 72.14 |  |  |
| 8.47 | 81.24 | 82.76 |  |  | 9.20 | 86.87 | 72.11 |  |  |
| 8.48 | 81.21 | 82.73 |  |  | 9.21 | 86.86 | 72.06 |  |  |
| 8.49 | 81.19 | 82.71 |  |  | 9.22 | 86.85 | 72.03 |  |  |
| 8.50 | 81.15 | 82.68 |  |  | 9.23 | 86.83 | 72.00 |  |  |
| 8.51 | 81.14 | 82.65 |  |  | 9.24 | 86.82 | 71.98 |  |  |
| 8.52 | 81.11 | 82.62 |  |  | 9.25 | 86.81 | 71.95 |  |  |
| 8.53 | 81.05 | 82.60 |  |  | 9.26 | 86.80 | 71.92 |  |  |
| 8.54 | 81.03 | 82.57 |  |  | 9.27 | 86.78 | 71.90 |  |  |
| 8.55 | 81.00 | 82.54 |  |  | 9.28 | 86.76 | 71.87 |  |  |
| 8.56 | 80.97 | 82.51 |  |  | 9.29 | 86.73 | 71.84 |  |  |
| 8.57 | 80.93 | 82.48 |  |  | 9.30 | 86.71 | 71.81 |  |  |
| 8.58 | 80.88 | 82.46 |  |  | 9.31 | 86.70 | 71.79 |  |  |
| 8.59 | 80.86 | 82.43 |  |  | 9.32 | 86.68 | 71.73 |  |  |
| 8.60 | 80.82 | 82.40 |  |  | 9.33 | 86.65 | 71.68 |  |  |
| 8.61 | 80.80 | 82.37 |  |  | 9.34 | 86.64 | 71.65 |  |  |
| 8.62 | 80.76 | 82.35 |  |  | 9.35 | 86.61 | 71.63 |  |  |
| 8.63 | 80.72 | 82.32 |  |  | 9.36 | 86.60 | 71.57 |  |  |
| 8.64 | 80.69 | 82.29 |  |  | 9.37 | 86.59 | 71.54 |  |  |
| 8.65 | 80.65 | 82.26 |  |  | 9.38 | 86.56 | 71.52 |  |  |
| 8.66 | 80.62 | 82.23 |  |  | 9.39 | 86.55 | 71.49 |  |  |
| 8.67 | 80.59 | 82.21 |  |  | 9.41 | 86.52 | 71.46 |  |  |
| 8.68 | 80.56 | 82.18 |  |  | 9.42 | 86.51 | 71.44 |  |  |
| 8.69 | 80.53 | 82.15 |  |  | 9.43 | 86.50 | 71.41 |  |  |
| 8.70 | 80.50 | 82.12 |  |  | 9.45 | 86.47 | 71.38 |  |  |
| 8.71 | 80.47 | 82.10 |  |  | 9.46 | 86.45 | 71.35 |  |  |
| 8.72 | 80.45 | 82.07 |  |  | 9.47 | 86.44 | 71.33 |  |  |
| 8.73 | 80.41 | 82.04 |  |  | 9.48 | 86.42 | 71.27 |  |  |
| 8.74 | 80.40 | 82.01 |  |  | 9.49 | 86.40 | 71.25 |  |  |
| 8.75 | 80.38 | 81.98 |  |  | 9.52 | 86.38 | 71.22 |  |  |
| 8.76 | 80.35 | 81.96 |  |  | 9.53 | 86.36 | 71.19 |  |  |
| 8.78 | 80.31 | 81.90 |  |  | 9.54 | 86.35 | 71.17 |  |  |
| 8.79 | 80.27 | 81.87 |  |  | 9.55 | 86.32 | 71.14 |  |  |
| 8.81 | 80.23 | 81.82 |  |  | 9.56 | 86.32 | 71.08 |  |  |
| 8.82 | 80.20 | 81.79 |  |  | 9.57 | 86.30 | 71.06 |  |  |
| 8.83 | 80.17 | 81.76 |  |  | 9.58 | 86.29 | 71.03 |  |  |
| 8.84 | 80.15 | 81.73 |  |  | 9.59 | 86.26 | 71.00 |  |  |
| 8.85 | 80.13 | 81.71 |  |  | 9.60 | 86.25 | 70.98 |  |  |
| 8.86 | 80.10 | 81.68 |  |  | 9.61 | 86.23 | 70.95 |  |  |
| 8.87 | 80.08 | 81.65 |  |  | 9.62 | 86.22 | 70.89 |  |  |
| 8.88 | 80.05 | 81.62 |  |  | 9.63 | 86.20 | 70.87 |  |  |
| 8.89 | 80.02 | 81.60 |  |  | 9.64 | 86.18 | 70.84 |  |  |
| 8.90 | 80.01 | 81.57 |  |  | 9.65 | 86.15 | 70.81 |  |  |
| 8.91 | 79.97 | 81.54 |  |  | 9.66 | 86.13 | 70.79 |  |  |
| 8.92 | 79.94 | 81.51 |  |  | 9.67 | 86.12 | 70.76 |  |  |
| 8.93 | 79.90 | 81.48 |  |  | 9.68 | 86.10 | 70.73 |  |  |
| 8.94 | 79.86 | 81.46 |  |  | 9.69 | 86.09 | 70.71 |  |  |
| 8.95 | 79.83 | 81.43 |  |  | 9.70 | 86.07 | 70.68 |  |  |
| 8.96 | 79.80 | 81.40 |  |  | 9.71 | 86.06 | 70.65 |  |  |
| 8.97 | 79.77 | 81.37 |  |  | 9.72 | 86.02 | 70.62 |  |  |
| 8.98 | 79.76 | 81.35 |  |  | 9.73 | 86.01 | 70.57 |  |  |
| 9.00 | 79.69 | 81.29 |  |  | 9.74 | 85.99 | 70.54 |  |  |
| 9.01 | 79.65 | 81.26 |  |  | 9.75 | 85.97 | 70.52 |  |  |
| 9.02 | 79.61 | 81.23 |  |  | 9.76 | 85.95 | 70.46 |  |  |
| 9.03 | 79.58 | 81.21 |  |  | 9.77 | 85.92 | 70.43 |  |  |
| 9.04 | 79.54 | 81.18 |  |  | 9.78 | 85.88 | 70.41 |  |  |
| 9.05 | 79.51 | 81.15 |  |  | 9.79 | 85.86 | 70.38 |  |  |
| 9.06 | 79.49 | 81.12 |  |  | 9.80 | 85.84 | 70.35 |  |  |
| 9.07 | 79.47 | 81.10 |  |  | 9.81 | 85.82 | 70.33 |  |  |
| 9.08 | 79.43 | 81.07 |  |  | 9.82 | 85.80 | 70.30 |  |  |
| 9.09 | 79.39 | 81.04 |  |  | 9.83 | 85.79 | 70.27 |  |  |
| 9.10 | 79.37 | 81.01 |  |  | 9.84 | 85.75 | 70.25 |  |  |
| 9.11 | 79.35 | 80.99 |  |  | 9.85 | 85.73 | 70.22 |  |  |
| 9.12 | 79.33 | 80.96 |  |  | 9.86 | 85.70 | 70.19 |  |  |
| 9.13 | 79.28 | 80.93 |  |  | 9.87 | 85.69 | 70.16 |  |  |
| 9.14 | 79.25 | 80.90 |  |  | 9.88 | 85.66 | 70.14 |  |  |
| 9.15 | 79.22 | 80.87 |  |  | 9.91 | 85.60 | 70.08 |  |  |
| 9.16 | 79.18 | 80.85 |  |  | 9.92 | 85.59 | 70.06 |  |  |
| 9.17 | 79.16 | 80.82 |  |  | 9.94 | 85.53 | 70.03 |  |  |
| 9.18 | 79.12 | 80.79 |  |  | 9.95 | 85.52 | 70.00 |  |  |
| 9.19 | 79.10 | 80.76 |  |  | 9.96 | 85.50 | 69.97 |  |  |
| 9.20 | 79.06 | 80.74 |  |  | 9.97 | 85.48 | 69.95 |  |  |
| 9.21 | 79.04 | 80.71 |  |  | 9.98 | 85.46 | 69.92 |  |  |
| 9.22 | 79.01 | 80.68 |  |  | 9.99 | 85.45 | 69.89 |  |  |
| 9.24 | 78.96 | 80.62 |  |  | 10.00 | 85.41 | 69.87 |  |  |
| 9.25 | 78.93 | 80.60 |  |  | 10.01 | 85.38 | 69.84 |  |  |
| 9.26 | 78.90 | 80.57 |  |  | 10.02 | 85.37 | 69.79 |  |  |
| 9.28 | 78.86 | 80.51 |  |  | 10.03 | 85.35 | 69.76 |  |  |
| 9.30 | 78.81 | 80.46 |  |  | 10.05 | 85.31 | 69.73 |  |  |
| 9.31 | 78.77 | 80.43 |  |  | 10.07 | 85.28 | 69.70 |  |  |
| 9.32 | 78.74 | 80.40 |  |  | 10.08 | 85.25 | 69.68 |  |  |
| 9.33 | 78.72 | 80.37 |  |  | 10.09 | 85.23 | 69.65 |  |  |
| 9.34 | 78.70 | 80.35 |  |  | 10.10 | 85.21 | 69.62 |  |  |
| 9.35 | 78.67 | 80.32 |  |  | 10.11 | 85.18 | 69.60 |  |  |
| 9.36 | 78.64 | 80.29 |  |  | 10.12 | 85.17 | 69.57 |  |  |
| 9.37 | 78.62 | 80.26 |  |  | 10.13 | 85.15 | 69.54 |  |  |
| 9.38 | 78.59 | 80.24 |  |  | 10.14 | 85.13 | 69.51 |  |  |
| 9.39 | 78.57 | 80.21 |  |  | 10.15 | 85.10 | 69.49 |  |  |
| 9.40 | 78.55 | 80.18 |  |  | 10.16 | 85.08 | 69.46 |  |  |
| 9.41 | 78.53 | 80.15 |  |  | 10.17 | 85.06 | 69.43 |  |  |
| 9.42 | 78.50 | 80.12 |  |  | 10.18 | 85.04 | 69.41 |  |  |
| 9.43 | 78.49 | 80.10 |  |  | 10.19 | 85.02 | 69.38 |  |  |
| 9.44 | 78.46 | 80.07 |  |  | 10.20 | 85.01 | 69.33 |  |  |
| 9.45 | 78.45 | 80.04 |  |  | 10.21 | 84.98 | 69.30 |  |  |
| 9.46 | 78.41 | 80.01 |  |  | 10.23 | 84.95 | 69.27 |  |  |
| 9.48 | 78.38 | 79.96 |  |  | 10.24 | 84.94 | 69.24 |  |  |
| 9.49 | 78.36 | 79.93 |  |  | 10.25 | 84.93 | 69.22 |  |  |
| 9.52 | 78.33 | 79.85 |  |  | 10.26 | 84.91 | 69.19 |  |  |
| 9.53 | 78.32 | 79.82 |  |  | 10.27 | 84.89 | 69.16 |  |  |
| 9.54 | 78.29 | 79.79 |  |  | 10.28 | 84.86 | 69.14 |  |  |
| 9.55 | 78.27 | 79.76 |  |  | 10.29 | 84.84 | 69.11 |  |  |
| 9.56 | 78.25 | 79.74 |  |  | 10.30 | 84.82 | 69.08 |  |  |
| 9.57 | 78.23 | 79.71 |  |  | 10.31 | 84.80 | 69.05 |  |  |
| 9.58 | 78.20 | 79.68 |  |  | 10.33 | 84.77 | 69.03 |  |  |
| 9.59 | 78.18 | 79.65 |  |  | 10.34 | 84.75 | 69.00 |  |  |
| 9.60 | 78.16 | 79.62 |  |  | 10.36 | 84.72 | 68.97 |  |  |
| 9.61 | 78.14 | 79.60 |  |  | 10.37 | 84.70 | 68.95 |  |  |
| 9.62 | 78.13 | 79.57 |  |  | 10.38 | 84.68 | 68.92 |  |  |
| 9.63 | 78.12 | 79.54 |  |  | 10.39 | 84.67 | 68.89 |  |  |
| 9.64 | 78.12 | 79.51 |  |  | 10.40 | 84.64 | 68.87 |  |  |
| 9.65 | 78.09 | 79.49 |  |  | 10.41 | 84.62 | 68.84 |  |  |
| 9.66 | 78.07 | 79.46 |  |  | 10.42 | 84.59 | 68.78 |  |  |
| 9.67 | 78.06 | 79.43 |  |  | 10.43 | 84.58 | 68.76 |  |  |
| 9.69 | 78.02 | 79.37 |  |  | 10.44 | 84.57 | 68.73 |  |  |
| 9.70 | 78.01 | 79.35 |  |  | 10.45 | 84.55 | 68.70 |  |  |
| 9.71 | 78.00 | 79.32 |  |  | 10.46 | 84.53 | 68.68 |  |  |
| 9.72 | 77.96 | 79.29 |  |  | 10.48 | 84.49 | 68.65 |  |  |
| 9.73 | 77.94 | 79.26 |  |  | 10.49 | 84.48 | 68.62 |  |  |
| 9.75 | 77.89 | 79.21 |  |  | 10.50 | 84.47 | 68.59 |  |  |
| 9.76 | 77.88 | 79.18 |  |  | 10.51 | 84.45 | 68.57 |  |  |
| 9.77 | 77.85 | 79.15 |  |  | 10.53 | 84.42 | 68.54 |  |  |
| 9.79 | 77.81 | 79.10 |  |  | 10.54 | 84.41 | 68.51 |  |  |
| 9.80 | 77.80 | 79.07 |  |  | 10.55 | 84.40 | 68.49 |  |  |
| 9.81 | 77.77 | 79.04 |  |  | 10.56 | 84.39 | 68.46 |  |  |
| 9.82 | 77.75 | 79.01 |  |  | 10.57 | 84.37 | 68.43 |  |  |
| 9.83 | 77.74 | 78.99 |  |  | 10.58 | 84.35 | 68.41 |  |  |
| 9.84 | 77.72 | 78.96 |  |  | 10.59 | 84.33 | 68.38 |  |  |
| 9.85 | 77.70 | 78.93 |  |  | 10.60 | 84.32 | 68.30 |  |  |
| 9.86 | 77.68 | 78.90 |  |  | 10.62 | 84.28 | 68.27 |  |  |
| 9.87 | 77.67 | 78.88 |  |  | 10.63 | 84.26 | 68.19 |  |  |
| 9.88 | 77.65 | 78.85 |  |  | 10.64 | 84.23 | 68.16 |  |  |
| 9.89 | 77.63 | 78.82 |  |  | 10.65 | 84.21 | 68.13 |  |  |
| 9.90 | 77.61 | 78.79 |  |  | 10.66 | 84.19 | 68.11 |  |  |
| 9.91 | 77.58 | 78.76 |  |  | 10.67 | 84.18 | 68.08 |  |  |
| 9.92 | 77.57 | 78.74 |  |  | 10.69 | 84.16 | 68.05 |  |  |
| 9.93 | 77.55 | 78.71 |  |  | 10.70 | 84.14 | 68.03 |  |  |
| 9.94 | 77.52 | 78.68 |  |  | 10.71 | 84.12 | 68.00 |  |  |
| 9.95 | 77.50 | 78.65 |  |  | 10.73 | 84.08 | 67.97 |  |  |
| 9.96 | 77.48 | 78.63 |  |  | 10.74 | 84.07 | 67.95 |  |  |
| 9.97 | 77.47 | 78.60 |  |  | 10.75 | 84.05 | 67.92 |  |  |
| 9.98 | 77.44 | 78.57 |  |  | 10.76 | 84.03 | 67.89 |  |  |
| 10.00 | 77.39 | 78.51 |  |  | 10.77 | 84.01 | 67.86 |  |  |
| 10.01 | 77.37 | 78.49 |  |  | 10.78 | 83.99 | 67.84 |  |  |
| 10.02 | 77.34 | 78.46 |  |  | 10.79 | 83.97 | 67.81 |  |  |
| 10.03 | 77.32 | 78.43 |  |  | 10.80 | 83.96 | 67.78 |  |  |
| 10.04 | 77.29 | 78.40 |  |  | 10.81 | 83.94 | 67.76 |  |  |
| 10.05 | 77.28 | 78.38 |  |  | 10.82 | 83.93 | 67.73 |  |  |
| 10.06 | 77.27 | 78.35 |  |  | 10.83 | 83.91 | 67.70 |  |  |
| 10.07 | 77.24 | 78.32 |  |  | 10.84 | 83.89 | 67.67 |  |  |
| 10.09 | 77.20 | 78.26 |  |  | 10.85 | 83.88 | 67.65 |  |  |
| 10.10 | 77.17 | 78.24 |  |  | 10.86 | 83.86 | 67.59 |  |  |
| 10.11 | 77.14 | 78.21 |  |  | 10.87 | 83.84 | 67.57 |  |  |
| 10.12 | 77.12 | 78.18 |  |  | 10.88 | 83.82 | 67.54 |  |  |
| 10.13 | 77.09 | 78.15 |  |  | 10.89 | 83.81 | 67.51 |  |  |
| 10.14 | 77.06 | 78.13 |  |  | 10.90 | 83.79 | 67.49 |  |  |
| 10.15 | 77.03 | 78.10 |  |  | 10.91 | 83.77 | 67.46 |  |  |
| 10.16 | 77.01 | 78.07 |  |  | 10.92 | 83.76 | 67.40 |  |  |
| 10.17 | 76.98 | 78.04 |  |  | 10.93 | 83.73 | 67.38 |  |  |
| 10.18 | 76.95 | 78.01 |  |  | 10.94 | 83.71 | 67.32 |  |  |
| 10.19 | 76.93 | 77.99 |  |  | 10.96 | 83.68 | 67.30 |  |  |
| 10.20 | 76.88 | 77.96 |  |  | 10.97 | 83.67 | 67.27 |  |  |
| 10.21 | 76.85 | 77.93 |  |  | 10.98 | 83.65 | 67.24 |  |  |
| 10.22 | 76.81 | 77.90 |  |  | 10.99 | 83.64 | 67.21 |  |  |
| 10.23 | 76.78 | 77.88 |  |  | 11.00 | 83.62 | 67.19 |  |  |
| 10.24 | 76.76 | 77.85 |  |  | 11.02 | 83.60 | 67.16 |  |  |
| 10.25 | 76.73 | 77.82 |  |  | 11.03 | 83.57 | 67.13 |  |  |
| 10.26 | 76.71 | 77.79 |  |  | 11.04 | 83.55 | 67.11 |  |  |
| 10.27 | 76.69 | 77.76 |  |  | 11.05 | 83.53 | 67.08 |  |  |
| 10.28 | 76.66 | 77.74 |  |  | 11.06 | 83.52 | 67.05 |  |  |
| 10.29 | 76.63 | 77.71 |  |  | 11.07 | 83.50 | 67.03 |  |  |
| 10.30 | 76.61 | 77.68 |  |  | 11.08 | 83.49 | 67.00 |  |  |
| 10.31 | 76.59 | 77.65 |  |  | 11.09 | 83.46 | 66.97 |  |  |
| 10.32 | 76.57 | 77.63 |  |  | 11.10 | 83.45 | 66.92 |  |  |
| 10.33 | 76.55 | 77.60 |  |  | 11.11 | 83.44 | 66.89 |  |  |
| 10.34 | 76.53 | 77.57 |  |  | 11.12 | 83.42 | 66.84 |  |  |
| 10.35 | 76.51 | 77.54 |  |  | 11.13 | 83.40 | 66.81 |  |  |
| 10.36 | 76.48 | 77.52 |  |  | 11.14 | 83.38 | 66.78 |  |  |
| 10.37 | 76.45 | 77.49 |  |  | 11.15 | 83.36 | 66.75 |  |  |
| 10.38 | 76.42 | 77.46 |  |  | 11.16 | 83.35 | 66.70 |  |  |
| 10.39 | 76.41 | 77.43 |  |  | 11.17 | 83.33 | 66.67 |  |  |
| 10.40 | 76.37 | 77.40 |  |  | 11.18 | 83.32 | 66.65 |  |  |
| 10.41 | 76.35 | 77.38 |  |  | 11.19 | 83.30 | 66.62 |  |  |
| 10.42 | 76.33 | 77.35 |  |  | 11.21 | 83.24 | 66.59 |  |  |
| 10.43 | 76.31 | 77.32 |  |  | 11.23 | 83.20 | 66.57 |  |  |
| 10.44 | 76.29 | 77.29 |  |  | 11.24 | 83.17 | 66.54 |  |  |
| 10.45 | 76.28 | 77.27 |  |  | 11.26 | 83.12 | 66.51 |  |  |
| 10.46 | 76.26 | 77.24 |  |  | 11.27 | 83.08 | 66.48 |  |  |
| 10.47 | 76.22 | 77.21 |  |  | 11.28 | 83.06 | 66.46 |  |  |
| 10.48 | 76.21 | 77.18 |  |  | 11.29 | 83.04 | 66.43 |  |  |
| 10.49 | 76.19 | 77.15 |  |  | 11.30 | 83.02 | 66.40 |  |  |
| 10.51 | 76.14 | 77.10 |  |  | 11.31 | 83.00 | 66.38 |  |  |
| 10.53 | 76.10 | 77.04 |  |  | 11.32 | 82.97 | 66.35 |  |  |
| 10.54 | 76.08 | 77.02 |  |  | 11.33 | 82.95 | 66.32 |  |  |
| 10.56 | 76.02 | 76.96 |  |  | 11.34 | 82.93 | 66.29 |  |  |
| 10.57 | 76.01 | 76.93 |  |  | 11.35 | 82.91 | 66.27 |  |  |
| 10.58 | 76.00 | 76.90 |  |  | 11.36 | 82.88 | 66.24 |  |  |
| 10.59 | 75.98 | 76.88 |  |  | 11.37 | 82.86 | 66.21 |  |  |
| 10.60 | 75.96 | 76.85 |  |  | 11.38 | 82.84 | 66.19 |  |  |
| 10.61 | 75.94 | 76.82 |  |  | 11.39 | 82.81 | 66.16 |  |  |
| 10.62 | 75.92 | 76.79 |  |  | 11.41 | 82.75 | 66.13 |  |  |
| 10.63 | 75.90 | 76.77 |  |  | 11.42 | 82.73 | 66.11 |  |  |
| 10.64 | 75.88 | 76.74 |  |  | 11.43 | 82.70 | 66.08 |  |  |
| 10.65 | 75.85 | 76.71 |  |  | 11.45 | 82.66 | 66.05 |  |  |
| 10.66 | 75.84 | 76.68 |  |  | 11.46 | 82.64 | 66.02 |  |  |
| 10.67 | 75.83 | 76.65 |  |  | 11.47 | 82.61 | 66.00 |  |  |
| 10.68 | 75.82 | 76.63 |  |  | 11.48 | 82.60 | 65.97 |  |  |
| 10.69 | 75.80 | 76.60 |  |  | 11.49 | 82.57 | 65.94 |  |  |
| 10.70 | 75.79 | 76.57 |  |  | 11.50 | 82.55 | 65.89 |  |  |
| 10.71 | 75.77 | 76.54 |  |  | 11.51 | 82.52 | 65.86 |  |  |
| 10.72 | 75.76 | 76.52 |  |  | 11.52 | 82.49 | 65.83 |  |  |
| 10.74 | 75.73 | 76.46 |  |  | 11.53 | 82.47 | 65.78 |  |  |
| 10.75 | 75.72 | 76.43 |  |  | 11.54 | 82.45 | 65.75 |  |  |
| 10.76 | 75.69 | 76.40 |  |  | 11.55 | 82.44 | 65.73 |  |  |
| 10.77 | 75.67 | 76.38 |  |  | 11.56 | 82.41 | 65.70 |  |  |
| 10.79 | 75.65 | 76.32 |  |  | 11.57 | 82.39 | 65.67 |  |  |
| 10.80 | 75.62 | 76.29 |  |  | 11.58 | 82.37 | 65.65 |  |  |
| 10.81 | 75.61 | 76.27 |  |  | 11.60 | 82.34 | 65.62 |  |  |
| 10.82 | 75.61 | 76.24 |  |  | 11.62 | 82.29 | 65.59 |  |  |
| 10.83 | 75.59 | 76.21 |  |  | 11.65 | 82.20 | 65.56 |  |  |
| 10.84 | 75.57 | 76.18 |  |  | 11.66 | 82.19 | 65.54 |  |  |
| 10.85 | 75.56 | 76.15 |  |  | 11.67 | 82.17 | 65.51 |  |  |
| 10.86 | 75.56 | 76.13 |  |  | 11.68 | 82.12 | 65.48 |  |  |
| 10.87 | 75.54 | 76.10 |  |  | 11.69 | 82.09 | 65.43 |  |  |
| 10.88 | 75.52 | 76.07 |  |  | 11.70 | 82.08 | 65.40 |  |  |
| 10.89 | 75.50 | 76.04 |  |  | 11.72 | 82.04 | 65.37 |  |  |
| 10.90 | 75.49 | 76.02 |  |  | 11.73 | 82.02 | 65.35 |  |  |
| 10.91 | 75.47 | 75.99 |  |  | 11.74 | 82.00 | 65.32 |  |  |
| 10.93 | 75.45 | 75.93 |  |  | 11.75 | 81.98 | 65.27 |  |  |
| 10.95 | 75.42 | 75.88 |  |  | 11.76 | 81.95 | 65.24 |  |  |
| 10.96 | 75.40 | 75.85 |  |  | 11.77 | 81.92 | 65.21 |  |  |
| 10.99 | 75.36 | 75.77 |  |  | 11.78 | 81.89 | 65.19 |  |  |
| 11.00 | 75.35 | 75.74 |  |  | 11.81 | 81.80 | 65.16 |  |  |
| 11.01 | 75.33 | 75.71 |  |  | 11.82 | 81.78 | 65.13 |  |  |
| 11.02 | 75.31 | 75.68 |  |  | 11.83 | 81.73 | 65.10 |  |  |
| 11.03 | 75.30 | 75.66 |  |  | 11.84 | 81.71 | 65.08 |  |  |
| 11.04 | 75.29 | 75.63 |  |  | 11.85 | 81.68 | 65.05 |  |  |
| 11.05 | 75.28 | 75.60 |  |  | 11.86 | 81.65 | 65.02 |  |  |
| 11.07 | 75.26 | 75.54 |  |  | 11.87 | 81.62 | 65.00 |  |  |
| 11.08 | 75.24 | 75.52 |  |  | 11.88 | 81.59 | 64.97 |  |  |
| 11.09 | 75.22 | 75.49 |  |  | 11.89 | 81.57 | 64.94 |  |  |
| 11.10 | 75.20 | 75.46 |  |  | 11.90 | 81.54 | 64.91 |  |  |
| 11.11 | 75.18 | 75.43 |  |  | 11.91 | 81.51 | 64.86 |  |  |
| 11.13 | 75.14 | 75.38 |  |  | 11.92 | 81.50 | 64.83 |  |  |
| 11.14 | 75.12 | 75.35 |  |  | 11.93 | 81.47 | 64.81 |  |  |
| 11.15 | 75.10 | 75.32 |  |  | 11.94 | 81.45 | 64.78 |  |  |
| 11.16 | 75.09 | 75.29 |  |  | 11.96 | 81.38 | 64.75 |  |  |
| 11.17 | 75.06 | 75.27 |  |  | 11.97 | 81.35 | 64.73 |  |  |
| 11.18 | 75.04 | 75.24 |  |  | 11.98 | 81.32 | 64.67 |  |  |
| 11.20 | 75.01 | 75.18 |  |  | 12.00 | 81.25 | 64.64 |  |  |
| 11.22 | 74.98 | 75.13 |  |  | 12.02 | 81.19 | 64.62 |  |  |
| 11.23 | 74.96 | 75.10 |  |  | 12.04 | 81.15 | 64.59 |  |  |
| 11.24 | 74.94 | 75.07 |  |  | 12.05 | 81.11 | 64.56 |  |  |
| 11.25 | 74.92 | 75.04 |  |  | 12.06 | 81.10 | 64.54 |  |  |
| 11.26 | 74.91 | 75.02 |  |  | 12.07 | 81.08 | 64.51 |  |  |
| 11.27 | 74.88 | 74.99 |  |  | 12.08 | 81.05 | 64.48 |  |  |
| 11.28 | 74.86 | 74.96 |  |  | 12.09 | 81.01 | 64.46 |  |  |
| 11.29 | 74.85 | 74.93 |  |  | 12.10 | 80.98 | 64.43 |  |  |
| 11.31 | 74.81 | 74.88 |  |  | 12.11 | 80.95 | 64.40 |  |  |
| 11.32 | 74.79 | 74.85 |  |  | 12.12 | 80.93 | 64.35 |  |  |
| 11.33 | 74.77 | 74.82 |  |  | 12.14 | 80.89 | 64.32 |  |  |
| 11.34 | 74.76 | 74.79 |  |  | 12.15 | 80.86 | 64.27 |  |  |
| 11.35 | 74.74 | 74.77 |  |  | 12.16 | 80.83 | 64.24 |  |  |
| 11.36 | 74.72 | 74.74 |  |  | 12.17 | 80.81 | 64.21 |  |  |
| 11.38 | 74.68 | 74.68 |  |  | 12.18 | 80.78 | 64.18 |  |  |
| 11.39 | 74.66 | 74.66 |  |  | 12.19 | 80.76 | 64.16 |  |  |
| 11.40 | 74.65 | 74.63 |  |  | 12.20 | 80.73 | 64.13 |  |  |
| 11.41 | 74.63 | 74.60 |  |  | 12.21 | 80.72 | 64.10 |  |  |
| 11.42 | 74.61 | 74.57 |  |  | 12.22 | 80.69 | 64.08 |  |  |
| 11.43 | 74.60 | 74.54 |  |  | 12.23 | 80.68 | 64.05 |  |  |
| 11.45 | 74.55 | 74.49 |  |  | 12.24 | 80.65 | 64.02 |  |  |
| 11.46 | 74.53 | 74.46 |  |  | 12.25 | 80.64 | 64.00 |  |  |
| 11.47 | 74.51 | 74.43 |  |  | 12.26 | 80.61 | 63.97 |  |  |
| 11.48 | 74.48 | 74.41 |  |  | 12.27 | 80.60 | 63.94 |  |  |
| 11.49 | 74.45 | 74.38 |  |  | 12.28 | 80.58 | 63.91 |  |  |
| 11.50 | 74.43 | 74.35 |  |  | 12.29 | 80.56 | 63.89 |  |  |
| 11.51 | 74.40 | 74.32 |  |  | 12.30 | 80.53 | 63.86 |  |  |
| 11.52 | 74.39 | 74.29 |  |  | 12.31 | 80.52 | 63.83 |  |  |
| 11.53 | 74.37 | 74.27 |  |  | 12.32 | 80.50 | 63.81 |  |  |
| 11.54 | 74.35 | 74.24 |  |  | 12.33 | 80.48 | 63.78 |  |  |
| 11.55 | 74.32 | 74.21 |  |  | 12.34 | 80.46 | 63.75 |  |  |
| 11.56 | 74.31 | 74.18 |  |  | 12.35 | 80.44 | 63.72 |  |  |
| 11.57 | 74.28 | 74.16 |  |  | 12.36 | 80.41 | 63.70 |  |  |
| 11.59 | 74.23 | 74.10 |  |  | 12.37 | 80.38 | 63.67 |  |  |
| 11.60 | 74.21 | 74.07 |  |  | 12.39 | 80.33 | 63.64 |  |  |
| 11.62 | 74.17 | 74.02 |  |  | 12.40 | 80.31 | 63.62 |  |  |
| 11.63 | 74.15 | 73.99 |  |  | 12.41 | 80.29 | 63.59 |  |  |
| 11.64 | 74.14 | 73.96 |  |  | 12.42 | 80.26 | 63.56 |  |  |
| 11.65 | 74.12 | 73.93 |  |  | 12.43 | 80.25 | 63.54 |  |  |
| 11.66 | 74.11 | 73.91 |  |  | 12.44 | 80.22 | 63.51 |  |  |
| 11.67 | 74.10 | 73.88 |  |  | 12.45 | 80.21 | 63.48 |  |  |
| 11.68 | 74.08 | 73.85 |  |  | 12.46 | 80.18 | 63.45 |  |  |
| 11.69 | 74.05 | 73.82 |  |  | 12.47 | 80.15 | 63.43 |  |  |
| 11.70 | 74.03 | 73.80 |  |  | 12.48 | 80.12 | 63.40 |  |  |
| 11.71 | 74.02 | 73.77 |  |  | 12.49 | 80.10 | 63.37 |  |  |
| 11.72 | 74.00 | 73.74 |  |  | 12.50 | 80.08 | 63.32 |  |  |
| 11.73 | 73.98 | 73.71 |  |  | 12.51 | 80.06 | 63.29 |  |  |
| 11.74 | 73.97 | 73.68 |  |  | 12.52 | 80.04 | 63.26 |  |  |
| 11.75 | 73.94 | 73.66 |  |  | 12.54 | 80.00 | 63.21 |  |  |
| 11.76 | 73.91 | 73.63 |  |  | 12.55 | 79.97 | 63.18 |  |  |
| 11.77 | 73.90 | 73.60 |  |  | 12.56 | 79.94 | 63.16 |  |  |
| 11.78 | 73.89 | 73.57 |  |  | 12.57 | 79.90 | 63.13 |  |  |
| 11.79 | 73.87 | 73.55 |  |  | 12.58 | 79.87 | 63.10 |  |  |
| 11.81 | 73.85 | 73.49 |  |  | 12.59 | 79.83 | 63.08 |  |  |
| 11.82 | 73.84 | 73.46 |  |  | 12.60 | 79.80 | 62.99 |  |  |
| 11.84 | 73.80 | 73.41 |  |  | 12.61 | 79.77 | 62.97 |  |  |
| 11.85 | 73.78 | 73.38 |  |  | 12.62 | 79.72 | 62.94 |  |  |
| 11.86 | 73.77 | 73.35 |  |  | 12.63 | 79.69 | 62.91 |  |  |
| 11.87 | 73.75 | 73.32 |  |  | 12.65 | 79.63 | 62.89 |  |  |
| 11.88 | 73.74 | 73.30 |  |  | 12.66 | 79.61 | 62.86 |  |  |
| 11.89 | 73.72 | 73.27 |  |  | 12.67 | 79.58 | 62.78 |  |  |
| 11.90 | 73.71 | 73.24 |  |  | 12.68 | 79.55 | 62.75 |  |  |
| 11.91 | 73.70 | 73.21 |  |  | 12.69 | 79.50 | 62.72 |  |  |
| 11.92 | 73.68 | 73.18 |  |  | 12.70 | 79.46 | 62.70 |  |  |
| 11.93 | 73.67 | 73.16 |  |  | 12.72 | 79.40 | 62.67 |  |  |
| 11.95 | 73.64 | 73.10 |  |  | 12.73 | 79.37 | 62.64 |  |  |
| 11.96 | 73.62 | 73.07 |  |  | 12.74 | 79.34 | 62.59 |  |  |
| 11.97 | 73.60 | 73.05 |  |  | 12.75 | 79.32 | 62.56 |  |  |
| 11.98 | 73.59 | 73.02 |  |  | 12.76 | 79.27 | 62.53 |  |  |
| 11.99 | 73.58 | 72.99 |  |  | 12.78 | 79.21 | 62.51 |  |  |
| 12.00 | 73.57 | 72.96 |  |  | 12.79 | 79.18 | 62.48 |  |  |
| 12.01 | 73.56 | 72.93 |  |  | 12.80 | 79.15 | 62.45 |  |  |
| 12.02 | 73.54 | 72.91 |  |  | 12.82 | 79.11 | 62.43 |  |  |
| 12.03 | 73.52 | 72.88 |  |  | 12.84 | 79.04 | 62.40 |  |  |
| 12.05 | 73.50 | 72.82 |  |  | 12.85 | 79.02 | 62.37 |  |  |
| 12.06 | 73.49 | 72.80 |  |  | 12.87 | 78.94 | 62.34 |  |  |
| 12.07 | 73.47 | 72.77 |  |  | 12.88 | 78.91 | 62.32 |  |  |
| 12.08 | 73.46 | 72.74 |  |  | 12.89 | 78.88 | 62.29 |  |  |
| 12.09 | 73.45 | 72.71 |  |  | 12.90 | 78.85 | 62.26 |  |  |
| 12.10 | 73.43 | 72.68 |  |  | 12.91 | 78.81 | 62.24 |  |  |
| 12.11 | 73.43 | 72.66 |  |  | 12.93 | 78.77 | 62.21 |  |  |
| 12.12 | 73.41 | 72.63 |  |  | 12.94 | 78.74 | 62.18 |  |  |
| 12.13 | 73.39 | 72.60 |  |  | 12.95 | 78.70 | 62.16 |  |  |
| 12.15 | 73.37 | 72.55 |  |  | 12.96 | 78.66 | 62.13 |  |  |
| 12.16 | 73.36 | 72.52 |  |  | 12.97 | 78.61 | 62.10 |  |  |
| 12.17 | 73.35 | 72.49 |  |  | 12.98 | 78.60 | 62.05 |  |  |
| 12.18 | 73.34 | 72.46 |  |  | 12.99 | 78.57 | 62.02 |  |  |
| 12.19 | 73.33 | 72.43 |  |  | 13.00 | 78.55 | 61.99 |  |  |
| 12.20 | 73.33 | 72.41 |  |  | 13.01 | 78.52 | 61.97 |  |  |
| 12.22 | 73.31 | 72.35 |  |  | 13.02 | 78.49 | 61.94 |  |  |
| 12.23 | 73.30 | 72.32 |  |  | 13.03 | 78.47 | 61.91 |  |  |
| 12.24 | 73.29 | 72.30 |  |  | 13.04 | 78.44 | 61.88 |  |  |
| 12.25 | 73.27 | 72.27 |  |  | 13.05 | 78.38 | 61.86 |  |  |
| 12.26 | 73.27 | 72.24 |  |  | 13.06 | 78.35 | 61.83 |  |  |
| 12.27 | 73.26 | 72.21 |  |  | 13.07 | 78.30 | 61.80 |  |  |
| 12.28 | 73.25 | 72.19 |  |  | 13.08 | 78.26 | 61.78 |  |  |
| 12.29 | 73.23 | 72.16 |  |  | 13.09 | 78.23 | 61.75 |  |  |
| 12.30 | 73.21 | 72.13 |  |  | 13.10 | 78.20 | 61.72 |  |  |
| 12.31 | 73.19 | 72.10 |  |  | 13.11 | 78.19 | 61.67 |  |  |
| 12.32 | 73.18 | 72.07 |  |  | 13.12 | 78.15 | 61.64 |  |  |
| 12.33 | 73.16 | 72.05 |  |  | 13.13 | 78.13 | 61.61 |  |  |
| 12.34 | 73.15 | 72.02 |  |  | 13.14 | 78.09 | 61.59 |  |  |
| 12.35 | 73.12 | 71.99 |  |  | 13.15 | 78.07 | 61.56 |  |  |
| 12.36 | 73.11 | 71.96 |  |  | 13.16 | 78.05 | 61.53 |  |  |
| 12.37 | 73.10 | 71.94 |  |  | 13.17 | 78.02 | 61.51 |  |  |
| 12.38 | 73.09 | 71.91 |  |  | 13.18 | 77.99 | 61.48 |  |  |
| 12.39 | 73.08 | 71.88 |  |  | 13.19 | 77.95 | 61.45 |  |  |
| 12.40 | 73.06 | 71.85 |  |  | 13.22 | 77.87 | 61.42 |  |  |
| 12.41 | 73.05 | 71.82 |  |  | 13.23 | 77.84 | 61.40 |  |  |
| 12.42 | 73.03 | 71.80 |  |  | 13.24 | 77.81 | 61.37 |  |  |
| 12.44 | 72.98 | 71.74 |  |  | 13.25 | 77.77 | 61.34 |  |  |
| 12.45 | 72.95 | 71.71 |  |  | 13.26 | 77.74 | 61.32 |  |  |
| 12.46 | 72.93 | 71.69 |  |  | 13.27 | 77.71 | 61.29 |  |  |
| 12.47 | 72.90 | 71.66 |  |  | 13.28 | 77.69 | 61.26 |  |  |
| 12.49 | 72.85 | 71.60 |  |  | 13.29 | 77.66 | 61.24 |  |  |
| 12.50 | 72.84 | 71.57 |  |  | 13.30 | 77.63 | 61.18 |  |  |
| 12.51 | 72.82 | 71.55 |  |  | 13.31 | 77.62 | 61.15 |  |  |
| 12.52 | 72.81 | 71.52 |  |  | 13.32 | 77.59 | 61.13 |  |  |
| 12.53 | 72.79 | 71.49 |  |  | 13.33 | 77.55 | 61.10 |  |  |
| 12.54 | 72.78 | 71.46 |  |  | 13.34 | 77.53 | 61.07 |  |  |
| 12.56 | 72.74 | 71.41 |  |  | 13.35 | 77.51 | 61.05 |  |  |
| 12.57 | 72.71 | 71.38 |  |  | 13.36 | 77.48 | 61.02 |  |  |
| 12.58 | 72.69 | 71.35 |  |  | 13.37 | 77.45 | 60.99 |  |  |
| 12.59 | 72.67 | 71.32 |  |  | 13.38 | 77.41 | 60.96 |  |  |
| 12.60 | 72.66 | 71.30 |  |  | 13.39 | 77.39 | 60.94 |  |  |
| 12.61 | 72.64 | 71.27 |  |  | 13.41 | 77.33 | 60.91 |  |  |
| 12.62 | 72.61 | 71.24 |  |  | 13.43 | 77.25 | 60.88 |  |  |
| 12.63 | 72.60 | 71.21 |  |  | 13.44 | 77.21 | 60.86 |  |  |
| 12.64 | 72.59 | 71.19 |  |  | 13.45 | 77.17 | 60.83 |  |  |
| 12.65 | 72.58 | 71.16 |  |  | 13.46 | 77.14 | 60.80 |  |  |
| 12.66 | 72.56 | 71.13 |  |  | 13.47 | 77.12 | 60.78 |  |  |
| 12.67 | 72.54 | 71.10 |  |  | 13.48 | 77.08 | 60.75 |  |  |
| 12.68 | 72.51 | 71.07 |  |  | 13.49 | 77.05 | 60.72 |  |  |
| 12.69 | 72.49 | 71.05 |  |  | 13.50 | 77.00 | 60.69 |  |  |
| 12.70 | 72.47 | 71.02 |  |  | 13.51 | 76.96 | 60.67 |  |  |
| 12.71 | 72.46 | 70.99 |  |  | 13.52 | 76.90 | 60.64 |  |  |
| 12.72 | 72.45 | 70.96 |  |  | 13.53 | 76.87 | 60.61 |  |  |
| 12.73 | 72.43 | 70.94 |  |  | 13.54 | 76.83 | 60.59 |  |  |
| 12.74 | 72.42 | 70.91 |  |  | 13.55 | 76.79 | 60.53 |  |  |
| 12.75 | 72.39 | 70.88 |  |  | 13.56 | 76.73 | 60.50 |  |  |
| 12.76 | 72.37 | 70.85 |  |  | 13.57 | 76.70 | 60.45 |  |  |
| 12.77 | 72.36 | 70.82 |  |  | 13.58 | 76.67 | 60.42 |  |  |
| 12.78 | 72.34 | 70.80 |  |  | 13.59 | 76.64 | 60.40 |  |  |
| 12.79 | 72.33 | 70.77 |  |  | 13.60 | 76.59 | 60.37 |  |  |
| 12.80 | 72.32 | 70.74 |  |  | 13.61 | 76.55 | 60.34 |  |  |
| 12.81 | 72.30 | 70.71 |  |  | 13.62 | 76.53 | 60.32 |  |  |
| 12.82 | 72.29 | 70.69 |  |  | 13.63 | 76.49 | 60.29 |  |  |
| 12.83 | 72.27 | 70.66 |  |  | 13.64 | 76.44 | 60.26 |  |  |
| 12.84 | 72.25 | 70.63 |  |  | 13.65 | 76.40 | 60.23 |  |  |
| 12.85 | 72.23 | 70.60 |  |  | 13.66 | 76.35 | 60.21 |  |  |
| 12.86 | 72.22 | 70.58 |  |  | 13.67 | 76.32 | 60.18 |  |  |
| 12.87 | 72.21 | 70.55 |  |  | 13.68 | 76.28 | 60.15 |  |  |
| 12.88 | 72.18 | 70.52 |  |  | 13.69 | 76.24 | 60.10 |  |  |
| 12.89 | 72.16 | 70.49 |  |  | 13.70 | 76.19 | 60.07 |  |  |
| 12.90 | 72.15 | 70.46 |  |  | 13.71 | 76.15 | 60.04 |  |  |
| 12.91 | 72.13 | 70.44 |  |  | 13.72 | 76.12 | 60.02 |  |  |
| 12.92 | 72.10 | 70.41 |  |  | 13.73 | 76.08 | 59.99 |  |  |
| 12.95 | 72.05 | 70.33 |  |  | 13.74 | 76.02 | 59.96 |  |  |
| 12.96 | 72.04 | 70.30 |  |  | 13.75 | 75.96 | 59.94 |  |  |
| 12.98 | 72.01 | 70.24 |  |  | 13.76 | 75.92 | 59.91 |  |  |
| 12.99 | 72.00 | 70.21 |  |  | 13.77 | 75.88 | 59.88 |  |  |
| 13.00 | 71.98 | 70.19 |  |  | 13.78 | 75.85 | 59.86 |  |  |
| 13.01 | 71.97 | 70.16 |  |  | 13.80 | 75.76 | 59.83 |  |  |
| 13.02 | 71.95 | 70.13 |  |  | 13.81 | 75.72 | 59.80 |  |  |
| 13.03 | 71.92 | 70.10 |  |  | 13.82 | 75.68 | 59.77 |  |  |
| 13.04 | 71.90 | 70.08 |  |  | 13.83 | 75.63 | 59.75 |  |  |
| 13.05 | 71.87 | 70.05 |  |  | 13.84 | 75.60 | 59.72 |  |  |
| 13.06 | 71.86 | 70.02 |  |  | 13.85 | 75.57 | 59.69 |  |  |
| 13.07 | 71.84 | 69.99 |  |  | 13.86 | 75.54 | 59.67 |  |  |
| 13.08 | 71.84 | 69.96 |  |  | 13.87 | 75.48 | 59.64 |  |  |
| 13.09 | 71.82 | 69.94 |  |  | 13.88 | 75.45 | 59.61 |  |  |
| 13.10 | 71.82 | 69.91 |  |  | 13.89 | 75.41 | 59.58 |  |  |
| 13.11 | 71.80 | 69.88 |  |  | 13.90 | 75.36 | 59.56 |  |  |
| 13.12 | 71.78 | 69.85 |  |  | 13.91 | 75.32 | 59.53 |  |  |
| 13.13 | 71.74 | 69.83 |  |  | 13.92 | 75.28 | 59.50 |  |  |
| 13.14 | 71.73 | 69.80 |  |  | 13.93 | 75.23 | 59.48 |  |  |
| 13.15 | 71.72 | 69.77 |  |  | 13.94 | 75.20 | 59.45 |  |  |
| 13.16 | 71.69 | 69.74 |  |  | 13.95 | 75.15 | 59.42 |  |  |
| 13.17 | 71.67 | 69.71 |  |  | 13.96 | 75.11 | 59.40 |  |  |
| 13.18 | 71.65 | 69.69 |  |  | 13.97 | 75.06 | 59.37 |  |  |
| 13.19 | 71.63 | 69.66 |  |  | 13.98 | 75.03 | 59.34 |  |  |
| 13.20 | 71.62 | 69.63 |  |  | 13.99 | 74.99 | 59.29 |  |  |
| 13.21 | 71.61 | 69.60 |  |  | 14.00 | 74.94 | 59.21 |  |  |
| 13.22 | 71.58 | 69.58 |  |  | 14.01 | 74.89 | 59.18 |  |  |
| 13.23 | 71.58 | 69.55 |  |  | 14.02 | 74.84 | 59.15 |  |  |
| 13.24 | 71.56 | 69.52 |  |  | 14.03 | 74.79 | 59.12 |  |  |
| 13.25 | 71.55 | 69.49 |  |  | 14.05 | 74.71 | 59.10 |  |  |
| 13.26 | 71.54 | 69.46 |  |  | 14.06 | 74.67 | 59.07 |  |  |
| 13.27 | 71.53 | 69.44 |  |  | 14.07 | 74.63 | 59.04 |  |  |
| 13.29 | 71.49 | 69.38 |  |  | 14.08 | 74.58 | 59.02 |  |  |
| 13.30 | 71.48 | 69.35 |  |  | 14.09 | 74.53 | 58.99 |  |  |
| 13.31 | 71.45 | 69.33 |  |  | 14.10 | 74.46 | 58.96 |  |  |
| 13.32 | 71.44 | 69.30 |  |  | 14.11 | 74.42 | 58.94 |  |  |
| 13.33 | 71.41 | 69.27 |  |  | 14.12 | 74.35 | 58.91 |  |  |
| 13.34 | 71.39 | 69.24 |  |  | 14.13 | 74.30 | 58.88 |  |  |
| 13.36 | 71.38 | 69.19 |  |  | 14.14 | 74.27 | 58.85 |  |  |
| 13.37 | 71.36 | 69.16 |  |  | 14.16 | 74.21 | 58.83 |  |  |
| 13.38 | 71.33 | 69.13 |  |  | 14.17 | 74.17 | 58.80 |  |  |
| 13.39 | 71.32 | 69.10 |  |  | 14.18 | 74.13 | 58.77 |  |  |
| 13.40 | 71.30 | 69.08 |  |  | 14.19 | 74.07 | 58.75 |  |  |
| 13.41 | 71.29 | 69.05 |  |  | 14.20 | 74.02 | 58.72 |  |  |
| 13.42 | 71.27 | 69.02 |  |  | 14.21 | 73.95 | 58.69 |  |  |
| 13.43 | 71.25 | 68.99 |  |  | 14.22 | 73.93 | 58.66 |  |  |
| 13.44 | 71.23 | 68.96 |  |  | 14.23 | 73.91 | 58.64 |  |  |
| 13.47 | 71.19 | 68.88 |  |  | 14.24 | 73.87 | 58.61 |  |  |
| 13.48 | 71.17 | 68.85 |  |  | 14.25 | 73.85 | 58.58 |  |  |
| 13.49 | 71.15 | 68.83 |  |  | 14.26 | 73.79 | 58.53 |  |  |
| 13.50 | 71.14 | 68.80 |  |  | 14.27 | 73.73 | 58.50 |  |  |
| 13.51 | 71.12 | 68.77 |  |  | 14.28 | 73.69 | 58.48 |  |  |
| 13.52 | 71.09 | 68.74 |  |  | 14.29 | 73.66 | 58.45 |  |  |
| 13.54 | 71.06 | 68.69 |  |  | 14.30 | 73.62 | 58.42 |  |  |
| 13.55 | 71.04 | 68.66 |  |  | 14.31 | 73.58 | 58.39 |  |  |
| 13.56 | 71.03 | 68.63 |  |  | 14.32 | 73.52 | 58.37 |  |  |
| 13.57 | 71.01 | 68.60 |  |  | 14.33 | 73.49 | 58.31 |  |  |
| 13.58 | 70.99 | 68.58 |  |  | 14.34 | 73.45 | 58.29 |  |  |
| 13.59 | 70.97 | 68.55 |  |  | 14.35 | 73.40 | 58.23 |  |  |
| 13.60 | 70.95 | 68.52 |  |  | 14.36 | 73.36 | 58.20 |  |  |
| 13.61 | 70.93 | 68.49 |  |  | 14.37 | 73.30 | 58.18 |  |  |
| 13.62 | 70.92 | 68.47 |  |  | 14.38 | 73.26 | 58.15 |  |  |
| 13.63 | 70.89 | 68.44 |  |  | 14.39 | 73.22 | 58.10 |  |  |
| 13.64 | 70.87 | 68.41 |  |  | 14.40 | 73.17 | 58.07 |  |  |
| 13.65 | 70.85 | 68.38 |  |  | 14.42 | 73.07 | 58.02 |  |  |
| 13.66 | 70.84 | 68.35 |  |  | 14.43 | 73.02 | 57.99 |  |  |
| 13.68 | 70.79 | 68.30 |  |  | 14.44 | 72.96 | 57.96 |  |  |
| 13.69 | 70.78 | 68.27 |  |  | 14.45 | 72.91 | 57.93 |  |  |
| 13.70 | 70.76 | 68.24 |  |  | 14.46 | 72.85 | 57.91 |  |  |
| 13.71 | 70.74 | 68.22 |  |  | 14.47 | 72.80 | 57.88 |  |  |
| 13.72 | 70.73 | 68.19 |  |  | 14.48 | 72.76 | 57.85 |  |  |
| 13.73 | 70.71 | 68.16 |  |  | 14.49 | 72.73 | 57.83 |  |  |
| 13.75 | 70.68 | 68.10 |  |  | 14.50 | 72.68 | 57.80 |  |  |
| 13.76 | 70.66 | 68.08 |  |  | 14.51 | 72.64 | 57.77 |  |  |
| 13.77 | 70.64 | 68.05 |  |  | 14.52 | 72.61 | 57.74 |  |  |
| 13.78 | 70.63 | 68.02 |  |  | 14.53 | 72.57 | 57.72 |  |  |
| 13.79 | 70.61 | 67.99 |  |  | 14.55 | 72.46 | 57.64 |  |  |
| 13.80 | 70.60 | 67.97 |  |  | 14.56 | 72.42 | 57.61 |  |  |
| 13.81 | 70.57 | 67.94 |  |  | 14.57 | 72.39 | 57.58 |  |  |
| 13.82 | 70.56 | 67.91 |  |  | 14.58 | 72.34 | 57.56 |  |  |
| 13.84 | 70.52 | 67.85 |  |  | 14.60 | 72.25 | 57.53 |  |  |
| 13.85 | 70.51 | 67.83 |  |  | 14.62 | 72.18 | 57.50 |  |  |
| 13.86 | 70.49 | 67.80 |  |  | 14.63 | 72.14 | 57.47 |  |  |
| 13.88 | 70.45 | 67.74 |  |  | 14.64 | 72.09 | 57.45 |  |  |
| 13.89 | 70.43 | 67.72 |  |  | 14.65 | 72.06 | 57.42 |  |  |
| 13.91 | 70.39 | 67.66 |  |  | 14.66 | 72.01 | 57.39 |  |  |
| 13.92 | 70.36 | 67.63 |  |  | 14.67 | 71.96 | 57.37 |  |  |
| 13.93 | 70.34 | 67.60 |  |  | 14.68 | 71.92 | 57.34 |  |  |
| 13.94 | 70.32 | 67.58 |  |  | 14.69 | 71.87 | 57.31 |  |  |
| 13.95 | 70.30 | 67.55 |  |  | 14.71 | 71.79 | 57.28 |  |  |
| 13.96 | 70.29 | 67.52 |  |  | 14.72 | 71.75 | 57.23 |  |  |
| 13.97 | 70.27 | 67.49 |  |  | 14.73 | 71.71 | 57.20 |  |  |
| 13.98 | 70.26 | 67.47 |  |  | 14.74 | 71.63 | 57.18 |  |  |
| 13.99 | 70.25 | 67.44 |  |  | 14.75 | 71.60 | 57.15 |  |  |
| 14.00 | 70.22 | 67.41 |  |  | 14.76 | 71.54 | 57.12 |  |  |
| 14.01 | 70.19 | 67.38 |  |  | 14.77 | 71.50 | 57.10 |  |  |
| 14.02 | 70.17 | 67.35 |  |  | 14.78 | 71.45 | 57.07 |  |  |
| 14.03 | 70.15 | 67.33 |  |  | 14.79 | 71.41 | 57.04 |  |  |
| 14.04 | 70.15 | 67.30 |  |  | 14.80 | 71.36 | 57.01 |  |  |
| 14.05 | 70.12 | 67.27 |  |  | 14.81 | 71.31 | 56.99 |  |  |
| 14.07 | 70.09 | 67.22 |  |  | 14.82 | 71.28 | 56.96 |  |  |
| 14.08 | 70.08 | 67.19 |  |  | 14.83 | 71.22 | 56.93 |  |  |
| 14.10 | 70.03 | 67.13 |  |  | 14.84 | 71.17 | 56.91 |  |  |
| 14.11 | 70.02 | 67.11 |  |  | 14.85 | 71.12 | 56.88 |  |  |
| 14.12 | 69.99 | 67.08 |  |  | 14.87 | 71.02 | 56.82 |  |  |
| 14.14 | 69.96 | 67.02 |  |  | 14.88 | 70.98 | 56.80 |  |  |
| 14.15 | 69.94 | 66.99 |  |  | 14.89 | 70.91 | 56.77 |  |  |
| 14.16 | 69.91 | 66.97 |  |  | 14.90 | 70.85 | 56.74 |  |  |
| 14.17 | 69.87 | 66.94 |  |  | 14.91 | 70.79 | 56.72 |  |  |
| 14.18 | 69.86 | 66.91 |  |  | 14.92 | 70.74 | 56.69 |  |  |
| 14.19 | 69.85 | 66.88 |  |  | 14.93 | 70.70 | 56.66 |  |  |
| 14.20 | 69.83 | 66.86 |  |  | 14.94 | 70.64 | 56.64 |  |  |
| 14.21 | 69.81 | 66.83 |  |  | 14.95 | 70.58 | 56.61 |  |  |
| 14.22 | 69.79 | 66.80 |  |  | 14.96 | 70.54 | 56.58 |  |  |
| 14.23 | 69.76 | 66.77 |  |  | 14.98 | 70.42 | 56.55 |  |  |
| 14.24 | 69.75 | 66.74 |  |  | 14.99 | 70.37 | 56.53 |  |  |
| 14.25 | 69.73 | 66.72 |  |  | 15.00 | 70.33 | 56.50 |  |  |
| 14.26 | 69.71 | 66.69 |  |  | 15.01 | 70.27 | 56.47 |  |  |
| 14.27 | 69.69 | 66.66 |  |  | 15.02 | 70.21 | 56.45 |  |  |
| 14.29 | 69.65 | 66.61 |  |  | 15.04 | 70.11 | 56.39 |  |  |
| 14.30 | 69.62 | 66.58 |  |  | 15.05 | 70.06 | 56.36 |  |  |
| 14.31 | 69.58 | 66.55 |  |  | 15.06 | 70.03 | 56.34 |  |  |
| 14.32 | 69.57 | 66.52 |  |  | 15.07 | 69.96 | 56.31 |  |  |
| 14.33 | 69.55 | 66.49 |  |  | 15.08 | 69.90 | 56.28 |  |  |
| 14.34 | 69.53 | 66.47 |  |  | 15.09 | 69.86 | 56.26 |  |  |
| 14.35 | 69.52 | 66.44 |  |  | 15.10 | 69.81 | 56.23 |  |  |
| 14.36 | 69.50 | 66.41 |  |  | 15.11 | 69.74 | 56.20 |  |  |
| 14.37 | 69.48 | 66.38 |  |  | 15.12 | 69.69 | 56.18 |  |  |
| 14.40 | 69.44 | 66.30 |  |  | 15.13 | 69.65 | 56.15 |  |  |
| 14.41 | 69.43 | 66.27 |  |  | 15.14 | 69.60 | 56.12 |  |  |
| 14.42 | 69.41 | 66.24 |  |  | 15.15 | 69.56 | 56.09 |  |  |
| 14.43 | 69.40 | 66.22 |  |  | 15.16 | 69.51 | 56.07 |  |  |
| 14.44 | 69.38 | 66.19 |  |  | 15.17 | 69.44 | 56.04 |  |  |
| 14.45 | 69.35 | 66.16 |  |  | 15.19 | 69.34 | 56.01 |  |  |
| 14.46 | 69.33 | 66.13 |  |  | 15.20 | 69.30 | 55.99 |  |  |
| 14.47 | 69.30 | 66.11 |  |  | 15.22 | 69.17 | 55.96 |  |  |
| 14.48 | 69.29 | 66.08 |  |  | 15.23 | 69.15 | 55.93 |  |  |
| 14.49 | 69.27 | 66.05 |  |  | 15.25 | 69.04 | 55.90 |  |  |
| 14.50 | 69.25 | 66.02 |  |  | 15.26 | 68.99 | 55.88 |  |  |
| 14.52 | 69.22 | 65.97 |  |  | 15.27 | 68.92 | 55.85 |  |  |
| 14.53 | 69.21 | 65.94 |  |  | 15.28 | 68.88 | 55.82 |  |  |
| 14.54 | 69.19 | 65.91 |  |  | 15.29 | 68.79 | 55.80 |  |  |
| 14.55 | 69.17 | 65.88 |  |  | 15.30 | 68.76 | 55.77 |  |  |
| 14.56 | 69.16 | 65.86 |  |  | 15.31 | 68.69 | 55.74 |  |  |
| 14.57 | 69.15 | 65.83 |  |  | 15.32 | 68.64 | 55.72 |  |  |
| 14.58 | 69.13 | 65.80 |  |  | 15.33 | 68.58 | 55.69 |  |  |
| 14.59 | 69.12 | 65.77 |  |  | 15.34 | 68.51 | 55.66 |  |  |
| 14.60 | 69.09 | 65.74 |  |  | 15.35 | 68.47 | 55.63 |  |  |
| 14.61 | 69.07 | 65.72 |  |  | 15.36 | 68.38 | 55.61 |  |  |
| 14.62 | 69.06 | 65.69 |  |  | 15.37 | 68.31 | 55.58 |  |  |
| 14.63 | 69.05 | 65.66 |  |  | 15.38 | 68.24 | 55.55 |  |  |
| 14.64 | 69.02 | 65.63 |  |  | 15.39 | 68.17 | 55.53 |  |  |
| 14.65 | 69.01 | 65.61 |  |  | 15.40 | 68.09 | 55.50 |  |  |
| 14.66 | 68.99 | 65.58 |  |  | 15.41 | 68.02 | 55.47 |  |  |
| 14.67 | 68.99 | 65.55 |  |  | 15.42 | 67.92 | 55.42 |  |  |
| 14.68 | 68.98 | 65.52 |  |  | 15.45 | 67.69 | 55.39 |  |  |
| 14.69 | 68.97 | 65.49 |  |  | 15.46 | 67.61 | 55.36 |  |  |
| 14.70 | 68.93 | 65.47 |  |  | 15.47 | 67.55 | 55.34 |  |  |
| 14.71 | 68.92 | 65.44 |  |  | 15.48 | 67.49 | 55.31 |  |  |
| 14.72 | 68.90 | 65.41 |  |  | 15.49 | 67.39 | 55.28 |  |  |
| 14.73 | 68.88 | 65.38 |  |  | 15.50 | 67.33 | 55.26 |  |  |
| 14.74 | 68.87 | 65.36 |  |  | 15.51 | 67.24 | 55.23 |  |  |
| 14.75 | 68.86 | 65.33 |  |  | 15.52 | 67.16 | 55.20 |  |  |
| 14.76 | 68.84 | 65.30 |  |  | 15.53 | 67.10 | 55.17 |  |  |
| 14.77 | 68.83 | 65.27 |  |  | 15.54 | 67.04 | 55.15 |  |  |
| 14.78 | 68.81 | 65.25 |  |  | 15.55 | 66.96 | 55.12 |  |  |
| 14.79 | 68.79 | 65.22 |  |  | 15.56 | 66.89 | 55.09 |  |  |
| 14.80 | 68.78 | 65.19 |  |  | 15.57 | 66.79 | 55.07 |  |  |
| 14.81 | 68.76 | 65.16 |  |  | 15.58 | 66.73 | 55.04 |  |  |
| 14.82 | 68.74 | 65.13 |  |  | 15.59 | 66.67 | 55.01 |  |  |
| 14.83 | 68.73 | 65.11 |  |  | 15.60 | 66.58 | 54.98 |  |  |
| 14.84 | 68.72 | 65.08 |  |  | 15.61 | 66.51 | 54.96 |  |  |
| 14.85 | 68.70 | 65.05 |  |  | 15.62 | 66.44 | 54.93 |  |  |
| 14.86 | 68.68 | 65.02 |  |  | 15.63 | 66.36 | 54.90 |  |  |
| 14.87 | 68.66 | 65.00 |  |  | 15.64 | 66.26 | 54.88 |  |  |
| 14.88 | 68.65 | 64.97 |  |  | 15.65 | 66.20 | 54.85 |  |  |
| 14.89 | 68.64 | 64.94 |  |  | 15.66 | 66.11 | 54.82 |  |  |
| 14.92 | 68.60 | 64.86 |  |  | 15.67 | 66.04 | 54.80 |  |  |
| 14.93 | 68.58 | 64.83 |  |  | 15.68 | 65.96 | 54.77 |  |  |
| 14.94 | 68.56 | 64.80 |  |  | 15.69 | 65.88 | 54.74 |  |  |
| 14.95 | 68.54 | 64.77 |  |  | 15.70 | 65.80 | 54.71 |  |  |
| 14.96 | 68.52 | 64.75 |  |  | 15.71 | 65.74 | 54.69 |  |  |
| 14.97 | 68.49 | 64.72 |  |  | 15.72 | 65.66 | 54.66 |  |  |
| 14.98 | 68.48 | 64.69 |  |  | 15.73 | 65.57 | 54.61 |  |  |
| 14.99 | 68.47 | 64.66 |  |  | 15.74 | 65.50 | 54.58 |  |  |
| 15.00 | 68.45 | 64.63 |  |  | 15.75 | 65.41 | 54.55 |  |  |
| 15.01 | 68.44 | 64.61 |  |  | 15.76 | 65.31 | 54.52 |  |  |
| 15.02 | 68.42 | 64.58 |  |  | 15.77 | 65.26 | 54.50 |  |  |
| 15.03 | 68.40 | 64.55 |  |  | 15.78 | 65.17 | 54.47 |  |  |
| 15.05 | 68.36 | 64.50 |  |  | 15.79 | 65.09 | 54.44 |  |  |
| 15.06 | 68.33 | 64.47 |  |  | 15.80 | 65.00 | 54.42 |  |  |
| 15.08 | 68.31 | 64.41 |  |  | 15.81 | 64.93 | 54.39 |  |  |
| 15.09 | 68.28 | 64.38 |  |  | 15.82 | 64.84 | 54.36 |  |  |
| 15.10 | 68.27 | 64.36 |  |  | 15.83 | 64.75 | 54.34 |  |  |
| 15.11 | 68.24 | 64.33 |  |  | 15.84 | 64.66 | 54.31 |  |  |
| 15.12 | 68.22 | 64.30 |  |  | 15.85 | 64.58 | 54.28 |  |  |
| 15.13 | 68.20 | 64.27 |  |  | 15.86 | 64.50 | 54.25 |  |  |
| 15.14 | 68.19 | 64.25 |  |  | 15.87 | 64.42 | 54.23 |  |  |
| 15.15 | 68.16 | 64.22 |  |  | 15.88 | 64.32 | 54.20 |  |  |
| 15.16 | 68.14 | 64.19 |  |  | 15.89 | 64.21 | 54.15 |  |  |
| 15.17 | 68.11 | 64.16 |  |  | 15.90 | 64.13 | 54.12 |  |  |
| 15.18 | 68.09 | 64.13 |  |  | 15.91 | 64.04 | 54.09 |  |  |
| 15.19 | 68.08 | 64.11 |  |  | 15.92 | 63.96 | 54.06 |  |  |
| 15.20 | 68.05 | 64.08 |  |  | 15.93 | 63.90 | 54.04 |  |  |
| 15.21 | 68.03 | 64.05 |  |  | 15.94 | 63.77 | 53.98 |  |  |
| 15.22 | 68.01 | 64.02 |  |  | 15.95 | 63.69 | 53.96 |  |  |
| 15.23 | 67.99 | 64.00 |  |  | 15.96 | 63.59 | 53.93 |  |  |
| 15.24 | 67.97 | 63.97 |  |  | 15.97 | 63.50 | 53.90 |  |  |
| 15.25 | 67.96 | 63.94 |  |  | 15.98 | 63.39 | 53.88 |  |  |
| 15.28 | 67.90 | 63.86 |  |  | 15.99 | 63.30 | 53.85 |  |  |
| 15.29 | 67.88 | 63.83 |  |  | 16.00 | 63.22 | 53.82 |  |  |
| 15.32 | 67.85 | 63.75 |  |  | 16.01 | 63.12 | 53.79 |  |  |
| 15.33 | 67.83 | 63.72 |  |  | 16.02 | 63.03 | 53.77 |  |  |
| 15.34 | 67.79 | 63.69 |  |  | 16.03 | 62.92 | 53.74 |  |  |
| 15.35 | 67.77 | 63.66 |  |  | 16.04 | 62.84 | 53.71 |  |  |
| 15.37 | 67.72 | 63.61 |  |  | 16.05 | 62.74 | 53.69 |  |  |
| 15.38 | 67.68 | 63.58 |  |  | 16.06 | 62.65 | 53.66 |  |  |
| 15.39 | 67.65 | 63.55 |  |  | 16.07 | 62.57 | 53.63 |  |  |
| 15.40 | 67.62 | 63.52 |  |  | 16.08 | 62.47 | 53.60 |  |  |
| 15.41 | 67.61 | 63.50 |  |  | 16.09 | 62.36 | 53.58 |  |  |
| 15.42 | 67.58 | 63.47 |  |  | 16.11 | 62.17 | 53.55 |  |  |
| 15.43 | 67.56 | 63.44 |  |  | 16.12 | 62.05 | 53.52 |  |  |
| 15.44 | 67.52 | 63.41 |  |  | 16.13 | 61.98 | 53.50 |  |  |
| 15.45 | 67.48 | 63.39 |  |  | 16.14 | 61.89 | 53.47 |  |  |
| 15.46 | 67.46 | 63.36 |  |  | 16.15 | 61.80 | 53.44 |  |  |
| 15.47 | 67.43 | 63.33 |  |  | 16.16 | 61.73 | 53.42 |  |  |
| 15.48 | 67.41 | 63.30 |  |  | 16.17 | 61.65 | 53.36 |  |  |
| 15.49 | 67.39 | 63.27 |  |  | 16.18 | 61.53 | 53.33 |  |  |
| 15.50 | 67.37 | 63.25 |  |  | 16.19 | 61.44 | 53.31 |  |  |
| 15.51 | 67.35 | 63.22 |  |  | 16.20 | 61.37 | 53.28 |  |  |
| 15.52 | 67.33 | 63.19 |  |  | 16.21 | 61.29 | 53.25 |  |  |
| 15.53 | 67.30 | 63.16 |  |  | 16.22 | 61.19 | 53.23 |  |  |
| 15.54 | 67.29 | 63.14 |  |  | 16.23 | 61.11 | 53.20 |  |  |
| 15.55 | 67.27 | 63.11 |  |  | 16.24 | 61.01 | 53.17 |  |  |
| 15.56 | 67.25 | 63.08 |  |  | 16.25 | 60.93 | 53.14 |  |  |
| 15.57 | 67.22 | 63.05 |  |  | 16.26 | 60.83 | 53.12 |  |  |
| 15.58 | 67.18 | 63.02 |  |  | 16.27 | 60.74 | 53.09 |  |  |
| 15.60 | 67.13 | 62.97 |  |  | 16.28 | 60.66 | 53.06 |  |  |
| 15.61 | 67.12 | 62.94 |  |  | 16.29 | 60.56 | 53.04 |  |  |
| 15.63 | 67.06 | 62.89 |  |  | 16.30 | 60.48 | 53.01 |  |  |
| 15.64 | 67.03 | 62.86 |  |  | 16.31 | 60.38 | 52.98 |  |  |
| 15.65 | 67.02 | 62.83 |  |  | 16.32 | 60.31 | 52.96 |  |  |
| 15.66 | 67.00 | 62.80 |  |  | 16.33 | 60.23 | 52.90 |  |  |
| 15.67 | 66.98 | 62.77 |  |  | 16.34 | 60.15 | 52.87 |  |  |
| 15.68 | 66.96 | 62.75 |  |  | 16.35 | 60.04 | 52.85 |  |  |
| 15.69 | 66.94 | 62.72 |  |  | 16.36 | 59.97 | 52.82 |  |  |
| 15.70 | 66.93 | 62.69 |  |  | 16.37 | 59.88 | 52.79 |  |  |
| 15.71 | 66.91 | 62.66 |  |  | 16.38 | 59.80 | 52.77 |  |  |
| 15.72 | 66.89 | 62.64 |  |  | 16.39 | 59.72 | 52.74 |  |  |
| 15.73 | 66.86 | 62.61 |  |  | 16.40 | 59.67 | 52.71 |  |  |
| 15.74 | 66.84 | 62.58 |  |  | 16.41 | 59.62 | 52.69 |  |  |
| 15.75 | 66.82 | 62.55 |  |  | 16.42 | 59.53 | 52.66 |  |  |
| 15.76 | 66.79 | 62.52 |  |  | 16.43 | 59.40 | 52.63 |  |  |
| 15.77 | 66.76 | 62.50 |  |  | 16.44 | 59.28 | 52.60 |  |  |
| 15.78 | 66.73 | 62.47 |  |  | 16.45 | 59.21 | 52.58 |  |  |
| 15.79 | 66.70 | 62.44 |  |  | 16.46 | 59.12 | 52.55 |  |  |
| 15.80 | 66.67 | 62.41 |  |  | 16.47 | 59.01 | 52.52 |  |  |
| 15.81 | 66.65 | 62.39 |  |  | 16.48 | 58.94 | 52.50 |  |  |
| 15.82 | 66.62 | 62.36 |  |  | 16.49 | 58.83 | 52.47 |  |  |
| 15.83 | 66.59 | 62.33 |  |  | 16.50 | 58.75 | 52.41 |  |  |
| 15.84 | 66.57 | 62.30 |  |  | 16.51 | 58.66 | 52.39 |  |  |
| 15.85 | 66.55 | 62.27 |  |  | 16.52 | 58.56 | 52.36 |  |  |
| 15.86 | 66.53 | 62.25 |  |  | 16.53 | 58.48 | 52.33 |  |  |
| 15.87 | 66.49 | 62.22 |  |  | 16.54 | 58.38 | 52.31 |  |  |
| 15.88 | 66.47 | 62.19 |  |  | 16.55 | 58.29 | 52.28 |  |  |
| 15.89 | 66.44 | 62.16 |  |  | 16.56 | 58.22 | 52.25 |  |  |
| 15.90 | 66.41 | 62.14 |  |  | 16.57 | 58.12 | 52.23 |  |  |
| 15.91 | 66.36 | 62.11 |  |  | 16.58 | 58.02 | 52.20 |  |  |
| 15.92 | 66.34 | 62.08 |  |  | 16.59 | 57.92 | 52.17 |  |  |
| 15.93 | 66.31 | 62.05 |  |  | 16.60 | 57.83 | 52.14 |  |  |
| 15.94 | 66.29 | 62.02 |  |  | 16.61 | 57.75 | 52.12 |  |  |
| 15.95 | 66.27 | 62.00 |  |  | 16.62 | 57.64 | 52.09 |  |  |
| 15.96 | 66.24 | 61.97 |  |  | 16.63 | 57.53 | 52.06 |  |  |
| 15.97 | 66.22 | 61.94 |  |  | 16.64 | 57.41 | 52.04 |  |  |
| 15.98 | 66.19 | 61.91 |  |  | 16.65 | 57.32 | 52.01 |  |  |
| 15.99 | 66.18 | 61.89 |  |  | 16.67 | 57.15 | 51.98 |  |  |
| 16.00 | 66.17 | 61.86 |  |  | 16.68 | 57.06 | 51.95 |  |  |
| 16.01 | 66.14 | 61.83 |  |  | 16.69 | 56.96 | 51.93 |  |  |
| 16.02 | 66.12 | 61.80 |  |  | 16.70 | 56.87 | 51.90 |  |  |
| 16.03 | 66.08 | 61.78 |  |  | 16.73 | 56.54 | 51.87 |  |  |
| 16.04 | 66.05 | 61.75 |  |  | 16.74 | 56.44 | 51.85 |  |  |
| 16.05 | 66.02 | 61.72 |  |  | 16.75 | 56.34 | 51.82 |  |  |
| 16.06 | 66.01 | 61.69 |  |  | 16.76 | 56.26 | 51.77 |  |  |
| 16.07 | 65.97 | 61.66 |  |  | 16.77 | 56.18 | 51.74 |  |  |
| 16.08 | 65.94 | 61.64 |  |  | 16.78 | 56.07 | 51.71 |  |  |
| 16.09 | 65.91 | 61.61 |  |  | 16.79 | 55.99 | 51.68 |  |  |
| 16.10 | 65.89 | 61.58 |  |  | 16.80 | 55.90 | 51.66 |  |  |
| 16.11 | 65.86 | 61.55 |  |  | 16.81 | 55.78 | 51.63 |  |  |
| 16.12 | 65.82 | 61.53 |  |  | 16.82 | 55.71 | 51.58 |  |  |
| 16.13 | 65.79 | 61.50 |  |  | 16.83 | 55.58 | 51.55 |  |  |
| 16.14 | 65.76 | 61.47 |  |  | 16.84 | 55.49 | 51.49 |  |  |
| 16.15 | 65.72 | 61.44 |  |  | 16.85 | 55.42 | 51.47 |  |  |
| 16.16 | 65.69 | 61.41 |  |  | 16.86 | 55.30 | 51.44 |  |  |
| 16.17 | 65.67 | 61.39 |  |  | 16.87 | 55.22 | 51.41 |  |  |
| 16.18 | 65.63 | 61.36 |  |  | 16.88 | 55.10 | 51.39 |  |  |
| 16.19 | 65.61 | 61.33 |  |  | 16.89 | 55.03 | 51.36 |  |  |
| 16.20 | 65.58 | 61.30 |  |  | 16.90 | 54.94 | 51.33 |  |  |
| 16.21 | 65.55 | 61.28 |  |  | 16.91 | 54.85 | 51.31 |  |  |
| 16.22 | 65.52 | 61.25 |  |  | 16.92 | 54.77 | 51.25 |  |  |
| 16.23 | 65.48 | 61.22 |  |  | 16.93 | 54.69 | 51.22 |  |  |
| 16.24 | 65.44 | 61.19 |  |  | 16.94 | 54.56 | 51.17 |  |  |
| 16.26 | 65.39 | 61.14 |  |  | 16.95 | 54.45 | 51.14 |  |  |
| 16.27 | 65.36 | 61.11 |  |  | 16.96 | 54.35 | 51.12 |  |  |
| 16.28 | 65.33 | 61.08 |  |  | 16.97 | 54.24 | 51.09 |  |  |
| 16.29 | 65.29 | 61.05 |  |  | 16.98 | 54.17 | 51.06 |  |  |
| 16.30 | 65.28 | 61.03 |  |  | 16.99 | 54.09 | 51.03 |  |  |
| 16.31 | 65.26 | 61.00 |  |  | 17.00 | 53.99 | 51.01 |  |  |
| 16.32 | 65.22 | 60.97 |  |  | 17.01 | 53.88 | 50.98 |  |  |
| 16.33 | 65.20 | 60.94 |  |  | 17.02 | 53.78 | 50.95 |  |  |
| 16.34 | 65.16 | 60.91 |  |  | 17.03 | 53.68 | 50.93 |  |  |
| 16.35 | 65.11 | 60.89 |  |  | 17.04 | 53.57 | 50.90 |  |  |
| 16.36 | 65.08 | 60.86 |  |  | 17.05 | 53.49 | 50.87 |  |  |
| 16.37 | 65.05 | 60.83 |  |  | 17.06 | 53.40 | 50.85 |  |  |
| 16.38 | 65.03 | 60.80 |  |  | 17.07 | 53.29 | 50.79 |  |  |
| 16.39 | 64.99 | 60.78 |  |  | 17.08 | 53.17 | 50.76 |  |  |
| 16.41 | 64.93 | 60.72 |  |  | 17.09 | 53.05 | 50.74 |  |  |
| 16.43 | 64.86 | 60.66 |  |  | 17.10 | 52.96 | 50.71 |  |  |
| 16.44 | 64.83 | 60.64 |  |  | 17.11 | 52.86 | 50.68 |  |  |
| 16.45 | 64.81 | 60.61 |  |  | 17.12 | 52.75 | 50.66 |  |  |
| 16.46 | 64.78 | 60.58 |  |  | 17.13 | 52.63 | 50.63 |  |  |
| 16.47 | 64.77 | 60.55 |  |  | 17.14 | 52.55 | 50.60 |  |  |
| 16.48 | 64.74 | 60.53 |  |  | 17.15 | 52.46 | 50.57 |  |  |
| 16.49 | 64.70 | 60.50 |  |  | 17.16 | 52.35 | 50.55 |  |  |
| 16.51 | 64.65 | 60.44 |  |  | 17.17 | 52.23 | 50.52 |  |  |
| 16.52 | 64.61 | 60.41 |  |  | 17.18 | 52.12 | 50.49 |  |  |
| 16.53 | 64.58 | 60.39 |  |  | 17.19 | 52.03 | 50.47 |  |  |
| 16.54 | 64.54 | 60.36 |  |  | 17.20 | 51.91 | 50.44 |  |  |
| 16.55 | 64.50 | 60.33 |  |  | 17.21 | 51.81 | 50.41 |  |  |
| 16.56 | 64.47 | 60.30 |  |  | 17.22 | 51.71 | 50.39 |  |  |
| 16.57 | 64.44 | 60.28 |  |  | 17.23 | 51.59 | 50.36 |  |  |
| 16.58 | 64.41 | 60.25 |  |  | 17.24 | 51.50 | 50.33 |  |  |
| 16.59 | 64.39 | 60.22 |  |  | 17.26 | 51.30 | 50.28 |  |  |
| 16.60 | 64.38 | 60.19 |  |  | 17.27 | 51.21 | 50.22 |  |  |
| 16.61 | 64.33 | 60.16 |  |  | 17.28 | 51.12 | 50.20 |  |  |
| 16.62 | 64.30 | 60.14 |  |  | 17.29 | 51.03 | 50.17 |  |  |
| 16.64 | 64.24 | 60.08 |  |  | 17.30 | 50.94 | 50.14 |  |  |
| 16.65 | 64.21 | 60.05 |  |  | 17.31 | 50.87 | 50.11 |  |  |
| 16.66 | 64.16 | 60.03 |  |  | 17.32 | 50.75 | 50.09 |  |  |
| 16.67 | 64.12 | 60.00 |  |  | 17.33 | 50.67 | 50.06 |  |  |
| 16.68 | 64.08 | 59.97 |  |  | 17.34 | 50.58 | 50.03 |  |  |
| 16.70 | 64.02 | 59.92 |  |  | 17.35 | 50.49 | 50.01 |  |  |
| 16.71 | 64.00 | 59.89 |  |  | 17.36 | 50.39 | 49.98 |  |  |
| 16.73 | 63.92 | 59.83 |  |  | 17.37 | 50.29 | 49.95 |  |  |
| 16.74 | 63.90 | 59.80 |  |  | 17.38 | 50.19 | 49.93 |  |  |
| 16.76 | 63.84 | 59.75 |  |  | 17.39 | 50.11 | 49.90 |  |  |
| 16.77 | 63.80 | 59.72 |  |  | 17.40 | 49.99 | 49.87 |  |  |
| 16.78 | 63.76 | 59.69 |  |  | 17.41 | 49.90 | 49.84 |  |  |
| 16.79 | 63.73 | 59.67 |  |  | 17.42 | 49.79 | 49.82 |  |  |
| 16.80 | 63.69 | 59.64 |  |  | 17.43 | 49.71 | 49.79 |  |  |
| 16.81 | 63.66 | 59.61 |  |  | 17.44 | 49.61 | 49.76 |  |  |
| 16.82 | 63.62 | 59.58 |  |  | 17.45 | 49.53 | 49.74 |  |  |
| 16.83 | 63.60 | 59.55 |  |  | 17.46 | 49.44 | 49.71 |  |  |
| 16.84 | 63.56 | 59.53 |  |  | 17.47 | 49.36 | 49.68 |  |  |
| 16.85 | 63.53 | 59.50 |  |  | 17.48 | 49.28 | 49.65 |  |  |
| 16.86 | 63.50 | 59.47 |  |  | 17.49 | 49.20 | 49.63 |  |  |
| 16.87 | 63.48 | 59.44 |  |  | 17.50 | 49.12 | 49.57 |  |  |
| 16.88 | 63.44 | 59.42 |  |  | 17.51 | 49.02 | 49.55 |  |  |
| 16.89 | 63.41 | 59.39 |  |  | 17.52 | 48.93 | 49.52 |  |  |
| 16.90 | 63.39 | 59.36 |  |  | 17.53 | 48.84 | 49.49 |  |  |
| 16.91 | 63.37 | 59.33 |  |  | 17.54 | 48.74 | 49.47 |  |  |
| 16.92 | 63.34 | 59.30 |  |  | 17.55 | 48.65 | 49.44 |  |  |
| 16.94 | 63.28 | 59.25 |  |  | 17.56 | 48.53 | 49.41 |  |  |
| 16.95 | 63.25 | 59.22 |  |  | 17.57 | 48.45 | 49.38 |  |  |
| 16.96 | 63.24 | 59.19 |  |  | 17.58 | 48.38 | 49.36 |  |  |
| 16.98 | 63.19 | 59.14 |  |  | 17.59 | 48.28 | 49.33 |  |  |
| 16.99 | 63.16 | 59.11 |  |  | 17.60 | 48.19 | 49.30 |  |  |
| 17.01 | 63.09 | 59.05 |  |  | 17.61 | 48.11 | 49.28 |  |  |
| 17.02 | 63.05 | 59.03 |  |  | 17.62 | 48.02 | 49.22 |  |  |
| 17.03 | 63.04 | 59.00 |  |  | 17.63 | 47.91 | 49.19 |  |  |
| 17.04 | 63.00 | 58.97 |  |  | 17.64 | 47.80 | 49.17 |  |  |
| 17.05 | 62.97 | 58.94 |  |  | 17.65 | 47.72 | 49.14 |  |  |
| 17.07 | 62.90 | 58.89 |  |  | 17.66 | 47.62 | 49.11 |  |  |
| 17.08 | 62.88 | 58.86 |  |  | 17.68 | 47.45 | 49.09 |  |  |
| 17.09 | 62.85 | 58.83 |  |  | 17.69 | 47.35 | 49.06 |  |  |
| 17.10 | 62.82 | 58.80 |  |  | 17.70 | 47.25 | 49.01 |  |  |
| 17.11 | 62.78 | 58.78 |  |  | 17.71 | 47.15 | 48.98 |  |  |
| 17.12 | 62.75 | 58.75 |  |  | 17.72 | 47.05 | 48.95 |  |  |
| 17.13 | 62.72 | 58.72 |  |  | 17.73 | 46.96 | 48.92 |  |  |
| 17.14 | 62.69 | 58.69 |  |  | 17.74 | 46.87 | 48.90 |  |  |
| 17.15 | 62.64 | 58.67 |  |  | 17.75 | 46.76 | 48.87 |  |  |
| 17.16 | 62.63 | 58.64 |  |  | 17.76 | 46.68 | 48.84 |  |  |
| 17.17 | 62.59 | 58.61 |  |  | 17.77 | 46.60 | 48.82 |  |  |
| 17.19 | 62.52 | 58.55 |  |  | 17.78 | 46.52 | 48.79 |  |  |
| 17.20 | 62.47 | 58.53 |  |  | 17.79 | 46.42 | 48.76 |  |  |
| 17.21 | 62.44 | 58.50 |  |  | 17.80 | 46.32 | 48.73 |  |  |
| 17.22 | 62.41 | 58.47 |  |  | 17.81 | 46.21 | 48.71 |  |  |
| 17.23 | 62.39 | 58.44 |  |  | 17.82 | 46.11 | 48.68 |  |  |
| 17.24 | 62.35 | 58.42 |  |  | 17.83 | 46.02 | 48.65 |  |  |
| 17.25 | 62.30 | 58.39 |  |  | 17.84 | 45.92 | 48.60 |  |  |
| 17.26 | 62.25 | 58.36 |  |  | 17.85 | 45.82 | 48.57 |  |  |
| 17.27 | 62.22 | 58.33 |  |  | 17.86 | 45.74 | 48.55 |  |  |
| 17.28 | 62.19 | 58.31 |  |  | 17.87 | 45.63 | 48.52 |  |  |
| 17.29 | 62.15 | 58.28 |  |  | 17.88 | 45.55 | 48.49 |  |  |
| 17.30 | 62.14 | 58.25 |  |  | 17.89 | 45.45 | 48.46 |  |  |
| 17.31 | 62.10 | 58.22 |  |  | 17.90 | 45.36 | 48.44 |  |  |
| 17.32 | 62.06 | 58.19 |  |  | 17.91 | 45.25 | 48.41 |  |  |
| 17.33 | 62.04 | 58.17 |  |  | 17.92 | 45.14 | 48.38 |  |  |
| 17.34 | 62.00 | 58.14 |  |  | 17.93 | 45.04 | 48.36 |  |  |
| 17.35 | 61.97 | 58.11 |  |  | 17.94 | 44.95 | 48.33 |  |  |
| 17.36 | 61.93 | 58.08 |  |  | 17.95 | 44.87 | 48.30 |  |  |
| 17.37 | 61.89 | 58.06 |  |  | 17.96 | 44.78 | 48.27 |  |  |
| 17.38 | 61.86 | 58.03 |  |  | 17.97 | 44.67 | 48.25 |  |  |
| 17.39 | 61.83 | 58.00 |  |  | 17.98 | 44.57 | 48.22 |  |  |
| 17.40 | 61.80 | 57.97 |  |  | 17.99 | 44.44 | 48.19 |  |  |
| 17.41 | 61.75 | 57.94 |  |  | 18.00 | 44.35 | 48.17 |  |  |
| 17.42 | 61.72 | 57.92 |  |  | 18.01 | 44.23 | 48.14 |  |  |
| 17.43 | 61.68 | 57.89 |  |  | 18.02 | 44.12 | 48.11 |  |  |
| 17.44 | 61.65 | 57.86 |  |  | 18.03 | 44.02 | 48.09 |  |  |
| 17.45 | 61.62 | 57.83 |  |  | 18.04 | 43.93 | 48.03 |  |  |
| 17.46 | 61.61 | 57.81 |  |  | 18.05 | 43.81 | 48.00 |  |  |
| 17.47 | 61.57 | 57.78 |  |  | 18.06 | 43.73 | 47.98 |  |  |
| 17.48 | 61.52 | 57.75 |  |  | 18.07 | 43.60 | 47.92 |  |  |
| 17.49 | 61.48 | 57.72 |  |  | 18.08 | 43.49 | 47.90 |  |  |
| 17.50 | 61.45 | 57.69 |  |  | 18.09 | 43.40 | 47.87 |  |  |
| 17.51 | 61.41 | 57.67 |  |  | 18.10 | 43.30 | 47.84 |  |  |
| 17.52 | 61.37 | 57.64 |  |  | 18.11 | 43.20 | 47.79 |  |  |
| 17.53 | 61.35 | 57.61 |  |  | 18.12 | 43.11 | 47.76 |  |  |
| 17.54 | 61.31 | 57.58 |  |  | 18.13 | 43.00 | 47.73 |  |  |
| 17.55 | 61.27 | 57.56 |  |  | 18.14 | 42.93 | 47.71 |  |  |
| 17.56 | 61.23 | 57.53 |  |  | 18.15 | 42.84 | 47.68 |  |  |
| 17.57 | 61.20 | 57.50 |  |  | 18.16 | 42.75 | 47.65 |  |  |
| 17.59 | 61.13 | 57.44 |  |  | 18.17 | 42.65 | 47.63 |  |  |
| 17.60 | 61.08 | 57.42 |  |  | 18.18 | 42.56 | 47.60 |  |  |
| 17.61 | 61.04 | 57.39 |  |  | 18.19 | 42.47 | 47.57 |  |  |
| 17.62 | 61.01 | 57.36 |  |  | 18.20 | 42.39 | 47.54 |  |  |
| 17.63 | 60.97 | 57.33 |  |  | 18.21 | 42.28 | 47.49 |  |  |
| 17.64 | 60.94 | 57.31 |  |  | 18.22 | 42.15 | 47.46 |  |  |
| 17.66 | 60.88 | 57.25 |  |  | 18.23 | 42.09 | 47.44 |  |  |
| 17.67 | 60.83 | 57.22 |  |  | 18.24 | 41.97 | 47.41 |  |  |
| 17.68 | 60.79 | 57.19 |  |  | 18.25 | 41.85 | 47.38 |  |  |
| 17.69 | 60.75 | 57.17 |  |  | 18.26 | 41.75 | 47.35 |  |  |
| 17.70 | 60.70 | 57.14 |  |  | 18.27 | 41.64 | 47.33 |  |  |
| 17.72 | 60.61 | 57.08 |  |  | 18.28 | 41.50 | 47.30 |  |  |
| 17.73 | 60.58 | 57.06 |  |  | 18.29 | 41.38 | 47.27 |  |  |
| 17.74 | 60.52 | 57.03 |  |  | 18.30 | 41.27 | 47.22 |  |  |
| 17.75 | 60.47 | 57.00 |  |  | 18.31 | 41.15 | 47.17 |  |  |
| 17.76 | 60.43 | 56.97 |  |  | 18.32 | 41.04 | 47.14 |  |  |
| 17.77 | 60.41 | 56.94 |  |  | 18.33 | 40.96 | 47.11 |  |  |
| 17.78 | 60.36 | 56.92 |  |  | 18.34 | 40.87 | 47.08 |  |  |
| 17.80 | 60.29 | 56.86 |  |  | 18.35 | 40.79 | 47.06 |  |  |
| 17.82 | 60.20 | 56.81 |  |  | 18.36 | 40.68 | 47.03 |  |  |
| 17.83 | 60.17 | 56.78 |  |  | 18.37 | 40.58 | 47.00 |  |  |
| 17.84 | 60.14 | 56.75 |  |  | 18.38 | 40.49 | 46.98 |  |  |
| 17.85 | 60.10 | 56.72 |  |  | 18.39 | 40.37 | 46.95 |  |  |
| 17.86 | 60.05 | 56.69 |  |  | 18.40 | 40.26 | 46.92 |  |  |
| 17.88 | 59.98 | 56.64 |  |  | 18.41 | 40.16 | 46.89 |  |  |
| 17.89 | 59.94 | 56.61 |  |  | 18.42 | 40.05 | 46.87 |  |  |
| 17.90 | 59.90 | 56.58 |  |  | 18.43 | 39.94 | 46.84 |  |  |
| 17.91 | 59.86 | 56.56 |  |  | 18.44 | 39.83 | 46.81 |  |  |
| 17.92 | 59.80 | 56.53 |  |  | 18.45 | 39.72 | 46.79 |  |  |
| 17.93 | 59.75 | 56.50 |  |  | 18.46 | 39.60 | 46.76 |  |  |
| 17.94 | 59.72 | 56.47 |  |  | 18.47 | 39.48 | 46.73 |  |  |
| 17.95 | 59.68 | 56.45 |  |  | 18.48 | 39.40 | 46.71 |  |  |
| 17.96 | 59.65 | 56.42 |  |  | 18.49 | 39.31 | 46.68 |  |  |
| 17.97 | 59.60 | 56.39 |  |  | 18.50 | 39.22 | 46.65 |  |  |
| 17.98 | 59.55 | 56.36 |  |  | 18.51 | 39.11 | 46.62 |  |  |
| 17.99 | 59.51 | 56.33 |  |  | 18.52 | 39.01 | 46.60 |  |  |
| 18.00 | 59.47 | 56.31 |  |  | 18.53 | 38.92 | 46.57 |  |  |
| 18.02 | 59.40 | 56.25 |  |  | 18.54 | 38.81 | 46.54 |  |  |
| 18.03 | 59.36 | 56.22 |  |  | 18.55 | 38.69 | 46.49 |  |  |
| 18.04 | 59.32 | 56.20 |  |  | 18.56 | 38.61 | 46.46 |  |  |
| 18.06 | 59.24 | 56.14 |  |  | 18.57 | 38.54 | 46.43 |  |  |
| 18.07 | 59.20 | 56.11 |  |  | 18.58 | 38.43 | 46.41 |  |  |
| 18.09 | 59.11 | 56.06 |  |  | 18.59 | 38.35 | 46.38 |  |  |
| 18.11 | 59.01 | 56.00 |  |  | 18.60 | 38.26 | 46.35 |  |  |
| 18.12 | 58.99 | 55.97 |  |  | 18.61 | 38.17 | 46.33 |  |  |
| 18.13 | 58.94 | 55.95 |  |  | 18.62 | 38.05 | 46.30 |  |  |
| 18.14 | 58.90 | 55.92 |  |  | 18.63 | 37.96 | 46.27 |  |  |
| 18.15 | 58.85 | 55.89 |  |  | 18.64 | 37.88 | 46.25 |  |  |
| 18.16 | 58.79 | 55.86 |  |  | 18.65 | 37.79 | 46.22 |  |  |
| 18.17 | 58.77 | 55.83 |  |  | 18.67 | 37.62 | 46.19 |  |  |
| 18.19 | 58.68 | 55.78 |  |  | 18.68 | 37.52 | 46.16 |  |  |
| 18.20 | 58.64 | 55.75 |  |  | 18.69 | 37.45 | 46.14 |  |  |
| 18.21 | 58.60 | 55.72 |  |  | 18.70 | 37.34 | 46.11 |  |  |
| 18.22 | 58.56 | 55.70 |  |  | 18.72 | 37.13 | 46.08 |  |  |
| 18.23 | 58.53 | 55.67 |  |  | 18.74 | 36.91 | 46.06 |  |  |
| 18.24 | 58.48 | 55.64 |  |  | 18.75 | 36.80 | 46.03 |  |  |
| 18.26 | 58.40 | 55.58 |  |  | 18.76 | 36.67 | 46.00 |  |  |
| 18.27 | 58.36 | 55.56 |  |  | 18.77 | 36.58 | 45.97 |  |  |
| 18.28 | 58.32 | 55.53 |  |  | 18.78 | 36.49 | 45.95 |  |  |
| 18.29 | 58.27 | 55.50 |  |  | 18.79 | 36.38 | 45.92 |  |  |
| 18.30 | 58.23 | 55.47 |  |  | 18.80 | 36.28 | 45.89 |  |  |
| 18.31 | 58.18 | 55.45 |  |  | 18.81 | 36.17 | 45.87 |  |  |
| 18.32 | 58.15 | 55.42 |  |  | 18.82 | 36.09 | 45.84 |  |  |
| 18.33 | 58.13 | 55.39 |  |  | 18.83 | 35.98 | 45.81 |  |  |
| 18.34 | 58.08 | 55.36 |  |  | 18.84 | 35.86 | 45.79 |  |  |
| 18.35 | 58.03 | 55.33 |  |  | 18.85 | 35.72 | 45.76 |  |  |
| 18.36 | 57.98 | 55.31 |  |  | 18.86 | 35.61 | 45.73 |  |  |
| 18.37 | 57.93 | 55.28 |  |  | 18.87 | 35.54 | 45.70 |  |  |
| 18.38 | 57.90 | 55.25 |  |  | 18.88 | 35.44 | 45.68 |  |  |
| 18.39 | 57.87 | 55.22 |  |  | 18.89 | 35.36 | 45.65 |  |  |
| 18.40 | 57.84 | 55.20 |  |  | 18.90 | 35.26 | 45.62 |  |  |
| 18.41 | 57.81 | 55.17 |  |  | 18.91 | 35.13 | 45.60 |  |  |
| 18.43 | 57.72 | 55.11 |  |  | 18.92 | 35.05 | 45.57 |  |  |
| 18.44 | 57.67 | 55.08 |  |  | 18.93 | 34.96 | 45.54 |  |  |
| 18.45 | 57.63 | 55.06 |  |  | 18.94 | 34.86 | 45.51 |  |  |
| 18.46 | 57.60 | 55.03 |  |  | 18.95 | 34.76 | 45.49 |  |  |
| 18.47 | 57.56 | 55.00 |  |  | 18.96 | 34.66 | 45.43 |  |  |
| 18.48 | 57.54 | 54.97 |  |  | 18.97 | 34.56 | 45.41 |  |  |
| 18.49 | 57.50 | 54.95 |  |  | 18.98 | 34.44 | 45.38 |  |  |
| 18.50 | 57.46 | 54.92 |  |  | 18.99 | 34.35 | 45.35 |  |  |
| 18.51 | 57.40 | 54.89 |  |  | 19.00 | 34.24 | 45.33 |  |  |
| 18.52 | 57.36 | 54.86 |  |  | 19.01 | 34.17 | 45.30 |  |  |
| 18.53 | 57.33 | 54.84 |  |  | 19.02 | 34.07 | 45.27 |  |  |
| 18.54 | 57.29 | 54.81 |  |  | 19.03 | 33.96 | 45.24 |  |  |
| 18.55 | 57.23 | 54.78 |  |  | 19.04 | 33.85 | 45.22 |  |  |
| 18.56 | 57.20 | 54.75 |  |  | 19.05 | 33.75 | 45.19 |  |  |
| 18.57 | 57.17 | 54.72 |  |  | 19.07 | 33.56 | 45.16 |  |  |
| 18.58 | 57.14 | 54.70 |  |  | 19.08 | 33.48 | 45.14 |  |  |
| 18.59 | 57.10 | 54.67 |  |  | 19.09 | 33.37 | 45.11 |  |  |
| 18.60 | 57.05 | 54.64 |  |  | 19.10 | 33.26 | 45.08 |  |  |
| 18.61 | 57.02 | 54.61 |  |  | 19.11 | 33.16 | 45.05 |  |  |
| 18.62 | 57.00 | 54.59 |  |  | 19.12 | 33.07 | 45.03 |  |  |
| 18.63 | 56.95 | 54.56 |  |  | 19.13 | 32.98 | 45.00 |  |  |
| 18.64 | 56.88 | 54.53 |  |  | 19.14 | 32.88 | 44.97 |  |  |
| 18.65 | 56.85 | 54.50 |  |  | 19.16 | 32.69 | 44.92 |  |  |
| 18.66 | 56.82 | 54.47 |  |  | 19.17 | 32.60 | 44.89 |  |  |
| 18.67 | 56.78 | 54.45 |  |  | 19.18 | 32.52 | 44.87 |  |  |
| 18.68 | 56.75 | 54.42 |  |  | 19.19 | 32.41 | 44.84 |  |  |
| 18.69 | 56.71 | 54.39 |  |  | 19.20 | 32.31 | 44.81 |  |  |
| 18.70 | 56.65 | 54.36 |  |  | 19.22 | 32.09 | 44.78 |  |  |
| 18.71 | 56.62 | 54.34 |  |  | 19.23 | 31.98 | 44.76 |  |  |
| 18.72 | 56.59 | 54.31 |  |  | 19.24 | 31.90 | 44.73 |  |  |
| 18.73 | 56.55 | 54.28 |  |  | 19.25 | 31.79 | 44.70 |  |  |
| 18.74 | 56.50 | 54.25 |  |  | 19.26 | 31.68 | 44.68 |  |  |
| 18.75 | 56.46 | 54.22 |  |  | 19.27 | 31.60 | 44.65 |  |  |
| 18.76 | 56.41 | 54.20 |  |  | 19.28 | 31.50 | 44.59 |  |  |
| 18.77 | 56.36 | 54.17 |  |  | 19.29 | 31.39 | 44.57 |  |  |
| 18.78 | 56.32 | 54.14 |  |  | 19.30 | 31.32 | 44.54 |  |  |
| 18.79 | 56.28 | 54.11 |  |  | 19.31 | 31.22 | 44.51 |  |  |
| 18.80 | 56.26 | 54.09 |  |  | 19.32 | 31.12 | 44.49 |  |  |
| 18.81 | 56.22 | 54.06 |  |  | 19.33 | 31.02 | 44.46 |  |  |
| 18.82 | 56.20 | 54.03 |  |  | 19.34 | 30.92 | 44.43 |  |  |
| 18.83 | 56.16 | 54.00 |  |  | 19.35 | 30.80 | 44.38 |  |  |
| 18.84 | 56.11 | 53.97 |  |  | 19.36 | 30.68 | 44.35 |  |  |
| 18.85 | 56.09 | 53.95 |  |  | 19.37 | 30.59 | 44.32 |  |  |
| 18.86 | 56.06 | 53.92 |  |  | 19.38 | 30.51 | 44.30 |  |  |
| 18.87 | 56.03 | 53.89 |  |  | 19.39 | 30.42 | 44.27 |  |  |
| 18.88 | 55.99 | 53.86 |  |  | 19.40 | 30.32 | 44.24 |  |  |
| 18.90 | 55.92 | 53.81 |  |  | 19.41 | 30.23 | 44.22 |  |  |
| 18.91 | 55.90 | 53.78 |  |  | 19.42 | 30.16 | 44.19 |  |  |
| 18.92 | 55.87 | 53.75 |  |  | 19.43 | 30.04 | 44.16 |  |  |
| 18.93 | 55.84 | 53.72 |  |  | 19.44 | 29.97 | 44.13 |  |  |
| 18.94 | 55.81 | 53.70 |  |  | 19.45 | 29.88 | 44.11 |  |  |
| 18.95 | 55.78 | 53.67 |  |  | 19.46 | 29.79 | 44.08 |  |  |
| 18.96 | 55.73 | 53.64 |  |  | 19.47 | 29.68 | 44.05 |  |  |
| 18.97 | 55.71 | 53.61 |  |  | 19.48 | 29.59 | 44.03 |  |  |
| 18.98 | 55.67 | 53.59 |  |  | 19.49 | 29.51 | 44.00 |  |  |
| 18.99 | 55.64 | 53.56 |  |  | 19.50 | 29.43 | 43.97 |  |  |
| 19.00 | 55.61 | 53.53 |  |  | 19.51 | 29.35 | 43.95 |  |  |
| 19.01 | 55.58 | 53.50 |  |  | 19.52 | 29.25 | 43.92 |  |  |
| 19.02 | 55.54 | 53.47 |  |  | 19.53 | 29.16 | 43.86 |  |  |
| 19.03 | 55.51 | 53.45 |  |  | 19.54 | 29.05 | 43.84 |  |  |
| 19.04 | 55.48 | 53.42 |  |  | 19.55 | 28.92 | 43.81 |  |  |
| 19.05 | 55.44 | 53.39 |  |  | 19.56 | 28.84 | 43.76 |  |  |
| 19.06 | 55.40 | 53.36 |  |  | 19.57 | 28.74 | 43.73 |  |  |
| 19.07 | 55.36 | 53.34 |  |  | 19.58 | 28.66 | 43.70 |  |  |
| 19.08 | 55.33 | 53.31 |  |  | 19.59 | 28.58 | 43.67 |  |  |
| 19.09 | 55.30 | 53.28 |  |  | 19.60 | 28.50 | 43.65 |  |  |
| 19.10 | 55.26 | 53.25 |  |  | 19.61 | 28.39 | 43.62 |  |  |
| 19.11 | 55.24 | 53.22 |  |  | 19.62 | 28.31 | 43.59 |  |  |
| 19.12 | 55.21 | 53.20 |  |  | 19.63 | 28.20 | 43.57 |  |  |
| 19.13 | 55.17 | 53.17 |  |  | 19.64 | 28.09 | 43.54 |  |  |
| 19.14 | 55.11 | 53.14 |  |  | 19.65 | 28.00 | 43.51 |  |  |
| 19.15 | 55.08 | 53.11 |  |  | 19.66 | 27.92 | 43.49 |  |  |
| 19.16 | 55.05 | 53.09 |  |  | 19.67 | 27.81 | 43.46 |  |  |
| 19.18 | 55.01 | 53.03 |  |  | 19.68 | 27.69 | 43.43 |  |  |
| 19.19 | 54.99 | 53.00 |  |  | 19.70 | 27.56 | 43.40 |  |  |
| 19.20 | 54.95 | 52.98 |  |  | 19.71 | 27.48 | 43.38 |  |  |
| 19.21 | 54.92 | 52.95 |  |  | 19.72 | 27.41 | 43.35 |  |  |
| 19.22 | 54.90 | 52.92 |  |  | 19.73 | 27.34 | 43.32 |  |  |
| 19.23 | 54.87 | 52.89 |  |  | 19.74 | 27.24 | 43.30 |  |  |
| 19.24 | 54.83 | 52.86 |  |  | 19.75 | 27.17 | 43.27 |  |  |
| 19.25 | 54.80 | 52.84 |  |  | 19.76 | 27.10 | 43.24 |  |  |
| 19.26 | 54.77 | 52.81 |  |  | 19.77 | 27.03 | 43.19 |  |  |
| 19.27 | 54.74 | 52.78 |  |  | 19.78 | 26.96 | 43.16 |  |  |
| 19.28 | 54.71 | 52.75 |  |  | 19.79 | 26.89 | 43.13 |  |  |
| 19.29 | 54.68 | 52.73 |  |  | 19.80 | 26.81 | 43.11 |  |  |
| 19.30 | 54.62 | 52.70 |  |  | 19.81 | 26.74 | 43.08 |  |  |
| 19.31 | 54.58 | 52.67 |  |  | 19.82 | 26.67 | 43.05 |  |  |
| 19.32 | 54.55 | 52.64 |  |  | 19.83 | 26.60 | 43.03 |  |  |
| 19.33 | 54.52 | 52.61 |  |  | 19.84 | 26.52 | 42.97 |  |  |
| 19.34 | 54.49 | 52.59 |  |  | 19.85 | 26.46 | 42.92 |  |  |
| 19.35 | 54.44 | 52.56 |  |  | 19.86 | 26.39 | 42.89 |  |  |
| 19.36 | 54.42 | 52.53 |  |  | 19.87 | 26.31 | 42.86 |  |  |
| 19.37 | 54.39 | 52.50 |  |  | 19.88 | 26.25 | 42.84 |  |  |
| 19.38 | 54.35 | 52.48 |  |  | 19.89 | 26.15 | 42.81 |  |  |
| 19.39 | 54.31 | 52.45 |  |  | 19.90 | 26.08 | 42.78 |  |  |
| 19.40 | 54.28 | 52.42 |  |  | 19.91 | 26.00 | 42.75 |  |  |
| 19.41 | 54.24 | 52.39 |  |  | 19.92 | 25.91 | 42.73 |  |  |
| 19.42 | 54.21 | 52.36 |  |  | 19.93 | 25.84 | 42.70 |  |  |
| 19.43 | 54.17 | 52.34 |  |  | 19.94 | 25.77 | 42.67 |  |  |
| 19.44 | 54.13 | 52.31 |  |  | 19.95 | 25.72 | 42.65 |  |  |
| 19.45 | 54.08 | 52.28 |  |  | 19.96 | 25.66 | 42.62 |  |  |
| 19.47 | 54.01 | 52.23 |  |  | 19.97 | 25.57 | 42.59 |  |  |
| 19.48 | 53.97 | 52.20 |  |  | 19.98 | 25.50 | 42.57 |  |  |
| 19.49 | 53.95 | 52.17 |  |  | 19.99 | 25.43 | 42.54 |  |  |
| 19.50 | 53.92 | 52.14 |  |  | 20.00 | 25.37 | 42.48 |  |  |
| 19.51 | 53.90 | 52.11 |  |  | 20.01 | 25.29 | 42.46 |  |  |
| 19.52 | 53.85 | 52.09 |  |  | 20.02 | 25.22 | 42.43 |  |  |
| 19.53 | 53.82 | 52.06 |  |  | 20.03 | 25.16 | 42.40 |  |  |
| 19.54 | 53.79 | 52.03 |  |  | 20.04 | 25.11 | 42.38 |  |  |
| 19.55 | 53.77 | 52.00 |  |  | 20.05 | 25.05 | 42.35 |  |  |
| 19.56 | 53.72 | 51.98 |  |  | 20.06 | 24.98 | 42.29 |  |  |
| 19.57 | 53.68 | 51.95 |  |  | 20.07 | 24.94 | 42.27 |  |  |
| 19.58 | 53.64 | 51.92 |  |  | 20.08 | 24.88 | 42.24 |  |  |
| 19.59 | 53.59 | 51.89 |  |  | 20.09 | 24.82 | 42.21 |  |  |
| 19.60 | 53.56 | 51.86 |  |  | 20.11 | 24.71 | 42.19 |  |  |
| 19.61 | 53.53 | 51.84 |  |  | 20.12 | 24.65 | 42.16 |  |  |
| 19.62 | 53.51 | 51.81 |  |  | 20.14 | 24.54 | 42.13 |  |  |
| 19.64 | 53.45 | 51.75 |  |  | 20.16 | 24.41 | 42.11 |  |  |
| 19.65 | 53.42 | 51.73 |  |  | 20.17 | 24.36 | 42.08 |  |  |
| 19.66 | 53.37 | 51.70 |  |  | 20.18 | 24.30 | 42.05 |  |  |
| 19.67 | 53.34 | 51.67 |  |  | 20.19 | 24.23 | 42.02 |  |  |
| 19.68 | 53.30 | 51.64 |  |  | 20.20 | 24.16 | 42.00 |  |  |
| 19.69 | 53.25 | 51.61 |  |  | 20.21 | 24.11 | 41.94 |  |  |
| 19.70 | 53.21 | 51.59 |  |  | 20.23 | 24.00 | 41.92 |  |  |
| 19.71 | 53.18 | 51.56 |  |  | 20.24 | 23.94 | 41.89 |  |  |
| 19.72 | 53.15 | 51.53 |  |  | 20.25 | 23.88 | 41.86 |  |  |
| 19.73 | 53.11 | 51.50 |  |  | 20.26 | 23.84 | 41.83 |  |  |
| 19.74 | 53.09 | 51.48 |  |  | 20.27 | 23.77 | 41.81 |  |  |
| 19.75 | 53.06 | 51.45 |  |  | 20.28 | 23.72 | 41.78 |  |  |
| 19.76 | 53.03 | 51.42 |  |  | 20.29 | 23.66 | 41.75 |  |  |
| 19.77 | 53.00 | 51.39 |  |  | 20.30 | 23.61 | 41.73 |  |  |
| 19.78 | 52.98 | 51.37 |  |  | 20.31 | 23.57 | 41.70 |  |  |
| 19.79 | 52.94 | 51.34 |  |  | 20.32 | 23.52 | 41.67 |  |  |
| 19.80 | 52.92 | 51.31 |  |  | 20.33 | 23.47 | 41.65 |  |  |
| 19.81 | 52.88 | 51.28 |  |  | 20.34 | 23.39 | 41.62 |  |  |
| 19.82 | 52.85 | 51.25 |  |  | 20.36 | 23.31 | 41.59 |  |  |
| 19.83 | 52.82 | 51.23 |  |  | 20.37 | 23.23 | 41.54 |  |  |
| 19.84 | 52.79 | 51.20 |  |  | 20.38 | 23.18 | 41.48 |  |  |
| 19.85 | 52.76 | 51.17 |  |  | 20.39 | 23.13 | 41.46 |  |  |
| 19.86 | 52.73 | 51.14 |  |  | 20.40 | 23.09 | 41.43 |  |  |
| 19.87 | 52.69 | 51.12 |  |  | 20.41 | 23.03 | 41.40 |  |  |
| 19.88 | 52.65 | 51.09 |  |  | 20.43 | 22.93 | 41.37 |  |  |
| 19.89 | 52.62 | 51.06 |  |  | 20.44 | 22.87 | 41.35 |  |  |
| 19.90 | 52.59 | 51.03 |  |  | 20.45 | 22.83 | 41.32 |  |  |
| 19.91 | 52.55 | 51.00 |  |  | 20.46 | 22.77 | 41.29 |  |  |
| 19.92 | 52.52 | 50.98 |  |  | 20.47 | 22.72 | 41.27 |  |  |
| 19.93 | 52.49 | 50.95 |  |  | 20.48 | 22.65 | 41.24 |  |  |
| 19.94 | 52.43 | 50.92 |  |  | 20.49 | 22.59 | 41.21 |  |  |
| 19.95 | 52.38 | 50.89 |  |  | 20.50 | 22.53 | 41.19 |  |  |
| 19.96 | 52.35 | 50.87 |  |  | 20.51 | 22.49 | 41.16 |  |  |
| 19.97 | 52.32 | 50.84 |  |  | 20.52 | 22.45 | 41.13 |  |  |
| 19.98 | 52.29 | 50.81 |  |  | 20.53 | 22.40 | 41.10 |  |  |
| 20.00 | 52.22 | 50.75 |  |  | 20.54 | 22.35 | 41.08 |  |  |
| 20.01 | 52.17 | 50.73 |  |  | 20.55 | 22.30 | 41.05 |  |  |
| 20.02 | 52.13 | 50.70 |  |  | 20.56 | 22.25 | 41.02 |  |  |
| 20.03 | 52.09 | 50.67 |  |  | 20.57 | 22.20 | 41.00 |  |  |
| 20.04 | 52.06 | 50.64 |  |  | 20.58 | 22.17 | 40.97 |  |  |
| 20.05 | 52.00 | 50.62 |  |  | 20.59 | 22.13 | 40.94 |  |  |
| 20.06 | 51.98 | 50.59 |  |  | 20.60 | 22.09 | 40.92 |  |  |
| 20.07 | 51.94 | 50.56 |  |  | 20.61 | 22.03 | 40.89 |  |  |
| 20.08 | 51.90 | 50.53 |  |  | 20.62 | 21.98 | 40.86 |  |  |
| 20.09 | 51.84 | 50.50 |  |  | 20.63 | 21.94 | 40.83 |  |  |
| 20.10 | 51.81 | 50.48 |  |  | 20.64 | 21.88 | 40.81 |  |  |
| 20.11 | 51.78 | 50.45 |  |  | 20.65 | 21.84 | 40.78 |  |  |
| 20.12 | 51.75 | 50.42 |  |  | 20.66 | 21.78 | 40.75 |  |  |
| 20.13 | 51.70 | 50.39 |  |  | 20.67 | 21.75 | 40.73 |  |  |
| 20.14 | 51.66 | 50.37 |  |  | 20.69 | 21.65 | 40.70 |  |  |
| 20.15 | 51.63 | 50.34 |  |  | 20.70 | 21.62 | 40.67 |  |  |
| 20.16 | 51.60 | 50.31 |  |  | 20.71 | 21.58 | 40.64 |  |  |
| 20.17 | 51.58 | 50.28 |  |  | 20.72 | 21.51 | 40.62 |  |  |
| 20.18 | 51.53 | 50.25 |  |  | 20.73 | 21.45 | 40.56 |  |  |
| 20.19 | 51.50 | 50.23 |  |  | 20.74 | 21.41 | 40.54 |  |  |
| 20.20 | 51.48 | 50.20 |  |  | 20.75 | 21.36 | 40.51 |  |  |
| 20.21 | 51.45 | 50.17 |  |  | 20.76 | 21.32 | 40.48 |  |  |
| 20.22 | 51.42 | 50.14 |  |  | 20.77 | 21.26 | 40.46 |  |  |
| 20.23 | 51.39 | 50.12 |  |  | 20.78 | 21.22 | 40.43 |  |  |
| 20.24 | 51.36 | 50.09 |  |  | 20.79 | 21.19 | 40.40 |  |  |
| 20.25 | 51.33 | 50.06 |  |  | 20.80 | 21.15 | 40.37 |  |  |
| 20.26 | 51.29 | 50.03 |  |  | 20.81 | 21.10 | 40.35 |  |  |
| 20.27 | 51.28 | 50.00 |  |  | 20.83 | 21.01 | 40.32 |  |  |
| 20.28 | 51.24 | 49.98 |  |  | 20.84 | 20.97 | 40.27 |  |  |
| 20.29 | 51.20 | 49.95 |  |  | 20.85 | 20.92 | 40.24 |  |  |
| 20.31 | 51.13 | 49.89 |  |  | 20.86 | 20.87 | 40.21 |  |  |
| 20.32 | 51.09 | 49.87 |  |  | 20.87 | 20.85 | 40.18 |  |  |
| 20.33 | 51.06 | 49.84 |  |  | 20.88 | 20.79 | 40.16 |  |  |
| 20.34 | 51.02 | 49.81 |  |  | 20.89 | 20.75 | 40.13 |  |  |
| 20.35 | 51.00 | 49.78 |  |  | 20.90 | 20.72 | 40.08 |  |  |
| 20.36 | 50.96 | 49.75 |  |  | 20.91 | 20.66 | 40.05 |  |  |
| 20.37 | 50.92 | 49.73 |  |  | 20.92 | 20.62 | 40.02 |  |  |
| 20.38 | 50.88 | 49.70 |  |  | 20.93 | 20.59 | 40.00 |  |  |
| 20.39 | 50.86 | 49.67 |  |  | 20.94 | 20.54 | 39.97 |  |  |
| 20.40 | 50.82 | 49.64 |  |  | 20.95 | 20.50 | 39.94 |  |  |
| 20.41 | 50.80 | 49.62 |  |  | 20.96 | 20.45 | 39.91 |  |  |
| 20.42 | 50.78 | 49.59 |  |  | 20.97 | 20.42 | 39.89 |  |  |
| 20.43 | 50.76 | 49.56 |  |  | 20.98 | 20.37 | 39.86 |  |  |
| 20.45 | 50.68 | 49.51 |  |  | 20.99 | 20.34 | 39.83 |  |  |
| 20.46 | 50.65 | 49.48 |  |  | 21.00 | 20.31 | 39.81 |  |  |
| 20.48 | 50.59 | 49.42 |  |  | 21.01 | 20.26 | 39.78 |  |  |
| 20.49 | 50.57 | 49.39 |  |  | 21.02 | 20.23 | 39.75 |  |  |
| 20.50 | 50.53 | 49.37 |  |  | 21.03 | 20.18 | 39.72 |  |  |
| 20.51 | 50.50 | 49.34 |  |  | 21.04 | 20.12 | 39.64 |  |  |
| 20.52 | 50.48 | 49.31 |  |  | 21.05 | 20.09 | 39.59 |  |  |
| 20.53 | 50.44 | 49.28 |  |  | 21.06 | 20.04 | 39.56 |  |  |
| 20.54 | 50.42 | 49.26 |  |  | 21.08 | 19.98 | 39.54 |  |  |
| 20.55 | 50.40 | 49.23 |  |  | 21.09 | 19.94 | 39.51 |  |  |
| 20.56 | 50.35 | 49.20 |  |  | 21.10 | 19.89 | 39.48 |  |  |
| 20.58 | 50.28 | 49.14 |  |  | 21.12 | 19.81 | 39.45 |  |  |
| 20.59 | 50.25 | 49.12 |  |  | 21.13 | 19.77 | 39.43 |  |  |
| 20.60 | 50.20 | 49.09 |  |  | 21.14 | 19.72 | 39.40 |  |  |
| 20.61 | 50.17 | 49.06 |  |  | 21.15 | 19.68 | 39.37 |  |  |
| 20.62 | 50.14 | 49.03 |  |  | 21.16 | 19.63 | 39.35 |  |  |
| 20.63 | 50.09 | 49.01 |  |  | 21.17 | 19.58 | 39.32 |  |  |
| 20.64 | 50.04 | 48.98 |  |  | 21.18 | 19.55 | 39.29 |  |  |
| 20.65 | 50.01 | 48.95 |  |  | 21.19 | 19.50 | 39.26 |  |  |
| 20.66 | 49.97 | 48.92 |  |  | 21.20 | 19.47 | 39.24 |  |  |
| 20.67 | 49.95 | 48.89 |  |  | 21.21 | 19.42 | 39.21 |  |  |
| 20.68 | 49.92 | 48.87 |  |  | 21.22 | 19.39 | 39.18 |  |  |
| 20.70 | 49.84 | 48.81 |  |  | 21.23 | 19.34 | 39.13 |  |  |
| 20.71 | 49.81 | 48.78 |  |  | 21.24 | 19.31 | 39.10 |  |  |
| 20.72 | 49.76 | 48.76 |  |  | 21.25 | 19.26 | 39.08 |  |  |
| 20.73 | 49.75 | 48.73 |  |  | 21.26 | 19.23 | 39.05 |  |  |
| 20.74 | 49.72 | 48.70 |  |  | 21.27 | 19.16 | 39.02 |  |  |
| 20.75 | 49.68 | 48.67 |  |  | 21.28 | 19.10 | 38.99 |  |  |
| 20.76 | 49.66 | 48.64 |  |  | 21.29 | 19.06 | 38.97 |  |  |
| 20.77 | 49.63 | 48.62 |  |  | 21.30 | 19.03 | 38.94 |  |  |
| 20.78 | 49.61 | 48.59 |  |  | 21.31 | 18.99 | 38.91 |  |  |
| 20.79 | 49.57 | 48.56 |  |  | 21.32 | 18.94 | 38.89 |  |  |
| 20.80 | 49.55 | 48.53 |  |  | 21.33 | 18.90 | 38.86 |  |  |
| 20.81 | 49.52 | 48.51 |  |  | 21.34 | 18.85 | 38.83 |  |  |
| 20.82 | 49.47 | 48.48 |  |  | 21.35 | 18.81 | 38.80 |  |  |
| 20.83 | 49.44 | 48.45 |  |  | 21.36 | 18.77 | 38.78 |  |  |
| 20.84 | 49.39 | 48.42 |  |  | 21.38 | 18.66 | 38.75 |  |  |
| 20.85 | 49.37 | 48.39 |  |  | 21.39 | 18.62 | 38.72 |  |  |
| 20.87 | 49.28 | 48.34 |  |  | 21.40 | 18.55 | 38.70 |  |  |
| 20.88 | 49.26 | 48.31 |  |  | 21.41 | 18.50 | 38.67 |  |  |
| 20.89 | 49.22 | 48.28 |  |  | 21.42 | 18.45 | 38.64 |  |  |
| 20.90 | 49.20 | 48.26 |  |  | 21.43 | 18.39 | 38.62 |  |  |
| 20.91 | 49.17 | 48.23 |  |  | 21.44 | 18.33 | 38.59 |  |  |
| 20.92 | 49.14 | 48.20 |  |  | 21.45 | 18.28 | 38.53 |  |  |
| 20.93 | 49.12 | 48.17 |  |  | 21.46 | 18.23 | 38.51 |  |  |
| 20.94 | 49.11 | 48.14 |  |  | 21.49 | 18.11 | 38.48 |  |  |
| 20.95 | 49.08 | 48.12 |  |  | 21.50 | 18.08 | 38.45 |  |  |
| 20.96 | 49.06 | 48.09 |  |  | 21.51 | 18.04 | 38.43 |  |  |
| 20.97 | 49.02 | 48.06 |  |  | 21.52 | 18.00 | 38.40 |  |  |
| 20.98 | 48.98 | 48.03 |  |  | 21.53 | 17.97 | 38.37 |  |  |
| 20.99 | 48.96 | 48.01 |  |  | 21.54 | 17.92 | 38.34 |  |  |
| 21.00 | 48.93 | 47.98 |  |  | 21.55 | 17.89 | 38.32 |  |  |
| 21.01 | 48.91 | 47.95 |  |  | 21.56 | 17.85 | 38.26 |  |  |
| 21.02 | 48.89 | 47.92 |  |  | 21.57 | 17.79 | 38.24 |  |  |
| 21.03 | 48.85 | 47.90 |  |  | 21.58 | 17.74 | 38.21 |  |  |
| 21.04 | 48.83 | 47.87 |  |  | 21.59 | 17.71 | 38.18 |  |  |
| 21.05 | 48.80 | 47.84 |  |  | 21.60 | 17.67 | 38.16 |  |  |
| 21.06 | 48.77 | 47.81 |  |  | 21.61 | 17.63 | 38.10 |  |  |
| 21.07 | 48.74 | 47.78 |  |  | 21.62 | 17.58 | 38.07 |  |  |
| 21.08 | 48.70 | 47.76 |  |  | 21.63 | 17.55 | 38.05 |  |  |
| 21.09 | 48.67 | 47.73 |  |  | 21.64 | 17.51 | 38.02 |  |  |
| 21.10 | 48.64 | 47.70 |  |  | 21.65 | 17.48 | 37.99 |  |  |
| 21.11 | 48.61 | 47.67 |  |  | 21.66 | 17.45 | 37.97 |  |  |
| 21.12 | 48.58 | 47.65 |  |  | 21.67 | 17.40 | 37.94 |  |  |
| 21.13 | 48.55 | 47.62 |  |  | 21.68 | 17.36 | 37.91 |  |  |
| 21.14 | 48.53 | 47.59 |  |  | 21.69 | 17.33 | 37.88 |  |  |
| 21.15 | 48.50 | 47.56 |  |  | 21.70 | 17.30 | 37.86 |  |  |
| 21.16 | 48.46 | 47.53 |  |  | 21.71 | 17.26 | 37.83 |  |  |
| 21.17 | 48.44 | 47.51 |  |  | 21.72 | 17.23 | 37.80 |  |  |
| 21.18 | 48.42 | 47.48 |  |  | 21.74 | 17.17 | 37.78 |  |  |
| 21.19 | 48.39 | 47.45 |  |  | 21.75 | 17.14 | 37.75 |  |  |
| 21.20 | 48.37 | 47.42 |  |  | 21.76 | 17.11 | 37.72 |  |  |
| 21.21 | 48.34 | 47.40 |  |  | 21.77 | 17.08 | 37.70 |  |  |
| 21.22 | 48.31 | 47.37 |  |  | 21.78 | 17.05 | 37.67 |  |  |
| 21.23 | 48.27 | 47.34 |  |  | 21.79 | 17.02 | 37.64 |  |  |
| 21.24 | 48.23 | 47.31 |  |  | 21.80 | 16.98 | 37.59 |  |  |
| 21.25 | 48.18 | 47.28 |  |  | 21.81 | 16.93 | 37.56 |  |  |
| 21.26 | 48.15 | 47.26 |  |  | 21.82 | 16.90 | 37.53 |  |  |
| 21.27 | 48.11 | 47.23 |  |  | 21.83 | 16.86 | 37.51 |  |  |
| 21.28 | 48.08 | 47.20 |  |  | 21.84 | 16.82 | 37.48 |  |  |
| 21.29 | 48.05 | 47.17 |  |  | 21.85 | 16.80 | 37.45 |  |  |
| 21.30 | 48.02 | 47.15 |  |  | 21.86 | 16.77 | 37.42 |  |  |
| 21.31 | 47.99 | 47.12 |  |  | 21.87 | 16.73 | 37.40 |  |  |
| 21.32 | 47.97 | 47.09 |  |  | 21.88 | 16.69 | 37.37 |  |  |
| 21.33 | 47.93 | 47.06 |  |  | 21.89 | 16.65 | 37.34 |  |  |
| 21.34 | 47.91 | 47.03 |  |  | 21.90 | 16.61 | 37.32 |  |  |
| 21.36 | 47.84 | 46.98 |  |  | 21.91 | 16.57 | 37.29 |  |  |
| 21.37 | 47.82 | 46.95 |  |  | 21.92 | 16.53 | 37.26 |  |  |
| 21.38 | 47.78 | 46.92 |  |  | 21.93 | 16.50 | 37.24 |  |  |
| 21.39 | 47.74 | 46.90 |  |  | 21.94 | 16.48 | 37.21 |  |  |
| 21.40 | 47.72 | 46.87 |  |  | 21.95 | 16.45 | 37.18 |  |  |
| 21.41 | 47.68 | 46.84 |  |  | 21.96 | 16.43 | 37.15 |  |  |
| 21.42 | 47.65 | 46.81 |  |  | 21.97 | 16.40 | 37.13 |  |  |
| 21.43 | 47.61 | 46.78 |  |  | 21.98 | 16.38 | 37.10 |  |  |
| 21.44 | 47.59 | 46.76 |  |  | 21.99 | 16.36 | 37.07 |  |  |
| 21.45 | 47.54 | 46.73 |  |  | 22.00 | 16.32 | 37.05 |  |  |
| 21.46 | 47.49 | 46.70 |  |  | 22.01 | 16.29 | 37.02 |  |  |
| 21.47 | 47.44 | 46.67 |  |  | 22.02 | 16.26 | 36.99 |  |  |
| 21.48 | 47.41 | 46.65 |  |  | 22.03 | 16.24 | 36.96 |  |  |
| 21.49 | 47.38 | 46.62 |  |  | 22.04 | 16.22 | 36.94 |  |  |
| 21.50 | 47.36 | 46.59 |  |  | 22.05 | 16.18 | 36.91 |  |  |
| 21.51 | 47.33 | 46.56 |  |  | 22.06 | 16.15 | 36.88 |  |  |
| 21.52 | 47.30 | 46.53 |  |  | 22.07 | 16.13 | 36.83 |  |  |
| 21.54 | 47.24 | 46.48 |  |  | 22.08 | 16.11 | 36.80 |  |  |
| 21.56 | 47.17 | 46.42 |  |  | 22.09 | 16.09 | 36.78 |  |  |
| 21.57 | 47.14 | 46.40 |  |  | 22.10 | 16.07 | 36.75 |  |  |
| 21.58 | 47.11 | 46.37 |  |  | 22.11 | 16.04 | 36.72 |  |  |
| 21.59 | 47.09 | 46.34 |  |  | 22.12 | 16.02 | 36.69 |  |  |
| 21.60 | 47.06 | 46.31 |  |  | 22.14 | 15.95 | 36.67 |  |  |
| 21.61 | 47.04 | 46.28 |  |  | 22.15 | 15.93 | 36.61 |  |  |
| 21.62 | 47.00 | 46.26 |  |  | 22.16 | 15.91 | 36.59 |  |  |
| 21.63 | 46.97 | 46.23 |  |  | 22.17 | 15.88 | 36.56 |  |  |
| 21.64 | 46.92 | 46.20 |  |  | 22.18 | 15.84 | 36.53 |  |  |
| 21.65 | 46.89 | 46.17 |  |  | 22.21 | 15.74 | 36.50 |  |  |
| 21.66 | 46.87 | 46.15 |  |  | 22.22 | 15.71 | 36.48 |  |  |
| 21.67 | 46.83 | 46.12 |  |  | 22.23 | 15.68 | 36.45 |  |  |
| 21.68 | 46.79 | 46.09 |  |  | 22.24 | 15.64 | 36.42 |  |  |
| 21.69 | 46.76 | 46.06 |  |  | 22.25 | 15.61 | 36.40 |  |  |
| 21.70 | 46.73 | 46.04 |  |  | 22.26 | 15.57 | 36.37 |  |  |
| 21.72 | 46.65 | 45.98 |  |  | 22.27 | 15.55 | 36.34 |  |  |
| 21.73 | 46.63 | 45.95 |  |  | 22.28 | 15.53 | 36.32 |  |  |
| 21.75 | 46.57 | 45.90 |  |  | 22.29 | 15.50 | 36.29 |  |  |
| 21.76 | 46.52 | 45.87 |  |  | 22.30 | 15.46 | 36.26 |  |  |
| 21.77 | 46.49 | 45.84 |  |  | 22.31 | 15.43 | 36.23 |  |  |
| 21.78 | 46.44 | 45.81 |  |  | 22.32 | 15.41 | 36.21 |  |  |
| 21.79 | 46.43 | 45.79 |  |  | 22.33 | 15.39 | 36.18 |  |  |
| 21.80 | 46.40 | 45.76 |  |  | 22.34 | 15.35 | 36.15 |  |  |
| 21.81 | 46.37 | 45.73 |  |  | 22.35 | 15.31 | 36.13 |  |  |
| 21.82 | 46.35 | 45.70 |  |  | 22.36 | 15.28 | 36.10 |  |  |
| 21.83 | 46.32 | 45.67 |  |  | 22.37 | 15.25 | 36.07 |  |  |
| 21.84 | 46.27 | 45.65 |  |  | 22.38 | 15.24 | 36.04 |  |  |
| 21.85 | 46.25 | 45.62 |  |  | 22.39 | 15.22 | 36.02 |  |  |
| 21.86 | 46.21 | 45.59 |  |  | 22.40 | 15.20 | 35.99 |  |  |
| 21.87 | 46.18 | 45.56 |  |  | 22.41 | 15.18 | 35.96 |  |  |
| 21.88 | 46.15 | 45.54 |  |  | 22.42 | 15.18 | 35.94 |  |  |
| 21.90 | 46.10 | 45.48 |  |  | 22.43 | 15.14 | 35.91 |  |  |
| 21.91 | 46.07 | 45.45 |  |  | 22.44 | 15.11 | 35.88 |  |  |
| 21.92 | 46.04 | 45.42 |  |  | 22.45 | 15.09 | 35.86 |  |  |
| 21.93 | 45.99 | 45.40 |  |  | 22.46 | 15.07 | 35.83 |  |  |
| 21.94 | 45.94 | 45.37 |  |  | 22.49 | 14.99 | 35.80 |  |  |
| 21.95 | 45.90 | 45.34 |  |  | 22.50 | 14.97 | 35.77 |  |  |
| 21.96 | 45.86 | 45.31 |  |  | 22.51 | 14.94 | 35.75 |  |  |
| 21.97 | 45.83 | 45.29 |  |  | 22.52 | 14.89 | 35.72 |  |  |
| 21.98 | 45.79 | 45.26 |  |  | 22.54 | 14.83 | 35.69 |  |  |
| 21.99 | 45.75 | 45.23 |  |  | 22.55 | 14.80 | 35.67 |  |  |
| 22.00 | 45.69 | 45.20 |  |  | 22.56 | 14.77 | 35.61 |  |  |
| 22.01 | 45.65 | 45.17 |  |  | 22.57 | 14.74 | 35.58 |  |  |
| 22.02 | 45.61 | 45.15 |  |  | 22.58 | 14.71 | 35.56 |  |  |
| 22.03 | 45.58 | 45.12 |  |  | 22.59 | 14.70 | 35.53 |  |  |
| 22.04 | 45.55 | 45.09 |  |  | 22.60 | 14.65 | 35.50 |  |  |
| 22.05 | 45.54 | 45.06 |  |  | 22.61 | 14.63 | 35.48 |  |  |
| 22.06 | 45.52 | 45.04 |  |  | 22.62 | 14.60 | 35.40 |  |  |
| 22.07 | 45.49 | 45.01 |  |  | 22.63 | 14.58 | 35.34 |  |  |
| 22.08 | 45.47 | 44.98 |  |  | 22.66 | 14.47 | 35.31 |  |  |
| 22.09 | 45.43 | 44.95 |  |  | 22.67 | 14.44 | 35.29 |  |  |
| 22.11 | 45.38 | 44.90 |  |  | 22.68 | 14.41 | 35.26 |  |  |
| 22.13 | 45.30 | 44.84 |  |  | 22.69 | 14.38 | 35.23 |  |  |
| 22.14 | 45.27 | 44.81 |  |  | 22.70 | 14.36 | 35.21 |  |  |
| 22.15 | 45.25 | 44.79 |  |  | 22.71 | 14.34 | 35.18 |  |  |
| 22.16 | 45.22 | 44.76 |  |  | 22.72 | 14.32 | 35.15 |  |  |
| 22.17 | 45.20 | 44.73 |  |  | 22.73 | 14.29 | 35.10 |  |  |
| 22.18 | 45.16 | 44.70 |  |  | 22.74 | 14.27 | 35.04 |  |  |
| 22.19 | 45.13 | 44.67 |  |  | 22.75 | 14.25 | 35.02 |  |  |
| 22.20 | 45.09 | 44.65 |  |  | 22.76 | 14.21 | 34.99 |  |  |
| 22.21 | 45.07 | 44.62 |  |  | 22.77 | 14.17 | 34.96 |  |  |
| 22.22 | 45.03 | 44.59 |  |  | 22.78 | 14.15 | 34.94 |  |  |
| 22.23 | 45.00 | 44.56 |  |  | 22.79 | 14.14 | 34.91 |  |  |
| 22.24 | 44.97 | 44.54 |  |  | 22.80 | 14.13 | 34.88 |  |  |
| 22.25 | 44.95 | 44.51 |  |  | 22.81 | 14.11 | 34.83 |  |  |
| 22.26 | 44.91 | 44.48 |  |  | 22.82 | 14.09 | 34.80 |  |  |
| 22.27 | 44.89 | 44.45 |  |  | 22.83 | 14.06 | 34.77 |  |  |
| 22.28 | 44.87 | 44.42 |  |  | 22.84 | 14.03 | 34.75 |  |  |
| 22.29 | 44.85 | 44.40 |  |  | 22.85 | 14.00 | 34.72 |  |  |
| 22.30 | 44.80 | 44.37 |  |  | 22.86 | 13.97 | 34.69 |  |  |
| 22.31 | 44.77 | 44.34 |  |  | 22.87 | 13.94 | 34.66 |  |  |
| 22.32 | 44.74 | 44.31 |  |  | 22.88 | 13.91 | 34.64 |  |  |
| 22.33 | 44.72 | 44.29 |  |  | 22.89 | 13.88 | 34.61 |  |  |
| 22.34 | 44.69 | 44.26 |  |  | 22.90 | 13.87 | 34.58 |  |  |
| 22.35 | 44.65 | 44.23 |  |  | 22.91 | 13.84 | 34.56 |  |  |
| 22.37 | 44.59 | 44.18 |  |  | 22.92 | 13.82 | 34.53 |  |  |
| 22.38 | 44.56 | 44.15 |  |  | 22.93 | 13.80 | 34.50 |  |  |
| 22.40 | 44.51 | 44.09 |  |  | 22.94 | 13.78 | 34.48 |  |  |
| 22.41 | 44.47 | 44.06 |  |  | 22.95 | 13.75 | 34.45 |  |  |
| 22.42 | 44.43 | 44.04 |  |  | 22.96 | 13.73 | 34.42 |  |  |
| 22.43 | 44.40 | 44.01 |  |  | 23.00 | 13.64 | 34.39 |  |  |
| 22.44 | 44.35 | 43.98 |  |  | 23.01 | 13.61 | 34.37 |  |  |
| 22.45 | 44.32 | 43.95 |  |  | 23.02 | 13.58 | 34.34 |  |  |
| 22.46 | 44.30 | 43.93 |  |  | 23.04 | 13.54 | 34.31 |  |  |
| 22.47 | 44.26 | 43.90 |  |  | 23.05 | 13.52 | 34.29 |  |  |
| 22.48 | 44.22 | 43.87 |  |  | 23.06 | 13.52 | 34.26 |  |  |
| 22.49 | 44.18 | 43.84 |  |  | 23.07 | 13.50 | 34.20 |  |  |
| 22.50 | 44.12 | 43.81 |  |  | 23.08 | 13.47 | 34.18 |  |  |
| 22.51 | 44.06 | 43.79 |  |  | 23.09 | 13.45 | 34.15 |  |  |
| 22.52 | 44.02 | 43.76 |  |  | 23.10 | 13.43 | 34.10 |  |  |
| 22.53 | 43.99 | 43.73 |  |  | 23.11 | 13.40 | 34.07 |  |  |
| 22.54 | 43.96 | 43.70 |  |  | 23.12 | 13.39 | 34.04 |  |  |
| 22.55 | 43.92 | 43.68 |  |  | 23.13 | 13.36 | 34.02 |  |  |
| 22.56 | 43.89 | 43.65 |  |  | 23.14 | 13.33 | 33.99 |  |  |
| 22.57 | 43.84 | 43.62 |  |  | 23.15 | 13.31 | 33.96 |  |  |
| 22.58 | 43.82 | 43.59 |  |  | 23.16 | 13.28 | 33.93 |  |  |
| 22.59 | 43.79 | 43.56 |  |  | 23.17 | 13.26 | 33.91 |  |  |
| 22.60 | 43.75 | 43.54 |  |  | 23.18 | 13.23 | 33.88 |  |  |
| 22.61 | 43.72 | 43.51 |  |  | 23.19 | 13.21 | 33.85 |  |  |
| 22.62 | 43.69 | 43.48 |  |  | 23.20 | 13.17 | 33.83 |  |  |
| 22.63 | 43.65 | 43.45 |  |  | 23.21 | 13.14 | 33.80 |  |  |
| 22.64 | 43.60 | 43.43 |  |  | 23.22 | 13.11 | 33.77 |  |  |
| 22.66 | 43.50 | 43.37 |  |  | 23.23 | 13.08 | 33.74 |  |  |
| 22.67 | 43.48 | 43.34 |  |  | 23.24 | 13.07 | 33.72 |  |  |
| 22.69 | 43.38 | 43.29 |  |  | 23.25 | 13.06 | 33.69 |  |  |
| 22.70 | 43.35 | 43.26 |  |  | 23.27 | 13.03 | 33.66 |  |  |
| 22.71 | 43.32 | 43.23 |  |  | 23.28 | 13.02 | 33.64 |  |  |
| 22.72 | 43.28 | 43.20 |  |  | 23.30 | 12.99 | 33.61 |  |  |
| 22.73 | 43.25 | 43.18 |  |  | 23.31 | 12.97 | 33.58 |  |  |
| 22.74 | 43.19 | 43.15 |  |  | 23.33 | 12.95 | 33.56 |  |  |
| 22.75 | 43.16 | 43.12 |  |  | 23.34 | 12.93 | 33.53 |  |  |
| 22.76 | 43.12 | 43.09 |  |  | 23.35 | 12.92 | 33.50 |  |  |
| 22.77 | 43.08 | 43.06 |  |  | 23.36 | 12.90 | 33.47 |  |  |
| 22.78 | 43.04 | 43.04 |  |  | 23.37 | 12.89 | 33.42 |  |  |
| 22.79 | 42.99 | 43.01 |  |  | 23.38 | 12.87 | 33.39 |  |  |
| 22.80 | 42.95 | 42.98 |  |  | 23.39 | 12.85 | 33.37 |  |  |
| 22.81 | 42.92 | 42.95 |  |  | 23.40 | 12.84 | 33.34 |  |  |
| 22.83 | 42.84 | 42.90 |  |  | 23.41 | 12.82 | 33.31 |  |  |
| 22.84 | 42.82 | 42.87 |  |  | 23.42 | 12.81 | 33.28 |  |  |
| 22.85 | 42.79 | 42.84 |  |  | 23.43 | 12.80 | 33.26 |  |  |
| 22.87 | 42.72 | 42.79 |  |  | 23.44 | 12.79 | 33.18 |  |  |
| 22.88 | 42.68 | 42.76 |  |  | 23.46 | 12.76 | 33.15 |  |  |
| 22.89 | 42.65 | 42.73 |  |  | 23.47 | 12.73 | 33.12 |  |  |
| 22.90 | 42.61 | 42.70 |  |  | 23.48 | 12.72 | 33.10 |  |  |
| 22.91 | 42.56 | 42.68 |  |  | 23.49 | 12.71 | 33.07 |  |  |
| 22.92 | 42.54 | 42.65 |  |  | 23.50 | 12.71 | 33.04 |  |  |
| 22.94 | 42.47 | 42.59 |  |  | 23.51 | 12.69 | 33.01 |  |  |
| 22.95 | 42.44 | 42.57 |  |  | 23.52 | 12.67 | 32.99 |  |  |
| 22.97 | 42.33 | 42.51 |  |  | 23.53 | 12.64 | 32.96 |  |  |
| 22.98 | 42.28 | 42.48 |  |  | 23.55 | 12.61 | 32.93 |  |  |
| 22.99 | 42.25 | 42.45 |  |  | 23.56 | 12.59 | 32.91 |  |  |
| 23.00 | 42.21 | 42.43 |  |  | 23.57 | 12.58 | 32.88 |  |  |
| 23.01 | 42.18 | 42.40 |  |  | 23.58 | 12.56 | 32.85 |  |  |
| 23.02 | 42.16 | 42.37 |  |  | 23.59 | 12.53 | 32.82 |  |  |
| 23.03 | 42.14 | 42.34 |  |  | 23.60 | 12.52 | 32.80 |  |  |
| 23.04 | 42.11 | 42.32 |  |  | 23.61 | 12.49 | 32.77 |  |  |
| 23.05 | 42.08 | 42.29 |  |  | 23.62 | 12.47 | 32.74 |  |  |
| 23.06 | 42.05 | 42.26 |  |  | 23.63 | 12.46 | 32.72 |  |  |
| 23.07 | 42.03 | 42.23 |  |  | 23.64 | 12.43 | 32.66 |  |  |
| 23.08 | 42.00 | 42.20 |  |  | 23.65 | 12.41 | 32.64 |  |  |
| 23.10 | 41.93 | 42.15 |  |  | 23.66 | 12.38 | 32.61 |  |  |
| 23.11 | 41.88 | 42.12 |  |  | 23.67 | 12.37 | 32.58 |  |  |
| 23.12 | 41.86 | 42.09 |  |  | 23.68 | 12.34 | 32.55 |  |  |
| 23.13 | 41.82 | 42.07 |  |  | 23.69 | 12.32 | 32.53 |  |  |
| 23.15 | 41.77 | 42.01 |  |  | 23.70 | 12.29 | 32.50 |  |  |
| 23.16 | 41.75 | 41.98 |  |  | 23.71 | 12.26 | 32.47 |  |  |
| 23.17 | 41.71 | 41.95 |  |  | 23.72 | 12.24 | 32.45 |  |  |
| 23.18 | 41.66 | 41.93 |  |  | 23.73 | 12.22 | 32.42 |  |  |
| 23.19 | 41.64 | 41.90 |  |  | 23.74 | 12.20 | 32.36 |  |  |
| 23.20 | 41.61 | 41.87 |  |  | 23.75 | 12.20 | 32.34 |  |  |
| 23.21 | 41.57 | 41.84 |  |  | 23.76 | 12.16 | 32.28 |  |  |
| 23.22 | 41.54 | 41.82 |  |  | 23.77 | 12.14 | 32.26 |  |  |
| 23.24 | 41.49 | 41.76 |  |  | 23.78 | 12.13 | 32.20 |  |  |
| 23.25 | 41.44 | 41.73 |  |  | 23.79 | 12.10 | 32.18 |  |  |
| 23.26 | 41.40 | 41.70 |  |  | 23.80 | 12.10 | 32.15 |  |  |
| 23.27 | 41.36 | 41.68 |  |  | 23.81 | 12.09 | 32.12 |  |  |
| 23.28 | 41.33 | 41.65 |  |  | 23.82 | 12.07 | 32.09 |  |  |
| 23.29 | 41.29 | 41.62 |  |  | 23.83 | 12.05 | 32.07 |  |  |
| 23.30 | 41.27 | 41.59 |  |  | 23.84 | 12.03 | 32.04 |  |  |
| 23.31 | 41.24 | 41.57 |  |  | 23.85 | 12.02 | 32.01 |  |  |
| 23.32 | 41.19 | 41.54 |  |  | 23.86 | 12.01 | 31.99 |  |  |
| 23.34 | 41.12 | 41.48 |  |  | 23.87 | 12.00 | 31.96 |  |  |
| 23.35 | 41.10 | 41.45 |  |  | 23.88 | 11.99 | 31.93 |  |  |
| 23.36 | 41.06 | 41.43 |  |  | 23.90 | 11.96 | 31.90 |  |  |
| 23.37 | 41.02 | 41.40 |  |  | 23.91 | 11.95 | 31.88 |  |  |
| 23.38 | 40.96 | 41.37 |  |  | 23.92 | 11.93 | 31.85 |  |  |
| 23.39 | 40.91 | 41.34 |  |  | 23.94 | 11.91 | 31.82 |  |  |
| 23.40 | 40.87 | 41.32 |  |  | 23.95 | 11.89 | 31.80 |  |  |
| 23.41 | 40.81 | 41.29 |  |  | 23.97 | 11.86 | 31.77 |  |  |
| 23.42 | 40.79 | 41.26 |  |  | 23.98 | 11.85 | 31.74 |  |  |
| 23.43 | 40.75 | 41.23 |  |  | 23.99 | 11.84 | 31.72 |  |  |
| 23.44 | 40.71 | 41.20 |  |  | 24.00 | 11.83 | 31.69 |  |  |
| 23.45 | 40.66 | 41.18 |  |  | 24.01 | 11.81 | 31.63 |  |  |
| 23.46 | 40.63 | 41.15 |  |  | 24.02 | 11.80 | 31.61 |  |  |
| 23.47 | 40.60 | 41.12 |  |  | 24.03 | 11.78 | 31.58 |  |  |
| 23.48 | 40.57 | 41.09 |  |  | 24.04 | 11.77 | 31.55 |  |  |
| 23.49 | 40.53 | 41.07 |  |  | 24.05 | 11.76 | 31.53 |  |  |
| 23.50 | 40.47 | 41.04 |  |  | 24.06 | 11.75 | 31.50 |  |  |
| 23.51 | 40.43 | 41.01 |  |  | 24.07 | 11.74 | 31.47 |  |  |
| 23.52 | 40.41 | 40.98 |  |  | 24.08 | 11.73 | 31.44 |  |  |
| 23.53 | 40.35 | 40.95 |  |  | 24.09 | 11.71 | 31.42 |  |  |
| 23.54 | 40.33 | 40.93 |  |  | 24.10 | 11.70 | 31.39 |  |  |
| 23.55 | 40.29 | 40.90 |  |  | 24.11 | 11.68 | 31.36 |  |  |
| 23.56 | 40.23 | 40.87 |  |  | 24.12 | 11.67 | 31.34 |  |  |
| 23.57 | 40.20 | 40.84 |  |  | 24.13 | 11.66 | 31.31 |  |  |
| 23.58 | 40.15 | 40.82 |  |  | 24.14 | 11.64 | 31.28 |  |  |
| 23.59 | 40.13 | 40.79 |  |  | 24.15 | 11.62 | 31.26 |  |  |
| 23.60 | 40.08 | 40.76 |  |  | 24.16 | 11.60 | 31.23 |  |  |
| 23.61 | 40.06 | 40.73 |  |  | 24.17 | 11.59 | 31.17 |  |  |
| 23.62 | 40.01 | 40.71 |  |  | 24.18 | 11.58 | 31.15 |  |  |
| 23.64 | 39.94 | 40.65 |  |  | 24.19 | 11.57 | 31.12 |  |  |
| 23.65 | 39.92 | 40.62 |  |  | 24.20 | 11.55 | 31.09 |  |  |
| 23.66 | 39.87 | 40.59 |  |  | 24.21 | 11.54 | 31.07 |  |  |
| 23.67 | 39.82 | 40.57 |  |  | 24.22 | 11.54 | 31.04 |  |  |
| 23.68 | 39.79 | 40.54 |  |  | 24.23 | 11.52 | 31.01 |  |  |
| 23.69 | 39.74 | 40.51 |  |  | 24.24 | 11.51 | 30.98 |  |  |
| 23.70 | 39.71 | 40.48 |  |  | 24.25 | 11.51 | 30.93 |  |  |
| 23.71 | 39.67 | 40.46 |  |  | 24.26 | 11.49 | 30.90 |  |  |
| 23.72 | 39.62 | 40.43 |  |  | 24.27 | 11.48 | 30.88 |  |  |
| 23.73 | 39.59 | 40.40 |  |  | 24.28 | 11.47 | 30.85 |  |  |
| 23.74 | 39.55 | 40.37 |  |  | 24.29 | 11.45 | 30.82 |  |  |
| 23.75 | 39.52 | 40.34 |  |  | 24.31 | 11.43 | 30.80 |  |  |
| 23.76 | 39.48 | 40.32 |  |  | 24.32 | 11.42 | 30.77 |  |  |
| 23.77 | 39.45 | 40.29 |  |  | 24.33 | 11.40 | 30.74 |  |  |
| 23.78 | 39.42 | 40.26 |  |  | 24.34 | 11.39 | 30.71 |  |  |
| 23.79 | 39.38 | 40.23 |  |  | 24.35 | 11.37 | 30.69 |  |  |
| 23.80 | 39.33 | 40.21 |  |  | 24.36 | 11.36 | 30.66 |  |  |
| 23.81 | 39.30 | 40.18 |  |  | 24.37 | 11.35 | 30.63 |  |  |
| 23.82 | 39.27 | 40.15 |  |  | 24.38 | 11.33 | 30.61 |  |  |
| 23.83 | 39.23 | 40.12 |  |  | 24.39 | 11.32 | 30.58 |  |  |
| 23.84 | 39.19 | 40.09 |  |  | 24.40 | 11.30 | 30.55 |  |  |
| 23.85 | 39.16 | 40.07 |  |  | 24.42 | 11.27 | 30.52 |  |  |
| 23.86 | 39.13 | 40.04 |  |  | 24.43 | 11.26 | 30.50 |  |  |
| 23.87 | 39.09 | 40.01 |  |  | 24.44 | 11.24 | 30.47 |  |  |
| 23.88 | 39.05 | 39.98 |  |  | 24.45 | 11.23 | 30.44 |  |  |
| 23.90 | 39.00 | 39.93 |  |  | 24.46 | 11.21 | 30.42 |  |  |
| 23.91 | 38.96 | 39.90 |  |  | 24.47 | 11.19 | 30.39 |  |  |
| 23.92 | 38.92 | 39.87 |  |  | 24.48 | 11.18 | 30.36 |  |  |
| 23.93 | 38.89 | 39.84 |  |  | 24.49 | 11.16 | 30.34 |  |  |
| 23.95 | 38.79 | 39.79 |  |  | 24.52 | 11.12 | 30.31 |  |  |
| 23.96 | 38.77 | 39.76 |  |  | 24.54 | 11.10 | 30.28 |  |  |
| 23.97 | 38.72 | 39.73 |  |  | 24.55 | 11.10 | 30.23 |  |  |
| 23.98 | 38.70 | 39.71 |  |  | 24.56 | 11.08 | 30.20 |  |  |
| 23.99 | 38.66 | 39.68 |  |  | 24.58 | 11.04 | 30.17 |  |  |
| 24.00 | 38.63 | 39.65 |  |  | 24.59 | 11.02 | 30.12 |  |  |
| 24.01 | 38.60 | 39.62 |  |  | 24.60 | 11.01 | 30.09 |  |  |
| 24.02 | 38.57 | 39.59 |  |  | 24.61 | 11.00 | 30.06 |  |  |
| 24.03 | 38.52 | 39.57 |  |  | 24.62 | 10.99 | 30.04 |  |  |
| 24.04 | 38.48 | 39.54 |  |  | 24.63 | 10.97 | 30.01 |  |  |
| 24.05 | 38.45 | 39.51 |  |  | 24.64 | 10.96 | 29.98 |  |  |
| 24.06 | 38.42 | 39.48 |  |  | 24.65 | 10.93 | 29.93 |  |  |
| 24.07 | 38.38 | 39.46 |  |  | 24.66 | 10.91 | 29.90 |  |  |
| 24.08 | 38.32 | 39.43 |  |  | 24.67 | 10.89 | 29.85 |  |  |
| 24.09 | 38.28 | 39.40 |  |  | 24.68 | 10.88 | 29.82 |  |  |
| 24.10 | 38.25 | 39.37 |  |  | 24.69 | 10.86 | 29.79 |  |  |
| 24.11 | 38.22 | 39.34 |  |  | 24.70 | 10.84 | 29.77 |  |  |
| 24.12 | 38.17 | 39.32 |  |  | 24.71 | 10.83 | 29.74 |  |  |
| 24.13 | 38.14 | 39.29 |  |  | 24.72 | 10.82 | 29.71 |  |  |
| 24.14 | 38.12 | 39.26 |  |  | 24.73 | 10.81 | 29.66 |  |  |
| 24.15 | 38.08 | 39.23 |  |  | 24.74 | 10.80 | 29.60 |  |  |
| 24.16 | 38.04 | 39.21 |  |  | 24.75 | 10.77 | 29.58 |  |  |
| 24.17 | 38.01 | 39.18 |  |  | 24.76 | 10.75 | 29.55 |  |  |
| 24.18 | 37.97 | 39.15 |  |  | 24.77 | 10.73 | 29.52 |  |  |
| 24.19 | 37.92 | 39.12 |  |  | 24.78 | 10.72 | 29.50 |  |  |
| 24.20 | 37.90 | 39.10 |  |  | 24.79 | 10.70 | 29.47 |  |  |
| 24.21 | 37.86 | 39.07 |  |  | 24.80 | 10.69 | 29.44 |  |  |
| 24.22 | 37.83 | 39.04 |  |  | 24.82 | 10.65 | 29.42 |  |  |
| 24.23 | 37.78 | 39.01 |  |  | 24.83 | 10.63 | 29.36 |  |  |
| 24.24 | 37.76 | 38.98 |  |  | 24.85 | 10.61 | 29.31 |  |  |
| 24.25 | 37.73 | 38.96 |  |  | 24.86 | 10.61 | 29.28 |  |  |
| 24.26 | 37.69 | 38.93 |  |  | 24.88 | 10.57 | 29.25 |  |  |
| 24.27 | 37.64 | 38.90 |  |  | 24.89 | 10.55 | 29.23 |  |  |
| 24.28 | 37.62 | 38.87 |  |  | 24.91 | 10.51 | 29.20 |  |  |
| 24.29 | 37.59 | 38.85 |  |  | 24.92 | 10.48 | 29.17 |  |  |
| 24.30 | 37.56 | 38.82 |  |  | 24.93 | 10.46 | 29.14 |  |  |
| 24.31 | 37.53 | 38.79 |  |  | 24.94 | 10.44 | 29.09 |  |  |
| 24.32 | 37.50 | 38.76 |  |  | 24.95 | 10.42 | 29.06 |  |  |
| 24.35 | 37.41 | 38.68 |  |  | 24.96 | 10.42 | 29.04 |  |  |
| 24.36 | 37.39 | 38.65 |  |  | 24.97 | 10.41 | 29.01 |  |  |
| 24.37 | 37.35 | 38.62 |  |  | 24.98 | 10.40 | 28.96 |  |  |
| 24.38 | 37.33 | 38.60 |  |  | 24.99 | 10.38 | 28.93 |  |  |
| 24.39 | 37.28 | 38.57 |  |  | 25.00 | 10.36 | 28.90 |  |  |
| 24.40 | 37.25 | 38.54 |  |  | 25.01 | 10.35 | 28.82 |  |  |
| 24.43 | 37.17 | 38.46 |  |  | 25.02 | 10.33 | 28.79 |  |  |
| 24.44 | 37.13 | 38.43 |  |  | 25.03 | 10.31 | 28.74 |  |  |
| 24.46 | 37.07 | 38.37 |  |  | 25.04 | 10.30 | 28.71 |  |  |
| 24.47 | 37.04 | 38.35 |  |  | 25.05 | 10.28 | 28.66 |  |  |
| 24.48 | 37.03 | 38.32 |  |  | 25.06 | 10.27 | 28.63 |  |  |
| 24.49 | 37.00 | 38.29 |  |  | 25.08 | 10.25 | 28.60 |  |  |
| 24.50 | 36.96 | 38.26 |  |  | 25.09 | 10.22 | 28.58 |  |  |
| 24.51 | 36.93 | 38.23 |  |  | 25.10 | 10.21 | 28.55 |  |  |
| 24.52 | 36.89 | 38.21 |  |  | 25.11 | 10.19 | 28.52 |  |  |
| 24.53 | 36.86 | 38.18 |  |  | 25.12 | 10.18 | 28.50 |  |  |
| 24.54 | 36.84 | 38.15 |  |  | 25.13 | 10.15 | 28.47 |  |  |
| 24.55 | 36.81 | 38.12 |  |  | 25.16 | 10.10 | 28.44 |  |  |
| 24.56 | 36.79 | 38.10 |  |  | 25.17 | 10.09 | 28.41 |  |  |
| 24.57 | 36.76 | 38.07 |  |  | 25.18 | 10.07 | 28.39 |  |  |
| 24.58 | 36.72 | 38.04 |  |  | 25.19 | 10.05 | 28.36 |  |  |
| 24.59 | 36.69 | 38.01 |  |  | 25.20 | 10.04 | 28.33 |  |  |
| 24.60 | 36.64 | 37.98 |  |  | 25.21 | 10.02 | 28.31 |  |  |
| 24.61 | 36.62 | 37.96 |  |  | 25.22 | 10.00 | 28.28 |  |  |
| 24.62 | 36.59 | 37.93 |  |  | 25.23 | 9.99 | 28.25 |  |  |
| 24.64 | 36.54 | 37.87 |  |  | 25.24 | 9.97 | 28.23 |  |  |
| 24.65 | 36.50 | 37.85 |  |  | 25.25 | 9.95 | 28.20 |  |  |
| 24.66 | 36.47 | 37.82 |  |  | 25.26 | 9.95 | 28.14 |  |  |
| 24.67 | 36.42 | 37.79 |  |  | 25.28 | 9.93 | 28.12 |  |  |
| 24.68 | 36.38 | 37.76 |  |  | 25.29 | 9.91 | 28.09 |  |  |
| 24.70 | 36.32 | 37.71 |  |  | 25.31 | 9.89 | 28.06 |  |  |
| 24.71 | 36.28 | 37.68 |  |  | 25.33 | 9.86 | 28.04 |  |  |
| 24.72 | 36.25 | 37.65 |  |  | 25.34 | 9.85 | 28.01 |  |  |
| 24.73 | 36.22 | 37.62 |  |  | 25.35 | 9.84 | 27.98 |  |  |
| 24.74 | 36.19 | 37.60 |  |  | 25.37 | 9.82 | 27.95 |  |  |
| 24.76 | 36.14 | 37.54 |  |  | 25.38 | 9.81 | 27.93 |  |  |
| 24.77 | 36.11 | 37.51 |  |  | 25.39 | 9.80 | 27.90 |  |  |
| 24.78 | 36.09 | 37.48 |  |  | 25.40 | 9.79 | 27.87 |  |  |
| 24.79 | 36.06 | 37.46 |  |  | 25.41 | 9.76 | 27.85 |  |  |
| 24.80 | 36.04 | 37.43 |  |  | 25.42 | 9.75 | 27.82 |  |  |
| 24.81 | 36.01 | 37.40 |  |  | 25.43 | 9.73 | 27.79 |  |  |
| 24.82 | 35.97 | 37.37 |  |  | 25.44 | 9.71 | 27.77 |  |  |
| 24.83 | 35.94 | 37.35 |  |  | 25.45 | 9.69 | 27.74 |  |  |
| 24.84 | 35.92 | 37.32 |  |  | 25.46 | 9.68 | 27.71 |  |  |
| 24.85 | 35.89 | 37.29 |  |  | 25.47 | 9.67 | 27.68 |  |  |
| 24.86 | 35.87 | 37.26 |  |  | 25.48 | 9.67 | 27.66 |  |  |
| 24.87 | 35.84 | 37.24 |  |  | 25.49 | 9.66 | 27.63 |  |  |
| 24.88 | 35.83 | 37.21 |  |  | 25.50 | 9.65 | 27.60 |  |  |
| 24.89 | 35.81 | 37.18 |  |  | 25.51 | 9.65 | 27.58 |  |  |
| 24.90 | 35.78 | 37.15 |  |  | 25.52 | 9.63 | 27.52 |  |  |
| 24.91 | 35.75 | 37.12 |  |  | 25.54 | 9.62 | 27.49 |  |  |
| 24.92 | 35.74 | 37.10 |  |  | 25.55 | 9.61 | 27.47 |  |  |
| 24.93 | 35.72 | 37.07 |  |  | 25.56 | 9.59 | 27.44 |  |  |
| 24.94 | 35.70 | 37.04 |  |  | 25.57 | 9.59 | 27.41 |  |  |
| 24.95 | 35.67 | 37.01 |  |  | 25.58 | 9.58 | 27.39 |  |  |
| 24.96 | 35.63 | 36.99 |  |  | 25.59 | 9.57 | 27.33 |  |  |
| 24.97 | 35.60 | 36.96 |  |  | 25.60 | 9.56 | 27.31 |  |  |
| 24.98 | 35.58 | 36.93 |  |  | 25.61 | 9.55 | 27.28 |  |  |
| 24.99 | 35.56 | 36.90 |  |  | 25.62 | 9.54 | 27.25 |  |  |
| 25.01 | 35.51 | 36.85 |  |  | 25.63 | 9.53 | 27.22 |  |  |
| 25.02 | 35.48 | 36.82 |  |  | 25.65 | 9.52 | 27.17 |  |  |
| 25.03 | 35.48 | 36.79 |  |  | 25.66 | 9.52 | 27.14 |  |  |
| 25.04 | 35.45 | 36.76 |  |  | 25.67 | 9.51 | 27.12 |  |  |
| 25.05 | 35.44 | 36.74 |  |  | 25.68 | 9.50 | 27.09 |  |  |
| 25.07 | 35.39 | 36.68 |  |  | 25.69 | 9.50 | 27.06 |  |  |
| 25.08 | 35.38 | 36.65 |  |  | 25.70 | 9.49 | 27.03 |  |  |
| 25.09 | 35.37 | 36.62 |  |  | 25.71 | 9.49 | 27.01 |  |  |
| 25.10 | 35.34 | 36.60 |  |  | 25.72 | 9.48 | 26.98 |  |  |
| 25.11 | 35.31 | 36.57 |  |  | 25.73 | 9.47 | 26.93 |  |  |
| 25.12 | 35.29 | 36.54 |  |  | 25.74 | 9.47 | 26.90 |  |  |
| 25.13 | 35.28 | 36.51 |  |  | 25.75 | 9.46 | 26.87 |  |  |
| 25.14 | 35.26 | 36.49 |  |  | 25.76 | 9.46 | 26.85 |  |  |
| 25.15 | 35.23 | 36.46 |  |  | 25.77 | 9.45 | 26.82 |  |  |
| 25.16 | 35.22 | 36.43 |  |  | 25.79 | 9.43 | 26.79 |  |  |
| 25.17 | 35.21 | 36.40 |  |  | 25.80 | 9.42 | 26.76 |  |  |
| 25.18 | 35.19 | 36.37 |  |  | 25.81 | 9.42 | 26.74 |  |  |
| 25.19 | 35.16 | 36.35 |  |  | 25.82 | 9.42 | 26.71 |  |  |
| 25.20 | 35.14 | 36.32 |  |  | 25.83 | 9.41 | 26.68 |  |  |
| 25.21 | 35.12 | 36.29 |  |  | 25.84 | 9.41 | 26.66 |  |  |
| 25.22 | 35.10 | 36.26 |  |  | 25.85 | 9.40 | 26.63 |  |  |
| 25.23 | 35.09 | 36.24 |  |  | 25.86 | 9.39 | 26.60 |  |  |
| 25.24 | 35.08 | 36.21 |  |  | 25.87 | 9.38 | 26.57 |  |  |
| 25.25 | 35.06 | 36.18 |  |  | 25.88 | 9.36 | 26.55 |  |  |
| 25.26 | 35.03 | 36.15 |  |  | 25.89 | 9.35 | 26.52 |  |  |
| 25.27 | 35.02 | 36.12 |  |  | 25.90 | 9.35 | 26.49 |  |  |
| 25.28 | 35.00 | 36.10 |  |  | 25.91 | 9.34 | 26.44 |  |  |
| 25.29 | 34.98 | 36.07 |  |  | 25.92 | 9.33 | 26.41 |  |  |
| 25.30 | 34.97 | 36.04 |  |  | 25.93 | 9.32 | 26.39 |  |  |
| 25.31 | 34.95 | 36.01 |  |  | 25.95 | 9.30 | 26.33 |  |  |
| 25.32 | 34.92 | 35.99 |  |  | 25.96 | 9.29 | 26.30 |  |  |
| 25.34 | 34.88 | 35.93 |  |  | 25.97 | 9.28 | 26.28 |  |  |
| 25.35 | 34.87 | 35.90 |  |  | 25.98 | 9.28 | 26.25 |  |  |
| 25.36 | 34.85 | 35.87 |  |  | 25.99 | 9.27 | 26.22 |  |  |
| 25.37 | 34.83 | 35.85 |  |  | 26.00 | 9.26 | 26.20 |  |  |
| 25.38 | 34.81 | 35.82 |  |  | 26.02 | 9.25 | 26.17 |  |  |
| 25.39 | 34.78 | 35.79 |  |  | 26.03 | 9.25 | 26.14 |  |  |
| 25.40 | 34.76 | 35.76 |  |  | 26.04 | 9.24 | 26.11 |  |  |
| 25.41 | 34.73 | 35.74 |  |  | 26.05 | 9.23 | 26.09 |  |  |
| 25.42 | 34.69 | 35.71 |  |  | 26.06 | 9.22 | 26.06 |  |  |
| 25.43 | 34.68 | 35.68 |  |  | 26.09 | 9.19 | 26.03 |  |  |
| 25.44 | 34.66 | 35.65 |  |  | 26.10 | 9.19 | 26.01 |  |  |
| 25.45 | 34.65 | 35.63 |  |  | 26.11 | 9.17 | 25.98 |  |  |
| 25.46 | 34.64 | 35.60 |  |  | 26.12 | 9.16 | 25.95 |  |  |
| 25.47 | 34.63 | 35.57 |  |  | 26.13 | 9.13 | 25.87 |  |  |
| 25.48 | 34.61 | 35.54 |  |  | 26.14 | 9.12 | 25.84 |  |  |
| 25.49 | 34.59 | 35.51 |  |  | 26.15 | 9.11 | 25.82 |  |  |
| 25.50 | 34.58 | 35.49 |  |  | 26.18 | 9.08 | 25.79 |  |  |
| 25.51 | 34.57 | 35.46 |  |  | 26.19 | 9.07 | 25.76 |  |  |
| 25.52 | 34.55 | 35.43 |  |  | 26.20 | 9.06 | 25.74 |  |  |
| 25.53 | 34.53 | 35.40 |  |  | 26.24 | 9.02 | 25.71 |  |  |
| 25.54 | 34.49 | 35.38 |  |  | 26.25 | 9.01 | 25.68 |  |  |
| 25.55 | 34.48 | 35.35 |  |  | 26.26 | 9.01 | 25.65 |  |  |
| 25.56 | 34.45 | 35.32 |  |  | 26.27 | 8.99 | 25.63 |  |  |
| 25.57 | 34.43 | 35.29 |  |  | 26.28 | 8.99 | 25.57 |  |  |
| 25.58 | 34.39 | 35.26 |  |  | 26.29 | 8.97 | 25.55 |  |  |
| 25.59 | 34.38 | 35.24 |  |  | 26.30 | 8.95 | 25.52 |  |  |
| 25.60 | 34.35 | 35.21 |  |  | 26.31 | 8.94 | 25.49 |  |  |
| 25.61 | 34.33 | 35.18 |  |  | 26.33 | 8.92 | 25.47 |  |  |
| 25.62 | 34.30 | 35.15 |  |  | 26.35 | 8.88 | 25.44 |  |  |
| 25.63 | 34.27 | 35.13 |  |  | 26.36 | 8.87 | 25.38 |  |  |
| 25.64 | 34.24 | 35.10 |  |  | 26.38 | 8.84 | 25.33 |  |  |
| 25.65 | 34.22 | 35.07 |  |  | 26.41 | 8.81 | 25.30 |  |  |
| 25.66 | 34.19 | 35.04 |  |  | 26.42 | 8.79 | 25.28 |  |  |
| 25.67 | 34.16 | 35.01 |  |  | 26.43 | 8.77 | 25.25 |  |  |
| 25.68 | 34.10 | 34.99 |  |  | 26.44 | 8.77 | 25.22 |  |  |
| 25.69 | 34.06 | 34.96 |  |  | 26.45 | 8.76 | 25.19 |  |  |
| 25.70 | 34.04 | 34.93 |  |  | 26.47 | 8.74 | 25.17 |  |  |
| 25.71 | 34.01 | 34.90 |  |  | 26.48 | 8.72 | 25.14 |  |  |
| 25.73 | 33.96 | 34.85 |  |  | 26.49 | 8.71 | 25.09 |  |  |
| 25.74 | 33.92 | 34.82 |  |  | 26.50 | 8.69 | 25.03 |  |  |
| 25.75 | 33.90 | 34.79 |  |  | 26.51 | 8.69 | 25.01 |  |  |
| 25.77 | 33.84 | 34.74 |  |  | 26.52 | 8.67 | 24.98 |  |  |
| 25.78 | 33.82 | 34.71 |  |  | 26.53 | 8.66 | 24.95 |  |  |
| 25.79 | 33.81 | 34.68 |  |  | 26.54 | 8.65 | 24.92 |  |  |
| 25.80 | 33.78 | 34.65 |  |  | 26.55 | 8.64 | 24.90 |  |  |
| 25.81 | 33.77 | 34.63 |  |  | 26.56 | 8.63 | 24.87 |  |  |
| 25.82 | 33.75 | 34.60 |  |  | 26.57 | 8.62 | 24.84 |  |  |
| 25.83 | 33.72 | 34.57 |  |  | 26.58 | 8.60 | 24.82 |  |  |
| 25.84 | 33.70 | 34.54 |  |  | 26.59 | 8.59 | 24.79 |  |  |
| 25.85 | 33.68 | 34.51 |  |  | 26.60 | 8.57 | 24.76 |  |  |
| 25.86 | 33.66 | 34.49 |  |  | 26.61 | 8.57 | 24.73 |  |  |
| 25.88 | 33.62 | 34.43 |  |  | 26.62 | 8.55 | 24.71 |  |  |
| 25.89 | 33.62 | 34.40 |  |  | 26.64 | 8.53 | 24.68 |  |  |
| 25.90 | 33.60 | 34.38 |  |  | 26.65 | 8.52 | 24.65 |  |  |
| 25.91 | 33.58 | 34.35 |  |  | 26.67 | 8.51 | 24.63 |  |  |
| 25.92 | 33.56 | 34.32 |  |  | 26.68 | 8.49 | 24.60 |  |  |
| 25.93 | 33.54 | 34.29 |  |  | 26.70 | 8.47 | 24.57 |  |  |
| 25.94 | 33.51 | 34.26 |  |  | 26.71 | 8.45 | 24.55 |  |  |
| 25.95 | 33.49 | 34.24 |  |  | 26.72 | 8.44 | 24.52 |  |  |
| 25.96 | 33.48 | 34.21 |  |  | 26.73 | 8.43 | 24.49 |  |  |
| 25.97 | 33.47 | 34.18 |  |  | 26.75 | 8.41 | 24.46 |  |  |
| 25.98 | 33.46 | 34.15 |  |  | 26.76 | 8.41 | 24.44 |  |  |
| 25.99 | 33.45 | 34.13 |  |  | 26.78 | 8.39 | 24.41 |  |  |
| 26.00 | 33.42 | 34.10 |  |  | 26.79 | 8.39 | 24.38 |  |  |
| 26.01 | 33.41 | 34.07 |  |  | 26.80 | 8.37 | 24.36 |  |  |
| 26.03 | 33.37 | 34.01 |  |  | 26.82 | 8.35 | 24.33 |  |  |
| 26.04 | 33.35 | 33.99 |  |  | 26.83 | 8.33 | 24.30 |  |  |
| 26.05 | 33.34 | 33.96 |  |  | 26.85 | 8.31 | 24.27 |  |  |
| 26.06 | 33.32 | 33.93 |  |  | 26.87 | 8.30 | 24.25 |  |  |
| 26.07 | 33.31 | 33.90 |  |  | 26.88 | 8.28 | 24.22 |  |  |
| 26.08 | 33.29 | 33.88 |  |  | 26.90 | 8.26 | 24.19 |  |  |
| 26.09 | 33.28 | 33.85 |  |  | 26.91 | 8.25 | 24.17 |  |  |
| 26.10 | 33.27 | 33.82 |  |  | 26.92 | 8.24 | 24.14 |  |  |
| 26.11 | 33.26 | 33.79 |  |  | 26.93 | 8.23 | 24.11 |  |  |
| 26.12 | 33.25 | 33.77 |  |  | 26.94 | 8.23 | 24.09 |  |  |
| 26.13 | 33.23 | 33.74 |  |  | 26.97 | 8.20 | 24.03 |  |  |
| 26.14 | 33.22 | 33.71 |  |  | 26.98 | 8.20 | 24.00 |  |  |
| 26.15 | 33.20 | 33.68 |  |  | 27.00 | 8.18 | 23.95 |  |  |
| 26.16 | 33.19 | 33.65 |  |  | 27.02 | 8.17 | 23.90 |  |  |
| 26.17 | 33.18 | 33.63 |  |  | 27.03 | 8.16 | 23.87 |  |  |
| 26.18 | 33.17 | 33.60 |  |  | 27.04 | 8.15 | 23.84 |  |  |
| 26.19 | 33.16 | 33.57 |  |  | 27.05 | 8.13 | 23.81 |  |  |
| 26.20 | 33.15 | 33.54 |  |  | 27.06 | 8.11 | 23.79 |  |  |
| 26.21 | 33.13 | 33.52 |  |  | 27.07 | 8.10 | 23.76 |  |  |
| 26.23 | 33.08 | 33.46 |  |  | 27.08 | 8.09 | 23.73 |  |  |
| 26.24 | 33.07 | 33.43 |  |  | 27.09 | 8.09 | 23.71 |  |  |
| 26.25 | 33.05 | 33.40 |  |  | 27.10 | 8.08 | 23.68 |  |  |
| 26.26 | 33.03 | 33.38 |  |  | 27.11 | 8.08 | 23.65 |  |  |
| 26.27 | 33.02 | 33.35 |  |  | 27.13 | 8.06 | 23.63 |  |  |
| 26.28 | 33.00 | 33.32 |  |  | 27.14 | 8.06 | 23.60 |  |  |
| 26.29 | 32.98 | 33.29 |  |  | 27.15 | 8.04 | 23.57 |  |  |
| 26.31 | 32.94 | 33.24 |  |  | 27.16 | 8.03 | 23.54 |  |  |
| 26.32 | 32.92 | 33.21 |  |  | 27.17 | 8.02 | 23.52 |  |  |
| 26.33 | 32.90 | 33.18 |  |  | 27.18 | 8.01 | 23.49 |  |  |
| 26.34 | 32.87 | 33.15 |  |  | 27.19 | 8.00 | 23.46 |  |  |
| 26.35 | 32.86 | 33.13 |  |  | 27.20 | 7.98 | 23.44 |  |  |
| 26.36 | 32.84 | 33.10 |  |  | 27.21 | 7.98 | 23.38 |  |  |
| 26.37 | 32.81 | 33.07 |  |  | 27.22 | 7.97 | 23.35 |  |  |
| 26.38 | 32.80 | 33.04 |  |  | 27.23 | 7.96 | 23.33 |  |  |
| 26.39 | 32.78 | 33.02 |  |  | 27.24 | 7.95 | 23.30 |  |  |
| 26.40 | 32.75 | 32.99 |  |  | 27.26 | 7.94 | 23.27 |  |  |
| 26.41 | 32.73 | 32.96 |  |  | 27.27 | 7.93 | 23.25 |  |  |
| 26.42 | 32.70 | 32.93 |  |  | 27.28 | 7.92 | 23.22 |  |  |
| 26.43 | 32.67 | 32.90 |  |  | 27.30 | 7.90 | 23.19 |  |  |
| 26.44 | 32.64 | 32.88 |  |  | 27.32 | 7.88 | 23.17 |  |  |
| 26.45 | 32.62 | 32.85 |  |  | 27.33 | 7.88 | 23.14 |  |  |
| 26.46 | 32.59 | 32.82 |  |  | 27.34 | 7.87 | 23.11 |  |  |
| 26.47 | 32.55 | 32.79 |  |  | 27.35 | 7.85 | 23.08 |  |  |
| 26.48 | 32.53 | 32.77 |  |  | 27.37 | 7.84 | 23.06 |  |  |
| 26.49 | 32.51 | 32.74 |  |  | 27.38 | 7.83 | 23.03 |  |  |
| 26.50 | 32.49 | 32.71 |  |  | 27.39 | 7.82 | 22.95 |  |  |
| 26.51 | 32.47 | 32.68 |  |  | 27.40 | 7.82 | 22.92 |  |  |
| 26.53 | 32.43 | 32.63 |  |  | 27.41 | 7.80 | 22.89 |  |  |
| 26.54 | 32.41 | 32.60 |  |  | 27.42 | 7.78 | 22.81 |  |  |
| 26.55 | 32.36 | 32.57 |  |  | 27.44 | 7.76 | 22.76 |  |  |
| 26.56 | 32.34 | 32.54 |  |  | 27.45 | 7.75 | 22.73 |  |  |
| 26.57 | 32.32 | 32.52 |  |  | 27.46 | 7.74 | 22.71 |  |  |
| 26.58 | 32.29 | 32.49 |  |  | 27.47 | 7.72 | 22.68 |  |  |
| 26.59 | 32.27 | 32.46 |  |  | 27.48 | 7.72 | 22.65 |  |  |
| 26.60 | 32.24 | 32.43 |  |  | 27.49 | 7.71 | 22.62 |  |  |
| 26.61 | 32.23 | 32.40 |  |  | 27.50 | 7.70 | 22.60 |  |  |
| 26.62 | 32.19 | 32.38 |  |  | 27.51 | 7.70 | 22.57 |  |  |
| 26.63 | 32.16 | 32.35 |  |  | 27.53 | 7.66 | 22.54 |  |  |
| 26.64 | 32.15 | 32.32 |  |  | 27.54 | 7.65 | 22.52 |  |  |
| 26.65 | 32.13 | 32.29 |  |  | 27.55 | 7.63 | 22.49 |  |  |
| 26.66 | 32.10 | 32.27 |  |  | 27.56 | 7.63 | 22.46 |  |  |
| 26.67 | 32.07 | 32.24 |  |  | 27.57 | 7.62 | 22.41 |  |  |
| 26.68 | 32.05 | 32.21 |  |  | 27.58 | 7.61 | 22.38 |  |  |
| 26.69 | 32.03 | 32.18 |  |  | 27.59 | 7.60 | 22.35 |  |  |
| 26.70 | 32.01 | 32.16 |  |  | 27.60 | 7.59 | 22.33 |  |  |
| 26.71 | 31.99 | 32.13 |  |  | 27.61 | 7.59 | 22.30 |  |  |
| 26.72 | 31.96 | 32.10 |  |  | 27.62 | 7.58 | 22.27 |  |  |
| 26.73 | 31.95 | 32.07 |  |  | 27.63 | 7.57 | 22.25 |  |  |
| 26.74 | 31.92 | 32.04 |  |  | 27.64 | 7.57 | 22.22 |  |  |
| 26.75 | 31.90 | 32.02 |  |  | 27.65 | 7.56 | 22.19 |  |  |
| 26.76 | 31.88 | 31.99 |  |  | 27.66 | 7.55 | 22.16 |  |  |
| 26.77 | 31.87 | 31.96 |  |  | 27.67 | 7.54 | 22.14 |  |  |
| 26.78 | 31.86 | 31.93 |  |  | 27.69 | 7.53 | 22.11 |  |  |
| 26.79 | 31.84 | 31.91 |  |  | 27.70 | 7.51 | 22.08 |  |  |
| 26.80 | 31.81 | 31.88 |  |  | 27.71 | 7.50 | 22.06 |  |  |
| 26.81 | 31.79 | 31.85 |  |  | 27.72 | 7.48 | 22.03 |  |  |
| 26.82 | 31.77 | 31.82 |  |  | 27.73 | 7.47 | 22.00 |  |  |
| 26.83 | 31.75 | 31.79 |  |  | 27.74 | 7.47 | 21.97 |  |  |
| 26.84 | 31.72 | 31.77 |  |  | 27.75 | 7.46 | 21.92 |  |  |
| 26.85 | 31.70 | 31.74 |  |  | 27.76 | 7.46 | 21.89 |  |  |
| 26.86 | 31.67 | 31.71 |  |  | 27.77 | 7.45 | 21.87 |  |  |
| 26.87 | 31.64 | 31.68 |  |  | 27.78 | 7.45 | 21.84 |  |  |
| 26.88 | 31.63 | 31.66 |  |  | 27.79 | 7.44 | 21.81 |  |  |
| 26.89 | 31.61 | 31.63 |  |  | 27.80 | 7.43 | 21.79 |  |  |
| 26.90 | 31.57 | 31.60 |  |  | 27.81 | 7.43 | 21.76 |  |  |
| 26.91 | 31.54 | 31.57 |  |  | 27.82 | 7.43 | 21.73 |  |  |
| 26.92 | 31.52 | 31.54 |  |  | 27.83 | 7.42 | 21.68 |  |  |
| 26.93 | 31.50 | 31.52 |  |  | 27.84 | 7.40 | 21.65 |  |  |
| 26.94 | 31.48 | 31.49 |  |  | 27.85 | 7.38 | 21.62 |  |  |
| 26.95 | 31.47 | 31.46 |  |  | 27.86 | 7.37 | 21.57 |  |  |
| 26.97 | 31.44 | 31.41 |  |  | 27.87 | 7.36 | 21.54 |  |  |
| 26.99 | 31.41 | 31.35 |  |  | 27.88 | 7.34 | 21.51 |  |  |
| 27.00 | 31.39 | 31.32 |  |  | 27.89 | 7.33 | 21.49 |  |  |
| 27.01 | 31.37 | 31.29 |  |  | 27.90 | 7.31 | 21.46 |  |  |
| 27.02 | 31.36 | 31.27 |  |  | 27.92 | 7.30 | 21.43 |  |  |
| 27.03 | 31.34 | 31.24 |  |  | 27.93 | 7.29 | 21.41 |  |  |
| 27.04 | 31.31 | 31.21 |  |  | 27.94 | 7.28 | 21.38 |  |  |
| 27.05 | 31.29 | 31.18 |  |  | 27.95 | 7.28 | 21.35 |  |  |
| 27.07 | 31.25 | 31.13 |  |  | 27.98 | 7.25 | 21.33 |  |  |
| 27.08 | 31.23 | 31.10 |  |  | 27.99 | 7.24 | 21.27 |  |  |
| 27.09 | 31.21 | 31.07 |  |  | 28.00 | 7.23 | 21.24 |  |  |
| 27.10 | 31.19 | 31.04 |  |  | 28.01 | 7.22 | 21.22 |  |  |
| 27.11 | 31.18 | 31.02 |  |  | 28.02 | 7.21 | 21.19 |  |  |
| 27.13 | 31.15 | 30.96 |  |  | 28.03 | 7.21 | 21.16 |  |  |
| 27.14 | 31.13 | 30.93 |  |  | 28.04 | 7.20 | 21.14 |  |  |
| 27.15 | 31.12 | 30.91 |  |  | 28.05 | 7.19 | 21.08 |  |  |
| 27.16 | 31.09 | 30.88 |  |  | 28.07 | 7.17 | 21.05 |  |  |
| 27.18 | 31.06 | 30.82 |  |  | 28.08 | 7.16 | 21.03 |  |  |
| 27.19 | 31.02 | 30.79 |  |  | 28.10 | 7.14 | 21.00 |  |  |
| 27.20 | 30.99 | 30.77 |  |  | 28.11 | 7.13 | 20.97 |  |  |
| 27.21 | 30.97 | 30.74 |  |  | 28.12 | 7.11 | 20.95 |  |  |
| 27.22 | 30.95 | 30.71 |  |  | 28.13 | 7.10 | 20.92 |  |  |
| 27.23 | 30.94 | 30.68 |  |  | 28.15 | 7.10 | 20.89 |  |  |
| 27.24 | 30.93 | 30.66 |  |  | 28.16 | 7.08 | 20.87 |  |  |
| 27.25 | 30.90 | 30.63 |  |  | 28.18 | 7.07 | 20.84 |  |  |
| 27.26 | 30.89 | 30.60 |  |  | 28.19 | 7.05 | 20.81 |  |  |
| 27.27 | 30.88 | 30.57 |  |  | 28.20 | 7.03 | 20.78 |  |  |
| 27.28 | 30.86 | 30.54 |  |  | 28.21 | 7.02 | 20.76 |  |  |
| 27.29 | 30.83 | 30.52 |  |  | 28.23 | 7.00 | 20.73 |  |  |
| 27.30 | 30.82 | 30.49 |  |  | 28.24 | 7.00 | 20.70 |  |  |
| 27.31 | 30.80 | 30.46 |  |  | 28.25 | 6.99 | 20.68 |  |  |
| 27.32 | 30.79 | 30.43 |  |  | 28.26 | 6.98 | 20.65 |  |  |
| 27.33 | 30.77 | 30.41 |  |  | 28.27 | 6.97 | 20.62 |  |  |
| 27.34 | 30.76 | 30.38 |  |  | 28.29 | 6.94 | 20.57 |  |  |
| 27.35 | 30.74 | 30.35 |  |  | 28.30 | 6.94 | 20.54 |  |  |
| 27.36 | 30.72 | 30.32 |  |  | 28.31 | 6.93 | 20.51 |  |  |
| 27.37 | 30.71 | 30.30 |  |  | 28.32 | 6.92 | 20.49 |  |  |
| 27.38 | 30.70 | 30.27 |  |  | 28.33 | 6.91 | 20.46 |  |  |
| 27.39 | 30.68 | 30.24 |  |  | 28.34 | 6.89 | 20.43 |  |  |
| 27.40 | 30.67 | 30.21 |  |  | 28.35 | 6.88 | 20.41 |  |  |
| 27.41 | 30.65 | 30.18 |  |  | 28.36 | 6.86 | 20.38 |  |  |
| 27.42 | 30.63 | 30.16 |  |  | 28.37 | 6.84 | 20.35 |  |  |
| 27.43 | 30.62 | 30.13 |  |  | 28.38 | 6.83 | 20.32 |  |  |
| 27.44 | 30.59 | 30.10 |  |  | 28.40 | 6.81 | 20.30 |  |  |
| 27.45 | 30.57 | 30.07 |  |  | 28.41 | 6.80 | 20.27 |  |  |
| 27.46 | 30.56 | 30.05 |  |  | 28.42 | 6.79 | 20.24 |  |  |
| 27.48 | 30.55 | 29.99 |  |  | 28.43 | 6.79 | 20.22 |  |  |
| 27.50 | 30.52 | 29.93 |  |  | 28.44 | 6.78 | 20.13 |  |  |
| 27.51 | 30.50 | 29.91 |  |  | 28.45 | 6.76 | 20.11 |  |  |
| 27.52 | 30.49 | 29.88 |  |  | 28.46 | 6.75 | 20.05 |  |  |
| 27.53 | 30.48 | 29.85 |  |  | 28.49 | 6.72 | 20.03 |  |  |
| 27.54 | 30.45 | 29.82 |  |  | 28.50 | 6.70 | 20.00 |  |  |
| 27.55 | 30.43 | 29.80 |  |  | 28.51 | 6.69 | 19.97 |  |  |
| 27.56 | 30.41 | 29.77 |  |  | 28.53 | 6.67 | 19.95 |  |  |
| 27.58 | 30.37 | 29.71 |  |  | 28.54 | 6.65 | 19.92 |  |  |
| 27.59 | 30.36 | 29.68 |  |  | 28.55 | 6.63 | 19.89 |  |  |
| 27.60 | 30.34 | 29.66 |  |  | 28.56 | 6.62 | 19.86 |  |  |
| 27.61 | 30.32 | 29.63 |  |  | 28.57 | 6.61 | 19.84 |  |  |
| 27.62 | 30.28 | 29.60 |  |  | 28.58 | 6.59 | 19.81 |  |  |
| 27.63 | 30.27 | 29.57 |  |  | 28.62 | 6.56 | 19.78 |  |  |
| 27.64 | 30.22 | 29.55 |  |  | 28.63 | 6.55 | 19.76 |  |  |
| 27.65 | 30.20 | 29.52 |  |  | 28.64 | 6.55 | 19.73 |  |  |
| 27.66 | 30.18 | 29.49 |  |  | 28.66 | 6.51 | 19.70 |  |  |
| 27.67 | 30.15 | 29.46 |  |  | 28.67 | 6.50 | 19.67 |  |  |
| 27.68 | 30.12 | 29.43 |  |  | 28.68 | 6.49 | 19.65 |  |  |
| 27.69 | 30.11 | 29.41 |  |  | 28.69 | 6.48 | 19.62 |  |  |
| 27.70 | 30.09 | 29.38 |  |  | 28.70 | 6.46 | 19.59 |  |  |
| 27.71 | 30.07 | 29.35 |  |  | 28.71 | 6.45 | 19.57 |  |  |
| 27.72 | 30.05 | 29.32 |  |  | 28.72 | 6.43 | 19.54 |  |  |
| 27.73 | 30.04 | 29.30 |  |  | 28.73 | 6.42 | 19.51 |  |  |
| 27.74 | 30.01 | 29.27 |  |  | 28.75 | 6.40 | 19.49 |  |  |
| 27.75 | 29.98 | 29.24 |  |  | 28.76 | 6.39 | 19.43 |  |  |
| 27.76 | 29.95 | 29.21 |  |  | 28.77 | 6.39 | 19.40 |  |  |
| 27.77 | 29.92 | 29.18 |  |  | 28.78 | 6.38 | 19.38 |  |  |
| 27.78 | 29.90 | 29.16 |  |  | 28.79 | 6.36 | 19.35 |  |  |
| 27.80 | 29.88 | 29.10 |  |  | 28.80 | 6.35 | 19.32 |  |  |
| 27.81 | 29.87 | 29.07 |  |  | 28.81 | 6.35 | 19.30 |  |  |
| 27.82 | 29.85 | 29.05 |  |  | 28.82 | 6.33 | 19.27 |  |  |
| 27.83 | 29.83 | 29.02 |  |  | 28.83 | 6.33 | 19.24 |  |  |
| 27.84 | 29.80 | 28.99 |  |  | 28.84 | 6.31 | 19.21 |  |  |
| 27.85 | 29.78 | 28.96 |  |  | 28.85 | 6.30 | 19.19 |  |  |
| 27.89 | 29.67 | 28.85 |  |  | 28.86 | 6.28 | 19.16 |  |  |
| 27.90 | 29.66 | 28.82 |  |  | 28.87 | 6.27 | 19.13 |  |  |
| 27.91 | 29.65 | 28.80 |  |  | 28.88 | 6.26 | 19.11 |  |  |
| 27.93 | 29.60 | 28.74 |  |  | 28.89 | 6.25 | 19.08 |  |  |
| 27.94 | 29.59 | 28.71 |  |  | 28.90 | 6.24 | 19.05 |  |  |
| 27.95 | 29.57 | 28.69 |  |  | 28.91 | 6.22 | 19.03 |  |  |
| 27.96 | 29.55 | 28.66 |  |  | 28.92 | 6.21 | 19.00 |  |  |
| 27.97 | 29.53 | 28.63 |  |  | 28.93 | 6.20 | 18.94 |  |  |
| 27.98 | 29.52 | 28.60 |  |  | 28.94 | 6.19 | 18.92 |  |  |
| 28.00 | 29.49 | 28.55 |  |  | 28.95 | 6.18 | 18.89 |  |  |
| 28.01 | 29.48 | 28.52 |  |  | 28.96 | 6.17 | 18.86 |  |  |
| 28.02 | 29.45 | 28.49 |  |  | 28.97 | 6.16 | 18.84 |  |  |
| 28.03 | 29.43 | 28.46 |  |  | 28.98 | 6.16 | 18.81 |  |  |
| 28.04 | 29.41 | 28.44 |  |  | 28.99 | 6.14 | 18.78 |  |  |
| 28.06 | 29.37 | 28.38 |  |  | 29.01 | 6.13 | 18.75 |  |  |
| 28.07 | 29.35 | 28.35 |  |  | 29.02 | 6.11 | 18.73 |  |  |
| 28.08 | 29.33 | 28.32 |  |  | 29.04 | 6.10 | 18.67 |  |  |
| 28.09 | 29.31 | 28.30 |  |  | 29.07 | 6.09 | 18.65 |  |  |
| 28.10 | 29.30 | 28.27 |  |  | 29.09 | 6.07 | 18.62 |  |  |
| 28.11 | 29.29 | 28.24 |  |  | 29.10 | 6.06 | 18.59 |  |  |
| 28.12 | 29.27 | 28.21 |  |  | 29.11 | 6.05 | 18.57 |  |  |
| 28.14 | 29.23 | 28.16 |  |  | 29.12 | 6.04 | 18.54 |  |  |
| 28.15 | 29.21 | 28.13 |  |  | 29.13 | 6.03 | 18.51 |  |  |
| 28.16 | 29.19 | 28.10 |  |  | 29.14 | 6.03 | 18.48 |  |  |
| 28.18 | 29.16 | 28.05 |  |  | 29.15 | 6.02 | 18.46 |  |  |
| 28.19 | 29.14 | 28.02 |  |  | 29.17 | 6.01 | 18.43 |  |  |
| 28.20 | 29.13 | 27.99 |  |  | 29.18 | 5.99 | 18.40 |  |  |
| 28.21 | 29.11 | 27.96 |  |  | 29.19 | 5.99 | 18.38 |  |  |
| 28.22 | 29.09 | 27.94 |  |  | 29.20 | 5.98 | 18.35 |  |  |
| 28.23 | 29.07 | 27.91 |  |  | 29.21 | 5.98 | 18.32 |  |  |
| 28.24 | 29.06 | 27.88 |  |  | 29.22 | 5.97 | 18.29 |  |  |
| 28.25 | 29.04 | 27.85 |  |  | 29.23 | 5.97 | 18.27 |  |  |
| 28.26 | 29.02 | 27.82 |  |  | 29.24 | 5.97 | 18.21 |  |  |
| 28.27 | 29.01 | 27.80 |  |  | 29.25 | 5.96 | 18.19 |  |  |
| 28.28 | 28.98 | 27.77 |  |  | 29.26 | 5.94 | 18.16 |  |  |
| 28.29 | 28.96 | 27.74 |  |  | 29.27 | 5.93 | 18.11 |  |  |
| 28.31 | 28.92 | 27.69 |  |  | 29.28 | 5.92 | 18.08 |  |  |
| 28.32 | 28.91 | 27.66 |  |  | 29.29 | 5.92 | 18.05 |  |  |
| 28.33 | 28.88 | 27.63 |  |  | 29.30 | 5.91 | 18.02 |  |  |
| 28.34 | 28.87 | 27.60 |  |  | 29.31 | 5.89 | 18.00 |  |  |
| 28.35 | 28.86 | 27.57 |  |  | 29.32 | 5.89 | 17.97 |  |  |
| 28.36 | 28.84 | 27.55 |  |  | 29.33 | 5.89 | 17.94 |  |  |
| 28.37 | 28.81 | 27.52 |  |  | 29.34 | 5.88 | 17.92 |  |  |
| 28.38 | 28.79 | 27.49 |  |  | 29.36 | 5.87 | 17.89 |  |  |
| 28.39 | 28.78 | 27.46 |  |  | 29.37 | 5.86 | 17.83 |  |  |
| 28.40 | 28.76 | 27.44 |  |  | 29.38 | 5.84 | 17.81 |  |  |
| 28.41 | 28.74 | 27.41 |  |  | 29.39 | 5.83 | 17.78 |  |  |
| 28.42 | 28.72 | 27.38 |  |  | 29.41 | 5.83 | 17.75 |  |  |
| 28.44 | 28.70 | 27.32 |  |  | 29.42 | 5.82 | 17.73 |  |  |
| 28.45 | 28.68 | 27.30 |  |  | 29.43 | 5.80 | 17.70 |  |  |
| 28.47 | 28.63 | 27.24 |  |  | 29.44 | 5.80 | 17.67 |  |  |
| 28.48 | 28.62 | 27.21 |  |  | 29.45 | 5.79 | 17.65 |  |  |
| 28.49 | 28.61 | 27.19 |  |  | 29.48 | 5.77 | 17.59 |  |  |
| 28.50 | 28.59 | 27.16 |  |  | 29.49 | 5.76 | 17.56 |  |  |
| 28.51 | 28.58 | 27.13 |  |  | 29.50 | 5.76 | 17.54 |  |  |
| 28.52 | 28.56 | 27.10 |  |  | 29.52 | 5.75 | 17.51 |  |  |
| 28.53 | 28.55 | 27.07 |  |  | 29.53 | 5.74 | 17.48 |  |  |
| 28.54 | 28.53 | 27.05 |  |  | 29.54 | 5.74 | 17.46 |  |  |
| 28.55 | 28.52 | 27.02 |  |  | 29.55 | 5.72 | 17.43 |  |  |
| 28.56 | 28.49 | 26.99 |  |  | 29.57 | 5.71 | 17.40 |  |  |
| 28.57 | 28.48 | 26.96 |  |  | 29.58 | 5.70 | 17.37 |  |  |
| 28.59 | 28.43 | 26.91 |  |  | 29.59 | 5.69 | 17.35 |  |  |
| 28.60 | 28.41 | 26.88 |  |  | 29.60 | 5.68 | 17.32 |  |  |
| 28.61 | 28.37 | 26.85 |  |  | 29.61 | 5.67 | 17.29 |  |  |
| 28.62 | 28.35 | 26.83 |  |  | 29.62 | 5.66 | 17.27 |  |  |
| 28.63 | 28.32 | 26.80 |  |  | 29.63 | 5.64 | 17.24 |  |  |
| 28.64 | 28.30 | 26.77 |  |  | 29.65 | 5.63 | 17.21 |  |  |
| 28.65 | 28.26 | 26.74 |  |  | 29.66 | 5.62 | 17.19 |  |  |
| 28.67 | 28.23 | 26.69 |  |  | 29.67 | 5.61 | 17.16 |  |  |
| 28.68 | 28.21 | 26.66 |  |  | 29.68 | 5.61 | 17.13 |  |  |
| 28.69 | 28.18 | 26.63 |  |  | 29.69 | 5.60 | 17.10 |  |  |
| 28.70 | 28.16 | 26.60 |  |  | 29.70 | 5.59 | 17.08 |  |  |
| 28.71 | 28.14 | 26.58 |  |  | 29.71 | 5.58 | 17.05 |  |  |
| 28.72 | 28.11 | 26.55 |  |  | 29.72 | 5.57 | 17.02 |  |  |
| 28.73 | 28.09 | 26.52 |  |  | 29.73 | 5.57 | 17.00 |  |  |
| 28.74 | 28.08 | 26.49 |  |  | 29.74 | 5.55 | 16.97 |  |  |
| 28.75 | 28.07 | 26.46 |  |  | 29.77 | 5.52 | 16.92 |  |  |
| 28.76 | 28.05 | 26.44 |  |  | 29.78 | 5.51 | 16.89 |  |  |
| 28.77 | 28.03 | 26.41 |  |  | 29.79 | 5.50 | 16.86 |  |  |
| 28.78 | 28.01 | 26.38 |  |  | 29.80 | 5.50 | 16.83 |  |  |
| 28.79 | 27.99 | 26.35 |  |  | 29.81 | 5.49 | 16.78 |  |  |
| 28.80 | 27.97 | 26.33 |  |  | 29.82 | 5.48 | 16.75 |  |  |
| 28.81 | 27.96 | 26.30 |  |  | 29.83 | 5.48 | 16.73 |  |  |
| 28.82 | 27.95 | 26.27 |  |  | 29.85 | 5.46 | 16.70 |  |  |
| 28.83 | 27.93 | 26.24 |  |  | 29.86 | 5.45 | 16.67 |  |  |
| 28.84 | 27.90 | 26.21 |  |  | 29.87 | 5.44 | 16.62 |  |  |
| 28.86 | 27.86 | 26.16 |  |  | 29.88 | 5.43 | 16.59 |  |  |
| 28.87 | 27.83 | 26.13 |  |  | 29.89 | 5.41 | 16.54 |  |  |
| 28.88 | 27.80 | 26.10 |  |  | 29.90 | 5.41 | 16.51 |  |  |
| 28.89 | 27.78 | 26.08 |  |  | 29.91 | 5.40 | 16.48 |  |  |
| 28.90 | 27.76 | 26.05 |  |  | 29.92 | 5.39 | 16.46 |  |  |
| 28.91 | 27.74 | 26.02 |  |  | 29.93 | 5.38 | 16.43 |  |  |
| 28.92 | 27.72 | 25.99 |  |  | 29.94 | 5.36 | 16.40 |  |  |
| 28.93 | 27.70 | 25.96 |  |  | 29.96 | 5.34 | 16.37 |  |  |
| 28.94 | 27.68 | 25.94 |  |  | 29.97 | 5.33 | 16.35 |  |  |
| 28.95 | 27.66 | 25.91 |  |  | 29.98 | 5.32 | 16.32 |  |  |
| 28.96 | 27.63 | 25.88 |  |  | 29.99 | 5.31 | 16.29 |  |  |
| 28.97 | 27.61 | 25.85 |  |  | 30.00 | 5.29 | 16.27 |  |  |
| 28.98 | 27.59 | 25.83 |  |  | 30.01 | 5.28 | 16.24 |  |  |
| 28.99 | 27.58 | 25.80 |  |  | 30.02 | 5.27 | 16.21 |  |  |
| 29.02 | 27.50 | 25.71 |  |  | 30.03 | 5.26 | 16.18 |  |  |
| 29.04 | 27.47 | 25.66 |  |  | 30.04 | 5.26 | 16.16 |  |  |
| 29.05 | 27.45 | 25.63 |  |  | 30.05 | 5.25 | 16.13 |  |  |
| 29.06 | 27.43 | 25.60 |  |  | 30.06 | 5.23 | 16.10 |  |  |
| 29.07 | 27.40 | 25.58 |  |  | 30.07 | 5.22 | 16.08 |  |  |
| 29.08 | 27.37 | 25.55 |  |  | 30.10 | 5.20 | 16.05 |  |  |
| 29.09 | 27.36 | 25.52 |  |  | 30.12 | 5.18 | 16.02 |  |  |
| 29.10 | 27.33 | 25.49 |  |  | 30.13 | 5.17 | 15.97 |  |  |
| 29.11 | 27.30 | 25.46 |  |  | 30.14 | 5.15 | 15.94 |  |  |
| 29.12 | 27.27 | 25.44 |  |  | 30.15 | 5.15 | 15.91 |  |  |
| 29.13 | 27.25 | 25.41 |  |  | 30.17 | 5.13 | 15.89 |  |  |
| 29.14 | 27.23 | 25.38 |  |  | 30.19 | 5.12 | 15.86 |  |  |
| 29.15 | 27.19 | 25.35 |  |  | 30.20 | 5.11 | 15.81 |  |  |
| 29.16 | 27.16 | 25.33 |  |  | 30.22 | 5.08 | 15.78 |  |  |
| 29.17 | 27.14 | 25.30 |  |  | 30.23 | 5.07 | 15.72 |  |  |
| 29.18 | 27.13 | 25.27 |  |  | 30.24 | 5.05 | 15.70 |  |  |
| 29.19 | 27.11 | 25.24 |  |  | 30.25 | 5.03 | 15.67 |  |  |
| 29.20 | 27.09 | 25.21 |  |  | 30.26 | 5.03 | 15.64 |  |  |
| 29.23 | 27.03 | 25.13 |  |  | 30.27 | 5.01 | 15.62 |  |  |
| 29.24 | 27.00 | 25.10 |  |  | 30.28 | 5.00 | 15.59 |  |  |
| 29.25 | 26.98 | 25.08 |  |  | 30.29 | 4.98 | 15.56 |  |  |
| 29.26 | 26.96 | 25.05 |  |  | 30.30 | 4.97 | 15.54 |  |  |
| 29.27 | 26.93 | 25.02 |  |  | 30.31 | 4.96 | 15.51 |  |  |
| 29.28 | 26.90 | 24.99 |  |  | 30.32 | 4.94 | 15.48 |  |  |
| 29.29 | 26.87 | 24.97 |  |  | 30.33 | 4.93 | 15.45 |  |  |
| 29.30 | 26.85 | 24.94 |  |  | 30.34 | 4.92 | 15.43 |  |  |
| 29.31 | 26.83 | 24.91 |  |  | 30.35 | 4.91 | 15.40 |  |  |
| 29.32 | 26.82 | 24.88 |  |  | 30.36 | 4.89 | 15.37 |  |  |
| 29.33 | 26.78 | 24.85 |  |  | 30.37 | 4.88 | 15.35 |  |  |
| 29.34 | 26.77 | 24.83 |  |  | 30.38 | 4.86 | 15.32 |  |  |
| 29.35 | 26.75 | 24.80 |  |  | 30.39 | 4.85 | 15.29 |  |  |
| 29.36 | 26.72 | 24.77 |  |  | 30.42 | 4.84 | 15.26 |  |  |
| 29.38 | 26.66 | 24.72 |  |  | 30.43 | 4.83 | 15.24 |  |  |
| 29.39 | 26.64 | 24.69 |  |  | 30.44 | 4.80 | 15.21 |  |  |
| 29.40 | 26.61 | 24.66 |  |  | 30.45 | 4.79 | 15.18 |  |  |
| 29.41 | 26.58 | 24.63 |  |  | 30.46 | 4.77 | 15.16 |  |  |
| 29.42 | 26.56 | 24.60 |  |  | 30.47 | 4.76 | 15.13 |  |  |
| 29.43 | 26.55 | 24.58 |  |  | 30.49 | 4.73 | 15.10 |  |  |
| 29.45 | 26.51 | 24.52 |  |  | 30.50 | 4.72 | 15.08 |  |  |
| 29.46 | 26.48 | 24.49 |  |  | 30.51 | 4.70 | 15.05 |  |  |
| 29.48 | 26.43 | 24.44 |  |  | 30.52 | 4.69 | 15.02 |  |  |
| 29.49 | 26.40 | 24.41 |  |  | 30.53 | 4.67 | 14.99 |  |  |
| 29.50 | 26.38 | 24.38 |  |  | 30.54 | 4.65 | 14.97 |  |  |
| 29.51 | 26.36 | 24.35 |  |  | 30.55 | 4.64 | 14.94 |  |  |
| 29.52 | 26.34 | 24.33 |  |  | 30.57 | 4.61 | 14.91 |  |  |
| 29.53 | 26.33 | 24.30 |  |  | 30.58 | 4.60 | 14.89 |  |  |
| 29.54 | 26.31 | 24.27 |  |  | 30.59 | 4.59 | 14.86 |  |  |
| 29.55 | 26.28 | 24.24 |  |  | 30.61 | 4.58 | 14.83 |  |  |
| 29.57 | 26.25 | 24.19 |  |  | 30.62 | 4.57 | 14.78 |  |  |
| 29.58 | 26.23 | 24.16 |  |  | 30.63 | 4.56 | 14.75 |  |  |
| 29.59 | 26.20 | 24.13 |  |  | 30.64 | 4.55 | 14.72 |  |  |
| 29.60 | 26.19 | 24.10 |  |  | 30.65 | 4.53 | 14.70 |  |  |
| 29.61 | 26.17 | 24.08 |  |  | 30.66 | 4.52 | 14.67 |  |  |
| 29.62 | 26.14 | 24.05 |  |  | 30.67 | 4.51 | 14.64 |  |  |
| 29.63 | 26.12 | 24.02 |  |  | 30.68 | 4.48 | 14.62 |  |  |
| 29.64 | 26.11 | 23.99 |  |  | 30.69 | 4.47 | 14.59 |  |  |
| 29.65 | 26.08 | 23.97 |  |  | 30.71 | 4.44 | 14.56 |  |  |
| 29.67 | 26.03 | 23.91 |  |  | 30.72 | 4.44 | 14.53 |  |  |
| 29.68 | 26.00 | 23.88 |  |  | 30.73 | 4.43 | 14.51 |  |  |
| 29.69 | 25.99 | 23.85 |  |  | 30.74 | 4.42 | 14.48 |  |  |
| 29.70 | 25.96 | 23.83 |  |  | 30.75 | 4.41 | 14.45 |  |  |
| 29.71 | 25.93 | 23.80 |  |  | 30.76 | 4.40 | 14.43 |  |  |
| 29.72 | 25.92 | 23.77 |  |  | 30.77 | 4.40 | 14.40 |  |  |
| 29.73 | 25.89 | 23.74 |  |  | 30.79 | 4.36 | 14.37 |  |  |
| 29.74 | 25.87 | 23.72 |  |  | 30.81 | 4.33 | 14.34 |  |  |
| 29.75 | 25.84 | 23.69 |  |  | 30.82 | 4.31 | 14.32 |  |  |
| 29.77 | 25.80 | 23.63 |  |  | 30.83 | 4.29 | 14.29 |  |  |
| 29.78 | 25.77 | 23.60 |  |  | 30.84 | 4.28 | 14.26 |  |  |
| 29.80 | 25.73 | 23.55 |  |  | 30.86 | 4.25 | 14.24 |  |  |
| 29.82 | 25.67 | 23.49 |  |  | 30.87 | 4.24 | 14.21 |  |  |
| 29.83 | 25.64 | 23.47 |  |  | 30.88 | 4.23 | 14.18 |  |  |
| 29.85 | 25.62 | 23.41 |  |  | 30.89 | 4.21 | 14.16 |  |  |
| 29.86 | 25.58 | 23.38 |  |  | 30.90 | 4.21 | 14.13 |  |  |
| 29.88 | 25.53 | 23.33 |  |  | 30.91 | 4.20 | 14.10 |  |  |
| 29.89 | 25.50 | 23.30 |  |  | 30.92 | 4.19 | 14.07 |  |  |
| 29.92 | 25.41 | 23.22 |  |  | 30.93 | 4.19 | 14.05 |  |  |
| 29.93 | 25.39 | 23.19 |  |  | 30.94 | 4.16 | 14.02 |  |  |
| 29.94 | 25.38 | 23.16 |  |  | 30.95 | 4.14 | 13.99 |  |  |
| 29.95 | 25.34 | 23.13 |  |  | 30.96 | 4.13 | 13.97 |  |  |
| 29.96 | 25.30 | 23.11 |  |  | 30.98 | 4.11 | 13.94 |  |  |
| 29.97 | 25.28 | 23.08 |  |  | 30.99 | 4.10 | 13.91 |  |  |
| 29.98 | 25.24 | 23.05 |  |  | 31.03 | 4.05 | 13.88 |  |  |
| 29.99 | 25.20 | 23.02 |  |  | 31.04 | 4.04 | 13.86 |  |  |
| 30.00 | 25.18 | 22.99 |  |  | 31.05 | 4.02 | 13.83 |  |  |
| 30.01 | 25.17 | 22.97 |  |  | 31.06 | 4.00 | 13.78 |  |  |
| 30.02 | 25.14 | 22.94 |  |  | 31.07 | 3.98 | 13.75 |  |  |
| 30.03 | 25.10 | 22.91 |  |  | 31.08 | 3.97 | 13.72 |  |  |
| 30.04 | 25.08 | 22.88 |  |  | 31.10 | 3.95 | 13.70 |  |  |
| 30.06 | 25.02 | 22.83 |  |  | 31.11 | 3.94 | 13.67 |  |  |
| 30.07 | 24.99 | 22.80 |  |  | 31.12 | 3.93 | 13.64 |  |  |
| 30.08 | 24.97 | 22.77 |  |  | 31.13 | 3.92 | 13.59 |  |  |
| 30.09 | 24.94 | 22.74 |  |  | 31.14 | 3.91 | 13.56 |  |  |
| 30.10 | 24.90 | 22.72 |  |  | 31.15 | 3.91 | 13.53 |  |  |
| 30.11 | 24.87 | 22.69 |  |  | 31.16 | 3.89 | 13.51 |  |  |
| 30.12 | 24.83 | 22.66 |  |  | 31.17 | 3.87 | 13.48 |  |  |
| 30.13 | 24.81 | 22.63 |  |  | 31.18 | 3.86 | 13.45 |  |  |
| 30.14 | 24.78 | 22.61 |  |  | 31.19 | 3.85 | 13.42 |  |  |
| 30.15 | 24.74 | 22.58 |  |  | 31.20 | 3.84 | 13.40 |  |  |
| 30.16 | 24.70 | 22.55 |  |  | 31.21 | 3.83 | 13.37 |  |  |
| 30.17 | 24.68 | 22.52 |  |  | 31.23 | 3.81 | 13.34 |  |  |
| 30.18 | 24.66 | 22.49 |  |  | 31.24 | 3.80 | 13.32 |  |  |
| 30.19 | 24.64 | 22.47 |  |  | 31.25 | 3.80 | 13.29 |  |  |
| 30.20 | 24.62 | 22.44 |  |  | 31.26 | 3.78 | 13.26 |  |  |
| 30.21 | 24.61 | 22.41 |  |  | 31.27 | 3.78 | 13.21 |  |  |
| 30.22 | 24.58 | 22.38 |  |  | 31.28 | 3.77 | 13.18 |  |  |
| 30.24 | 24.54 | 22.33 |  |  | 31.29 | 3.75 | 13.15 |  |  |
| 30.25 | 24.51 | 22.30 |  |  | 31.30 | 3.75 | 13.13 |  |  |
| 30.26 | 24.48 | 22.27 |  |  | 31.32 | 3.72 | 13.10 |  |  |
| 30.27 | 24.46 | 22.24 |  |  | 31.33 | 3.71 | 13.07 |  |  |
| 30.28 | 24.43 | 22.22 |  |  | 31.34 | 3.70 | 13.02 |  |  |
| 30.29 | 24.41 | 22.19 |  |  | 31.35 | 3.70 | 12.99 |  |  |
| 30.30 | 24.40 | 22.16 |  |  | 31.38 | 3.67 | 12.96 |  |  |
| 30.31 | 24.37 | 22.13 |  |  | 31.39 | 3.65 | 12.94 |  |  |
| 30.32 | 24.35 | 22.11 |  |  | 31.40 | 3.65 | 12.91 |  |  |
| 30.33 | 24.33 | 22.08 |  |  | 31.41 | 3.65 | 12.88 |  |  |
| 30.34 | 24.31 | 22.05 |  |  | 31.42 | 3.63 | 12.86 |  |  |
| 30.35 | 24.29 | 22.02 |  |  | 31.43 | 3.63 | 12.83 |  |  |
| 30.36 | 24.26 | 21.99 |  |  | 31.45 | 3.61 | 12.80 |  |  |
| 30.37 | 24.22 | 21.97 |  |  | 31.46 | 3.59 | 12.78 |  |  |
| 30.38 | 24.18 | 21.94 |  |  | 31.47 | 3.59 | 12.75 |  |  |
| 30.39 | 24.16 | 21.91 |  |  | 31.48 | 3.57 | 12.72 |  |  |
| 30.40 | 24.12 | 21.88 |  |  | 31.50 | 3.55 | 12.69 |  |  |
| 30.41 | 24.10 | 21.86 |  |  | 31.51 | 3.54 | 12.67 |  |  |
| 30.43 | 24.05 | 21.80 |  |  | 31.52 | 3.54 | 12.64 |  |  |
| 30.44 | 24.03 | 21.77 |  |  | 31.53 | 3.54 | 12.61 |  |  |
| 30.46 | 23.96 | 21.72 |  |  | 31.54 | 3.52 | 12.59 |  |  |
| 30.47 | 23.94 | 21.69 |  |  | 31.56 | 3.51 | 12.56 |  |  |
| 30.48 | 23.91 | 21.66 |  |  | 31.57 | 3.49 | 12.53 |  |  |
| 30.49 | 23.88 | 21.63 |  |  | 31.58 | 3.48 | 12.50 |  |  |
| 30.50 | 23.85 | 21.61 |  |  | 31.59 | 3.47 | 12.48 |  |  |
| 30.51 | 23.82 | 21.58 |  |  | 31.60 | 3.45 | 12.45 |  |  |
| 30.52 | 23.80 | 21.55 |  |  | 31.61 | 3.45 | 12.42 |  |  |
| 30.53 | 23.75 | 21.52 |  |  | 31.63 | 3.42 | 12.40 |  |  |
| 30.54 | 23.73 | 21.50 |  |  | 31.66 | 3.41 | 12.37 |  |  |
| 30.55 | 23.69 | 21.47 |  |  | 31.67 | 3.40 | 12.34 |  |  |
| 30.56 | 23.66 | 21.44 |  |  | 31.68 | 3.39 | 12.32 |  |  |
| 30.57 | 23.63 | 21.41 |  |  | 31.69 | 3.38 | 12.29 |  |  |
| 30.59 | 23.56 | 21.36 |  |  | 31.70 | 3.38 | 12.26 |  |  |
| 30.60 | 23.52 | 21.33 |  |  | 31.72 | 3.35 | 12.23 |  |  |
| 30.61 | 23.50 | 21.30 |  |  | 31.73 | 3.34 | 12.21 |  |  |
| 30.62 | 23.46 | 21.27 |  |  | 31.74 | 3.33 | 12.18 |  |  |
| 30.63 | 23.44 | 21.25 |  |  | 31.75 | 3.32 | 12.15 |  |  |
| 30.64 | 23.41 | 21.22 |  |  | 31.76 | 3.31 | 12.13 |  |  |
| 30.65 | 23.38 | 21.19 |  |  | 31.77 | 3.31 | 12.07 |  |  |
| 30.66 | 23.34 | 21.16 |  |  | 31.78 | 3.30 | 12.04 |  |  |
| 30.67 | 23.31 | 21.13 |  |  | 31.79 | 3.30 | 12.02 |  |  |
| 30.68 | 23.28 | 21.11 |  |  | 31.80 | 3.28 | 11.96 |  |  |
| 30.70 | 23.24 | 21.05 |  |  | 31.81 | 3.27 | 11.94 |  |  |
| 30.71 | 23.21 | 21.02 |  |  | 31.82 | 3.26 | 11.91 |  |  |
| 30.72 | 23.19 | 21.00 |  |  | 31.83 | 3.25 | 11.88 |  |  |
| 30.73 | 23.15 | 20.97 |  |  | 31.84 | 3.24 | 11.86 |  |  |
| 30.74 | 23.12 | 20.94 |  |  | 31.85 | 3.23 | 11.83 |  |  |
| 30.75 | 23.09 | 20.91 |  |  | 31.86 | 3.23 | 11.80 |  |  |
| 30.76 | 23.07 | 20.88 |  |  | 31.87 | 3.22 | 11.77 |  |  |
| 30.77 | 23.04 | 20.86 |  |  | 31.88 | 3.21 | 11.75 |  |  |
| 30.78 | 23.00 | 20.83 |  |  | 31.90 | 3.20 | 11.69 |  |  |
| 30.79 | 22.97 | 20.80 |  |  | 31.91 | 3.19 | 11.67 |  |  |
| 30.80 | 22.94 | 20.77 |  |  | 31.92 | 3.18 | 11.64 |  |  |
| 30.81 | 22.90 | 20.75 |  |  | 31.93 | 3.17 | 11.58 |  |  |
| 30.82 | 22.88 | 20.72 |  |  | 31.94 | 3.16 | 11.56 |  |  |
| 30.85 | 22.82 | 20.63 |  |  | 31.95 | 3.15 | 11.53 |  |  |
| 30.86 | 22.80 | 20.61 |  |  | 31.96 | 3.14 | 11.50 |  |  |
| 30.88 | 22.74 | 20.55 |  |  | 31.97 | 3.13 | 11.48 |  |  |
| 30.89 | 22.71 | 20.52 |  |  | 31.99 | 3.09 | 11.45 |  |  |
| 30.90 | 22.69 | 20.50 |  |  | 32.00 | 3.09 | 11.42 |  |  |
| 30.91 | 22.66 | 20.47 |  |  | 32.01 | 3.08 | 11.40 |  |  |
| 30.92 | 22.63 | 20.44 |  |  | 32.03 | 3.06 | 11.37 |  |  |
| 30.93 | 22.61 | 20.41 |  |  | 32.04 | 3.05 | 11.34 |  |  |
| 30.94 | 22.59 | 20.38 |  |  | 32.05 | 3.04 | 11.31 |  |  |
| 30.95 | 22.56 | 20.36 |  |  | 32.07 | 3.02 | 11.29 |  |  |
| 30.96 | 22.51 | 20.33 |  |  | 32.08 | 3.02 | 11.26 |  |  |
| 30.97 | 22.49 | 20.30 |  |  | 32.09 | 3.02 | 11.23 |  |  |
| 30.98 | 22.47 | 20.27 |  |  | 32.10 | 3.00 | 11.21 |  |  |
| 30.99 | 22.44 | 20.25 |  |  | 32.11 | 2.99 | 11.18 |  |  |
| 31.00 | 22.40 | 20.22 |  |  | 32.12 | 2.97 | 11.15 |  |  |
| 31.01 | 22.37 | 20.19 |  |  | 32.13 | 2.97 | 11.12 |  |  |
| 31.02 | 22.35 | 20.16 |  |  | 32.14 | 2.96 | 11.10 |  |  |
| 31.03 | 22.33 | 20.13 |  |  | 32.16 | 2.94 | 11.07 |  |  |
| 31.04 | 22.30 | 20.11 |  |  | 32.17 | 2.93 | 11.04 |  |  |
| 31.05 | 22.27 | 20.08 |  |  | 32.18 | 2.92 | 11.02 |  |  |
| 31.08 | 22.20 | 20.00 |  |  | 32.19 | 2.92 | 10.99 |  |  |
| 31.09 | 22.17 | 19.97 |  |  | 32.20 | 2.91 | 10.96 |  |  |
| 31.10 | 22.14 | 19.94 |  |  | 32.21 | 2.90 | 10.94 |  |  |
| 31.11 | 22.13 | 19.91 |  |  | 32.22 | 2.90 | 10.91 |  |  |
| 31.12 | 22.10 | 19.89 |  |  | 32.23 | 2.89 | 10.85 |  |  |
| 31.13 | 22.06 | 19.86 |  |  | 32.26 | 2.84 | 10.83 |  |  |
| 31.15 | 21.99 | 19.80 |  |  | 32.27 | 2.82 | 10.80 |  |  |
| 31.17 | 21.94 | 19.75 |  |  | 32.28 | 2.81 | 10.77 |  |  |
| 31.18 | 21.91 | 19.72 |  |  | 32.29 | 2.80 | 10.75 |  |  |
| 31.19 | 21.87 | 19.69 |  |  | 32.30 | 2.78 | 10.72 |  |  |
| 31.20 | 21.83 | 19.66 |  |  | 32.31 | 2.77 | 10.69 |  |  |
| 31.21 | 21.81 | 19.64 |  |  | 32.32 | 2.76 | 10.66 |  |  |
| 31.22 | 21.78 | 19.61 |  |  | 32.33 | 2.74 | 10.64 |  |  |
| 31.23 | 21.75 | 19.58 |  |  | 32.34 | 2.74 | 10.61 |  |  |
| 31.25 | 21.70 | 19.52 |  |  | 32.35 | 2.73 | 10.58 |  |  |
| 31.26 | 21.66 | 19.50 |  |  | 32.36 | 2.72 | 10.53 |  |  |
| 31.27 | 21.62 | 19.47 |  |  | 32.37 | 2.71 | 10.50 |  |  |
| 31.28 | 21.61 | 19.44 |  |  | 32.38 | 2.70 | 10.45 |  |  |
| 31.29 | 21.57 | 19.41 |  |  | 32.39 | 2.70 | 10.42 |  |  |
| 31.30 | 21.56 | 19.39 |  |  | 32.40 | 2.69 | 10.39 |  |  |
| 31.31 | 21.52 | 19.36 |  |  | 32.41 | 2.68 | 10.37 |  |  |
| 31.32 | 21.48 | 19.33 |  |  | 32.42 | 2.68 | 10.34 |  |  |
| 31.33 | 21.46 | 19.30 |  |  | 32.43 | 2.67 | 10.31 |  |  |
| 31.34 | 21.42 | 19.27 |  |  | 32.44 | 2.66 | 10.29 |  |  |
| 31.35 | 21.39 | 19.25 |  |  | 32.45 | 2.64 | 10.26 |  |  |
| 31.36 | 21.36 | 19.22 |  |  | 32.46 | 2.64 | 10.23 |  |  |
| 31.37 | 21.33 | 19.19 |  |  | 32.47 | 2.63 | 10.20 |  |  |
| 31.38 | 21.29 | 19.16 |  |  | 32.48 | 2.61 | 10.18 |  |  |
| 31.39 | 21.27 | 19.14 |  |  | 32.49 | 2.59 | 10.15 |  |  |
| 31.40 | 21.25 | 19.11 |  |  | 32.50 | 2.57 | 10.12 |  |  |
| 31.41 | 21.21 | 19.08 |  |  | 32.51 | 2.56 | 10.10 |  |  |
| 31.42 | 21.18 | 19.05 |  |  | 32.52 | 2.55 | 10.07 |  |  |
| 31.43 | 21.13 | 19.02 |  |  | 32.53 | 2.54 | 10.04 |  |  |
| 31.45 | 21.07 | 18.97 |  |  | 32.54 | 2.52 | 10.02 |  |  |
| 31.46 | 21.04 | 18.94 |  |  | 32.56 | 2.49 | 9.99 |  |  |
| 31.47 | 20.96 | 18.91 |  |  | 32.58 | 2.44 | 9.96 |  |  |
| 31.49 | 20.88 | 18.86 |  |  | 32.59 | 2.43 | 9.93 |  |  |
| 31.51 | 20.80 | 18.80 |  |  | 32.60 | 2.42 | 9.91 |  |  |
| 31.52 | 20.75 | 18.77 |  |  | 32.61 | 2.41 | 9.88 |  |  |
| 31.53 | 20.72 | 18.75 |  |  | 32.62 | 2.39 | 9.85 |  |  |
| 31.54 | 20.68 | 18.72 |  |  | 32.63 | 2.38 | 9.83 |  |  |
| 31.55 | 20.61 | 18.69 |  |  | 32.64 | 2.36 | 9.77 |  |  |
| 31.56 | 20.57 | 18.66 |  |  | 32.65 | 2.34 | 9.74 |  |  |
| 31.57 | 20.53 | 18.64 |  |  | 32.66 | 2.33 | 9.69 |  |  |
| 31.58 | 20.50 | 18.61 |  |  | 32.67 | 2.32 | 9.66 |  |  |
| 31.59 | 20.46 | 18.58 |  |  | 32.68 | 2.31 | 9.64 |  |  |
| 31.60 | 20.40 | 18.55 |  |  | 32.69 | 2.29 | 9.61 |  |  |
| 31.61 | 20.37 | 18.52 |  |  | 32.70 | 2.27 | 9.58 |  |  |
| 31.62 | 20.34 | 18.50 |  |  | 32.71 | 2.26 | 9.56 |  |  |
| 31.63 | 20.29 | 18.47 |  |  | 32.72 | 2.24 | 9.47 |  |  |
| 31.64 | 20.25 | 18.44 |  |  | 32.73 | 2.22 | 9.45 |  |  |
| 31.65 | 20.19 | 18.41 |  |  | 32.74 | 2.20 | 9.42 |  |  |
| 31.66 | 20.15 | 18.39 |  |  | 32.75 | 2.17 | 9.39 |  |  |
| 31.67 | 20.12 | 18.36 |  |  | 32.76 | 2.16 | 9.37 |  |  |
| 31.68 | 20.08 | 18.33 |  |  | 32.77 | 2.14 | 9.34 |  |  |
| 31.69 | 20.04 | 18.30 |  |  | 32.78 | 2.13 | 9.31 |  |  |
| 31.70 | 20.00 | 18.27 |  |  | 32.79 | 2.09 | 9.28 |  |  |
| 31.71 | 19.96 | 18.25 |  |  | 32.80 | 2.07 | 9.26 |  |  |
| 31.73 | 19.89 | 18.19 |  |  | 32.81 | 2.06 | 9.23 |  |  |
| 31.74 | 19.86 | 18.16 |  |  | 32.83 | 2.03 | 9.20 |  |  |
| 31.75 | 19.82 | 18.14 |  |  | 32.84 | 2.02 | 9.18 |  |  |
| 31.76 | 19.78 | 18.11 |  |  | 32.85 | 2.00 | 9.15 |  |  |
| 31.77 | 19.73 | 18.08 |  |  | 32.86 | 1.98 | 9.12 |  |  |
| 31.78 | 19.69 | 18.05 |  |  | 32.87 | 1.98 | 9.10 |  |  |
| 31.79 | 19.64 | 18.03 |  |  | 32.88 | 1.96 | 9.07 |  |  |
| 31.80 | 19.59 | 18.00 |  |  | 32.89 | 1.93 | 9.04 |  |  |
| 31.81 | 19.53 | 17.97 |  |  | 32.90 | 1.90 | 9.01 |  |  |
| 31.82 | 19.49 | 17.94 |  |  | 32.91 | 1.88 | 8.99 |  |  |
| 31.84 | 19.39 | 17.89 |  |  | 32.92 | 1.86 | 8.96 |  |  |
| 31.85 | 19.33 | 17.86 |  |  | 32.93 | 1.86 | 8.93 |  |  |
| 31.86 | 19.30 | 17.83 |  |  | 32.94 | 1.83 | 8.91 |  |  |
| 31.87 | 19.27 | 17.80 |  |  | 32.95 | 1.81 | 8.88 |  |  |
| 31.88 | 19.21 | 17.78 |  |  | 32.96 | 1.79 | 8.85 |  |  |
| 31.89 | 19.17 | 17.75 |  |  | 32.97 | 1.78 | 8.82 |  |  |
| 31.90 | 19.13 | 17.72 |  |  | 32.98 | 1.75 | 8.80 |  |  |
| 31.91 | 19.12 | 17.69 |  |  | 32.99 | 1.74 | 8.74 |  |  |
| 31.92 | 19.08 | 17.66 |  |  | 33.00 | 1.71 | 8.72 |  |  |
| 31.93 | 19.03 | 17.64 |  |  | 33.01 | 1.69 | 8.69 |  |  |
| 31.94 | 18.96 | 17.61 |  |  | 33.02 | 1.66 | 8.64 |  |  |
| 31.95 | 18.91 | 17.58 |  |  | 33.03 | 1.63 | 8.61 |  |  |
| 31.96 | 18.87 | 17.55 |  |  | 33.04 | 1.62 | 8.58 |  |  |
| 31.97 | 18.84 | 17.53 |  |  | 33.05 | 1.61 | 8.55 |  |  |
| 31.98 | 18.79 | 17.50 |  |  | 33.06 | 1.60 | 8.53 |  |  |
| 31.99 | 18.75 | 17.47 |  |  | 33.07 | 1.59 | 8.50 |  |  |
| 32.00 | 18.73 | 17.44 |  |  | 33.08 | 1.57 | 8.47 |  |  |
| 32.01 | 18.69 | 17.41 |  |  | 33.09 | 1.55 | 8.45 |  |  |
| 32.02 | 18.65 | 17.39 |  |  | 33.10 | 1.54 | 8.42 |  |  |
| 32.03 | 18.62 | 17.36 |  |  | 33.11 | 1.52 | 8.39 |  |  |
| 32.04 | 18.56 | 17.33 |  |  | 33.12 | 1.51 | 8.36 |  |  |
| 32.05 | 18.53 | 17.30 |  |  | 33.13 | 1.48 | 8.31 |  |  |
| 32.06 | 18.49 | 17.28 |  |  | 33.14 | 1.48 | 8.28 |  |  |
| 32.08 | 18.42 | 17.22 |  |  | 33.15 | 1.46 | 8.26 |  |  |
| 32.09 | 18.39 | 17.19 |  |  | 33.16 | 1.44 | 8.23 |  |  |
| 32.10 | 18.36 | 17.16 |  |  | 33.17 | 1.43 | 8.20 |  |  |
| 32.11 | 18.32 | 17.14 |  |  | 33.18 | 1.41 | 8.18 |  |  |
| 32.12 | 18.29 | 17.11 |  |  | 33.19 | 1.39 | 8.15 |  |  |
| 32.13 | 18.24 | 17.08 |  |  | 33.20 | 1.38 | 8.12 |  |  |
| 32.15 | 18.14 | 17.03 |  |  | 33.21 | 1.37 | 8.09 |  |  |
| 32.16 | 18.10 | 17.00 |  |  | 33.22 | 1.35 | 8.07 |  |  |
| 32.17 | 18.06 | 16.97 |  |  | 33.23 | 1.34 | 8.04 |  |  |
| 32.18 | 18.02 | 16.94 |  |  | 33.24 | 1.31 | 8.01 |  |  |
| 32.19 | 17.97 | 16.91 |  |  | 33.26 | 1.28 | 7.99 |  |  |
| 32.20 | 17.92 | 16.89 |  |  | 33.27 | 1.27 | 7.96 |  |  |
| 32.21 | 17.88 | 16.86 |  |  | 33.28 | 1.25 | 7.93 |  |  |
| 32.22 | 17.81 | 16.83 |  |  | 33.30 | 1.22 | 7.90 |  |  |
| 32.23 | 17.75 | 16.80 |  |  | 33.31 | 1.19 | 7.88 |  |  |
| 32.24 | 17.71 | 16.78 |  |  | 33.32 | 1.18 | 7.85 |  |  |
| 32.25 | 17.68 | 16.75 |  |  | 33.33 | 1.16 | 7.82 |  |  |
| 32.26 | 17.63 | 16.72 |  |  | 33.34 | 1.15 | 7.80 |  |  |
| 32.27 | 17.58 | 16.69 |  |  | 33.35 | 1.13 | 7.77 |  |  |
| 32.28 | 17.53 | 16.66 |  |  | 33.37 | 1.10 | 7.74 |  |  |
| 32.29 | 17.48 | 16.64 |  |  | 33.38 | 1.09 | 7.72 |  |  |
| 32.30 | 17.44 | 16.61 |  |  | 33.40 | 1.06 | 7.69 |  |  |
| 32.31 | 17.39 | 16.58 |  |  | 33.41 | 1.03 | 7.66 |  |  |
| 32.32 | 17.35 | 16.55 |  |  | 33.42 | 1.01 | 7.63 |  |  |
| 32.33 | 17.31 | 16.53 |  |  | 33.43 | 0.99 | 7.61 |  |  |
| 32.34 | 17.26 | 16.50 |  |  | 33.44 | 0.97 | 7.58 |  |  |
| 32.35 | 17.21 | 16.47 |  |  | 33.45 | 0.95 | 7.55 |  |  |
| 32.36 | 17.16 | 16.44 |  |  | 33.46 | 0.95 | 7.53 |  |  |
| 32.37 | 17.13 | 16.42 |  |  | 33.47 | 0.93 | 7.47 |  |  |
| 32.38 | 17.09 | 16.39 |  |  | 33.48 | 0.91 | 7.44 |  |  |
| 32.39 | 17.05 | 16.36 |  |  | 33.49 | 0.90 | 7.42 |  |  |
| 32.40 | 17.01 | 16.33 |  |  | 33.50 | 0.87 | 7.39 |  |  |
| 32.41 | 16.98 | 16.30 |  |  | 33.51 | 0.86 | 7.36 |  |  |
| 32.43 | 16.89 | 16.25 |  |  | 33.52 | 0.84 | 7.34 |  |  |
| 32.44 | 16.86 | 16.22 |  |  | 33.53 | 0.83 | 7.31 |  |  |
| 32.45 | 16.82 | 16.19 |  |  | 33.54 | 0.81 | 7.28 |  |  |
| 32.46 | 16.78 | 16.17 |  |  | 33.55 | 0.80 | 7.26 |  |  |
| 32.47 | 16.74 | 16.14 |  |  | 33.56 | 0.79 | 7.23 |  |  |
| 32.48 | 16.68 | 16.11 |  |  | 33.57 | 0.77 | 7.20 |  |  |
| 32.49 | 16.63 | 16.08 |  |  | 33.58 | 0.77 | 7.17 |  |  |
| 32.50 | 16.59 | 16.05 |  |  | 33.59 | 0.76 | 7.15 |  |  |
| 32.51 | 16.53 | 16.03 |  |  | 33.60 | 0.74 | 7.12 |  |  |
| 32.52 | 16.50 | 16.00 |  |  | 33.61 | 0.73 | 7.09 |  |  |
| 32.53 | 16.45 | 15.97 |  |  | 33.62 | 0.72 | 7.07 |  |  |
| 32.54 | 16.40 | 15.94 |  |  | 33.64 | 0.69 | 7.01 |  |  |
| 32.55 | 16.37 | 15.92 |  |  | 33.66 | 0.66 | 6.98 |  |  |
| 32.56 | 16.33 | 15.89 |  |  | 33.68 | 0.63 | 6.90 |  |  |
| 32.57 | 16.29 | 15.86 |  |  | 33.69 | 0.62 | 6.88 |  |  |
| 32.58 | 16.24 | 15.83 |  |  | 33.70 | 0.61 | 6.85 |  |  |
| 32.59 | 16.19 | 15.80 |  |  | 33.71 | 0.59 | 6.82 |  |  |
| 32.60 | 16.14 | 15.78 |  |  | 33.72 | 0.57 | 6.80 |  |  |
| 32.61 | 16.10 | 15.75 |  |  | 33.73 | 0.55 | 6.77 |  |  |
| 32.62 | 16.06 | 15.72 |  |  | 33.74 | 0.53 | 6.74 |  |  |
| 32.64 | 15.98 | 15.67 |  |  | 33.76 | 0.50 | 6.71 |  |  |
| 32.65 | 15.94 | 15.64 |  |  | 33.77 | 0.48 | 6.69 |  |  |
| 32.66 | 15.89 | 15.61 |  |  | 33.78 | 0.46 | 6.66 |  |  |
| 32.67 | 15.84 | 15.58 |  |  | 33.79 | 0.44 | 6.61 |  |  |
| 32.68 | 15.79 | 15.55 |  |  | 33.80 | 0.43 | 6.58 |  |  |
| 32.69 | 15.75 | 15.53 |  |  | 33.81 | 0.42 | 6.55 |  |  |
| 32.70 | 15.70 | 15.50 |  |  | 33.82 | 0.41 | 6.52 |  |  |
| 32.71 | 15.66 | 15.47 |  |  | 33.83 | 0.41 | 6.50 |  |  |
| 32.72 | 15.62 | 15.44 |  |  | 33.84 | 0.40 | 6.47 |  |  |
| 32.73 | 15.58 | 15.42 |  |  | 33.85 | 0.39 | 6.44 |  |  |
| 32.74 | 15.53 | 15.39 |  |  | 33.86 | 0.39 | 6.42 |  |  |
| 32.75 | 15.48 | 15.36 |  |  | 33.87 | 0.38 | 6.39 |  |  |
| 32.76 | 15.44 | 15.33 |  |  | 33.88 | 0.37 | 6.36 |  |  |
| 32.77 | 15.39 | 15.30 |  |  | 33.89 | 0.36 | 6.34 |  |  |
| 32.78 | 15.35 | 15.28 |  |  | 33.92 | 0.32 | 6.28 |  |  |
| 32.79 | 15.29 | 15.25 |  |  | 33.93 | 0.31 | 6.25 |  |  |
| 32.80 | 15.26 | 15.22 |  |  | 33.94 | 0.30 | 6.17 |  |  |
| 32.81 | 15.21 | 15.19 |  |  | 33.95 | 0.29 | 6.15 |  |  |
| 32.82 | 15.18 | 15.17 |  |  | 33.96 | 0.27 | 6.12 |  |  |
| 32.83 | 15.15 | 15.14 |  |  | 33.97 | 0.27 | 6.09 |  |  |
| 32.84 | 15.08 | 15.11 |  |  | 33.98 | 0.26 | 6.06 |  |  |
| 32.85 | 15.03 | 15.08 |  |  | 33.99 | 0.26 | 6.04 |  |  |
| 32.86 | 14.98 | 15.05 |  |  | 34.00 | 0.26 | 6.01 |  |  |
| 32.87 | 14.95 | 15.03 |  |  | 34.01 | 0.25 | 5.98 |  |  |
| 32.88 | 14.90 | 15.00 |  |  | 34.02 | 0.24 | 5.96 |  |  |
| 32.89 | 14.86 | 14.97 |  |  | 34.03 | 0.24 | 5.93 |  |  |
| 32.90 | 14.82 | 14.94 |  |  | 34.04 | 0.24 | 5.90 |  |  |
| 32.91 | 14.78 | 14.92 |  |  | 34.05 | 0.22 | 5.88 |  |  |
| 32.92 | 14.76 | 14.89 |  |  | 34.06 | 0.21 | 5.85 |  |  |
| 32.93 | 14.71 | 14.86 |  |  | 34.07 | 0.21 | 5.82 |  |  |
| 32.94 | 14.66 | 14.83 |  |  | 34.08 | 0.20 | 5.79 |  |  |
| 32.95 | 14.62 | 14.80 |  |  | 34.10 | 0.19 | 5.77 |  |  |
| 32.97 | 14.51 | 14.75 |  |  | 34.11 | 0.19 | 5.74 |  |  |
| 32.98 | 14.46 | 14.72 |  |  | 34.12 | 0.18 | 5.71 |  |  |
| 32.99 | 14.42 | 14.69 |  |  | 34.15 | 0.15 | 5.69 |  |  |
| 33.00 | 14.39 | 14.67 |  |  | 34.16 | 0.15 | 5.66 |  |  |
| 33.01 | 14.35 | 14.64 |  |  | 34.17 | 0.15 | 5.63 |  |  |
| 33.02 | 14.31 | 14.61 |  |  | 34.18 | 0.15 | 5.60 |  |  |
| 33.03 | 14.26 | 14.58 |  |  | 34.19 | 0.14 | 5.58 |  |  |
| 33.04 | 14.20 | 14.56 |  |  | 34.20 | 0.14 | 5.55 |  |  |
| 33.05 | 14.16 | 14.53 |  |  | 34.21 | 0.14 | 5.52 |  |  |
| 33.06 | 14.11 | 14.50 |  |  | 34.22 | 0.13 | 5.50 |  |  |
| 33.07 | 14.05 | 14.47 |  |  | 34.23 | 0.13 | 5.47 |  |  |
| 33.08 | 14.00 | 14.44 |  |  | 34.24 | 0.12 | 5.44 |  |  |
| 33.10 | 13.89 | 14.39 |  |  | 34.27 | 0.12 | 5.42 |  |  |
| 33.11 | 13.82 | 14.36 |  |  | 34.28 | 0.12 | 5.39 |  |  |
| 33.12 | 13.78 | 14.33 |  |  | 34.29 | 0.11 | 5.36 |  |  |
| 33.13 | 13.71 | 14.31 |  |  | 34.30 | 0.11 | 5.31 |  |  |
| 33.14 | 13.68 | 14.28 |  |  | 34.32 | 0.10 | 5.28 |  |  |
| 33.15 | 13.63 | 14.25 |  |  | 34.33 | 0.10 | 5.25 |  |  |
| 33.16 | 13.58 | 14.22 |  |  | 34.34 | 0.09 | 5.23 |  |  |
| 33.17 | 13.51 | 14.19 |  |  | 34.35 | 0.09 | 5.20 |  |  |
| 33.18 | 13.47 | 14.17 |  |  | 34.36 | 0.09 | 5.17 |  |  |
| 33.19 | 13.43 | 14.14 |  |  | 34.37 | 0.09 | 5.15 |  |  |
| 33.20 | 13.40 | 14.11 |  |  | 34.38 | 0.08 | 5.12 |  |  |
| 33.21 | 13.36 | 14.08 |  |  | 34.40 | 0.08 | 5.09 |  |  |
| 33.22 | 13.30 | 14.06 |  |  | 34.42 | 0.07 | 5.06 |  |  |
| 33.23 | 13.24 | 14.03 |  |  | 34.43 | 0.06 | 5.04 |  |  |
| 33.24 | 13.21 | 14.00 |  |  | 34.44 | 0.06 | 5.01 |  |  |
| 33.25 | 13.15 | 13.97 |  |  | 34.45 | 0.06 | 4.98 |  |  |
| 33.26 | 13.11 | 13.94 |  |  | 34.47 | 0.06 | 4.96 |  |  |
| 33.27 | 13.06 | 13.92 |  |  | 34.48 | 0.06 | 4.93 |  |  |
| 33.28 | 12.99 | 13.89 |  |  | 34.50 | 0.06 | 4.90 |  |  |
| 33.29 | 12.92 | 13.86 |  |  | 34.51 | 0.05 | 4.87 |  |  |
| 33.30 | 12.88 | 13.83 |  |  | 34.52 | 0.05 | 4.82 |  |  |
| 33.31 | 12.83 | 13.81 |  |  | 34.53 | 0.05 | 4.79 |  |  |
| 33.32 | 12.76 | 13.78 |  |  | 34.55 | 0.04 | 4.77 |  |  |
| 33.33 | 12.72 | 13.75 |  |  | 34.58 | 0.04 | 4.74 |  |  |
| 33.34 | 12.66 | 13.72 |  |  | 34.59 | 0.03 | 4.71 |  |  |
| 33.35 | 12.60 | 13.69 |  |  | 34.60 | 0.03 | 4.69 |  |  |
| 33.36 | 12.54 | 13.67 |  |  | 34.61 | 0.03 | 4.66 |  |  |
| 33.37 | 12.49 | 13.64 |  |  | 34.62 | 0.03 | 4.63 |  |  |
| 33.38 | 12.47 | 13.61 |  |  | 34.63 | 0.03 | 4.60 |  |  |
| 33.39 | 12.42 | 13.58 |  |  | 34.64 | 0.03 | 4.58 |  |  |
| 33.40 | 12.36 | 13.56 |  |  | 34.65 | 0.02 | 4.55 |  |  |
| 33.41 | 12.30 | 13.53 |  |  | 34.67 | 0.02 | 4.52 |  |  |
| 33.42 | 12.24 | 13.50 |  |  | 34.68 | 0.01 | 4.50 |  |  |
| 33.43 | 12.19 | 13.47 |  |  | 34.69 | 0.01 | 4.47 |  |  |
| 33.44 | 12.14 | 13.44 |  |  | 34.70 | 0.01 | 4.44 |  |  |
| 33.45 | 12.10 | 13.42 |  |  | 34.71 | 0.01 | 4.41 |  |  |
| 33.46 | 12.05 | 13.39 |  |  | 34.72 | 0.01 | 4.39 |  |  |
| 33.47 | 12.01 | 13.36 |  |  | 34.74 | 0.01 | 4.36 |  |  |
| 33.48 | 11.99 | 13.33 |  |  | 34.75 | 0.01 | 4.33 |  |  |
| 33.49 | 11.93 | 13.31 |  |  | 34.78 | 0.01 | 4.31 |  |  |
| 33.50 | 11.89 | 13.28 |  |  | 34.79 | 0.00 | 4.28 |  |  |
| 33.51 | 11.85 | 13.25 |  |  | 34.81 | 0.00 | 4.25 |  |  |
| 33.52 | 11.82 | 13.22 |  |  | 34.82 | 0.00 | 4.23 |  |  |
| 33.53 | 11.75 | 13.19 |  |  | 38.91 | 0.00 | 4.20 |  |  |
| 33.55 | 11.63 | 13.14 |  |  | 38.91 | 0.00 | 4.17 |  |  |
| 33.56 | 11.59 | 13.11 |  |  |  |  |  |  |  |
| 33.57 | 11.54 | 13.08 |  |  |  |  |  |  |  |
| 33.58 | 11.49 | 13.06 |  |  |  |  |  |  |  |
| 33.60 | 11.39 | 13.00 |  |  |  |  |  |  |  |

### Data used to calculate results in Fig 4 and Table 3.

**S3 Table**

| **#** | ***IR_(±3mm/0mm)_*** | | ***ORI*** | |  |
| --- | --- | --- | --- | --- | --- |
|  | **Static** | **Dynamic** | **Static** | **Dynamic** | |
| 1 | 1.306 | 1.221 | 0.352 | 0.568 | |
| 2 | 1.310 | 1.190 | 0.281 | 0.473 | |
| 3 | 1.095 | 1.098 | 0.773 | 0.838 | |
| 4 | 1.195 | 1.200 | 0.393 | 0.503 | |
| 5 | 1.125 | 1.091 | 0.680 | 0.930 | |
| 6 | 1.129 | 1.126 | 0.720 | 0.709 | |
| 7 | 1.327 | 1.217 | 0.241 | 0.442 | |
| 8 | 1.247 | 1.179 | 0.325 | 0.433 | |
| 9 | 1.154 | 1.150 | 0.584 | 0.621 | |
| 10 | 1.280 | 1.169 | 0.366 | 0.640 | |

### Data used to calculate results in Table 4 and present results in Fig 5.

**S4 Table**

|  | **Film** | | | | **Delta 4** | | | |
| --- | --- | --- | --- | --- | --- | --- | --- | --- |
| **Criteria** | **US** | **MS** | **UD** | **MD** | **US** | **MS** | **UD** | **MD** |
| 3%/1mm | 91.99 | 84.16 | 89.64 | 84.41 | 91.95 | 84.34 | 99.67 | 99.74 |
| 2%/2mm | 91.99 | 87.51 | 93.64 | 95.59 | 99.71 | 99.40 | 100.00 | 100.00 |
| 3%/2mm | 96.23 | 95.27 | 95.44 | 98.02 | 100.00 | 100.00 | 100.00 | 100.00 |
| 3%/3mm | 98.69 | 98.31 | 97.08 | 99.12 | 100.00 | 100.00 | 100.00 | 100.00 |

### Data used in Fig 7.

**S5 Table**

|  | **US** | | | | **UD** | | |
| --- | --- | --- | --- | --- | --- | --- | --- |
| **Coordinates**  **(mm)** | | **Film (Gy)** | **TPS (Gy)** | **Difference**  **(%)** | **Film (Gy)** | **TPS (Gy)** | **Difference**  **(%)** |
| 7.50 | | 1.09 | 1.15 | 4.98 | 1.45 | 1.48 | 1.66 |
| 8.00 | | 1.11 | 1.15 | 3.16 | 1.45 | 1.47 | 1.64 |
| 8.50 | | 1.12 | 1.14 | 1.92 | 1.43 | 1.47 | 2.77 |
| 9.00 | | 1.13 | 1.14 | 1.27 | 1.45 | 1.46 | 1.21 |
| 9.50 | | 1.13 | 1.14 | 1.55 | 1.45 | 1.47 | 0.94 |
| 10.00 | | 1.11 | 1.14 | 2.44 | 1.45 | 1.46 | 1.15 |
| 10.50 | | 1.10 | 1.13 | 2.42 | 1.44 | 1.47 | 1.71 |
| 11.00 | | 1.11 | 1.13 | 1.83 | 1.44 | 1.47 | 1.84 |
| 11.50 | | 1.11 | 1.12 | 1.50 | 1.43 | 1.47 | 2.38 |
| 12.00 | | 1.10 | 1.12 | 1.34 | 1.43 | 1.47 | 2.56 |
| 12.50 | | 1.10 | 1.11 | 1.21 | 1.43 | 1.46 | 2.11 |
| 13.00 | | 1.10 | 1.11 | 0.93 | 1.43 | 1.46 | 2.31 |
| 13.50 | | 1.09 | 1.11 | 1.68 | 1.43 | 1.46 | 2.16 |
| 14.00 | | 1.08 | 1.10 | 1.80 | 1.42 | 1.46 | 2.31 |
| 14.50 | | 1.08 | 1.10 | 2.27 | 1.42 | 1.46 | 2.62 |
| 15.00 | | 1.07 | 1.10 | 2.46 | 1.42 | 1.46 | 2.37 |
| 15.50 | | 1.07 | 1.09 | 1.60 | 1.41 | 1.46 | 3.31 |
| 16.00 | | 1.07 | 1.09 | 2.06 | 1.41 | 1.46 | 3.46 |
| 16.50 | | 1.07 | 1.09 | 1.90 | 1.41 | 1.45 | 2.86 |
| 17.00 | | 1.07 | 1.09 | 1.85 | 1.41 | 1.45 | 2.79 |
| 17.50 | | 1.07 | 1.09 | 1.08 | 1.41 | 1.45 | 2.97 |
| 18.00 | | 1.08 | 1.09 | 0.89 | 1.41 | 1.45 | 2.87 |
| 18.50 | | 1.08 | 1.09 | 0.84 | 1.41 | 1.45 | 3.01 |
| 19.00 | | 1.09 | 1.09 | 0.59 | 1.41 | 1.45 | 2.45 |
| 19.50 | | 1.09 | 1.10 | 0.46 | 1.42 | 1.45 | 1.85 |
| 20.00 | | 1.09 | 1.10 | 0.88 | 1.43 | 1.45 | 1.76 |
| 20.50 | | 1.10 | 1.11 | 0.94 | 1.42 | 1.46 | 2.43 |
| 21.00 | | 1.11 | 1.12 | 0.91 | 1.43 | 1.46 | 2.66 |
| 21.50 | | 1.11 | 1.12 | 0.59 | 1.43 | 1.46 | 2.66 |
| 22.00 | | 1.12 | 1.12 | 0.54 | 1.43 | 1.47 | 2.82 |
| 22.50 | | 1.12 | 1.12 | -0.35 | 1.43 | 1.47 | 3.01 |
| 23.00 | | 1.12 | 1.12 | -0.37 | 1.43 | 1.48 | 3.14 |
| 23.50 | | 1.12 | 1.12 | 0.14 | 1.43 | 1.48 | 3.37 |
| 24.00 | | 1.11 | 1.12 | 0.97 | 1.42 | 1.47 | 3.59 |
| 24.50 | | 1.10 | 1.12 | 1.70 | 1.42 | 1.48 | 3.72 |

### Data used in Figs 8a and b.

**S6 Table**

|  | **MS** | | | **MD** | | |
| --- | --- | --- | --- | --- | --- | --- |
| **Coordinates (mm)** | **Film**  **(Gy)** | **TPS**  **(Gy)** | **Difference**  **(%)** | **Film**  **(Gy)** | **TPS**  **(Gy)** | **Difference**  **(%)** |
| 7.50 | 1.70 | 1.69 | -0.81 | 1.74 | 1.76 | 1.00 |
| 8.00 | 1.71 | 1.68 | -1.70 | 1.73 | 1.77 | 1.78 |
| 8.50 | 1.75 | 1.69 | -3.50 | 1.76 | 1.76 | 0.46 |
| 9.00 | 1.75 | 1.69 | -3.70 | 1.75 | 1.76 | 0.65 |
| 9.50 | 1.76 | 1.68 | -4.48 | 1.75 | 1.76 | 0.27 |
| 10.00 | 1.77 | 1.69 | -4.84 | 1.78 | 1.75 | -1.37 |
| 10.50 | 1.78 | 1.69 | -5.16 | 1.78 | 1.75 | -1.66 |
| 11.00 | 1.79 | 1.69 | -5.49 | 1.75 | 1.75 | -0.06 |
| 11.50 | 1.79 | 1.69 | -5.67 | 1.75 | 1.75 | 0.18 |
| 12.00 | 1.78 | 1.69 | -5.03 | 1.77 | 1.75 | -1.25 |
| 12.50 | 1.77 | 1.69 | -4.82 | 1.77 | 1.75 | -1.09 |
| 13.00 | 1.77 | 1.69 | -4.82 | 1.74 | 1.75 | 0.66 |
| 13.50 | 1.77 | 1.69 | -4.58 | 1.72 | 1.75 | 1.45 |
| 14.00 | 1.77 | 1.68 | -4.80 | 1.73 | 1.75 | 1.04 |
| 14.50 | 1.76 | 1.68 | -4.63 | 1.74 | 1.75 | 0.84 |
| 15.00 | 1.76 | 1.69 | -4.05 | 1.75 | 1.75 | 0.09 |
| 15.50 | 1.75 | 1.69 | -3.49 | 1.76 | 1.74 | -0.88 |
| 16.00 | 1.75 | 1.68 | -3.68 | 1.76 | 1.74 | -1.23 |
| 16.50 | 1.75 | 1.68 | -3.87 | 1.75 | 1.74 | -0.47 |
| 17.00 | 1.76 | 1.68 | -4.65 | 1.74 | 1.74 | 0.20 |
| 17.50 | 1.77 | 1.68 | -4.66 | 1.73 | 1.74 | 0.50 |
| 18.00 | 1.76 | 1.69 | -4.08 | 1.74 | 1.74 | 0.16 |
| 18.50 | 1.78 | 1.69 | -5.03 | 1.73 | 1.75 | 0.90 |
| 19.00 | 1.78 | 1.70 | -4.74 | 1.73 | 1.75 | 0.86 |
| 19.50 | 1.77 | 1.71 | -3.73 | 1.73 | 1.75 | 0.76 |
| 20.00 | 1.79 | 1.71 | -4.16 | 1.73 | 1.74 | 0.67 |
| 20.50 | 1.78 | 1.71 | -3.75 | 1.73 | 1.74 | 0.51 |
| 21.00 | 1.77 | 1.71 | -3.18 | 1.72 | 1.73 | 0.38 |
| 21.50 | 1.78 | 1.72 | -3.64 | 1.71 | 1.73 | 1.01 |
| 22.00 | 1.78 | 1.72 | -3.60 | 1.72 | 1.73 | 0.94 |
| 22.50 | 1.76 | 1.72 | -2.34 | 1.71 | 1.74 | 1.65 |
| 23.00 | 1.77 | 1.72 | -2.53 | 1.71 | 1.74 | 1.95 |
| 23.50 | 1.76 | 1.73 | -2.14 | 1.73 | 1.74 | 0.65 |
| 24.00 | 1.76 | 1.73 | -1.53 | 1.72 | 1.74 | 1.18 |
| 24.50 | 1.76 | 1.74 | -1.29 | 1.71 | 1.75 | 2.36 |

### Data used in Figs 8c and d.

**S7 Table**

|  | **US** | | | **UD** | | |
| --- | --- | --- | --- | --- | --- | --- |
| **Coordinates**  **(mm)** | **Delta4**  **(Gy)** | **TPS (Gy)** | **Difference**  **(%)** | **Delta4 (Gy)** | **TPS (Gy)** | **Difference (%)** |
| -25.00 | 0.60 | 0.60 | -1.03 | 0.65 | 0.66 | 0.66 |
| -20.00 | 0.89 | 0.88 | -1.54 | 1.00 | 0.99 | -0.51 |
| -15.00 | 1.13 | 1.11 | -1.93 | 1.24 | 1.23 | -0.43 |
| -10.00 | 1.24 | 1.22 | -1.89 | 1.32 | 1.32 | -0.02 |
| 10.00 | 1.27 | 1.25 | -1.37 | 1.35 | 1.34 | -0.47 |
| 15.00 | 1.21 | 1.20 | -1.42 | 1.30 | 1.29 | -0.54 |
| 20.00 | 1.03 | 1.01 | -2.00 | 1.16 | 1.14 | -0.99 |
| 25.00 | 0.68 | 0.68 | -0.32 | 0.84 | 0.84 | -0.13 |

### Data used in Figs 9a and b.

**S8 Table**

|  | **MS** | | | **MD** | | |
| --- | --- | --- | --- | --- | --- | --- |
| **Coordinates (mm)** | **Delta4**  **(Gy)** | **TPS**  **(Gy)** | **Difference**  **(%)** | **Delta4**  **(Gy)** | **TPS**  **(Gy)** | **Difference**  **(%)** |
| -25.00 | 0.58 | 0.60 | 2.69 | 0.66 | 0.65 | -2.13 |
| -20.00 | 0.80 | 0.84 | 4.17 | 0.96 | 0.94 | -2.01 |
| -15.00 | 0.91 | 0.96 | 5.99 | 1.15 | 1.15 | 0.07 |
| -10.00 | 0.91 | 0.97 | 6.22 | 1.26 | 1.25 | -0.69 |
| 10.00 | 0.93 | 0.96 | 3.78 | 1.27 | 1.27 | 0.00 |
| 15.00 | 0.90 | 0.93 | 3.54 | 1.21 | 1.21 | -0.11 |
| 20.00 | 0.79 | 0.80 | 1.45 | 1.06 | 1.05 | -0.85 |
| 25.00 | 0.54 | 0.55 | 1.74 | 0.74 | 0.73 | -1.32 |

### Data used in Figs 9c and d.
